# New benzoxazole derivatives as potential VEGFR-2 inhibitors and apoptosis inducers: design, synthesis, anti-proliferative evaluation, flowcytometric analysis, and *in silico* studies

**DOI:** 10.1080/14756366.2021.2015343

**Published:** 2021-12-27

**Authors:** Hazem Elkady, Alaa Elwan, Hesham A. El-Mahdy, Ahmed S. Doghish, Ahmed Ismail, Mohammed S. Taghour, Eslam B. Elkaeed, Ibrahim H. Eissa, Mohammed A. Dahab, Hazem A. Mahdy, Mohamed M. Khalifa

**Affiliations:** a Pharmaceutical Medicinal Chemistry & Drug Design Department, Faculty of Pharmacy (Boys), Al-Azhar University, Cairo, Egypt; b Biochemistry and Molecular Biology Department, Faculty of Pharmacy (Boys), Al-Azhar University, Cairo, Egypt; c Biochemistry Department, Faculty of Pharmacy, Badr University in Cairo (BUC), Badr, Egypt; d Department of Pharmaceutical Sciences, College of Pharmacy, AlMaarefa University, Riyadh, Saudi Arabia

**Keywords:** Anti-proliferative, apoptosis, benzoxazole, VEGFR-2 inhibitors

## Abstract

A new series of benzoxazole derivatives were designed and synthesised to have the main essential pharmacophoric features of VEGFR-2 inhibitors. Cytotoxic activities were evaluated for all derivatives against two human cancer cell lines, MCF-7 and HepG2. Also, the effect of the most cytotoxic derivatives on VEGFR-2 protein concentration was assessed by ELISA. Compounds **14o**, **14l**, and **14b** showed the highest activities with VEGFR-2 protein concentrations of 586.3, 636.2, and 705.7 pg/ml, respectively. Additionally, the anti-angiogenic property of compound **14b** against human umbilical vascular endothelial cell (HUVEC) was performed using a wound healing migration assay. Compound **14b** reduced proliferation and migratory potential of HUVEC cells. Furthermore, compound **14b** was subjected to further biological investigations including cell cycle and apoptosis analyses. Compound **14b** arrested the HepG2 cell growth at the Pre-G1 phase and induced apoptosis by 16.52%, compared to 0.67% in the control (HepG2) cells. The effect of apoptosis was buttressed by a 4.8-fold increase in caspase-3 level compared to the control cells. Besides, different *in silico* docking studies were also performed to get better insights into the possible binding mode of the target compounds with VEGFR-2 active sites.

## 1. Introduction

Angiogenesis, a complex process of new blood vessel creation, is crucial for cell development and reproduction^1^^,^^2^. Considering the similar function in cancerous cells, uncontrolled or abnormal angiogenesis has been linked to tumour progression and metastasis^3^. Therefore, finding efficient anti-angiogenesis agents could be considered as a hopeful approach for cancer treatment^4^.

Growth factors, including vascular endothelial growth factors (VEGFs) and their receptors (VEGFRs), control angiogenesis^5–7^. Three main vascular endothelial growth factor receptor subtypes are well-defined namely, VEGFR-1, VEGFR-2, and VEGFR-3^8^. These receptors are the key players' intermediates in controlling tumour angiogenesis and in the development of new blood vessel networks essential to supply nutrition and oxygen for tumour growth^9^. Among the three VEGFRs subtypes, VEGFR-2 plays the most critical role in promoting tumour angiogenesis^10^. Following its activation by VEGF, VEGFR-2 initiates downstream signal transduction *via* dimerisation and then autophosphorylation of tyrosine receptor. These signalling pathways result in tumour angiogenesis^11^. Thus, hindering the VEGF/VEGFR-2 signalling pathway or reducing its response by tyrosine kinases inhibitors (TKIs) is a supreme significant target in anti-angiogenesis therapy against cancer^12^. Over the last decades, several small molecules have been approved for obstructing this critical pathway in angiogenesis^13^^,^^14^. Development of tumour resistance to the effect of the current clinically used small-molecule TKIs opens the door for the investigation of the effectiveness of new chemotypes.

**Figure 1.**
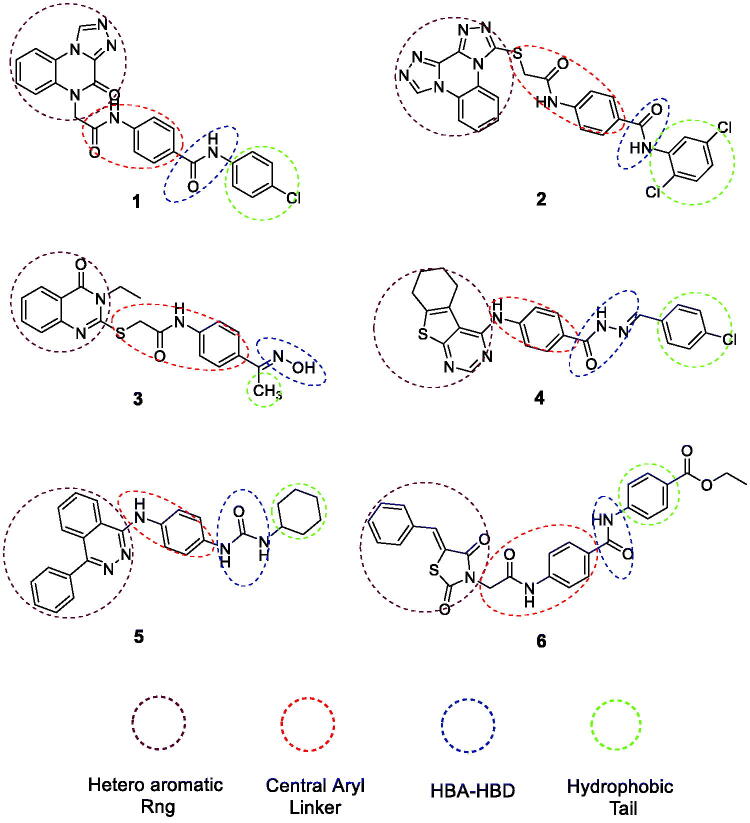
Some reported VEGFR-2 inhibitors with the essential pharmacophoric features of VEGFR-2 inhibitor agents.

Through our trip in finding novel anticancer agents^9^^,^^15–21^, our research team has introduced several small molecules serving as ATP competitive inhibitors of VEGFR-2 depending on different scaffolds including, quinoxaline **1**^22^^,^^23^, bis([1,2,4]triazolo)[4,3-*a*:3′,4′-*c*]quinoxaline **2**^24^, quinazolin-4(3*H*)-one **3**^25^, thieno[2,3-*d*]pyrimidine **4**^26^^,^^27^, 4-phenylphthalazine **5**^28^ and 5-benzylidenethiazolidine-2,4-dione **6**^29^ derivatives. The potency of some derivatives against the VEGFR-2 enzyme exceeded the already marketed VEGFR-2 inhibitors. All these derivatives achieved the basic pharmacophoric requirements needed to fit with the VEGFR-2 active binding site including (a) a “hinge-binding” head segment which is a flat heteroaromatic ring system that occupies the hinge region of ATP binding site^30^, (b) a central aromatic linker to cross the kinase gatekeeper residues linking the hinge-binding segment with the hydrogen-bonding moiety^31^, (c) a “hydrogen-bonding moiety” which interacts *via* hydrogen bonding with Glu883 and/or Asp1044 residues in DFG motif of the enzyme, and (d) a terminal lipophilic tail that occupied the allosteric lipophilic pocket through numerous hydrophobic interactions^32^ (Figure 1).

### 1.1. Rationale of molecular design

**Figure 2.**
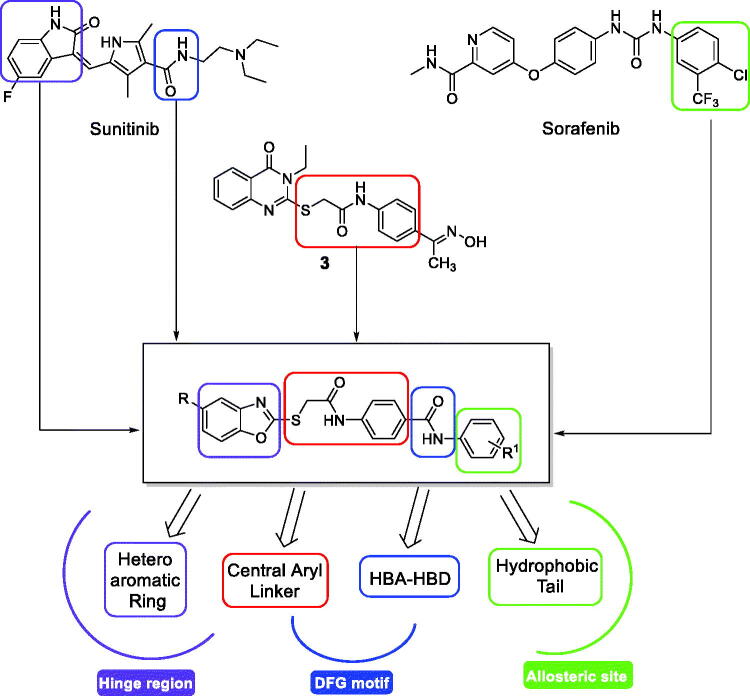
Summary of the proposed VEGFR-2 inhibitors modifications.

In view of the above-mentioned pharmacophoric requirements and depending on our ongoing project to develop novel cytotoxic small molecules based on various chemotypes, it is considered of interest to begin a research work directed towards the design of a new series of anti-angiogenic VEGFR-2 inhibitors. A cross-hybridisation approach between different pharmacophoric elements of the well-known TKIs was the idea of the current study. The approach applied for designing the new target compounds is demonstrated in Figure 2.

Herein, and while conserving the carboxamide moiety of sunitinib to serve as a hydrogen-bonding donor/acceptor moiety, a molecular replacement of the indolinylidene core of sunitinib by a benzoxazole core was performed in the hope of testing the effect of the introduction of another heteroatom to the aromatic scaffold in binding with the hinge region of ATP binding site.

Additionally, the fluorine atom in sunitinib was also replaced either by hydrogen, methyl, or chlorine bioisosteres. On the other side, the terminal phenyl ring of sorafenib was kept playing its key role of occupying the allosteric lipophilic pocket. Contrariwise, the 4-chlorine atom and the 3-trifluoro methyl group of sorafenib were replaced by different substituents as represented in the target compounds. Regarding the central aromatic linker moiety, we found that our previously reported linker of compound **3**^25^ could give the opportunity to the designed compounds to be oriented into the DFG motif and allosteric binding site as well (Figure 2).

The wide diversity of modifications enabled us to study the SAR of the designed candidates as potent anti-proliferative agents with potential VEGFR-2 inhibitory effects. To confirm such a design, *in silico* molecular docking studies of the designed compounds were performed against the prospective biological target (VEGFR-2).

## 2. Results and discussion

### 2.1. Chemistry

**Scheme 1.**
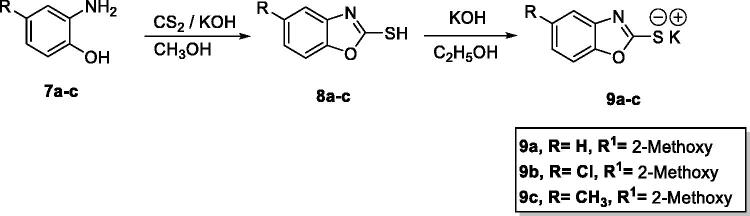
General synthetic route of target salts **9a–c**.

**Scheme 2.**
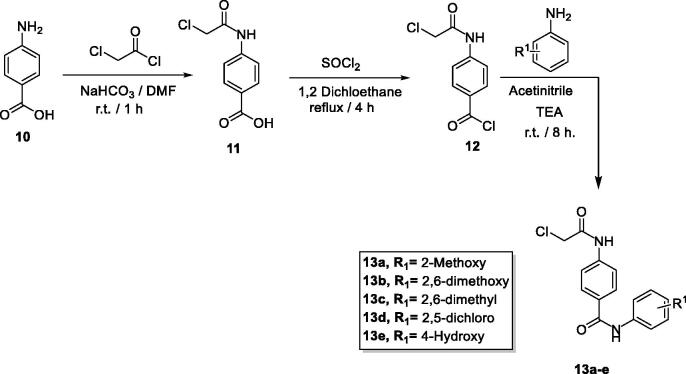
General synthetic route of target intermediates **13a–e**.

**Scheme 3.**
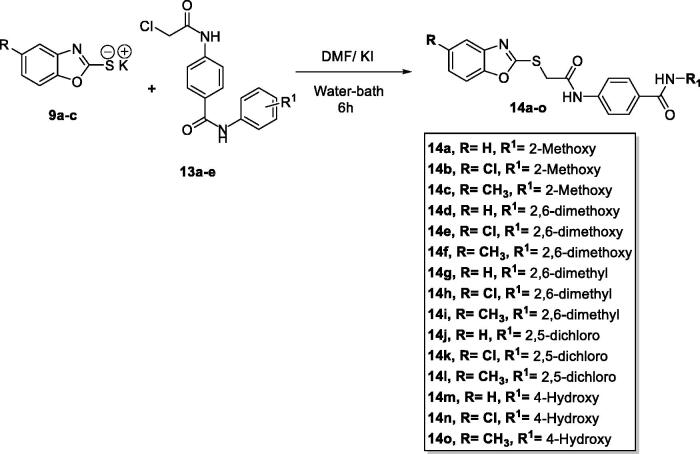
General synthetic route of target final compounds **14a–o**.

The target benzoxazole derivatives **14a–o** were synthesised following the general methodologies outlined in Schemes 1–3. The starting compounds, 2-mercapto-benzoxazoles **8a–c** were synthesised by refluxing the appropriate 2-aminophenol derivatives **7a–c**, carbon disulphide, and potassium hydroxide in methanol following the reported procedure^33^. Then, compounds **8a–c** were treated with alcoholic KOH to afford the corresponding potassium salts, **9a–c** (Scheme 1). On the other hand, 4-aminobenzoic acid **10** was reacted with chloroacetyl chloride in DMF to afford the chloroacetamide intermediate **11**. Acylation of compound **11** was performed using thionyl chloride to yield 4–(2-chloroacetamido)benzoyl chloride **12** as described in the reported procedures^14^^,^^34^. Treating of **12** with commercially available amines namely, 2-methoxyaniline, 2,6-dimethoxyaniline, 2,6-dimethylaniline, 2,4-dichloroaniline, and 4-hydroxyaniline, in acetonitrile containing triethylamine (TEA), afforded the target key intermediates **13a–e** (Scheme 2).

The potassium salts **9a–c** were heated in dry DMF with the formerly prepared derivatives **13a–e** to afford the final target compounds **14a–o**, (Scheme 3).

### 2.2. Biological testing

#### 2.2.1. Breast cancer and hepatocellular carcinoma, *in vitro* anti-proliferative activities

The anti-proliferative activities of the newly synthesised compounds were assessed *in vitro* against two human cancer cell lines namely, breast cancer (MCF-7) and hepatocellular carcinoma (HepG2) cell lines, using the standard MTT method^35^. The tested cell lines were chosen carefully depending on their VEGF overexpression. Sorafenib, the potent VEGFR-2 inhibitor drug, was co-assayed as a positive control. The cytotoxicity results were demonstrated in Table 1. A general observation of the obtained results revealed that all the newly synthesised members had high inhibitory activities towards the two cancer cell lines with IC_50_ values ranging from 4.054 ± 0.17 to 32.53 ± 1.97 µM for MCF-7 and from 3.22 ± 0.13 to 32.11 ± 2.09 µM for HepG2.

**Table 1.** *In vitro* cytotoxic activities of the assessed compounds against MCF-7 and HepG2 cell lines.

Comp. ID	*In vitro* cytotoxicity IC_50_ (µM)	
	MCF-7	HepG2
14a	4.054 ± 0.17	3.95 ± 0.18
14b	4.754 ± 0.21	4.61 ± 0.34
14c	19.36 ± 1.29	20.19 ± 1.82
14d	15.8 ± 1.04	18.47 ± 1.26
14e	16.52 ± 1.18	25 ± 1.47
14f	21.06 ± 1.5	22.05 ± 1.79
14g	5.8 ± 0.22	10.73 ± 0.83
14h	9.44 ± 0.59	19.29 ± 1.07
14i	6.94 ± 0.22	3.22 ± 0.13
14j	14.76 ± 0.99	11.86 ± 0.79
14k	7.75 ± 0.24	11.42 ± 0.93
14l	6.87 ± 0.23	6.70 ± 0.47
14m	32.53 ± 1.97	32.11 ± 2.09
14n	7.098 ± 0.5	9.93 ± 0.85
14o	18.15 ± 1.42	7.01 ± 0.52
Sorafenib	7.28 ± 0.58	3.38 ± 0.21

With reference to their cytotoxic activity, it was noticed that counterparts incorporating 5-chlorobenzo[*d*]oxazole moiety were slightly more advantageous than the unsubstituted benzo[*d*]oxazole analogs. However, the 5-methylbenzo[*d*]oxazole-containing derivatives displayed less potent inhibitory activity against the tested cell lines.

With respect to the 5-chlorobenzo[*d*]oxazole-based members, the best cytotoxic activities against both MCF-7 and HepG2 cell lines appeared with the parent derivative bearing a terminal 2-methoxy phenyl moiety **14b** with IC_50_ values of 4.75 ± 0.21 and 4.61 ± 0.34 µM, respectively. Meanwhile, on 5-chlorobenzo[*d*]oxazole-based derivatives, the 2,5-dichloro phenyl and the 4-hydroxy phenyl containing compounds, **14k** and **14n,** exhibited almost equipotent cytotoxic activity against the tested cell lines (IC_50_ = 7.75 ± 0.24, 11.42 ± 0.93 µM for **14k**, 7.098 ± 0.5 and 9.93 ± 0.85 µM for **14n**). However, the rest of the substituent provided moderate IC_50_ values against the tested cell lines.

As for unsubstituted benzo[*d*]oxazole derivatives, results of the *in vitro* anti-proliferative screening revealed that hybridisation of the nucleus with terminal 2-methoxy phenyl moiety **14a** improved the inhibitory activity against HepG2 (IC_50_ = 3.95 ± 0.18 µM) and MCF-7 (IC_50_ = 4.054 ± 0.17 µM) as well. In addition, derivative **14g**, bearing 2,6-dimethyl phenyl moiety, possessed noticeable inhibitory activity against MCF-7 cell line with IC_50_ of 5.8 ± 0.22 µM with a moderate effect regarding HepG2 cell (IC_50_ = 10.73 ± 0.83 µM). Cytotoxic activities were slightly decreased regarding derivatives bearing terminal 2,5-dichloro phenyl **14j** or 2,6-dimethoxy phenyl **14d** moieties with IC_50_ values ranging from 11.86 ± 0.79 to 18.47 ± 1.26 µM. Substitution of the terminal phenyl ring with 4-hydroxy group **14m** decreased the potency compared to other derivatives.

**Figure 3.**
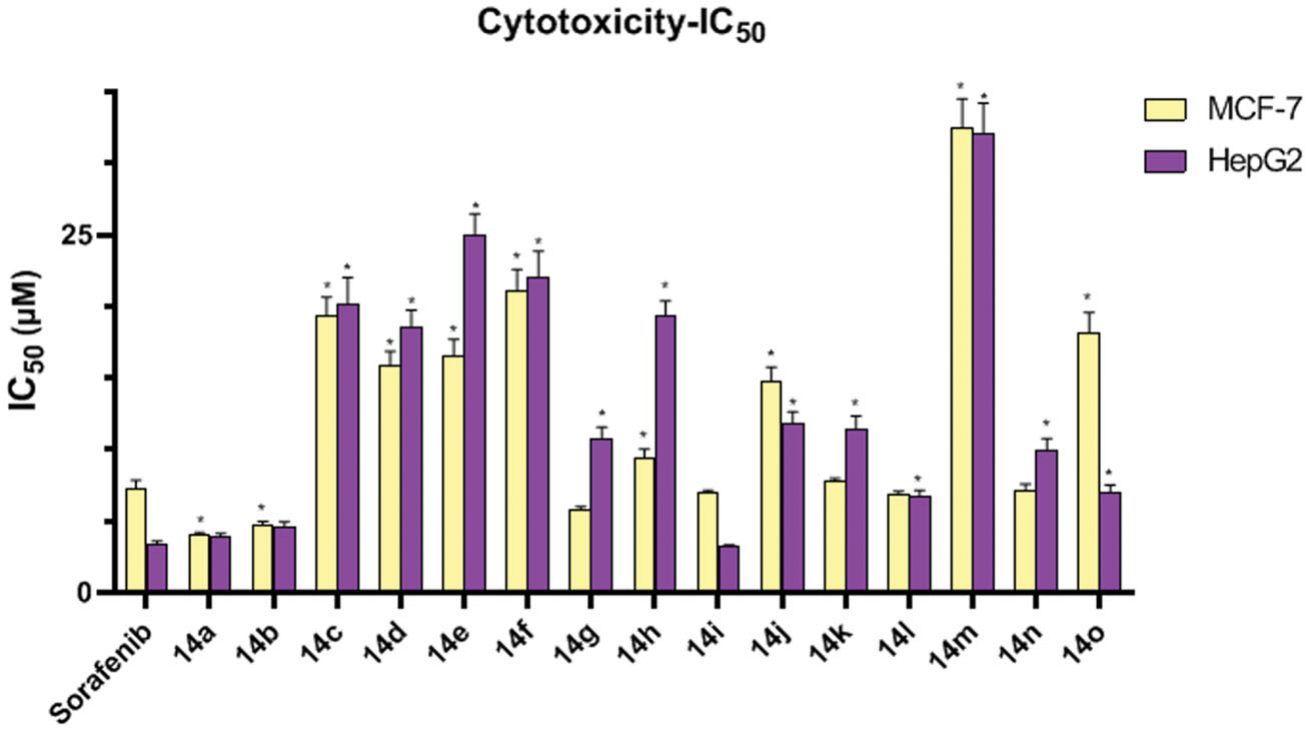
*In vitro* cytotoxic activities of different chemical compounds. *Significant from Sorafenib group at *p* < 0.001.

Lastly in this regard, concerning 5-methylbenzo[*d*]oxazole containing derivatives, it was found that compound **14i** displayed the strongest anti-proliferative effect against HepG2 cell line (IC_50_ = 3.22 ± 0.13 µM) compared to the reference drug, sorafenib. Compound **14i**, moreover, showed a strong effect with respect to MCF-7 cells with IC_50_ of 6.94 ± 0.22 µM. It is also noteworthy that member **14l,** 2,5-dichloro phenyl, strongly inhibited the MCF-7 and HepG2 proliferation with IC_50_ values of 6.87 ± 0.23 and 6.70 ± 0.47 µM, respectively. Other modifications of the terminal phenyl ring did not increase the cytotoxic activity with increasing the IC_50_ range (7.01 ± 0.52 to 22.05 ± 1.79 µM) comparing the other derivatives (Figure 3).

#### 2.2.2. Assessment of VEGFR-2 protein concentration

**Figure 4.**
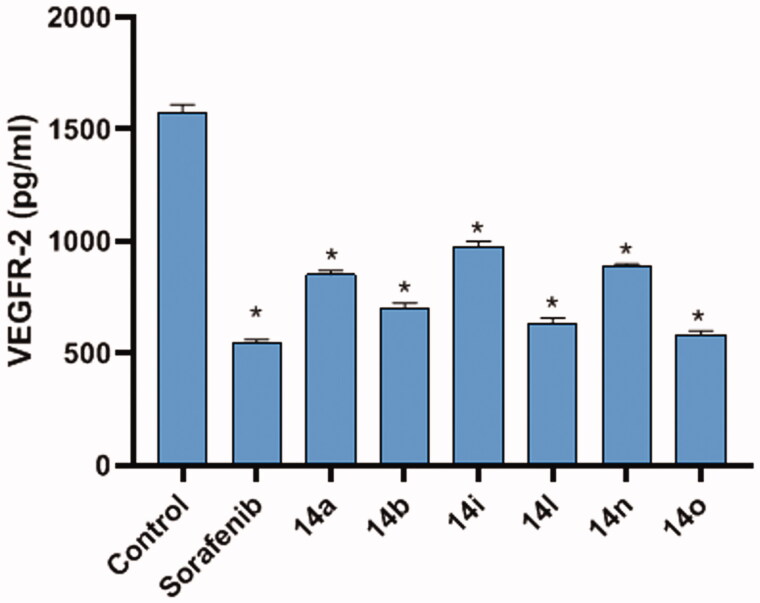
The effect of the most cytotoxic compounds **14b**, **14n**, **14l**, **14i**, **14o**, and **14a** were investigated on VEGFR-2 in HepG2 cells compared to sorafenib as a reference drug. HepG2 cells were treated with sorafenib (3.38 µM), **14b** (4.61 µM), **14n** (9.93 µM), **14l** (6.70 µM), **14i** (3.22 µM), **14o** (7.01 µM), and **14a** (3.95 µM). Data are represented as mean ± SEM of three different experiments. *Significant from the control group at *p*-value <0.001.

The effect of the most cytotoxic compounds **14b, 14n, 14l, 14i, 14o,** and **14a** was investigated on VEGFR-2 in HepG2 cells compared to sorafenib as a reference drug. HepG2 cells were treated with sorafenib (3.38 µM), **14b** (4.61 µM), **14n** (9.93 µM), **14l** (6.70 µM), **14i** (3.22 µM), **14o** (7.01 µM), and **14a** (3.95 µM). The inhibitory effects of the tested compounds on VEGFR-2 protein concentrations were summarised in Table 2 and Figure 4.

**Table 2.** The inhibitory effects of the assessed compounds on VEGFR-2 protein concentration in HepG2 cells compared to Sorafenib.

Comp. ID	VEGFR-2 protein concentration (pg/ml)
	HepG2
Control	1573 ± 36.9
Sorafenib	547.8 ± 13.5
**14a**	852.9 ± 16.3
**14b**	705.7 ± 20.3
**14i**	974.7 ± 25.4
**14l**	636.2 ± 22.4
**14n**	893.3 ± 6.34
**14o**	586.3 ± 16.1

Data are represented as mean ± SEM of three different experiments.

Compound **14o** exhibited the most potent VEGFR-2 inhibitory effect (VEGFR-2 protein concentration = 586.3 ± 16.1 pg/ml**)** which was comparable to that of sorafenib (547.8 pg/ml). Additionally, compounds **14b** and **14l** showed promising effects with VEGFR-2 protein concentrations of 705.7 ± 20.3 and 636.2 ± 22.4 pg/ml, respectively. On the other hand, compounds **14n, 14i,** and **14a** showed moderate to weak effects with VEGFR-2 protein concentrations of 893.3 ± 6.34, 974.7 ± 25.4, and 852.9 ± 16.3 pg/ml, respectively.

#### 2.2.3. Wound healing assay

The compound **14b** reduced human umbilical vascular endothelial cell (HUVEC) proliferation and migratory potential.

**Figure 5.**
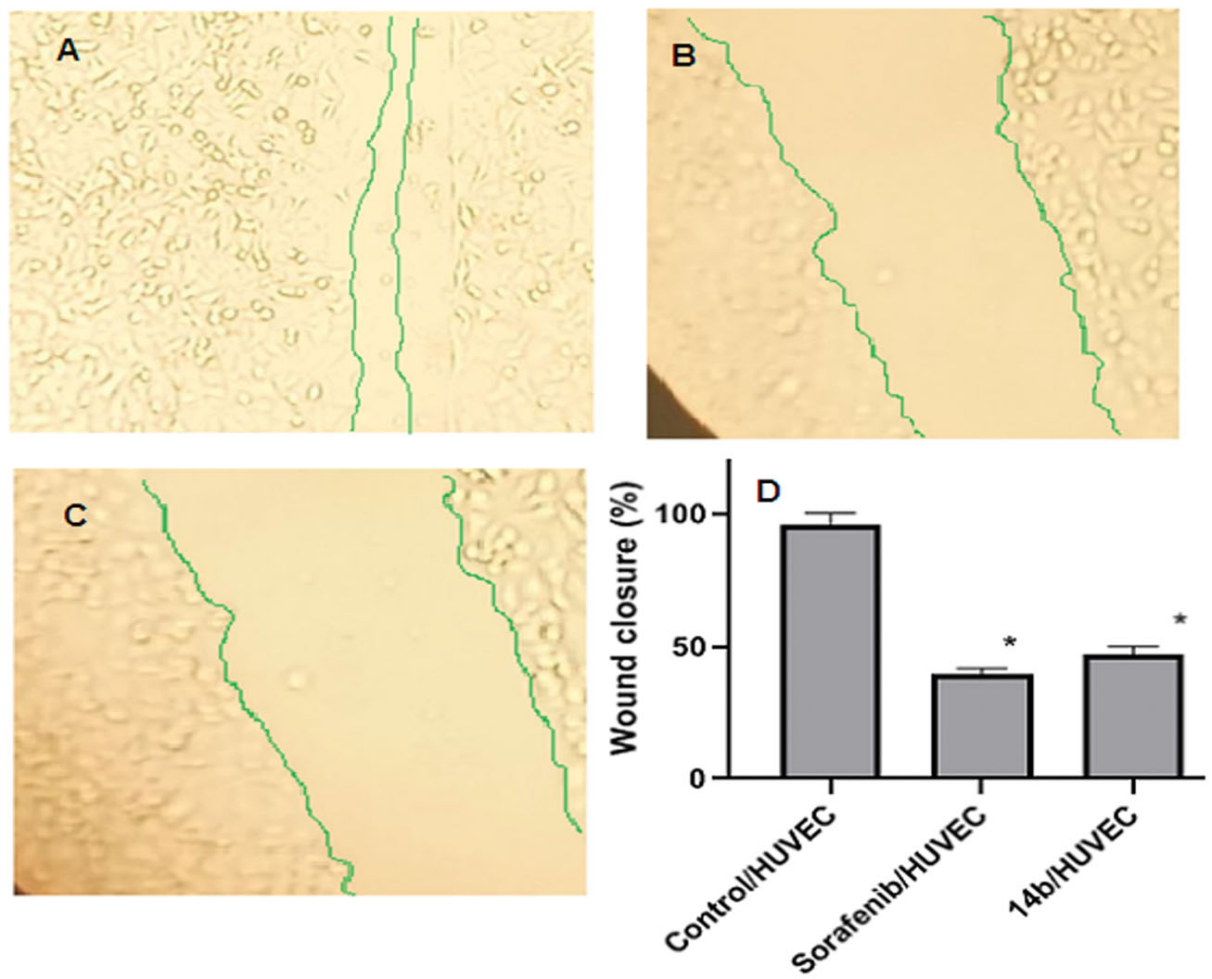
Effects of compound **14b** on endothelial cell migration in HUVEC cells compared to sorafenib. (A) Control/HUVECs, (B) HUVECs were treated with compound **14b** for 72 h. (C) HUVECs were treated with sorafenib for 72 h. (D) Represents the graphical illustration for % of wound closure in control, sorafenib and **14b** treated cells. Data are represented as mean ± SEM of three different experiments. *Significant from the control group at *p* < 0.001.

One of the hallmarks of angiogenesis is cell migration, which happens in the earlier stages of the angiogenic cascade. A wound-healing assay was performed to investigate the migratory effect of compound **14b**. Compound **14b** and sorafenib extremely reduced the HUVECs migration potential exhibiting deeply reduced wound healing patterns after 72 h. Wound closure (%) was significantly lower in the compound **14b** group (47.2 ± 2.88) and sorafenib group (39.8 ± 1.9) when compared to the control group (95.86 ± 4.51) (Figure 5).

#### 2.2.4. Cell cycle analysis

Compound **14b** which demonstrated remarkable cytotoxic potency and significant inhibitory effect against VEGFR-2 was nominated for further cellular mechanistic study. This involved study of its impact on cell cycle progression and induction of apoptosis in HepG2 cells.

**Figure 6.**
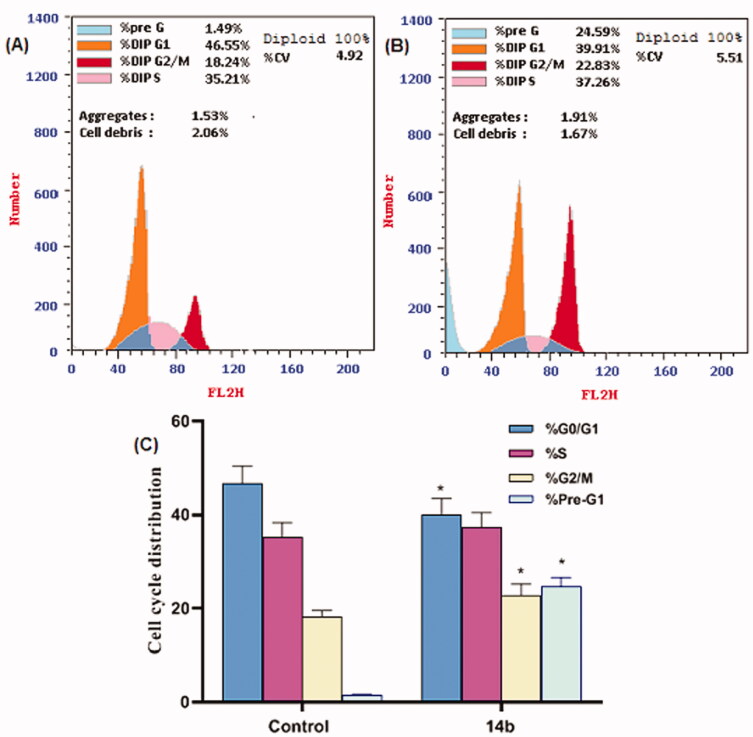
Flow cytometry analysis for cell cycle distribution of HepG2 cells. (A) Control (HepG2 cells), (B) The representative histogram shows the cell cycle distribution of cells treated with **14b**, and (C) Represents the graphical illustration for cell cycle distribution analysis among different treated cells. *Significant from the control group at *p* < 0.001.

The cell cycle process was analysed after exposure of HepG2 cells to **14b** with a concentration of 4.61 µM for 48 h. Flow cytometry data^36^ revealed that the percentage of cells arrested at the Pre-G1 phase increased from 1.49% (in control cells) to 24.59% (in **14b)** treated cells. In addition, the percentage of HepG2 cells mild increased at the S phase from 35.21 to 37.26%. Such findings revealed that compound **14b** arrested the HepG2 cell growth mostly at the Pre-G1 phase (Table 3 and Figure 6).

**Table 3.** Flow cytometry analysis for cell cycle distribution of HepG2 cells.

Comp. ID	%G0/G1	%S	%G2/M	%Pre-G1
Control	46.55 ± 3.82	35.21 ± 3.13	18.24 ± 1.30	1.49 ± 0.12
**14b**	39.91 ± 3.52	37.26 ± 3.22	22.83 ± 2.41	24.59 ± 1.99

Data are represented as mean ± SEM of three different experiments.

#### 2.2.5. Apoptosis analysis

**Figure 7.**
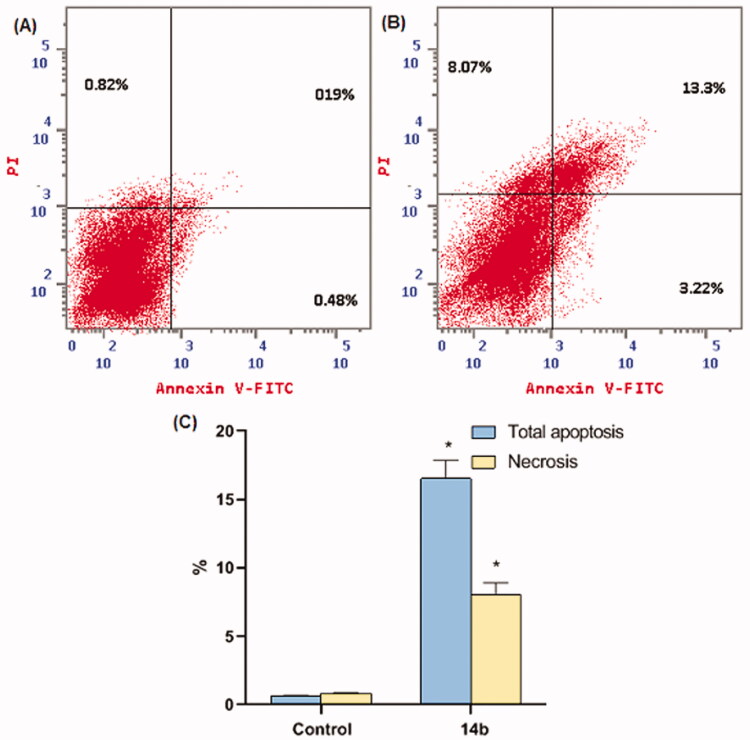
Compound **14b** induced apoptosis in HepG2 cells. (A) Control (HepG2 cells), (B) **14b**, and (C) Represent the graphical illustration for % of apoptotic and necrotic cells among cells among control (HepG2) cells and compound **14b** treated cells. *Significant from the control group at *p* < 0.001.

To quantify the apoptosis triggered by **14b**, Annexin-V/propidium iodide (PI) staining assay was conducted^37^. In such a procedure, compound **14b** at a concentration of 4.61 µM was applied on HepG2 cells for 48 h. As shown in Table 4 and Figure 7, the apoptotic effect of **14b** in HepG2 cells was about twenty-four times more than observed in control cells. In detail, compound **14b** induced apoptosis by 16.52%, compared to 0.67% in the control cells.

**Table 4.** Compound **14b** induced apoptosis in HepG2 cells.

Comp. ID	Apoptosis	Necrosis
	Total	Total
Control	0.67 ± 0.02	0.82 ± 0.074
**14b**	16.52 ± 1.35	8.07 ± 0.82

Percentages are reported as mean ± SEM of three different experiments. *P < 0.001 indicates a statistically significant difference from the corresponding control (HepG2) group.

#### 2.2.6. Caspase-3 determination

**Figure 8.**
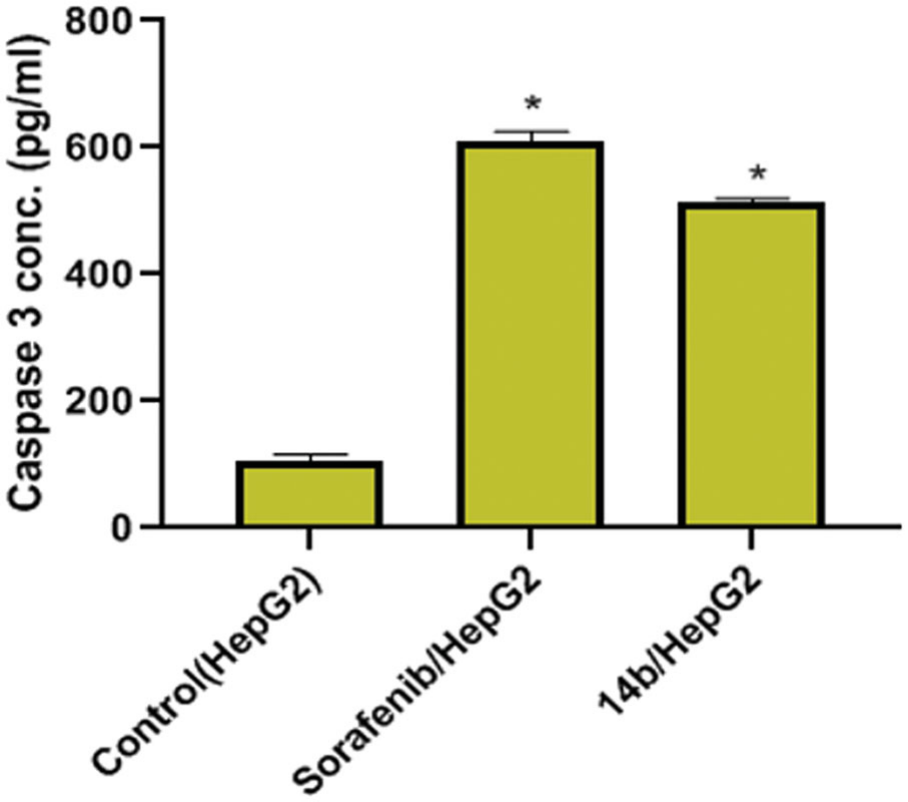
Effects of compound **14b** on Caspase 3 level in HepG2 cells. Values are reported as mean ± SEM of three different experiments. *p < 0.001 indicates statistically significant differences from the control (HepG2) group.

To investigate the effect of the synthesised compounds on caspase-3 level, the most promising member **14b** was applied on the most sensitive cells (HepG2) at a concentration of 4.61 µM for 48 h. The results revealed that compound **14b** produced a significant increase in the level of caspase-3 (4.8-fold) compared to the control (HepG2) cells (Figure 8).

### 2.3. Docking study

**Figure 9.**
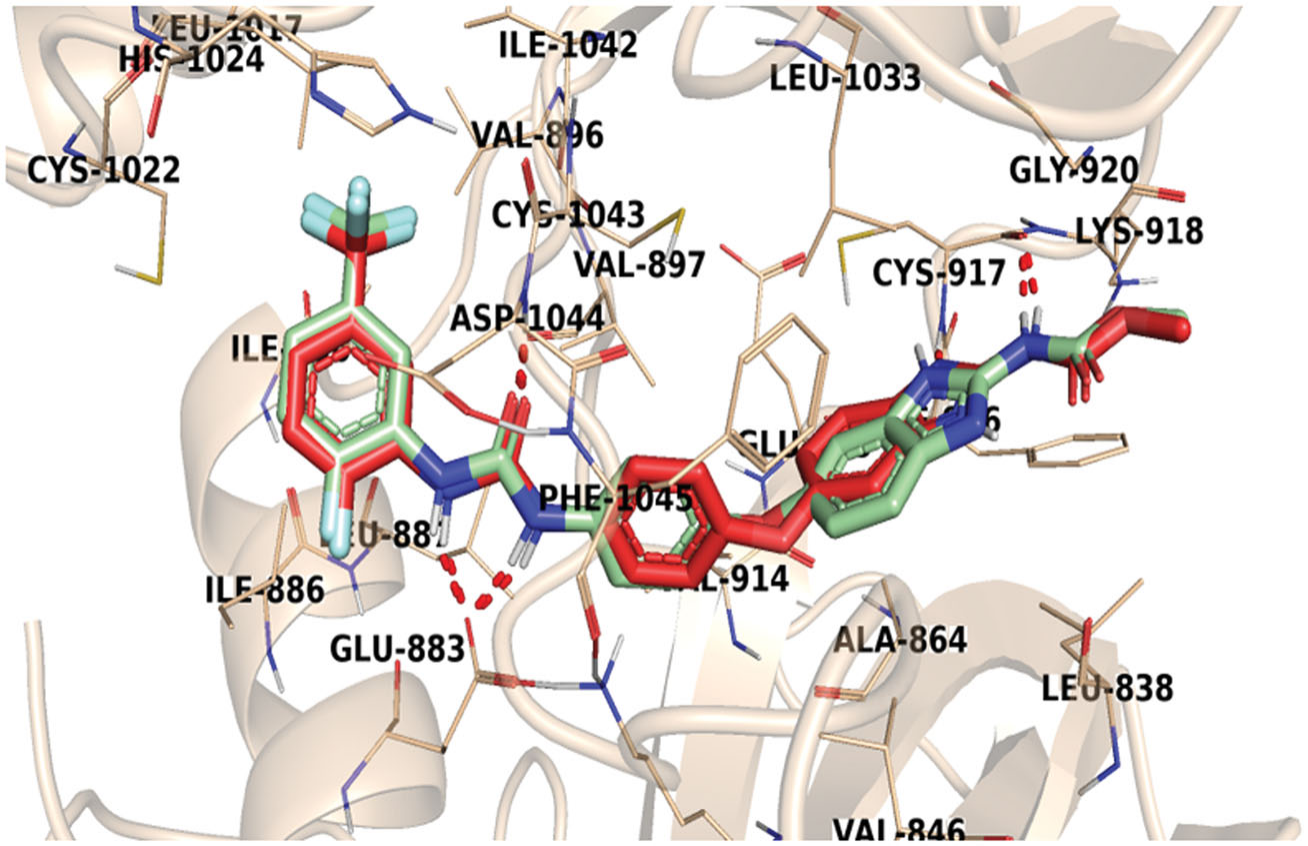
Superimposition of the co-crystallized molecule (mint green) and the docking pose (red) of the same molecule inside the VEGFR-2 kinase active site.

A docking study was carried out in the hope of getting an insight into the mode of interaction of the synthesised compounds to their biomolecular targets^38–40^. Thus, VEGFR-2 kinase domain crystal structure PDB ID: 2OH4 in complex with a benzimidazole-urea inhibitor was adopted for the current study. After protonation and preparation of the protein, the validity of the used docking protocol was checked by redocking of the bound benzimidazole-urea inhibitor. The redocking validation step successfully regenerated the experimental binding pattern of the co-crystallized ligand with high efficiency. The docking pose reproduced the key interactions accomplished by the co-crystallized ligand in the active site *via* binding with Cys917 in the hinge region, Asp1044 of the DFG motif, and Glu883 in the α-C helix. The reproduced binding mode in addition to the small RMSD (0.71 Å) between the docked pose and the co-crystallized ligand proved the effectiveness of the adopted protocol for the planned docking study (Figure 9).

**Figure 10.**
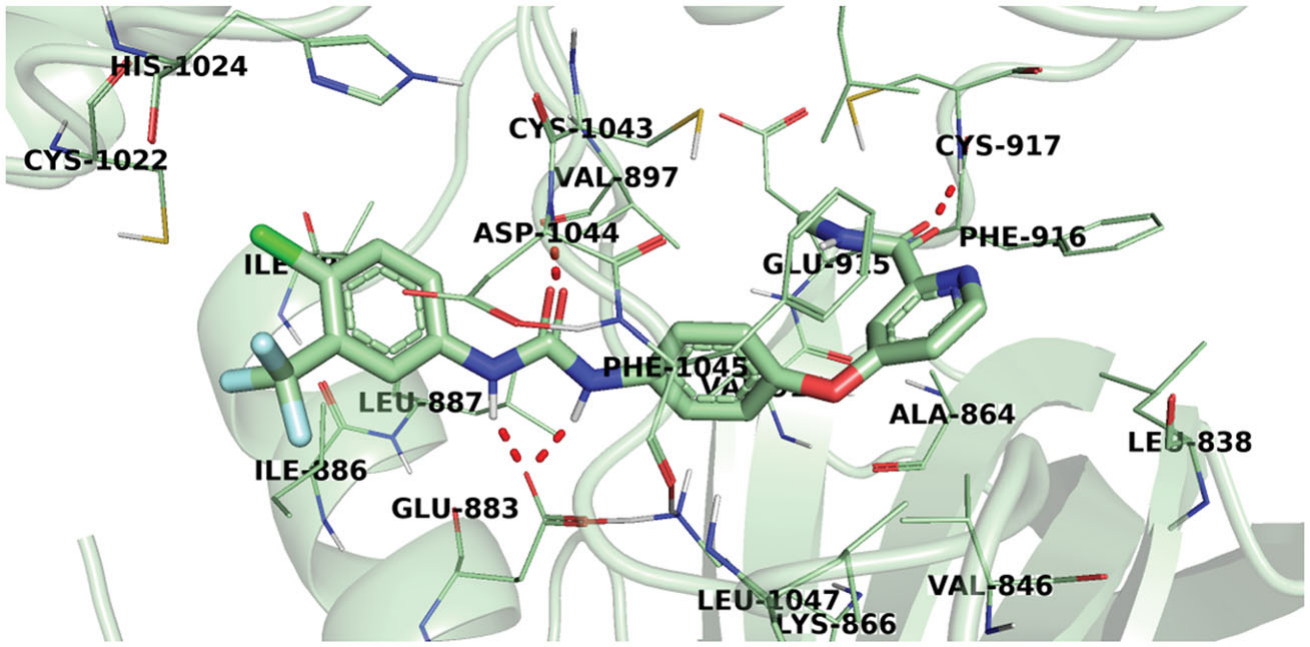
3D representation of sorafenib with VEGFR-2 active site.

Sorafenib, a potent VEGFR-2 inhibitor used in the experimental *in vitro* assays, was used as a reference in the docking study as well. Sorafenib interacted by its urea NH groups with the carboxylate side chain of Glu883 in the α-C helix through H-bond interactions. While the urea carbonyl group was involved in an H-bond interaction with the NH group of Asp1044 of the DFG motif. On the hinge region, sorafenib was found to interact by an H-bond with Cys917. Sorafenib interacted, furthermore, via several hydrophobic interactions with the hydrophobic pocked formed by Lys886, Val897, Ile886, Phe1045, and Cys917 (Figure 10).

**Figure 11.**
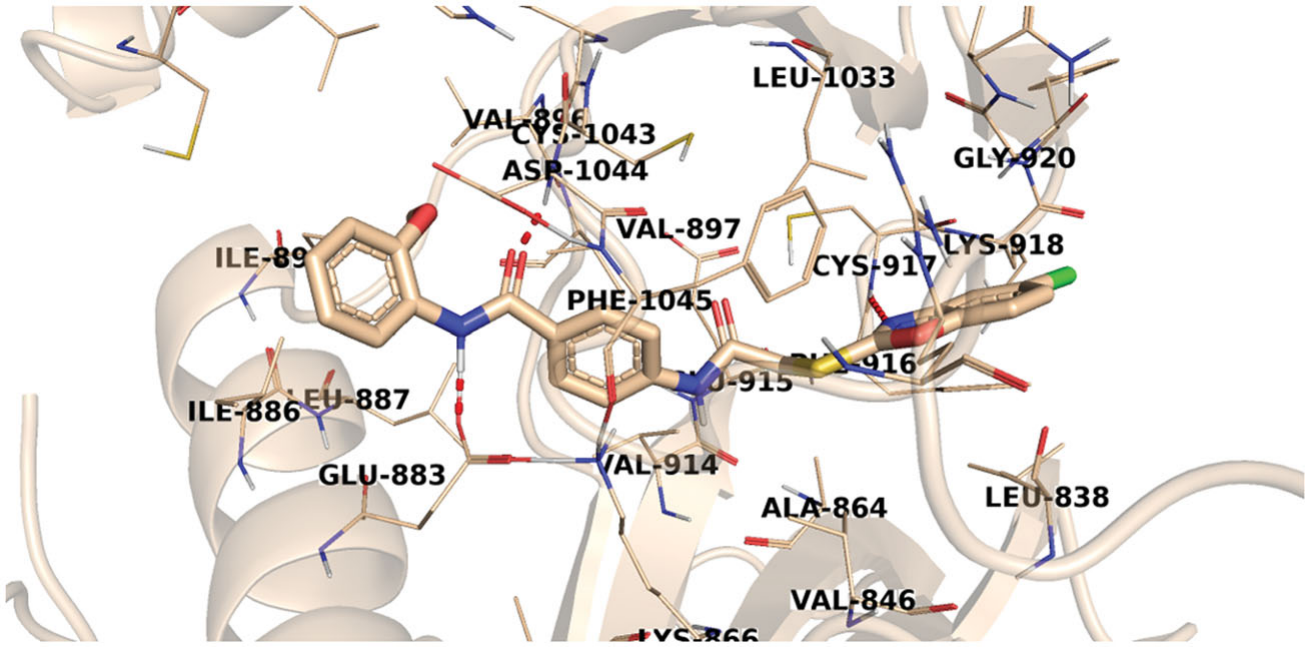
The predicted binding pattern of **14b** with the active site of VEGFR-2.

**Figure 12.**
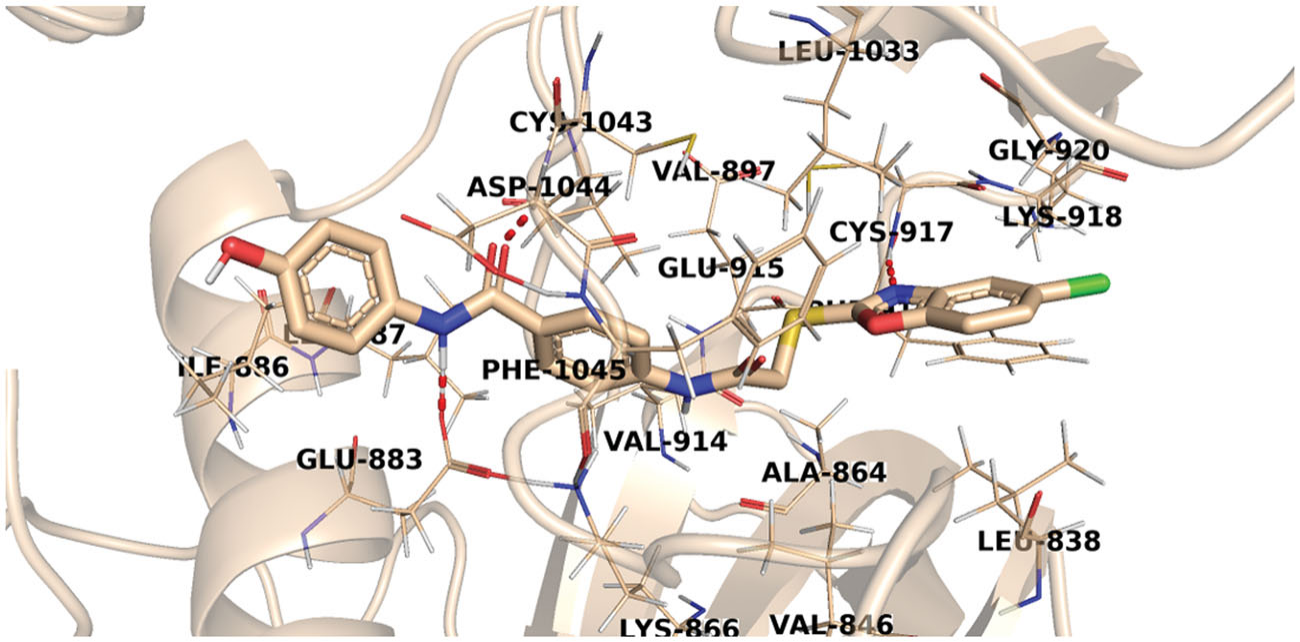
The predicted binding pattern of **14n** with the active site of VEGFR-2.

**Figure 13.**
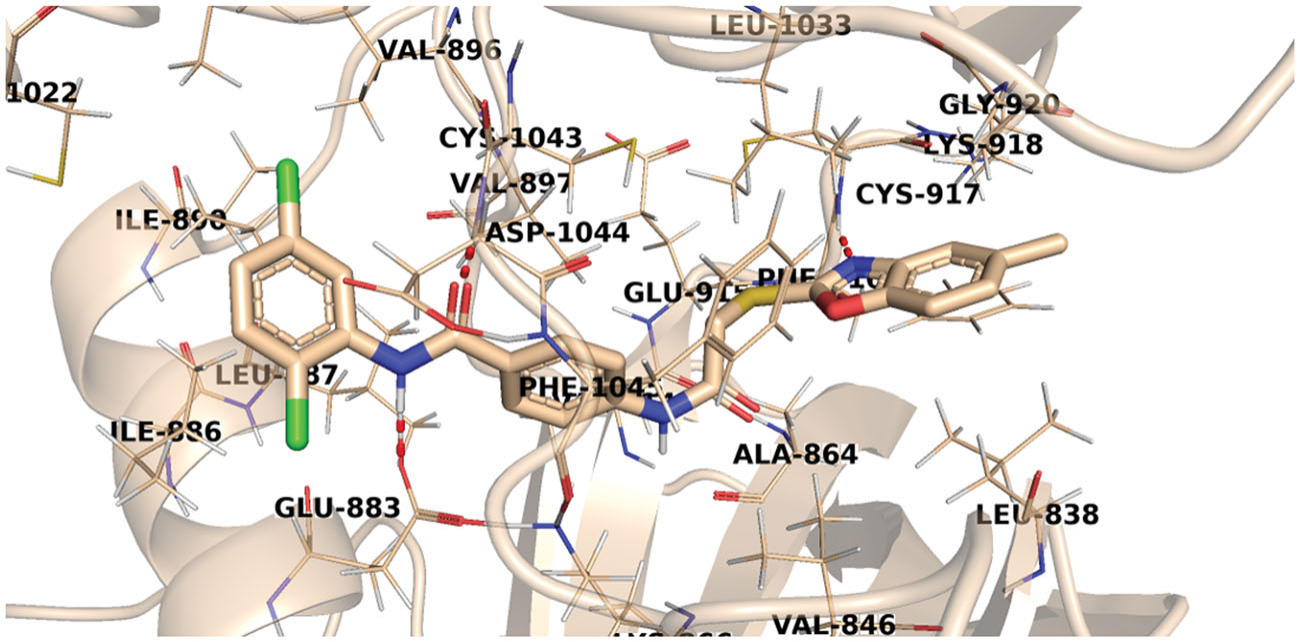
The predicted binding pattern of **14l** with the active site of VEGFR-2.

Investigation of the docking results revealed that the synthesised compounds were able to identify the VEGFR-2 kinase ATP binding site and interact with key amino acids thereof in a manner like that of sorafenib. The studied compounds all occupied the same orientation of sorafenib in the VEGFR-2 kinase active pocket. Thus, as displayed in Figures 11–13, the benzoxazole moieties of the designed compounds **14b**, **14n**, and **14l** were oriented towards the hinge region of the active site forming an H-bond between their nitrogen and Cys917 residue. On the other side, the benzamide scaffolds of the titled compounds were accommodated in the pocket central area, the gate area, interacting *via* one H-bond with the carboxylate side chain of Glu883 and another one with the NH moiety of Asp1044 of the conserved DFG motif in VEGFR-2. However, the orientation of the later moieties allowed the compounds’ hydrophobic substituents to fit in the hydrophobic allosteric pocket in the active site permitting these hydrophobic substituents to interact with hydrophobic side chains of Ile886, Leu887, Ile890, Val896, Val897, Leu1017, and Ile1042 residues lining the back pocket of VEGFR-2.

### 2.4. *In silico* ADME analysis

Results of ADME analysis were illustrated in the Supplementary Data.

### 2.5. Toxicity studies

The toxicity profiles of all the tested compounds were examined. This involves using seven constructed toxicity models (illustrated in Table 5) utilising Discovery studio 4.0 software (Supplementary Data).

## 3. Conclusion

A new series of benzoxazole derivatives was designed hoping to discover novel VEGFR-2 inhibitor agents. Fifteen compounds were synthesised and tested *in vitro* for their anti-proliferative activities against two human cancer cell lines, MCF-7 and HepG2. The tested members exhibited a promising cytotoxic effect with IC_50_ values ranging from 3.22 ± 0.13 to 32.53 ± 1.97 µM. Amongst, six compounds were further investigated for their *in vitro* effect against VEGFR-2 enzyme level. Compounds **14o, 14l**, **14b** and showed the highest effect with a VEGFR-2 protein concentration of 586.3, 636.2, and 705.7 pg/ml, respectively. Also, compound **14b** reduced HUVEC cells proliferation and migratory potential. Moreover, Caspase-3 activation assay was performed for compound **14b** on HepG2 cells. It produced a significant increase in the level of caspase-3 (4.8-fold) compared to the control HepG2 cells. Furthermore, Compound **14b** arrested the cell cycle in the Pre-G1 phase with induction of apoptosis in HepG2 cells. Besides, different *in silico* studies including docking, ADMET, and toxicity were performed. However, the *in silico* studies supported the previous results *via* prediction of the possible binding interactions of the designed compounds with the VEGFR-2 active site.

## 4. Materials and methods

### 4.1. Chemistry

#### 4.1.1. General

All the reagents, chemicals, apparatus were described in Supplementary Data. Compounds **8a–c**, **9a–c**, **11**, **12**, and **13a–e** were obtained according to the reported procedures^14^^,^^33^^,^^34^.

#### 4.1.2. General procedure for preparation of the target compounds 14a-o

A mixture of potassium salts **9a–c** (0.001 mol) and the appropriate 4–(2-chloroacetamido)-*N*-(substituted) phenyl benzamide **13a–e** (0.001 mol), and KI (0.001 mol) in DMF (10 ml) was heated on a water bath for 6 h. After completion of the reaction, the mixture was poured on crushed ice. The precipitates were filtered, dried, and crystallised from methanol to afford the corresponding target compounds **14a–o**.

##### 4.1.2.1. 4-(2-(Benzo[d]oxazol-2-ylthio)acetamido)-N-(2-methoxyphenyl)benzamide 14a

Yellow powder **(**yield, 70%); m. p. = 267–269 °C; HPLC purity 97.50%; IR (KBr, cm^−1^): 3273, 3183 (NH), 3096, 3049 (CH aromatic) 2960, 2845 (CH aliphatic), 1674, 1702 (C=O); ^1^H NMR (400 MHz, DMSO-*d*_6_) *δ* 10.77 (s, 1H), 9.35 (s, 1H), 7.98 (d, *J* = 8.4 Hz, 2H), 7.81 (d, *J* = 7.9 Hz, 1H), 7.76 (d, *J* = 8.3 Hz, 2H), 7.69 − 7.63 (m, 2H), 7.37 − 7.32 (m, 2H), 7.18 (t, *J* = 7.9 Hz, 1H), 7.09 (d, *J* = 8.3 Hz, 1H), 6.98 (t, *J* = 7.6 Hz, 1H), 4.47 (s, 2H), 3.85 (s, 3H); ^13 ^C NMR (101 MHz, DMSO-*d*_6_) δ 165.96, 164.76, 164.33, 151.81 (d, *J* = 2.7 Hz), 142.16, 141.68, 129.72, 129.06, 127.38, 126.02, 125.18, 124.86, 124.59, 120.68, 118.97, 118.74, 111.78, 110.73, 56.17, 37.31; MS (*m/z*) for C_23_H_19_N_3_O_4_S (433.48): 433 (base peak, 100%).

##### 4.1.2.2. 4-(2-((5-Chlorobenzo[d]oxazol-2-yl)thio)acetamido)-N-(2-methoxyphenyl)benzamide 14b

Yellow powder **(**yield, 72%); m. p. = 262–264 °C; HPLC purity 97.55%; IR (KBr, cm^−1^): 3272 (NH), 3099, 3043 (CH aromatic) 2946, 2865 (CH aliphatic), 1675 (C=O); ^1^H NMR (400 MHz, DMSO-*d*_6_) *δ* 10.77 (s, 1H), 9.35 (s, 1H), 7.98 (d, *J* = 8.3 Hz, 2H), 7.82 − 7.79 (m, 1H), 7.76 (d, *J* = 3.0 Hz, 2H), 7.74 − 7.69 (m, 2H), 7.38 (dd, *J* = 8.7, 2.1 Hz, 1H), 7.20 − 7.16 (m, 1H), 7.10 (d, *J* = 8.2 Hz, 1H), 6.98 (t, *J* = 7.6 Hz, 1H), 4.48 (s, 2H), 3.85 (s, 3H); ^13 ^C NMR (101 MHz, DMSO-*d*_6_) δ 166.42, 165.75, 164.74, 151.81, 150.62, 142.96, 142.10, 129.74, 129.47, 129.05, 127.37, 126.02, 124.79, 124.60, 120.67, 118.97, 118.52, 112.02, 111.79, 56.18, 37.42.

##### 4.1.2.3. N-(2-Methoxyphenyl)-4–(2-((5-methylbenzo[d]oxazol-2-yl)thio)acetamido)benzamide 14c

Yellowish white crystal **(**yield, 74%); m. p. = 258–260 °C; HPLC purity 100.00%; IR (KBr, cm^−1^): 3273, 3188 (NH), 3054, 3049 (CH aromatic) 2948, 2849 (CH aliphatic), 1703, 1658 (C=O); ^1^H NMR (400 MHz, DMSO-*d*_6_) *δ* 10.77 (s, 1H), 9.35 (s, 1H), 7.99 − 7.97 (m, 2H), 7.81 (dd, *J* = 7.9, 1.7 Hz, 1H), 7.77 − 7.74 (m, 2H), 7.53 (d, *J* = 8.3 Hz, 1H), 7.45 − 7.43 (m, 1H), 7.20 − 7.14 (m, 2H), 7.13 − 7.10 (m, 1H), 7.00 − 6.96 (m, 1H), 4.45 (s, 2H), 3.85 (s, 3H), 2.40 (s, 3H); ^13 ^C NMR (101 MHz, DMSO-*d*_6_) δ 165.99, 164.75, 164.18, 151.79, 150.09, 142.16, 141.88, 134.58, 129.72, 129.04, 127.39, 126.00, 125.65, 124.55, 120.68, 118.96, 118.67, 111.78, 110.10, 56.18, 37.29, 21.40.

##### 4.1.2.4. 4-(2-(Benzo[d]oxazol-2-ylthio)acetamido)-N-(2,6-dimethoxyphenyl)benzamide 14d

White crystal **(**yield, 65%); m. p. = 250–252 °C; IR (KBr, cm^−1^): 3276, 3193 (NH), 3054 (CH aromatic) 2937 (CH aliphatic), 1653 (C=O); ^1^H NMR (400 MHz, DMSO-*d*_6_) *δ* 10.79 (s, 1H), 9.41 (s, 1H), 8.00 (s, 1H), 7.76 (s, 2H), 7.67 (s, 2H), 7.40 (s, 1H), 7.36 (s, 1H), 7.35 (s, 2H), 7.23 − 7.18 (m, 1H), 6.96 − 6.84 (m, 1H), 4.47 (s, 2H), 4.06 (s, 3H), 3.99 (s, 3H); ^13 ^C NMR (101 MHz, DMSO-*d*_6_) δ 165.98, 164.78, 164.33, 151.90, 151.83, 142.21, 141.68, 129.12, 126.84, 125.20, 124.87, 124.46, 119.00, 118.75, 110.75, 110.11, 56.46, 37.31.

##### 4.1.2.5. 4-(2-((5-Chlorobenzo[d]oxazol-2-yl)thio)acetamido)-N-(2,6-dimethoxyphenyl)-benzamide 14e

Yellowish powder **(**yield, 68%); m. p. = 248–250 °C; HPLC purity 95.44%; IR (KBr, cm^−1^): 3285 (NH), 2936, 2854 (CH aliphatic), 1769, 1664 (C=O); ^1^H NMR (400 MHz, DMSO-*d*_6_) *δ* 10.79 (s, 1H), 9.41 (s, 1H), 8.02 (s, 2H), 7.93 (d, *J* = 8.2 Hz, 2H), 7.78 − 7.77 (m, 2H), 7.71 (s, 1H), 7.40 (s, 2H), 7.38 (d, *J* = 2.1 Hz, 1H), 4.48 (s, 2H), 4.00 (s, 3H), 3.99 (s, 3H); ^13 ^C NMR (101 MHz, DMSO-*d*_6_) δ 166.42, 165.78, 164.77, 151.91, 150.63, 142.96, 142.15, 137.75, 129.70, 129.48, 129.11, 124.81, 124.48, 118.99, 118.53, 112.04, 110.10, 56.45, 37.40.

##### 4.1.2.6. N-(2,6-Dimethoxyphenyl)-4–(2-((5-methylbenzo[d]oxazol-2-yl)thio)acetamido)-benzamide 14f

Greenish white crystal **(**yield, 72%); m. p. = 270–272 °C; HPLC purity 97.33%; IR (KBr, cm^−1^): 3268 (NH), 3069 (CH aromatic) 2930, 2855 (CH aliphatic), 1654 (C=O); ^1^H NMR (400 MHz, DMSO-*d*_6_) *δ* 10.77 (s, 1H), 9.40 (s, 1H), 7.95 (s, 2H), 7.76 (s, 2H), 7.52 (s, 2H), 7.34 (s, 2H), 7.13 (s, 1H), 6.67 (d, *J* = 65.9 Hz, 1H), 4.45 (s, 2H), 3.98 (s, 6H), 2.40 (s, 3H); ^13 ^C NMR (101 MHz, DMSO-*d*_6_) δ 166.01, 164.77, 151.88, 150.09, 142.21, 141.88, 137.73, 134.59, 129.11, 126.84, 125.65, 124.43, 118.99, 118.68, 110.10, 56.45, 37.30, 21.41.

##### 4.1.2.7. 4-(2-(Benzo[d]oxazol-2-ylthio)acetamido)-N-(2,6-dimethylphenyl)benzamide 14g

White crystal **(**yield, 75%); m. p. = 266–268 °C; HPLC purity 96.25%; IR (KBr, cm^−1^): 3262 (NH), 3061 (CH aromatic) 2928, 2856 (CH aliphatic), 1651 (C=O); ^1^H NMR (400 MHz, DMSO-*d*_6_) *δ* 10.77 (s, 1H), 9.70 (s, 1H), 8.07 (dd, *J* = 9.3, 2.4 Hz, 1H), 8.01 (d, *J* = 8.5 Hz, 2H), 7.90 (d, *J* = 8.5 Hz, 1H), 7.75 (d, *J* = 8.4 Hz, 2H), 7.55 (t, *J* = 6.8 Hz, 1H), 7.35 (dd, *J* = 6.3, 2.7 Hz, 2H), 7.13 (s, 2H), 4.46 (s, 2H), 2.19 (s, 6H); ^13 ^C NMR (101 MHz, DMSO-*d*_6_) δ 165.92, 164.84, 164.33, 151.82, 142.01, 141.68, 136.14, 135.89, 129.67, 129.05, 128.18, 127.09, 125.20, 124.87, 118.96, 118.75, 110.75, 37.27, 18.57; MS (*m/z*) for C_24_H_21_N_3_O_3_S (431.51): 431 (base peak, 100%).

##### 4.1.2.8. 4-(2-((5-Chlorobenzo[d]oxazol-2-yl)thio)acetamido)-N-(2,6-dimethylphenyl)benzamide 14h

White powder **(**yield, 72%); m. p. = 262–264 °C; HPLC purity 95.37%; IR (KBr, cm^−1^): 3278, 3225 (NH), 3022 (CH aromatic) 2979, 2918, 2859 (CH aliphatic), 1656 (C=O); ^1^H NMR (400 MHz, DMSO-*d*_6_) *δ* 10.78 (s, 1H), 9.71 (s, 1H), 8.03 (s, 2H), 7.76 (s, 3H), 7.38 (s, 2H), 7.13 (s, 3H), 4.49 (s, 2H), 2.20 (s, 6H); ^13 ^C NMR (101 MHz, DMSO-*d*_6_) δ 166.43, 165.73, 164.86, 150.62, 142.97, 141.98, 136.15, 135.89, 129.71, 129.48, 129.07, 128.18, 127.09, 124.77, 118.99, 118.51, 111.99, 37.43, 18.58.

##### 4.1.2.9. N-(2,6-Dimethylphenyl)-4–(2-((5-methylbenzo[d]oxazol-2-yl)thio)acetamido)benzamide 14i

Yellowish green crystal **(**yield, 68%); m. p. = 263–265 °C; HPLC purity 96.77%; IR (KBr, cm^−1^): 3256, 3106 (NH), 2923, 2861 (CH aliphatic), 1761, 1641 (C=O); ^1^H NMR (400 MHz, DMSO-*d*_6_) *δ* 10.76 (s, 1H), 9.70 (s, 1H), 8.01 (d, *J* = 8.4 Hz, 2H), 7.75 (d, *J* = 8.4 Hz, 2H), 7.53 (d, *J* = 8.3 Hz, 1H), 7.44 (s, 1H), 7.17 − 7.14 (m, 2H), 7.13 (s, 2H), 4.44 (s, 2H), 2.41 (s, 3H), 2.19 (s, 6H); ^13 ^C NMR (101 MHz, DMSO-*d*_6_) δ 165.95, 164.85, 164.19, 150.08, 142.01, 141.88, 136.14, 135.88, 134.59, 129.66, 129.05, 128.18, 127.09, 125.66, 118.95, 118.67, 110.11, 37.25, 21.41, 18.57.

##### 4.1.2.10. 4-(2-(Benzo[d]oxazol-2-ylthio)acetamido)-N-(2,5-dichlorophenyl)benzamide 14j

White powder **(**yield, 74%); m. p. = 265–267 °C; HPLC purity 97.14%; IR (KBr, cm^−1^): 3269, 3186 (NH), 3058 (CH aromatic) 2992, 2936 (CH aliphatic), 1659 (C=O); ^1^H NMR (400 MHz, DMSO-*d*_6_) *δ* 10.80 (s, 1H), 10.06 (s, 1H), 8.00 (d, *J* = 8.4 Hz, 2H), 7.78 (s, 2H), 7.76 (s, 1H), 7.68 − 7.64 (m, 2H), 7.61 (d, *J* = 8.6 Hz, 1H), 7.40 − 7.38 (m, 1H), 7.36 − 7.32 (m, 2H), 4.47 (s, 2H); ^13 ^C NMR (101 MHz, DMSO-*d*_6_) δ 166.03, 165.24, 164.32, 151.82, 142.55, 141.67, 136.95, 131.90, 131.36, 129.43, 128.79, 128.23, 127.95, 127.42, 125.19, 124.87, 118.96, 118.75, 110.75, 37.29; MS (*m/z*) for C_22_H_15_Cl_2_N_3_O_3_S (472.34): 471 (base peak, 100%), 473 (M^+^ + 1, 70%).

##### 4.1.2.11. 4-(2-((5-Chlorobenzo[d]oxazol-2-yl)thio)acetamido)-N-(2,5-dichlorophenyl)-benzamide 14k

Off white powder **(**yield, 70%); m. p. = 259–261 °C; HPLC purity 99.13%; IR (KBr, cm^−1^): 3271, 3187 (NH), 3059 (CH aromatic) 2977, 2939 (CH aliphatic), 1664 (C=O); ^1^H NMR (400 MHz, DMSO-*d*_6_) *δ* 10.81 (s, 1H), 10.05 (s, 1H), 8.00 (d, *J* = 8.5 Hz, 2H), 7.77 (s, 2H), 7.75 (s, 2H), 7.71 (d, *J* = 8.6 Hz, 1H), 7.60 (d, *J* = 8.6 Hz, 1H), 7.37 (dd, *J* = 8.7, 4.0 Hz, 2H), 4.47 (s, 2H); ^13 ^C NMR (101 MHz, DMSO-*d*_6_) δ 166.40, 165.83, 165.23, 150.62, 142.96, 142.51, 136.93, 131.90, 131.35, 129.48, 129.43, 128.81, 128.22, 127.93, 127.41, 124.79, 118.97, 118.52, 112.02, 37.41; MS (*m/z*) for C_22_H_14_Cl_3_N_3_O_3_S (506.78): 506 (M^+,^ 36%), 345 (base peak, 100%).

##### 4.1.2.12. N-(2,5-Dichlorophenyl)-4–(2-((5-methylbenzo[d]oxazol-2-yl)thio)acetamido)-benzamide 14l

White powder **(**yield, 76%); m. p. = 255–257 °C; HPLC purity 100.00%; IR (KBr, cm^−1^): 3274, 3182 (NH), 3052 (CH aromatic), 2953 (CH aliphatic), 1699, 1660 (C=O); ^1^H NMR (400 MHz, DMSO-*d*_6_) *δ* 10.90 (s, 1H), 9.36 (s, 1H), 7.99 (d, *J* = 8.2 Hz, 2H), 7.80 (s, 1H), 7.64 (d, *J* = 7.9 Hz, 2H), 7.38 − 7.31 (m, 2H), 7.17 (d, *J* = 7.8 Hz, 1H), 7.09 (d, *J* = 8.2 Hz, 1H), 6.98 (t, *J* = 7.7 Hz, 1H), 4.48 (s, 2H), 3.85 (s, 3H); ^13 ^C NMR (101 MHz, DMSO-*d*_6_) δ 165.98, 164.78, 164.33, 151.82, 151.80, 142.22, 141.69, 129.69, 129.03, 127.38, 126.01, 125.17, 124.84, 124.58, 120.67, 118.98, 118.74, 111.77, 110.72, 56.17, 37.30.

##### 4.1.2.13. 4-(2-(Benzo[d]oxazol-2-ylthio)acetamido)-N-(4-hydroxyphenyl)benzamide 14m

Brownish powder **(**yield, 60%); m. p. = 280–282 °C; ^1^H NMR (400 MHz, DMSO-*d*_6_) *δ* 10.74 (s, 1H), 9.95 (s, 1H), 9.27 (s, 1H), 7.95 (d, *J* = 8.1 Hz, 2H), 7.73 (d, *J* = 8.3 Hz, 2H), 7.66 (d, *J* = 8.9 Hz, 2H), 7.54 (d, *J* = 8.4 Hz, 2H), 7.37 − 7.31 (m, 2H), 6.75 (d, *J* = 8.3 Hz, 2H), 4.46 (s, 2H); ^13 ^C NMR (101 MHz, DMSO-*d*_6_) δ 165.91, 164.74, 164.33, 154.11, 151.82, 141.85, 141.67, 131.21, 130.38, 129.05, 125.18, 124.85, 122.76, 118.83, 118.74, 115.43, 110.73, 37.29.

##### 4.1.2.14. 4-(2-((5-Chlorobenzo[d]oxazol-2-yl)thio)acetamido)-N-(4-hydroxyphenyl)benzamide 14n

Brownish white powder **(**yield, 62%); m. p. = 272–274 °C; HPLC purity 94.00%; IR (KBr, cm^−1^): 3267 (NH), 3067 (CH aromatic), 2930 (CH aliphatic), 1649 (C=O); ^1^H NMR (400 MHz, DMSO-*d*_6_) *δ* 10.75 (s, 1H), 9.96 (s, 1H), 9.28 (s, 1H), 7.97 (d, *J* = 8.3 Hz, 2H), 7.75 (s, 1H), 7.73 (s, 2H), 7.67 (d, *J* = 8.6 Hz, 1H), 7.55 (d, *J* = 8.4 Hz, 2H), 7.36 (d, *J* = 8.7 Hz, 1H), 6.77 (d, *J* = 8.4 Hz, 2H), 4.48 (s, 2H); ^13 ^C NMR (101 MHz, DMSO-*d*_6_) δ 166.41, 165.71, 164.75, 154.12, 150.60, 142.95, 141.81, 131.22, 130.41, 129.47, 129.05, 124.73, 122.79, 118.86, 118.49, 115.45, 111.93, 37.46.

##### 4.1.2.15. N-(4-Hydroxyphenyl)-4–(2-((5-methylbenzo[d]oxazol-2-yl)thio)acetamido)benzamide 14o

Yellowish crystal **(**yield, 65%); m. p. = 269–271 °C; HPLC purity 95.16%; IR (KBr, cm^−1^): 3264 (NH), 3043 (CH aromatic), 2926 (CH aliphatic), 1634 (C=O); ^1^H NMR (400 MHz, DMSO-*d*_6_) *δ* 10.75 (s, 1H), 9.96 (s, 1H), 9.28 (s, 1H), 7.97 (d, *J* = 8.3 Hz, 2H), 7.75 (d, *J* = 8.3 Hz, 2H), 7.55 (d, *J* = 8.4 Hz, 2H), 7.51 (d, *J* = 8.3 Hz, 1H), 7.42 (s, 1H), 7.12 (d, *J* = 8.3 Hz, 1H), 6.77 (d, *J* = 8.3 Hz, 2H), 4.44 (s, 2H), 2.39 (s, 3H); ^13 ^C NMR (101 MHz, DMSO-*d*_6_) δ 166.41, 165.71, 164.75, 154.12, 150.60, 142.95, 141.81, 131.22, 130.41, 129.47, 129.05, 124.73, 122.79, 118.86, 118.49, 115.45, 111.93, 37.46.

### 4.2. Biological evaluation

#### 4.2.1. *In vitro* anti-proliferative activity

The anti-proliferative activity of all tested compounds was performed on MCF-7 and HepG2 cells by using 3-(4,5-dimethylthiazol-2-yl)-2,5-diphenyltetrazolium bromide (MTT) assay^35^^,^^41–44^. The MTT assay is based on the capability of living cells to reduce the yellow product MTT to a blue product, formazan, by a reduction reaction occurring in the mitochondria. Briefly, in MTT assay, 5000 cells/well were plated in a 96-well plate and allowed to grow 24 h, then treated with Roswell Park Memorial Institute (RPMI) 1640 media that contain increased concentrations (0, 0.1, 1, 10, 100, and 1000 µM) of tested compounds. Each experiment was carried out in triplicate. Then media were removed and 100 µL of MTT was added to each well and incubated for 4 h. The formed formazan crystals were solubilised by adding 100 µL of dimethyl sulfoxide (DMSO) solution and absorbance was measured at 570 nm using ELISA microplate reader (Epoc-2 C micro-plate reader, Bio Tek, VT, USA). The IC_50_ values [the concentration required for 50% inhibition of cell viability] were calculated and the results are expressed as the relative percentage of the control cells (100% of cell viability).

#### 4.2.2. *In vitro* VEGFR-2 protein concentration assay

The *in vitro* assessment of VEGFR-2 protein concentration after exposure of HepG-2 cells to the most cytotoxic candidates was carried out using Enzyme-Linked Immunosorbent Assay (ELISA) kit (Cat. NO. EK0544) (AVIVA System Biology, USA) according to manufacturer instructions^45^.

#### 4.2.3. Wound healing assay

Allow 10 min for the 24-well plate with CytoSelect™ Wound Healing Inserts to warm up at room temperature before applying 500 ml of HUVECs cell suspension (1.0 × 10^6^) in media containing 10% foetal bovine serum (FBS) to each well. In a cell culture incubator, incubate the cells until they form a monolayer. Remove the implant from the well gently to begin the wound healing assay. Aspirate the media from the wells slowly and discard it.

To eliminate dead cells and debris, wash wells with the medium. Finally, fill wells with medium to keep cells hydrated, and examine them under a light microscope. The wells were subsequently filled with media containing the indicated concentrations of compound **14b** or sorafenib for 72 h. A light microscope is used to monitor the wound closure. Calculate the percentage of cells that have closed into the wound field^46^.

#### 4.2.4. Analysis of the cell cycle distribution phases

The propidium iodide staining followed by flow cytometric analysis was conducted according to the cell cycle kit (PN C03551) and previously published works^43^^,^^47^ to investigate the effect of compound **14b** on the cell cycle phases. In Brief, HepG2 cells were allowed to grow in 25 cm^3^ flask until reach 70–80% confluence, then treated with compound **14b** for 48 h. Then the cells were harvested and fixed. The cells were centrifuged at 2000 rpm for 5 min then, the supernatant was aspirated. The pellet of fixed cells was resuspended in a 0.5 ml cell cycle kit, vortexed, and incubated at 25 °C for 15 min. Finally, DNA was stained with 50 µg/ml propidium iodide for 30 min. Flow cytometric analysis of cell cycle performed on a COULTER^®^ EPICS^®^ XL™ Flow Cytometer (USA).

#### 4.2.5. Annexin V-FITC apoptosis assay

For the detection of apoptosis in treated cells, Annexin V—FITC—apoptosis detection kit (PN IM3546) was used, followed by flow cytometric analysis according to manufacturer protocol. In this assay, HepG-2 cells were allowed to grow in a 25 cm^3^ flask until 70–80% confluence. Then HepG-2 cells were treated with compound **14b** for 48 h followed by a wash in PBS and suspended in 1× binding buffer. To 100 µL of the cell suspensions, 1 µL of annexin V-FITC solution and 5 µL of dissolved PI were added and incubated for 15 min in the dark. Then 400 µL of ice-cold 1× binding buffer was added and mixed gently. The flow cytometric analysis for the percentage of apoptotic cells was performed on a COULTER^®^ EPICS^®^ XL™ Flow Cytometer (USA)^42^^,^^48^.

#### 4.2.6. Caspase-3 determination

The effect of compound **14b** on Caspase-3 level was assessed using ELISA kit (Catalog # KHO1091) according to manufacturer instructions.

### 4.3. Molecular docking studies

Molecular docking studies of synthesised compounds were carried out against VEGFR-2 (PDB ID: 2OH4, resolution: 2.05 Å) using MOE.14 software^27^^,^^49–54^ as shown in Supplementary Data.

## Supplementary Material

Supplemental Material
