# Supplementary material for: New benzoxazole derivatives as potential VEGFR-2 inhibitors and apoptosis inducers: design, synthesis, anti-proliferative evaluation, flowcytometric analysis, and in silico studies
Source: J Enzyme Inhib Med Chem. 2021 Dec 27;37(1):397–410. doi: 10.1080/14756366.2021.2015343 (PMC8725875; doi:10.1080/14756366.2021.2015343)

IR of comp. 14a

Peak Find - PBA4.jws

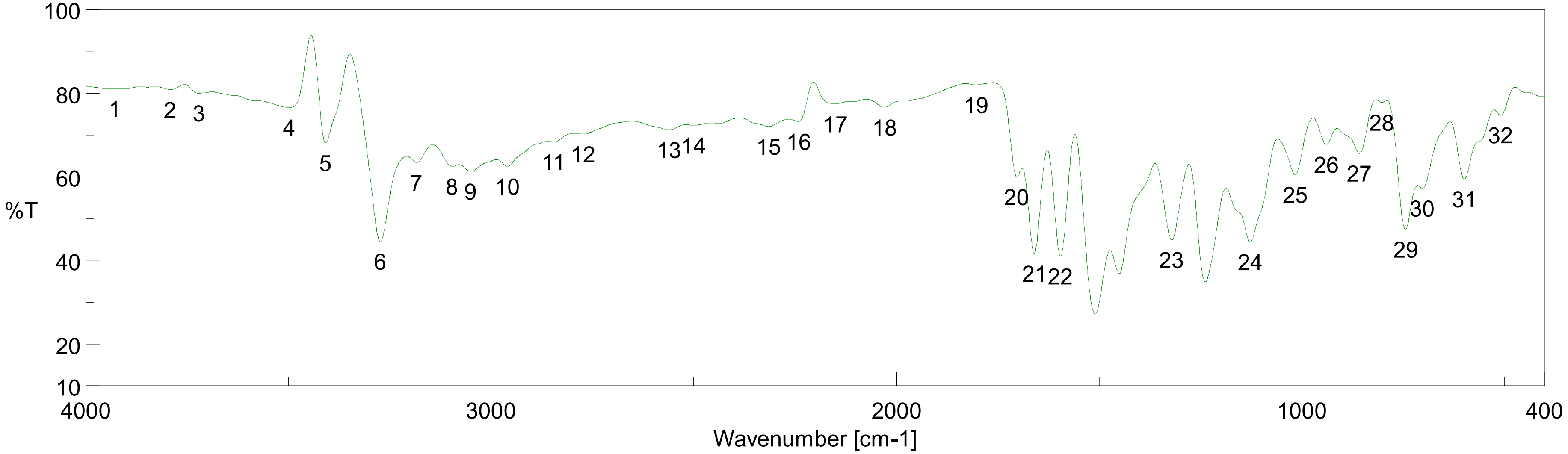

[Result of Peak Picking]

No.	Position	Intensity	No.	Position	Intensity	No.	Position	Intensity	No.	Position	Intensity
1	3929.25	81.054	2	3792.33	80.8358	3	3720.98	79.9616	4	3498.24	76.5509
5	3408.57	68.0954	6	3273.57	44.4843	7	3183.9	63.4105	8	3096.15	62.4796
9	3049.87	61.3067	10	2960.2	62.4984	11	2845.45	68.3026	12	2773.14	70.2443
13	2561.97	71.2275	14	2500.26	72.3374	15	2316.09	72.0166	16	2241.84	73.1928
17	2151.2	77.3985	18	2029.71	76.6288	19	1804.08	81.9624	20	1702.84	59.9278
21	1659.45	41.6617	22	1594.84	41.007	23	1321	44.9473	24	1127.19	44.412
25	1016.3	60.5109	26	939.163	67.7349	27	857.204	65.5736	28	802.242	77.7547
29	743.424	47.4147	30	701.962	57.196	31	598.789	59.4839	32	509.115	74.7025

Mass spec. of comp. 14a

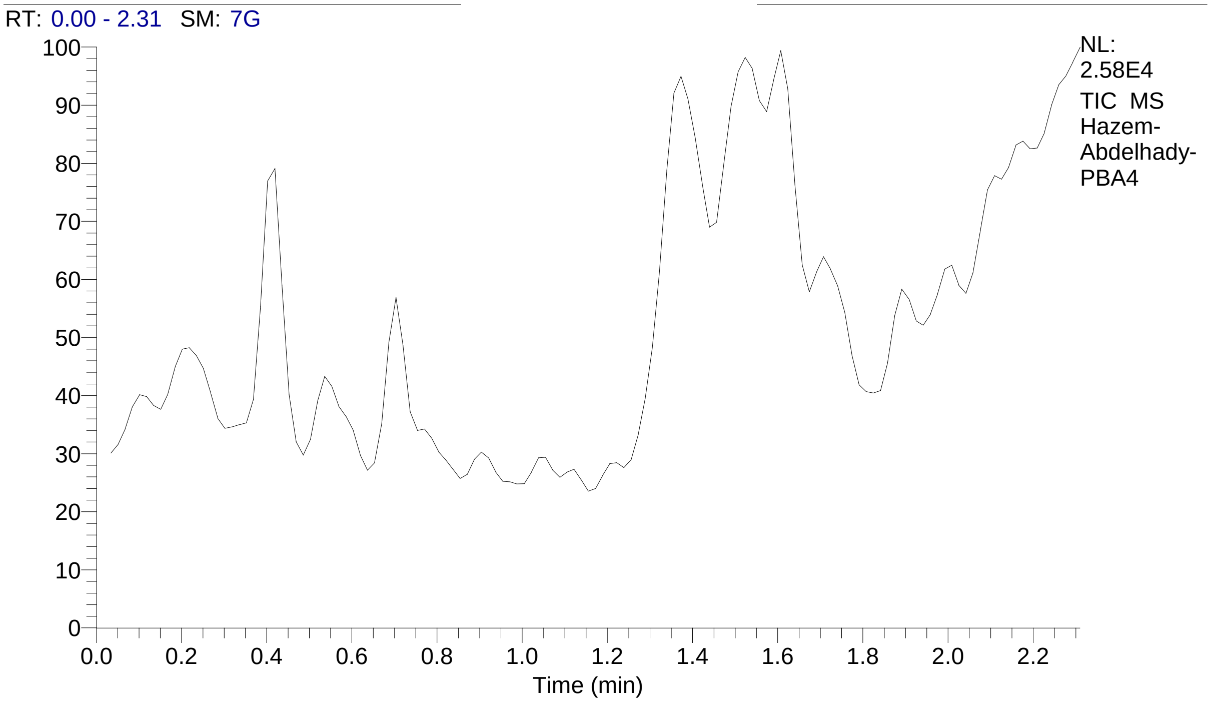

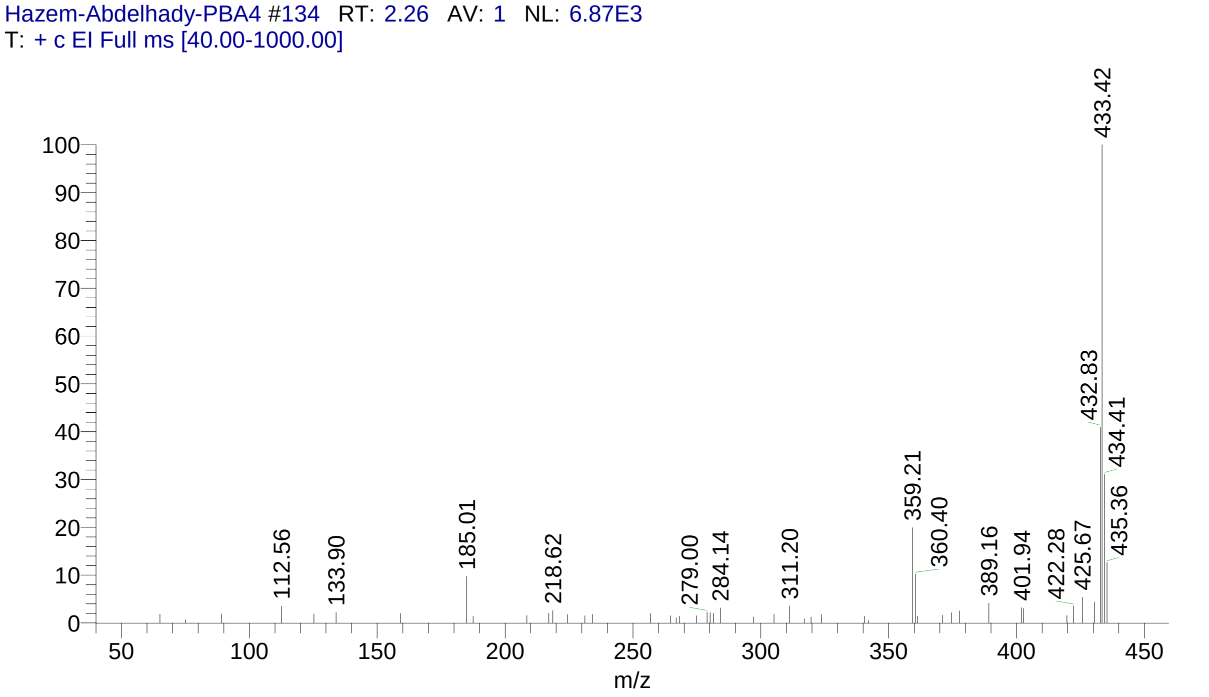

m/z	Intensity	Relative
65.05	128.5	1.87

75.00	47.3	0.69	
89.23	131.9	1.92	
112.56	241.3	3.51	
125.23	131.7	1.92	
133.90	151.2	2.20	
159.01	136.7	1.99	
185.01	668.1	9.72	
187.48	97.2	1.41	
208.48	107.3	1.56	
217.08	140.0	2.04	
218.62	176.9	2.57	
224.47	121.6	1.77	
231.19	101.6	1.48	
234.27	124.7	1.81	
256.97	135.9	1.98	
264.76	103.9	1.51	
266.94	73.3	1.07	
268.20	95.5	1.39	
274.94	104.7	1.52	
279.00	154.4	2.25	
280.21	146.9	2.14	
281.56	141.6	2.06	
284.14	216.7	3.15	
297.18	88.0	1.28	
305.16	122.9	1.79	
311.20	246.9	3.59	
317.00	59.6	0.87	
319.64	85.9	1.25	
323.73	121.5	1.77	
340.60	95.9	1.39	
342.02	36.4	0.53	
359.21	1368.3		19.91
360.40	703.8	10.24	
361.31	100.8	1.47	
370.99	111.1	1.62	
374.54	148.7	2.16	
377.64	174.9	2.55	
389.16	282.6	4.11	
401.94	218.0	3.17	
402.60	209.5	3.05	
419.68	105.1	1.53	
422.28	246.4	3.59	
425.67	369.2	5.37	
430.55	303.2	4.41	
432.83	2816.6		40.98
433.42	6872.6		100.00
434.41	2137.3		31.10
435.36	866.7	12.61	

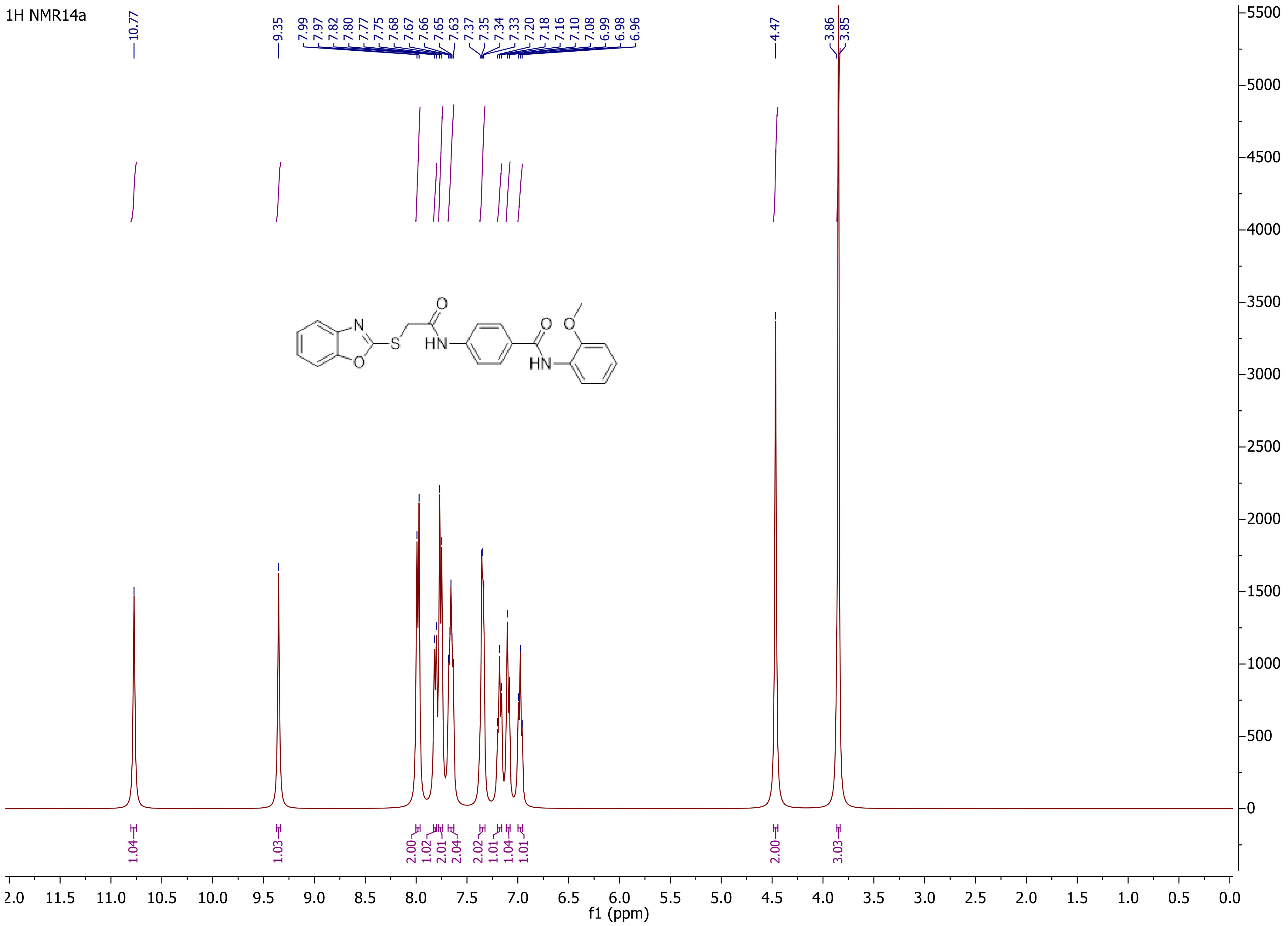
1H NMR14a
10.77
9.35
7.99 7.97 7.82 7.80 7.77 7.75 7.68 7.67 7.66 7.65 7.63 7.37 7.35 7.34 7.33 7.20 7.18 7.16 7.10 7.08 6.99 6.98 6.96
4.47
3.86 3.85
1.04
1.03
2.00 1.02 2.01 2.04 2.02 1.01 1.04 1.01
2.00
3.03
f1 (ppm)

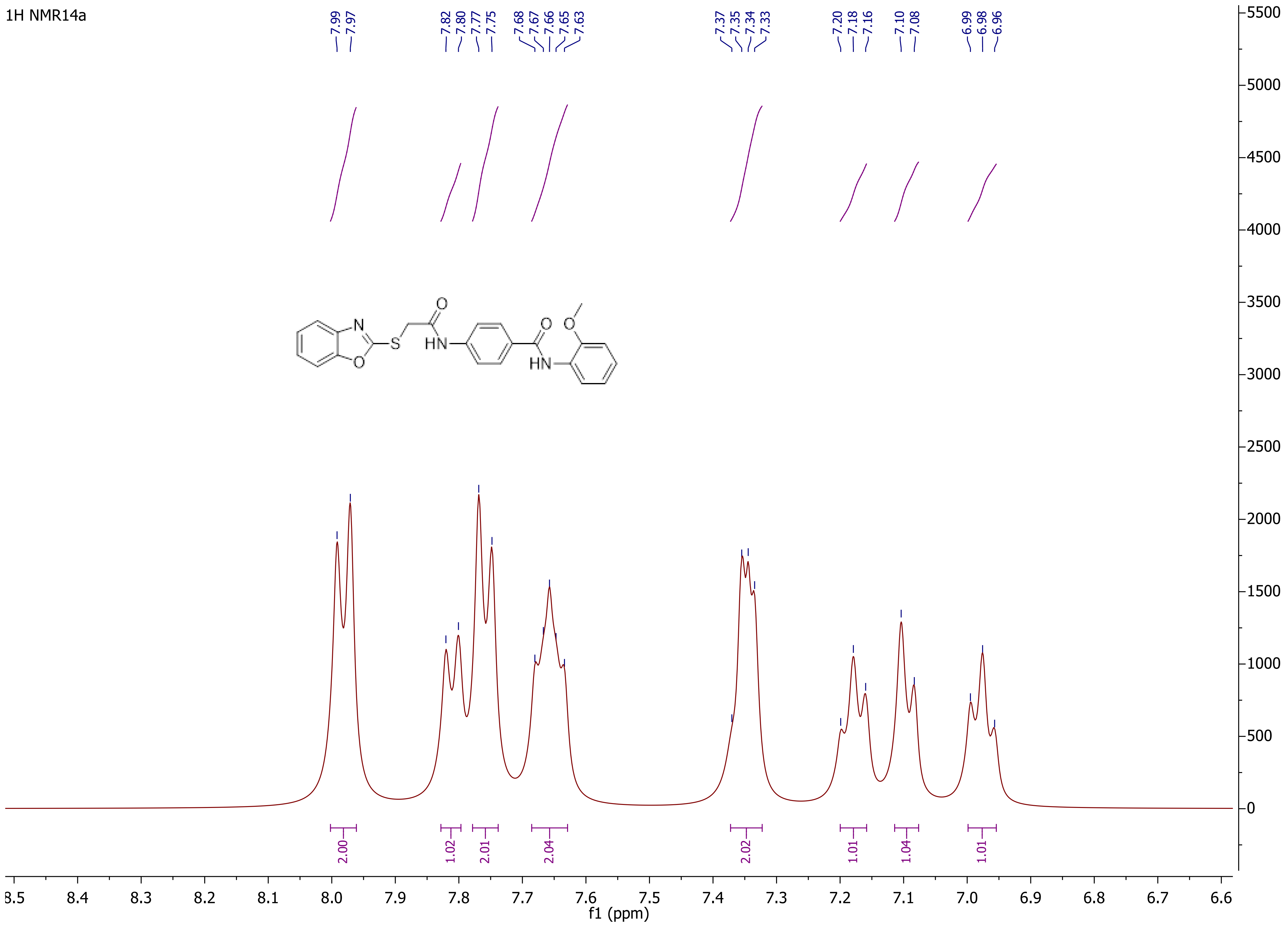
1H NMR14a
7.99
7.97
7.82
7.80
7.77
7.75
7.68
7.67
7.66
7.65
7.63
7.37
7.35
7.34
7.33
7.20
7.18
7.16
7.10
7.08
6.99
6.98
6.96
2.00
1.02
2.01
2.04
2.02
1.01
1.04
1.01
f1 (ppm)

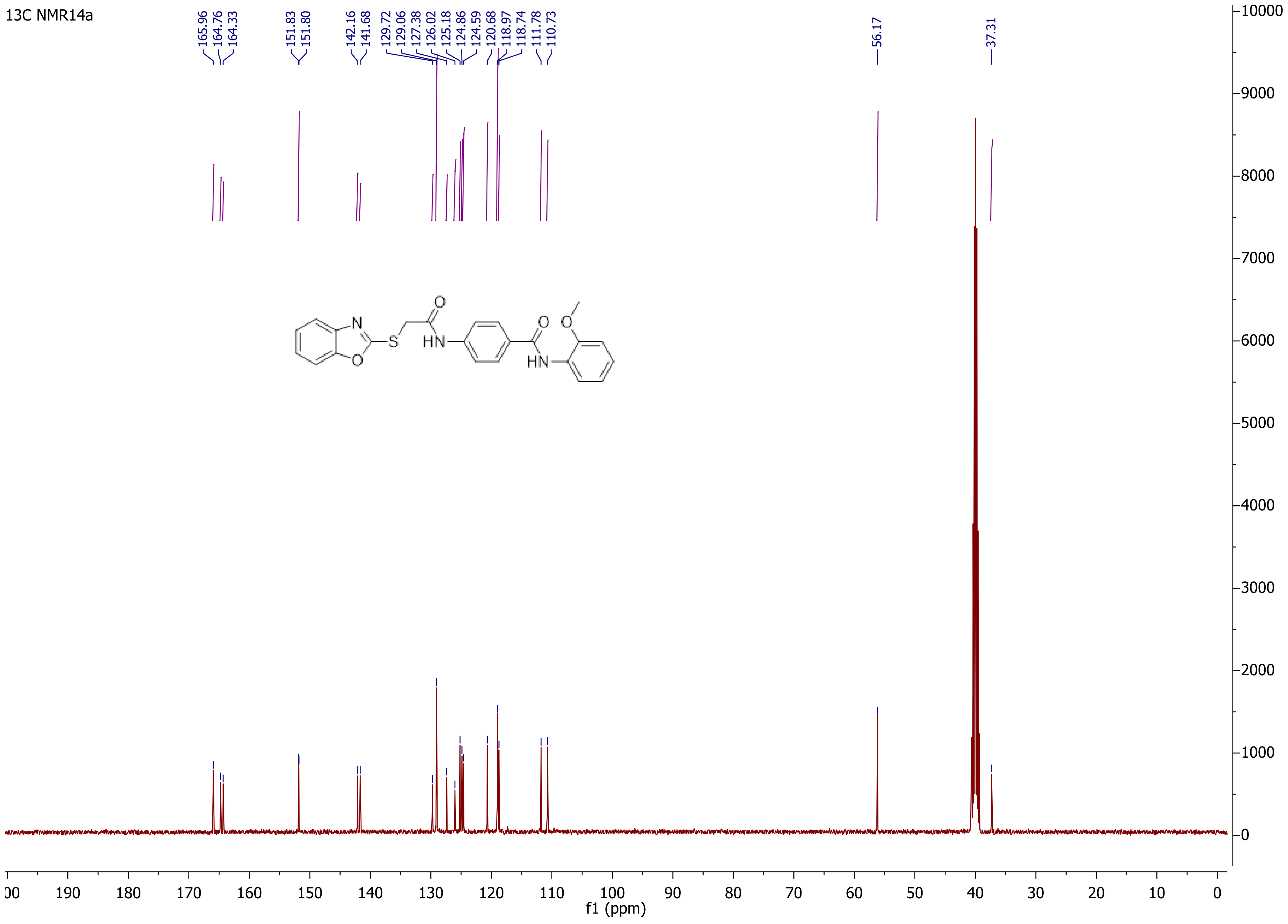

13C NMR14a
165.96
164.76
164.33
151.83
151.80
142.16
141.68
129.72
129.06
127.38
126.02
125.18
124.86
124.59
120.68
118.97
118.74
111.78
110.73
56.17
37.31
HN
HN
N
O
S
O
O
O
10000
9000
8000
7000
6000
5000
4000
3000
2000
1000
0
00
190
180
170
160
150
140
130
120
110
100
90
80
70
60
50
40
30
20
10
0
f1 (ppm)

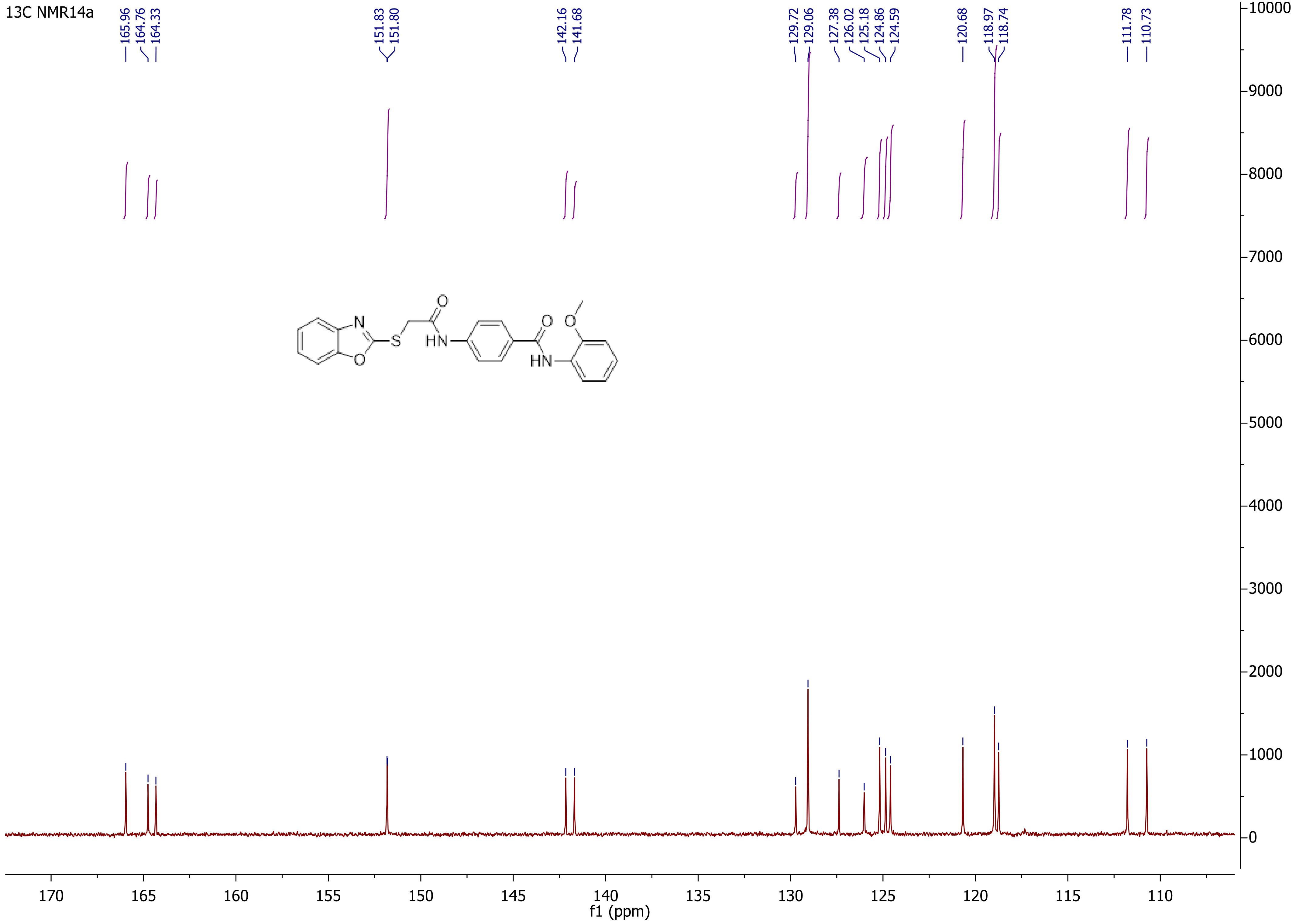
13C NMR14a
165.96
164.76
164.33
151.83
151.80
142.16
141.68
129.72
129.06
127.38
126.02
125.18
124.86
124.59
120.68
118.97
118.74
111.78
110.73
N
S
O
O
HN
O
O
HN
f1 (ppm)
170
165
160
155
150
145
140
135
130
125
120
115
110
10000
9000
8000
7000
6000
5000
4000
3000
2000
1000
0

IR of comp. 14b

Peak Find - 16.jws

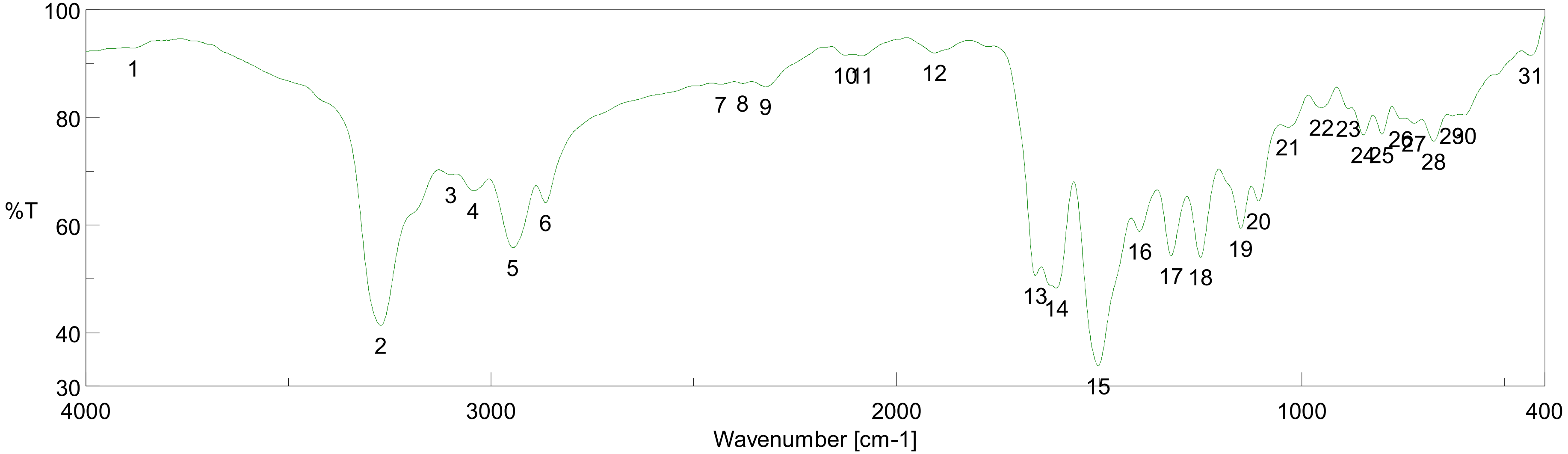

[Result of Peak Picking]

No.	Position	Intensity	No.	Position	Intensity	No.	Position	Intensity	No.	Position	Intensity
1	3882.97	92.8102	2	3272.61	41.3259	3	3099.05	69.3114	4	3043.12	66.3426
5	2946.7	55.7468	6	2865.7	64.1328	7	2433.73	86.1079	8	2378.76	86.2771
9	2321.87	85.6609	10	2126.13	91.5033	11	2084.67	91.383	12	1906.29	91.9338
13	1657.52	50.5694	14	1605.45	48.2052	15	1502.28	33.7637	16	1400.07	58.7769
17	1321.96	54.2399	18	1249.65	53.9906	19	1150.33	59.311	20	1105.98	64.4072
21	1032.69	78.0857	22	950.734	81.7663	23	885.166	81.6066	24	847.561	76.7036
25	802.242	76.8122	26	754.995	79.6765	27	722.211	78.8431	28	673.999	75.4962
29	628.68	80.2641	30	597.825	80.3875	31	434.869	91.4695			

1HNMR 14b

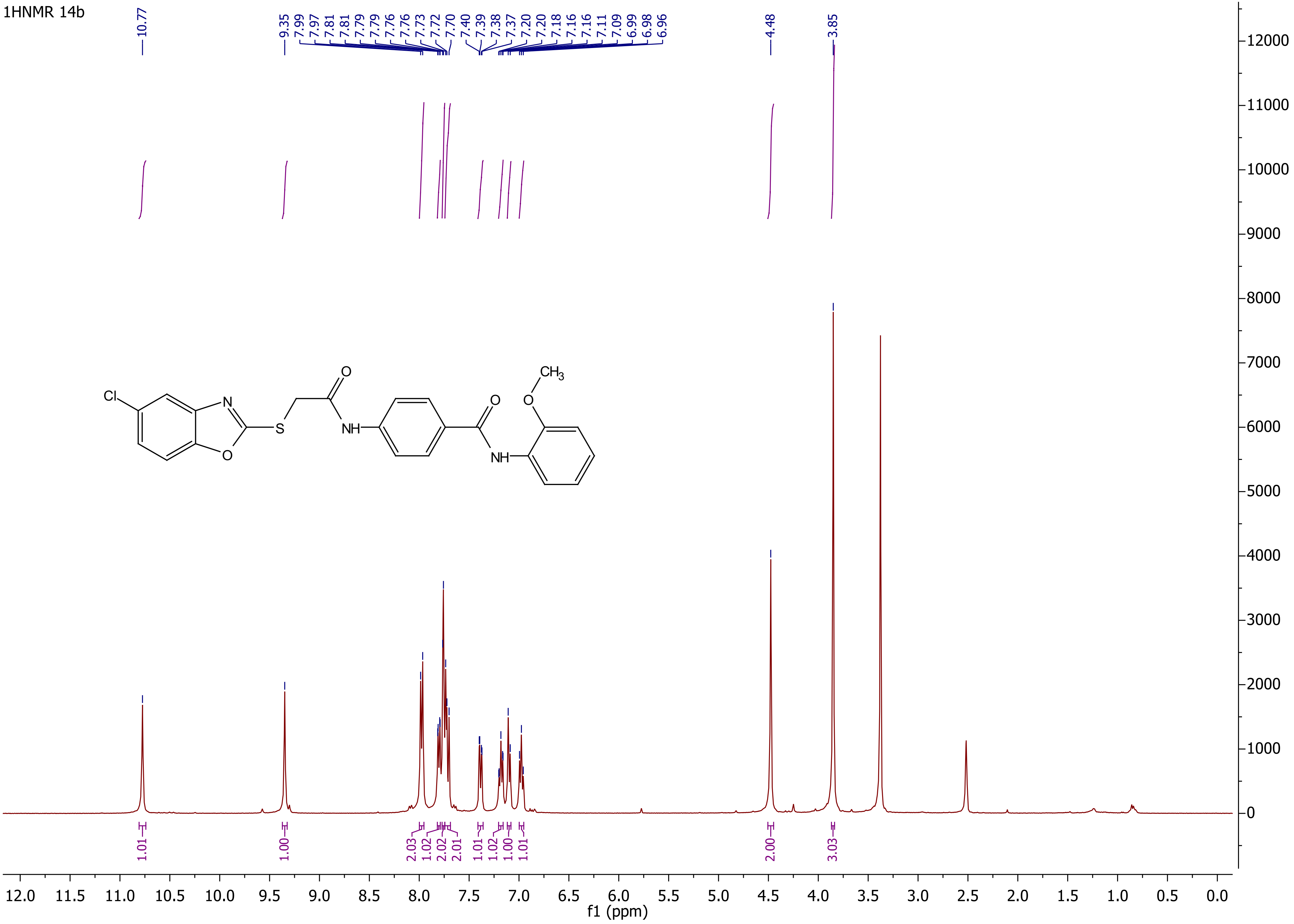

10.77
9.35
7.99
7.97
7.81
7.81
7.79
7.79
7.76
7.76
7.73
7.72
7.70
7.40
7.39
7.38
7.37
7.20
7.20
7.18
7.16
7.16
7.11
7.09
6.99
6.98
6.96
4.48
3.85
Cl
N
O
S
NH
O
O
CH3
NH
1.01
1.00
2.03
1.02
2.02
2.01
1.01
1.02
1.00
1.01
2.00
3.03
f1 (ppm)

1HNMR 14b

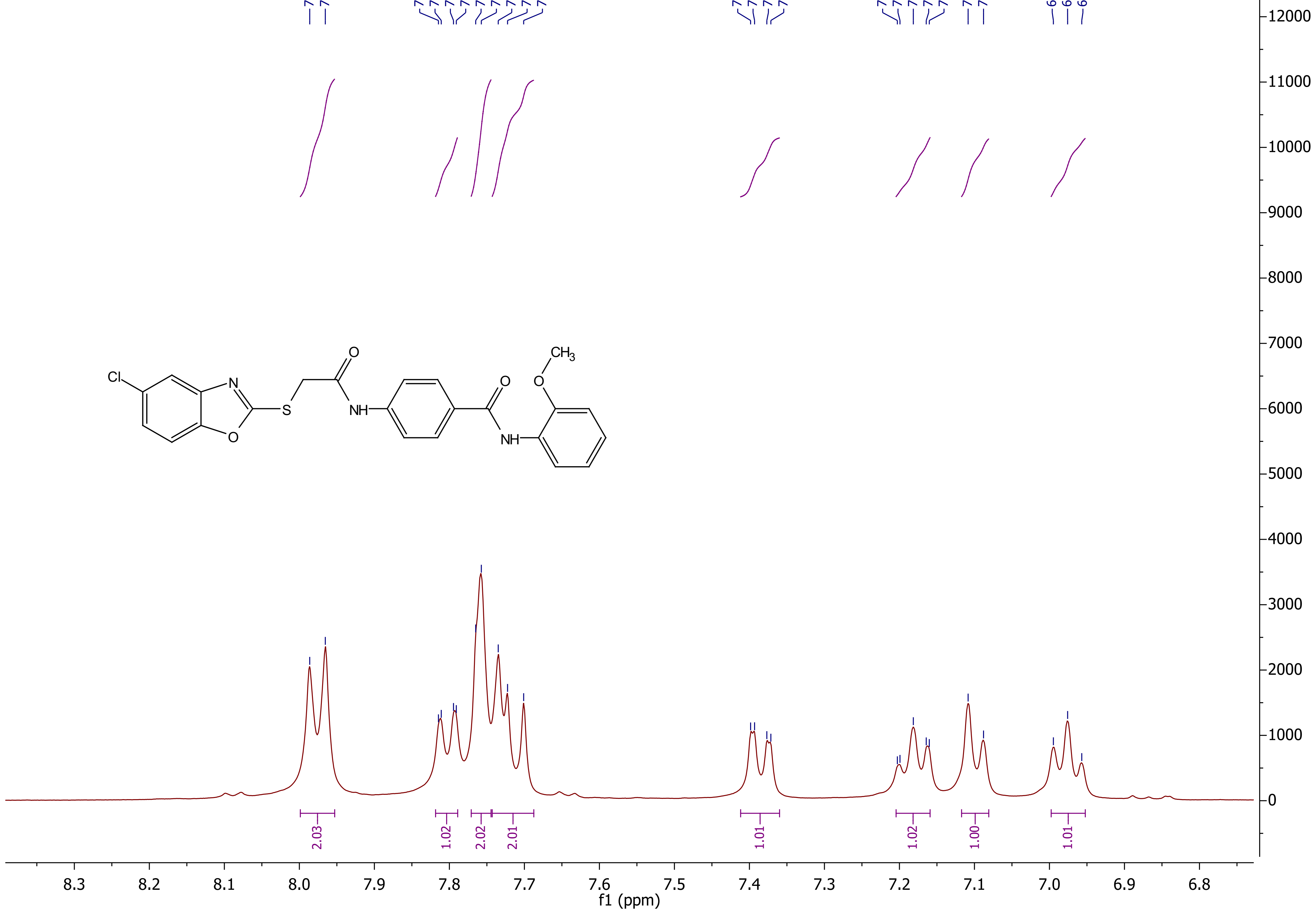
7.99
7.97
7.81
7.81
7.79
7.79
7.76
7.76
7.73
7.72
7.70
7.40
7.39
7.38
7.37
7.20
7.20
7.18
7.16
7.16
7.11
7.09
6.99
6.98
6.96
Cl
N
O
S
O
NH
O
NH
O
CH3
2.03
1.02
2.02
2.01
1.01
1.02
1.00
1.01
8.3
8.2
8.1
8.0
7.9
7.8
7.7
7.6
7.5
7.4
7.3
7.2
7.1
7.0
6.9
6.8
f1 (ppm)
12000
11000
10000
9000
8000
7000
6000
5000
4000
3000
2000
1000
0

13C NMR 14b

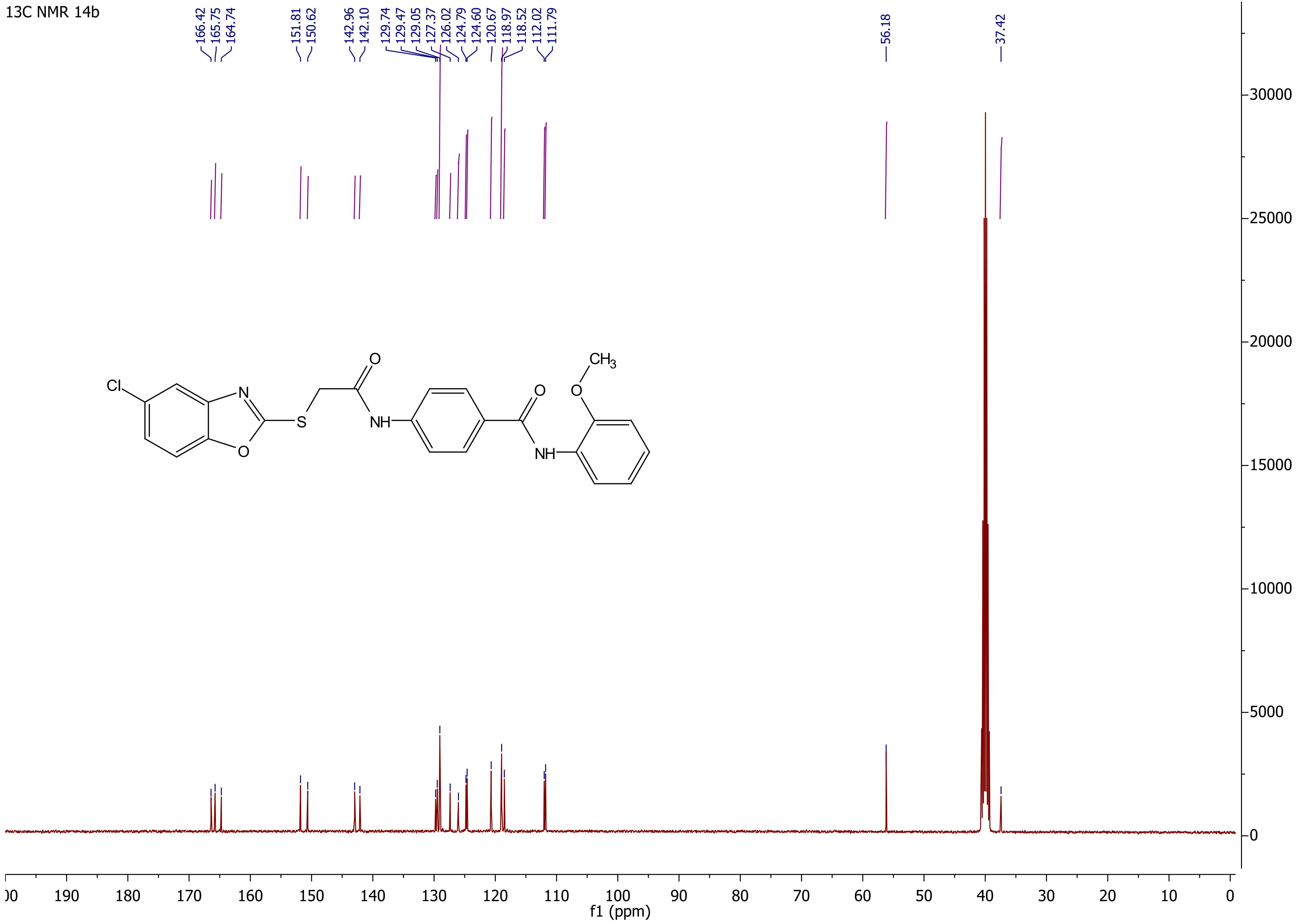

166.42
165.75
164.74
151.81
150.62
142.96
142.10
129.74
129.47
129.05
127.37
126.02
124.79
124.60
120.67
118.97
118.52
112.02
111.79
56.18
37.42
Cl
N
O
S
O
NH
O
NH
O
CH3
f1 (ppm)

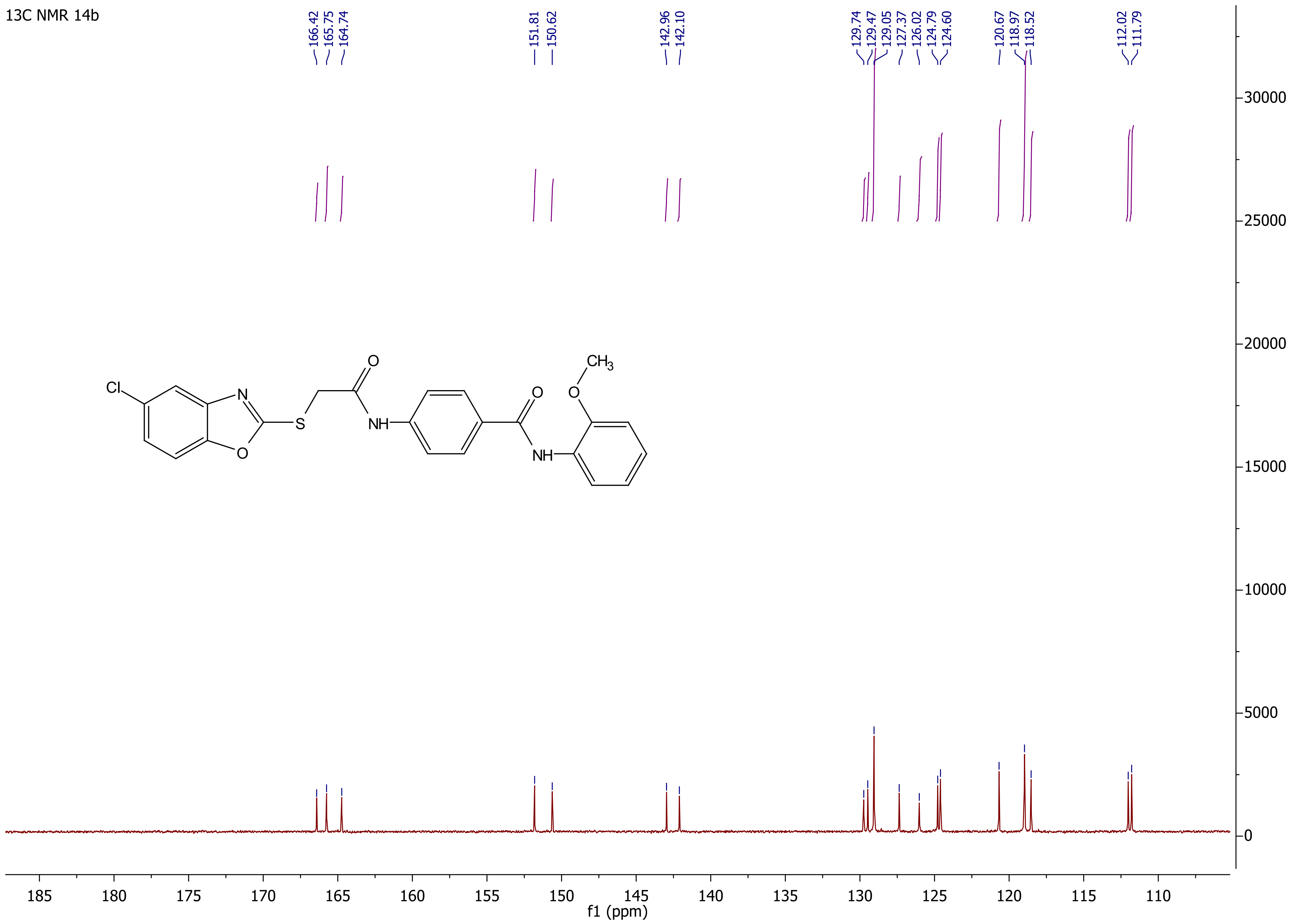

13C NMR 14b
166.42
165.75
164.74
151.81
150.62
142.96
142.10
129.74
129.47
129.05
127.37
126.02
124.79
124.60
120.67
118.97
118.52
112.02
111.79
Cl
N
O
S
O
NH
O
NH
O
CH3
f1 (ppm)

IR of comp. 14c

Peak Find - 1.jws

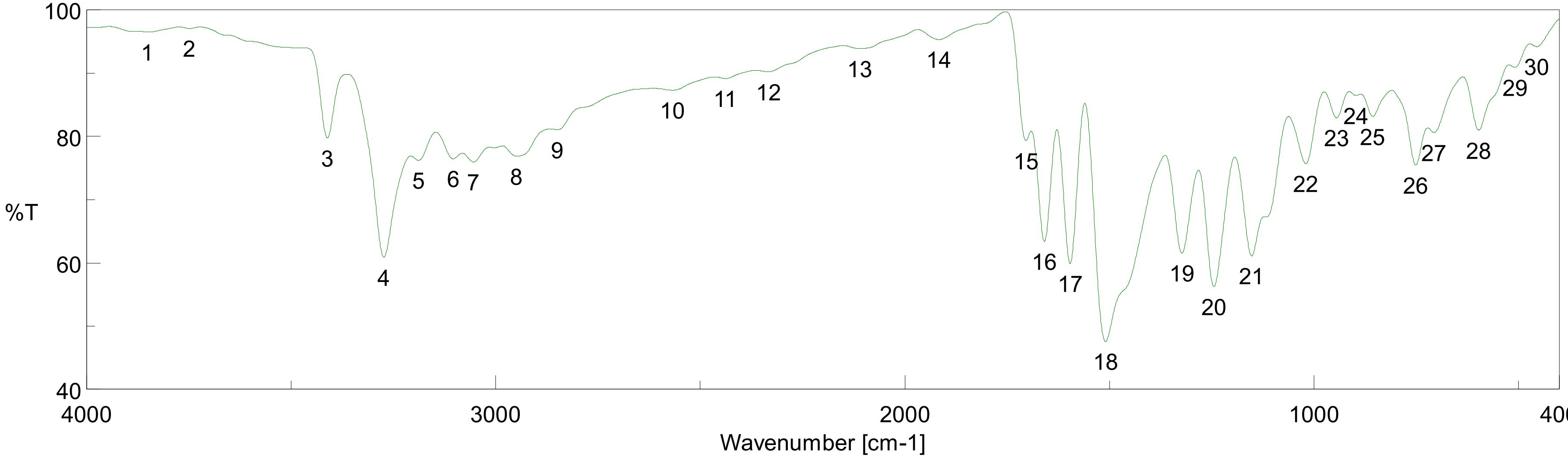

[Result of Peak Picking]

No.	Position	Intensity	No.	Position	Intensity	No.	Position	Intensity	No.	Position	Intensity
1	3852.11	96.4572	2	3748.94	97.0208	3	3411.46	79.7014	4	3273.57	60.8443
5	3188.72	76.1799	6	3103.87	76.4259	7	3054.69	75.9133	8	2948.63	76.8484
9	2849.31	81.0115	10	2567.75	87.2681	11	2438.55	89.1037	12	2333.45	90.1777
13	2110.71	93.8404	14	1916.9	95.2791	15	1703.8	79.2566	16	1658.48	63.3565
17	1595.81	59.8736	18	1509.03	47.5367	19	1322.93	61.5085	20	1243.86	56.2216
21	1152.26	61.0737	22	1019.19	75.6582	23	943.985	82.9053	24	896.737	86.4234
25	856.239	83.1116	26	751.138	75.4389	27	705.819	80.5725	28	596.861	80.9407
29	508.151	90.8596	30	455.118	94.16						

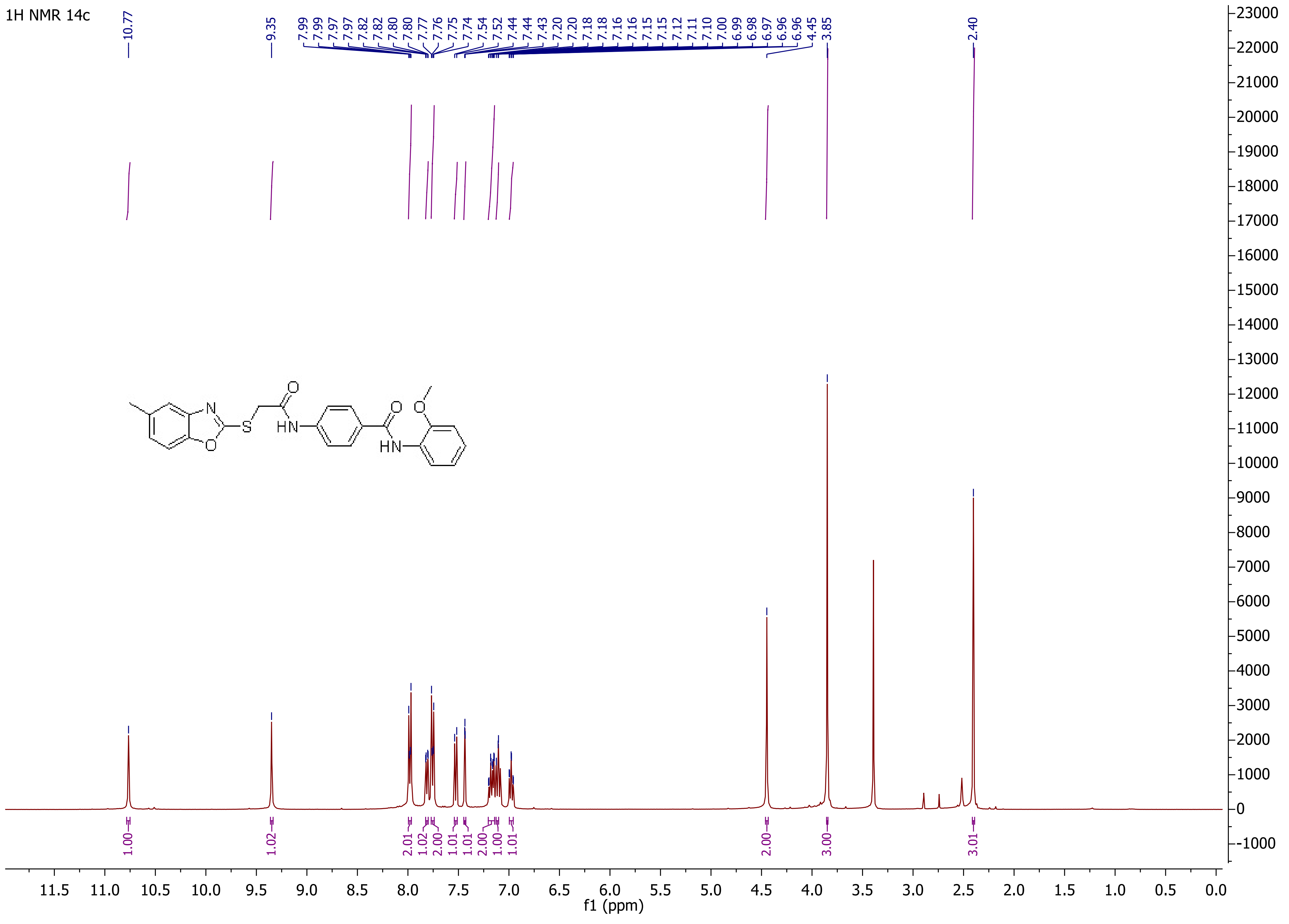
1H NMR 14c
10.77
9.35
7.99 7.99 7.97 7.97 7.82 7.82 7.80 7.80 7.77 7.76 7.75 7.74 7.54 7.52 7.44 7.44 7.43 7.20 7.20 7.18 7.18 7.16 7.16 7.15 7.15 7.12 7.11 7.10 7.00 6.99 6.98 6.97 6.96 6.96 4.45 3.85
2.40
N
S
O
HN
O
O
HN
1.00
1.02
2.01
1.02
2.00
1.01
1.01
2.00
1.00
1.01
2.00
3.00
3.01
f1 (ppm)

1H NMR 14c

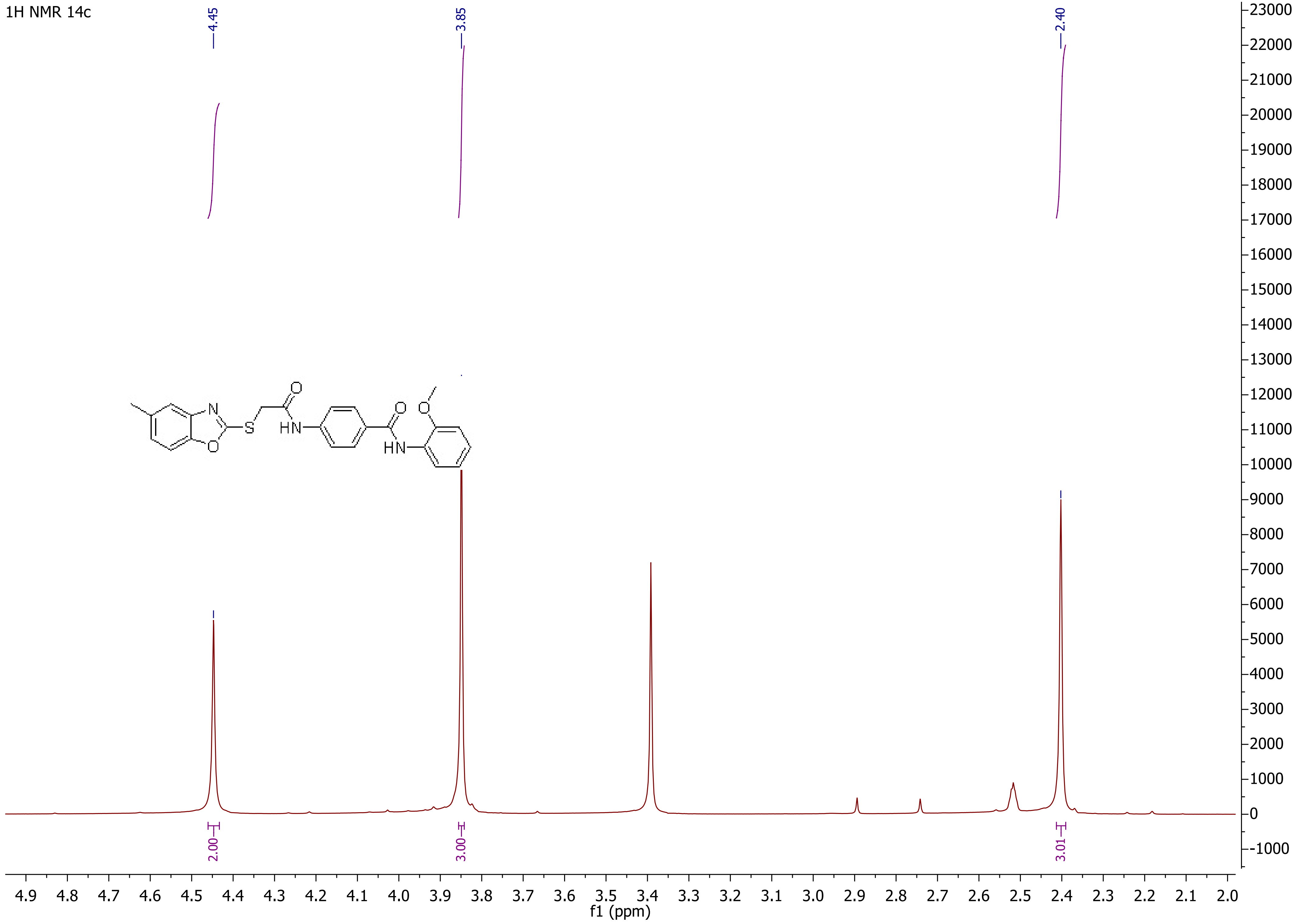

1H NMR 14c

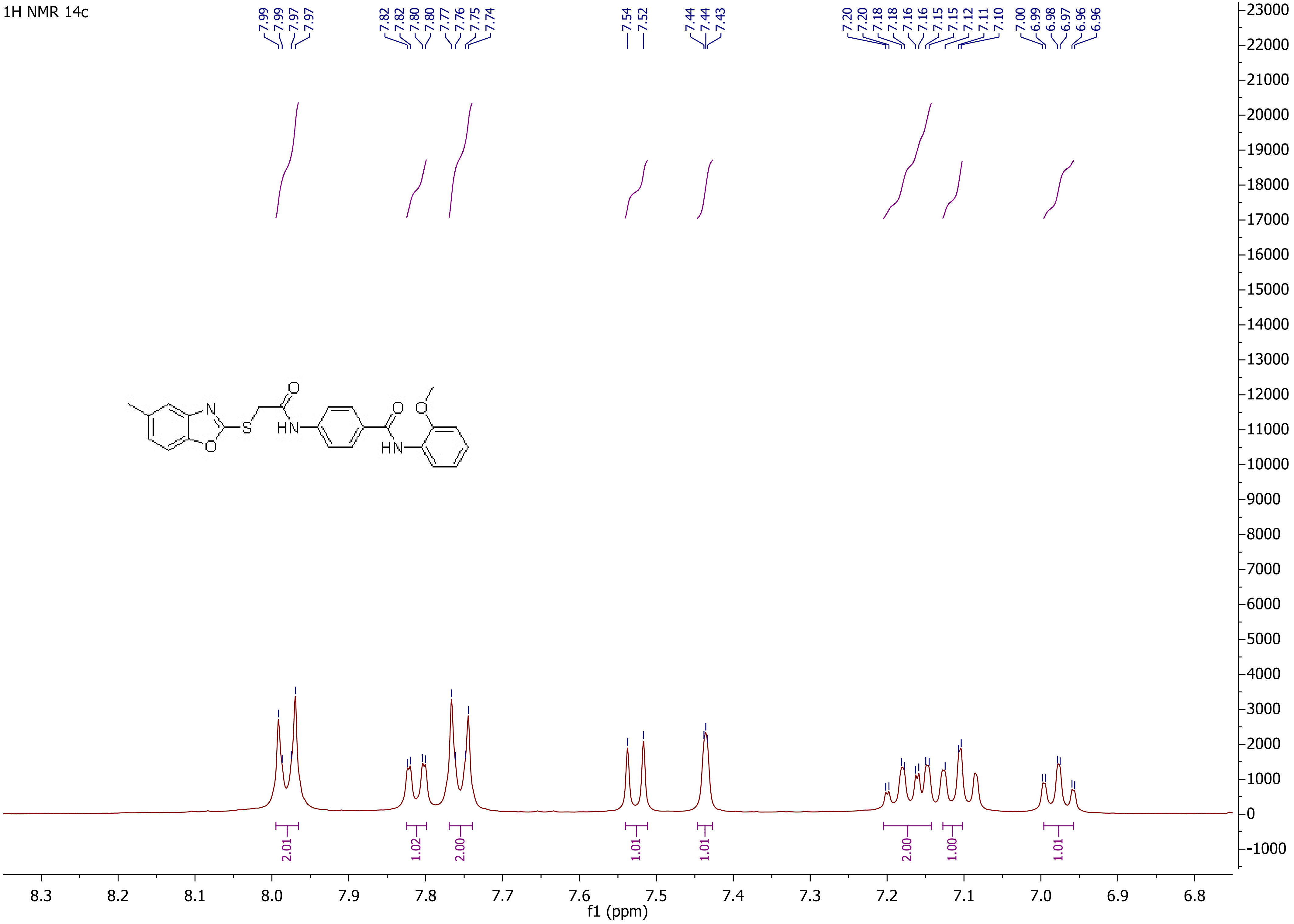
7.99
7.99
7.97
7.97
7.82
7.82
7.80
7.80
7.77
7.76
7.75
7.74
7.54
7.52
7.44
7.44
7.43
7.20
7.20
7.18
7.18
7.16
7.16
7.15
7.15
7.12
7.11
7.10
7.00
6.99
6.98
6.97
6.96
6.96
2.01
1.02
2.00
1.01
1.01
2.00
1.00
1.01
f1 (ppm)

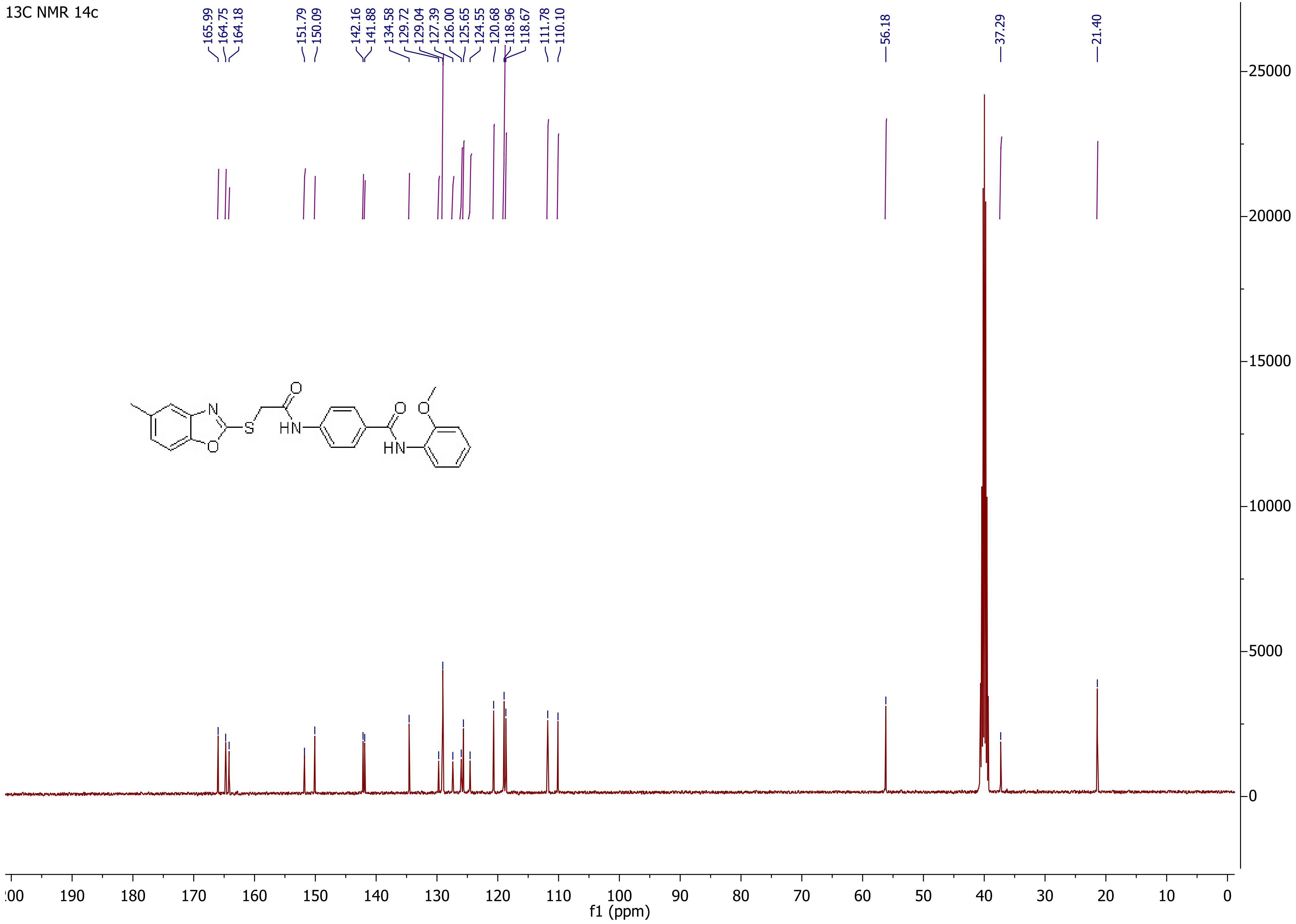

13C NMR 14c
165.99
164.75
164.18
151.79
150.09
142.16
141.88
134.58
129.72
129.04
127.39
126.00
125.65
124.55
120.68
118.96
118.67
111.78
110.10
56.18
37.29
21.40
25000
20000
15000
10000
5000
0
200
190
180
170
160
150
140
130
120
110
100
90
80
70
60
50
40
30
20
10
0
f1 (ppm)
N
O
S
O
HN
O
O
HN

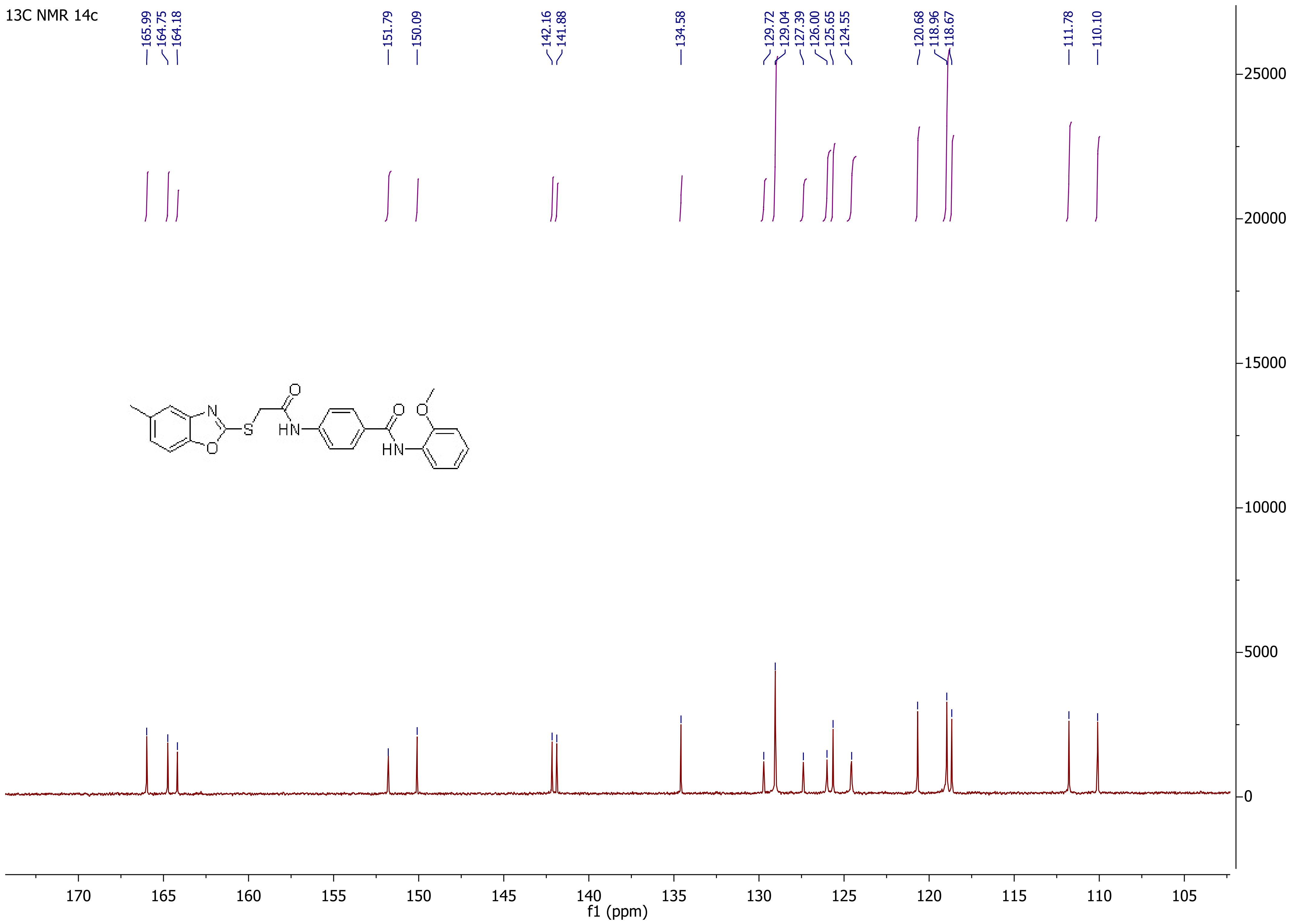
13C NMR 14c
165.99
164.75
164.18
151.79
150.09
142.16
141.88
134.58
129.72
129.04
127.39
126.00
125.65
124.55
120.68
118.96
118.67
111.78
110.10
25000
20000
15000
10000
5000
0
170
165
160
155
150
145
140
135
130
125
120
115
110
105
f1 (ppm)

IR of comp. 14d

Peak Find - 14.jws

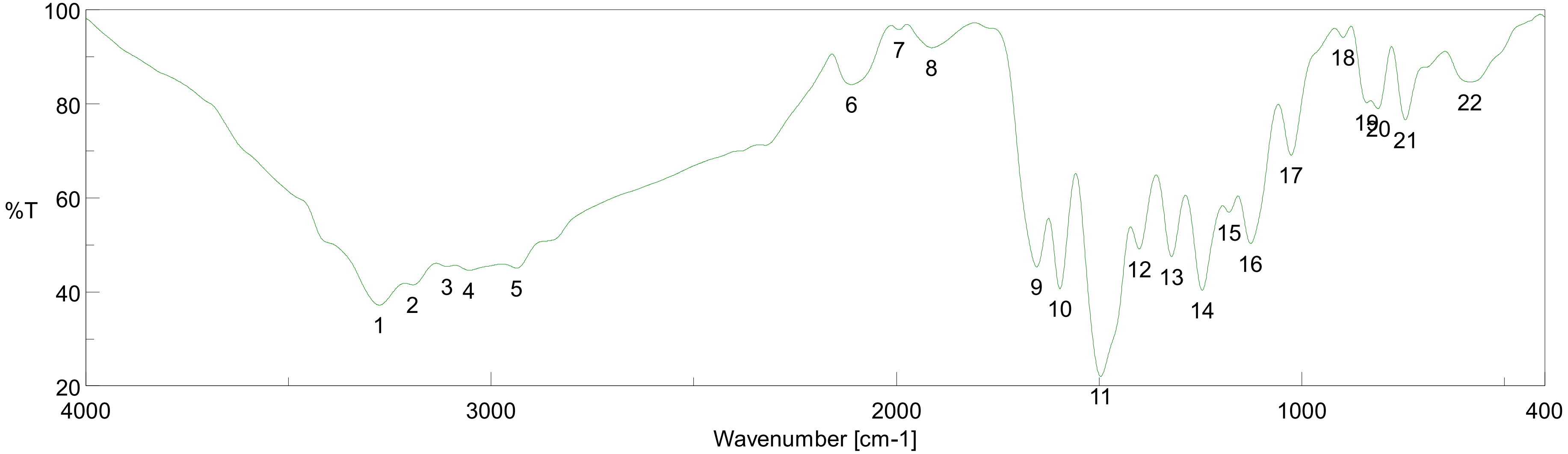

[Result of Peak Picking]

No.	Position	Intensity	No.	Position	Intensity	No.	Position	Intensity	No.	Position	Intensity
1	3276.47	37.1608	2	3193.54	41.4739	3	3108.69	45.3685	4	3054.69	44.5625
5	2937.06	45.0178	6	2110.71	84.0357	7	1993.07	95.7576	8	1913.04	91.8765
9	1653.66	45.2917	10	1596.77	40.6553	11	1495.53	22.021	12	1401.03	49.1059
13	1321	47.4503	14	1245.79	40.3128	15	1179.26	56.8664	16	1126.22	50.2319
17	1025.94	69.0539	18	897.701	94.0672	19	839.847	80.2092	20	810.92	78.9396
21	743.424	76.5802	22	584.325	84.6001						

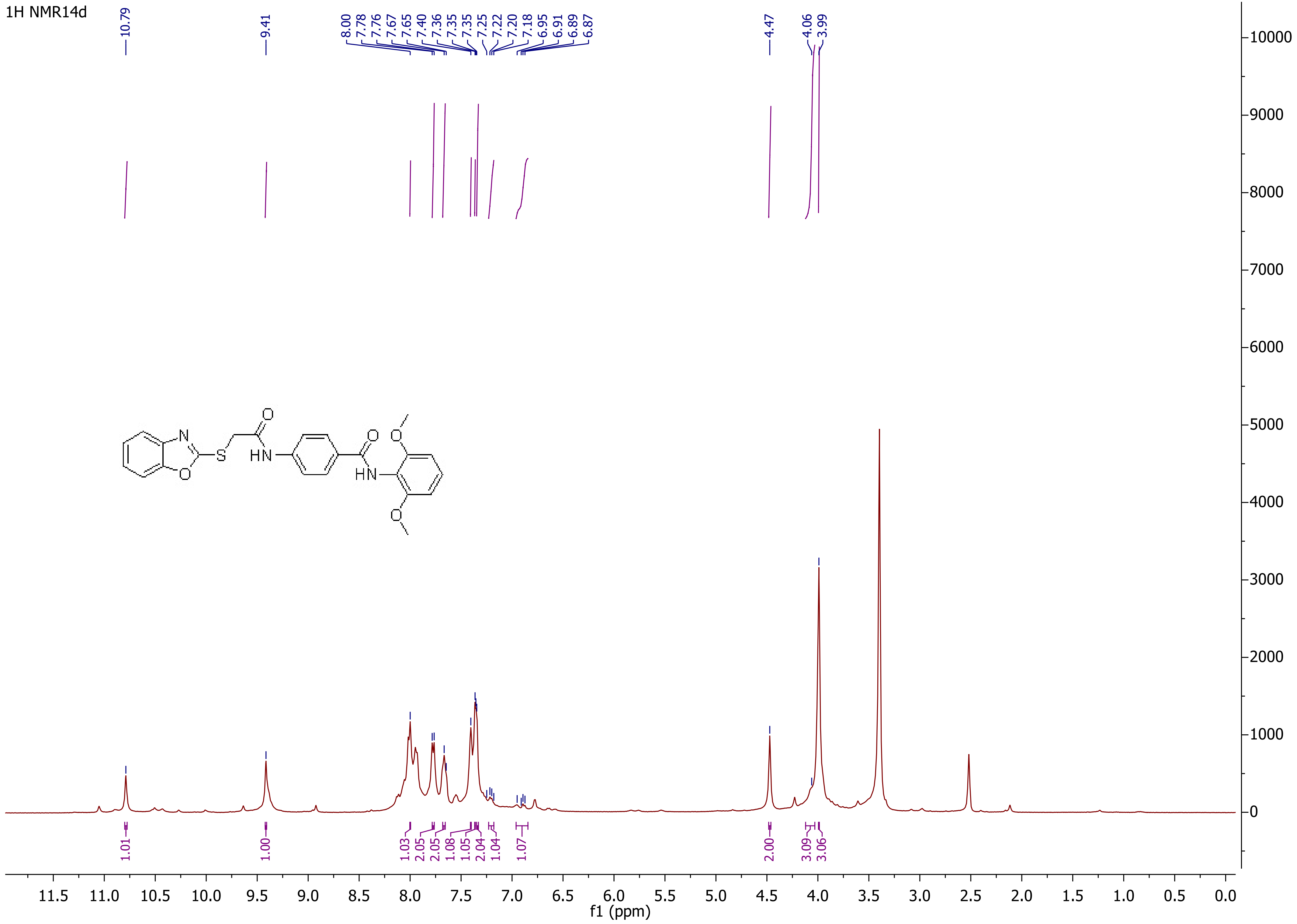
1H NMR14d
10.79
9.41
8.00
7.78
7.76
7.67
7.65
7.40
7.36
7.35
7.35
7.25
7.22
7.20
7.18
6.95
6.91
6.89
6.87
4.47
4.06
3.99
1.01
1.00
1.03
2.05
2.05
1.08
1.05
2.04
1.04
1.07
2.00
3.09
3.06
f1 (ppm)

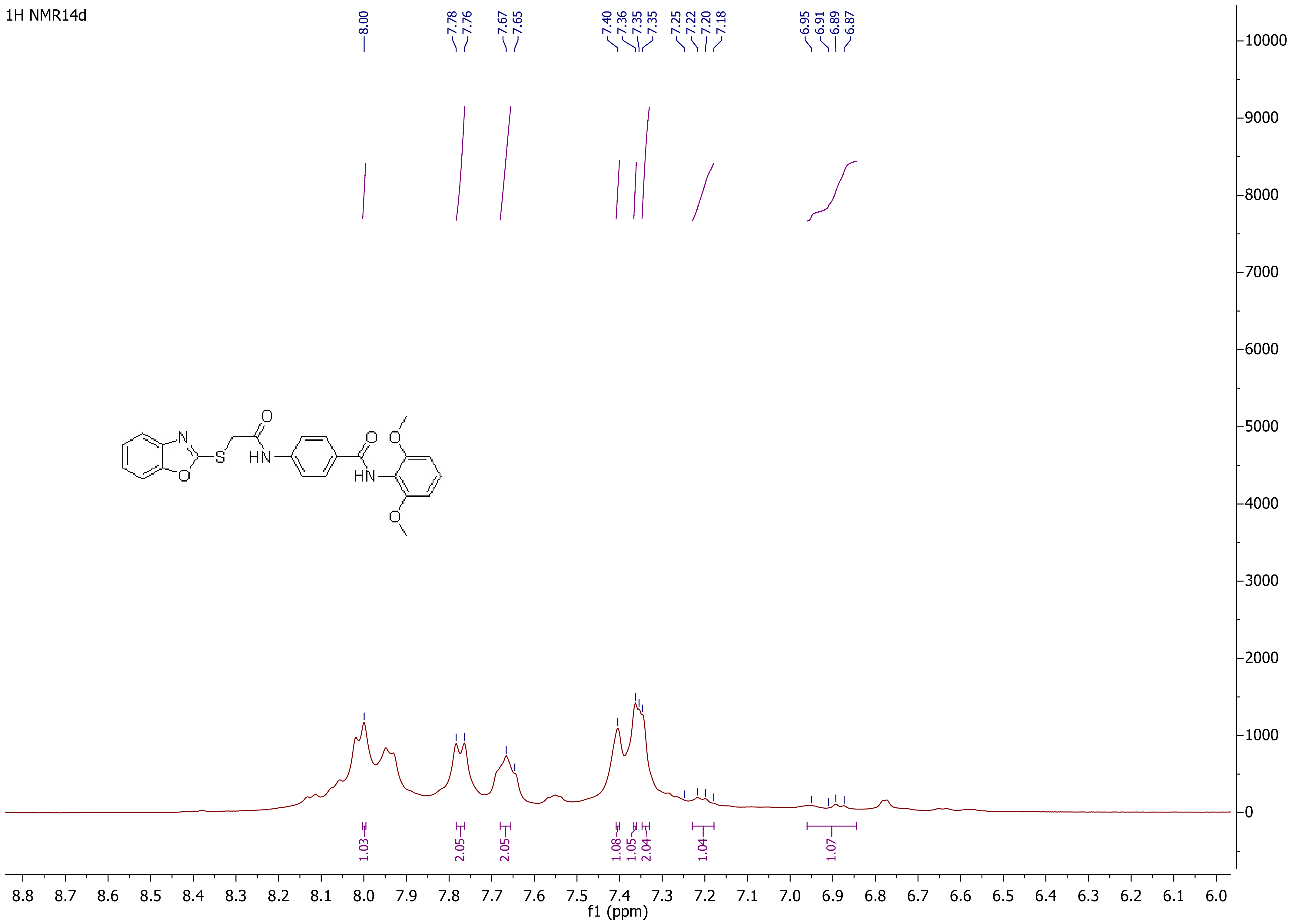
1H NMR14d
8.00
7.78
7.76
7.67
7.65
7.40
7.36
7.35
7.35
7.25
7.22
7.20
7.18
6.95
6.91
6.89
6.87
1.03
2.05
2.05
1.08
1.05
2.04
1.04
1.07
f1 (ppm)

13C NMR14d

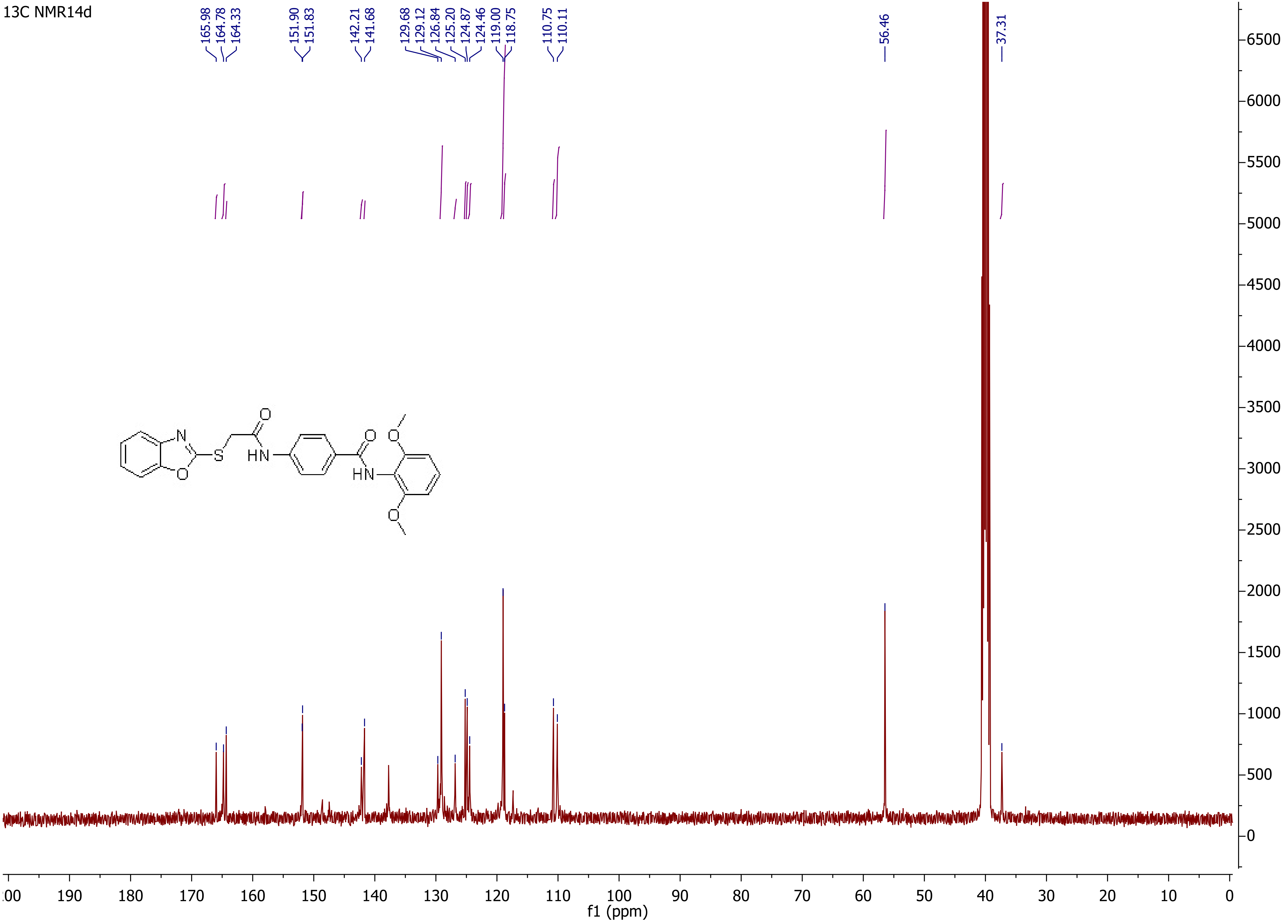
165.98
164.78
164.33
151.90
151.83
142.21
141.68
129.68
129.12
126.84
125.20
124.87
124.46
119.00
118.75
110.75
110.11
56.46
37.31
f1 (ppm)

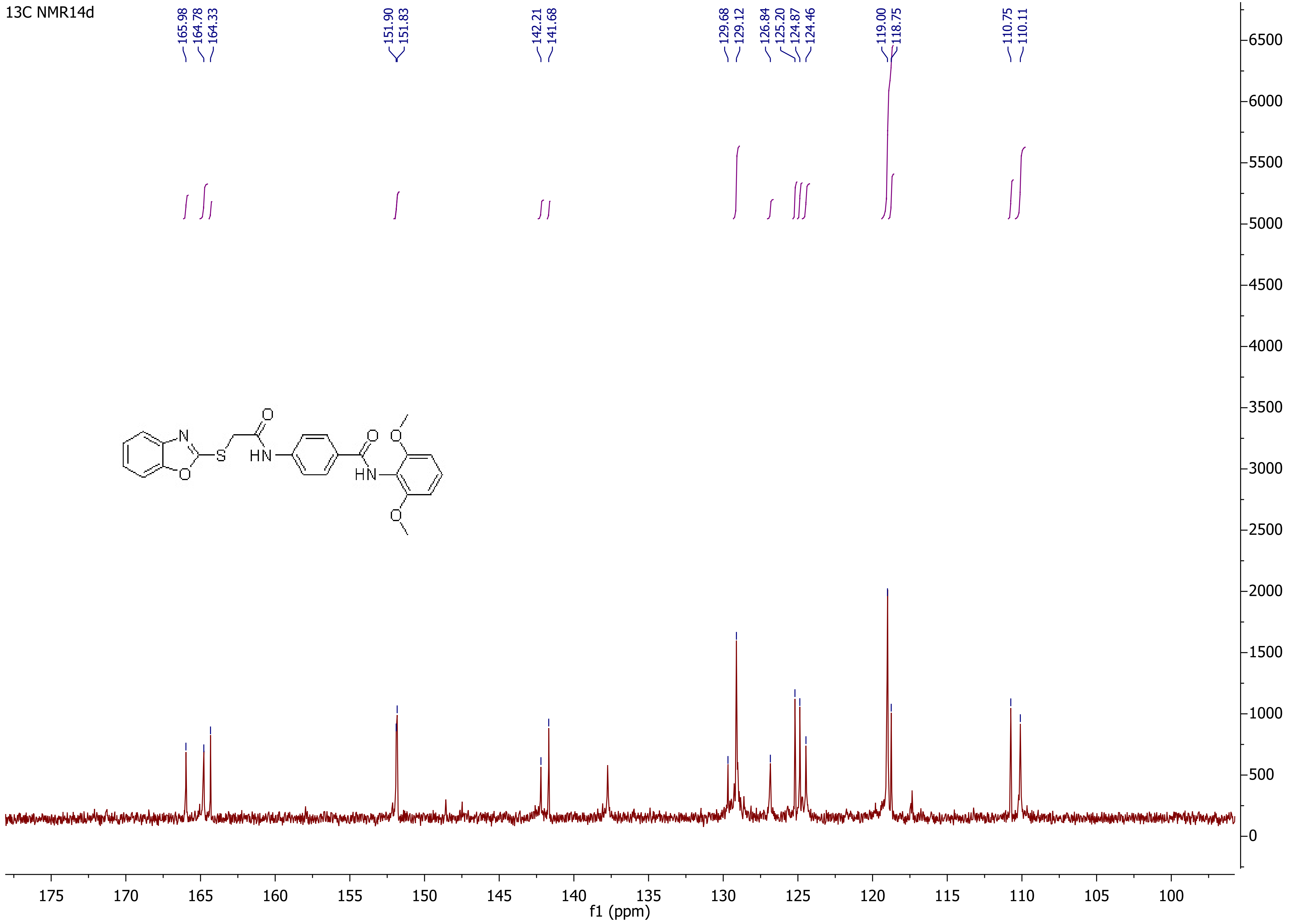
13C NMR14d
165.98
164.78
164.33
151.90
151.83
142.21
141.68
129.68
129.12
126.84
125.20
124.87
124.46
119.00
118.75
110.75
110.11
f1 (ppm)

IR of comp. 14e

Peak Find - 9.jws

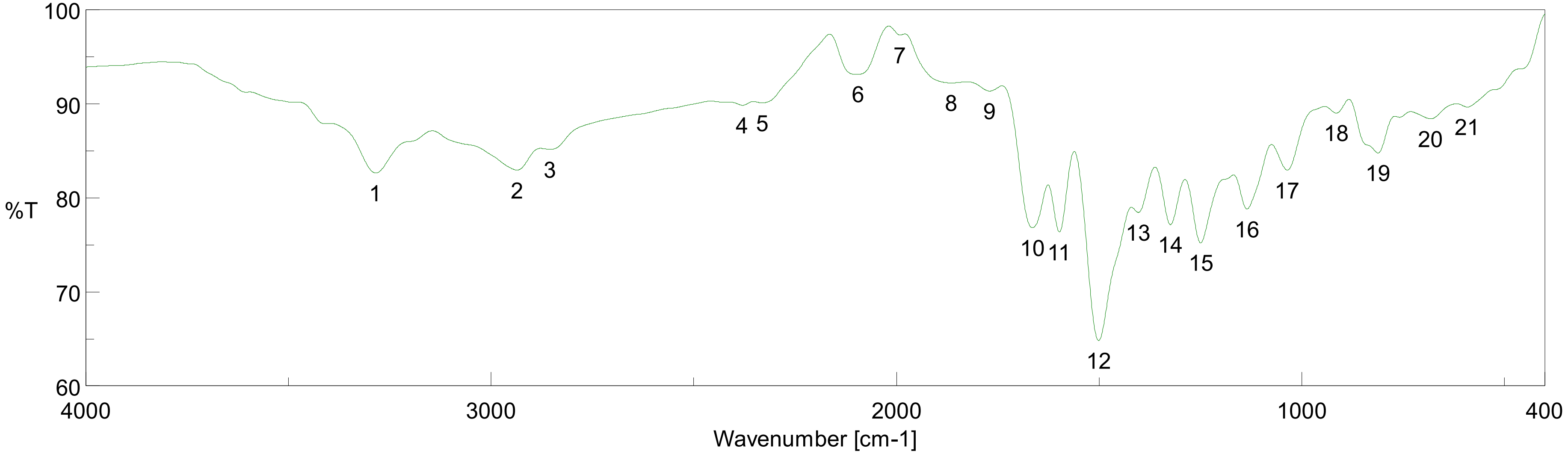

[Result of Peak Picking]

No.	Position	Intensity	No.	Position	Intensity	No.	Position	Intensity	No.	Position	Intensity
1	3285.14	82.6266	2	2936.09	82.9373	3	2854.13	85.144	4	2379.73	89.8246
5	2330.55	90.0798	6	2094.32	93.1203	7	1991.14	97.2974	8	1863.86	92.2013
9	1769.37	91.3348	10	1664.27	76.8049	11	1597.73	76.3487	12	1501.31	64.8097
13	1402.96	78.4258	14	1323.89	77.128	15	1248.68	75.2014	16	1134.9	78.7844
17	1035.59	82.9295	18	914.093	88.9966	19	811.885	84.7545	20	681.713	88.3959
21	591.075	89.6386									

1H NMR 14e

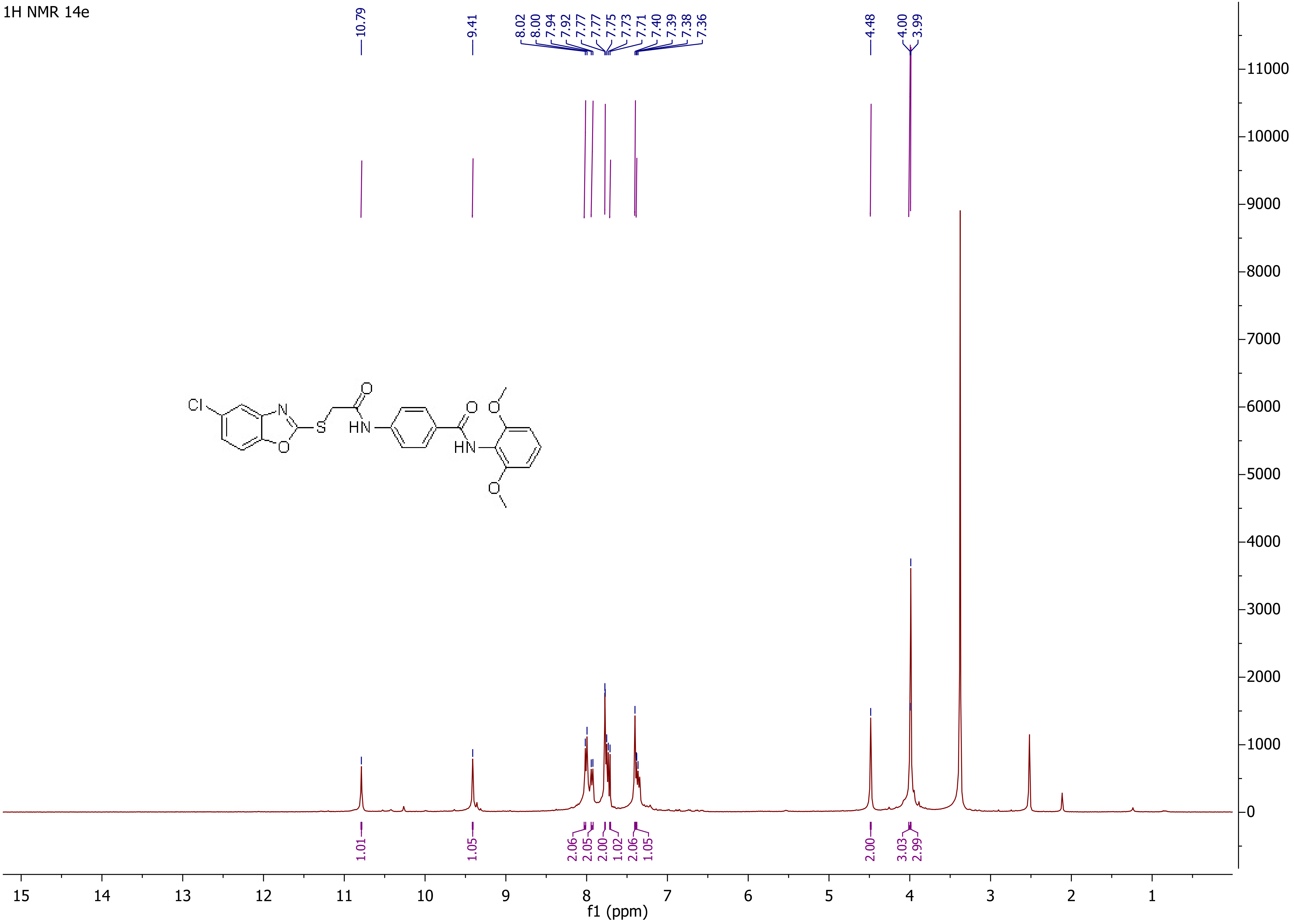
10.79
9.41
8.02 8.00 7.94 7.92 7.77 7.77 7.75 7.73 7.71 7.40 7.39 7.38 7.36
4.48
4.00 3.99
Cl
N
O
S
HN
HN
O
O
O
O
1.01
1.05
2.06 2.05 2.00 1.02 2.06 1.05
2.00
3.03 2.99
11000
10000
9000
8000
7000
6000
5000
4000
3000
2000
1000
0
15 14 13 12 11 10 9 8 7 6 5 4 3 2 1
f1 (ppm)

1H NMR 14e

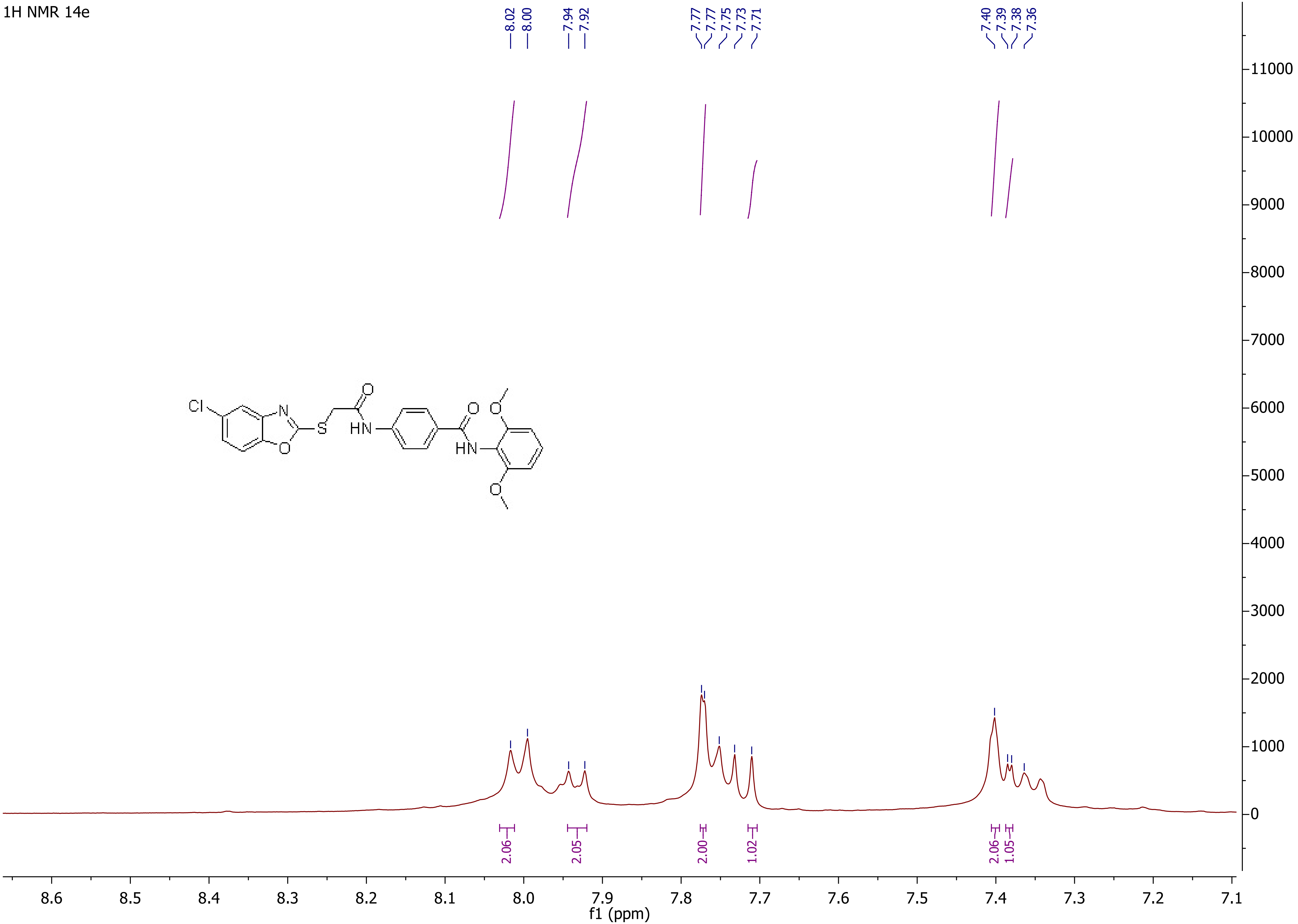

8.02
8.00
7.94
7.92
7.77
7.77
7.75
7.73
7.71
7.40
7.39
7.38
7.36
2.06
2.05
2.00
1.02
2.06
1.05
f1 (ppm)
Cl
N
S
O
HN
O
HN
O
O
O

13C NMR 14e

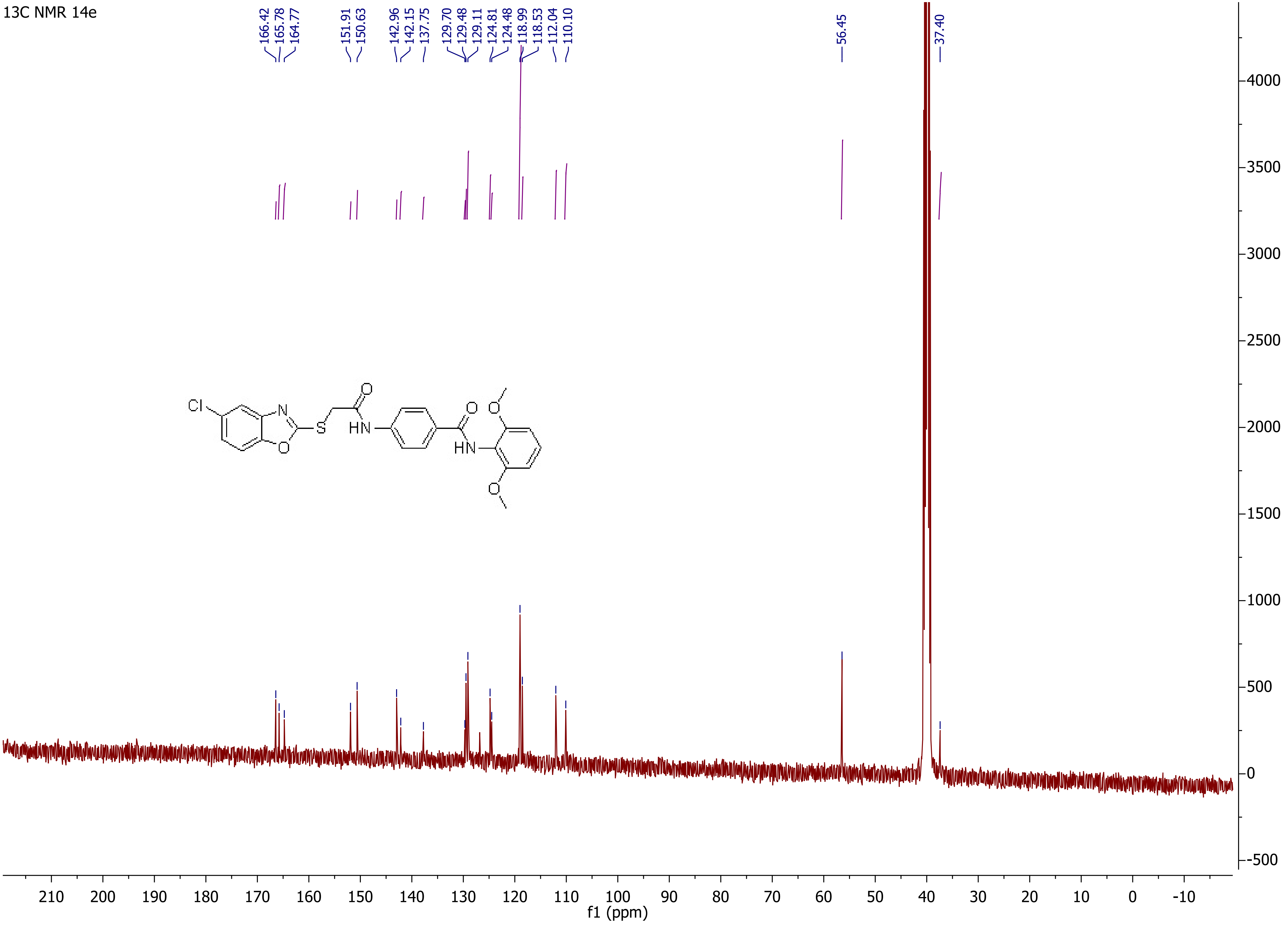

166.42
165.78
164.77
151.91
150.63
142.96
142.15
137.75
129.70
129.48
129.11
124.81
124.48
118.99
118.53
112.04
110.10
56.45
37.40
Cl
N
O
S
HN
O
O
HN
O
O
4000
3500
3000
2500
2000
1500
1000
500
0
-500
210 200 190 180 170 160 150 140 130 120 110 100 90 80 70 60 50 40 30 20 10 0 -10
f1 (ppm)

13C NMR 14e

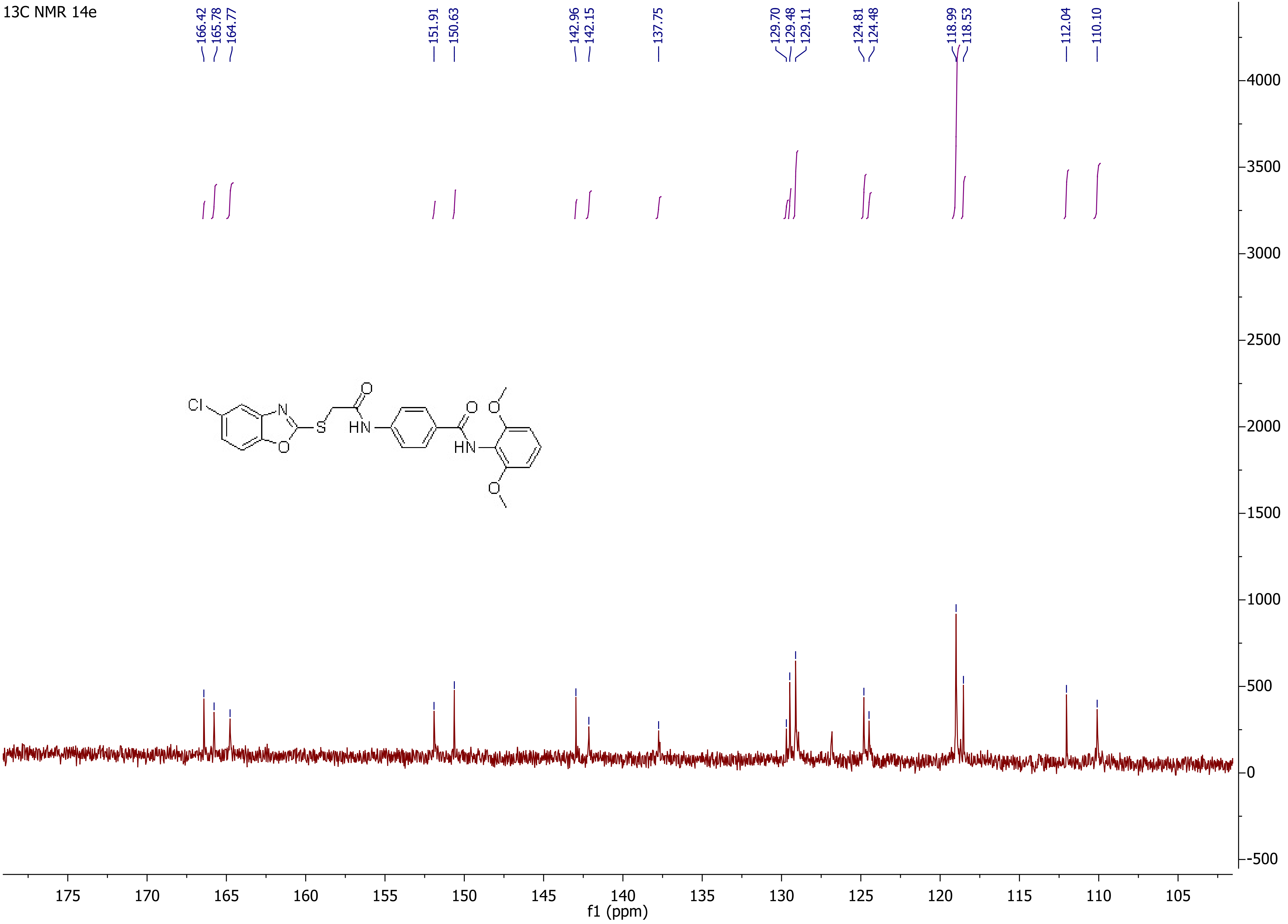
166.42
165.78
164.77
151.91
150.63
142.96
142.15
137.75
129.70
129.48
129.11
124.81
124.48
118.99
118.53
112.04
110.10
Cl
N
O
S
HN
O
O
HN
O
O
4000
3500
3000
2500
2000
1500
1000
500
0
-500
175
170
165
160
155
150
145
140
135
130
125
120
115
110
105
f1 (ppm)

IR of comp. 14f

Peak Find - 7.jws

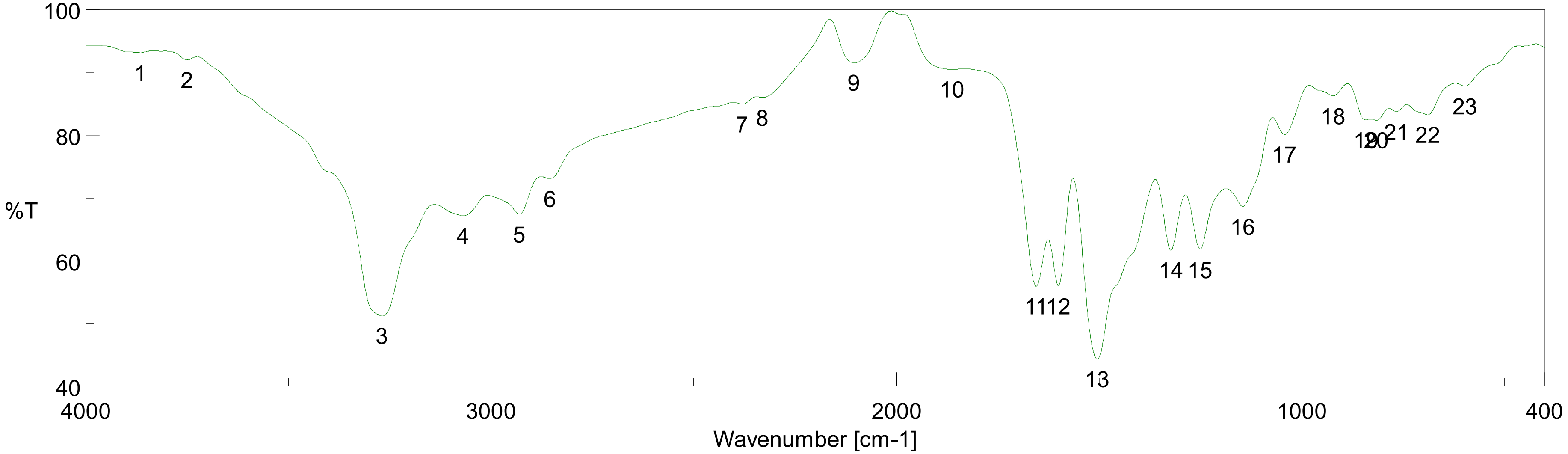

[Result of Peak Picking]

No.	Position	Intensity	No.	Position	Intensity	No.	Position	Intensity	No.	Position	Intensity
1	3865.61	93.116	2	3749.9	91.9923	3	3268.75	51.2055	4	3069.16	67.1565
5	2930.31	67.4031	6	2855.1	73.088	7	2379.73	84.9249	8	2330.55	86.0032
9	2104.92	91.5129	10	1862.9	90.4803	11	1654.62	55.945	12	1600.63	56.0001
13	1504.2	44.3115	14	1322.93	61.6804	15	1250.61	61.8141	16	1145.51	68.6469
17	1042.34	80.1058	18	922.771	86.2528	19	841.776	82.4124	20	815.742	82.3478
21	765.601	83.7349	22	689.427	83.2526	23	596.861	87.8229			

1H NMR 14f

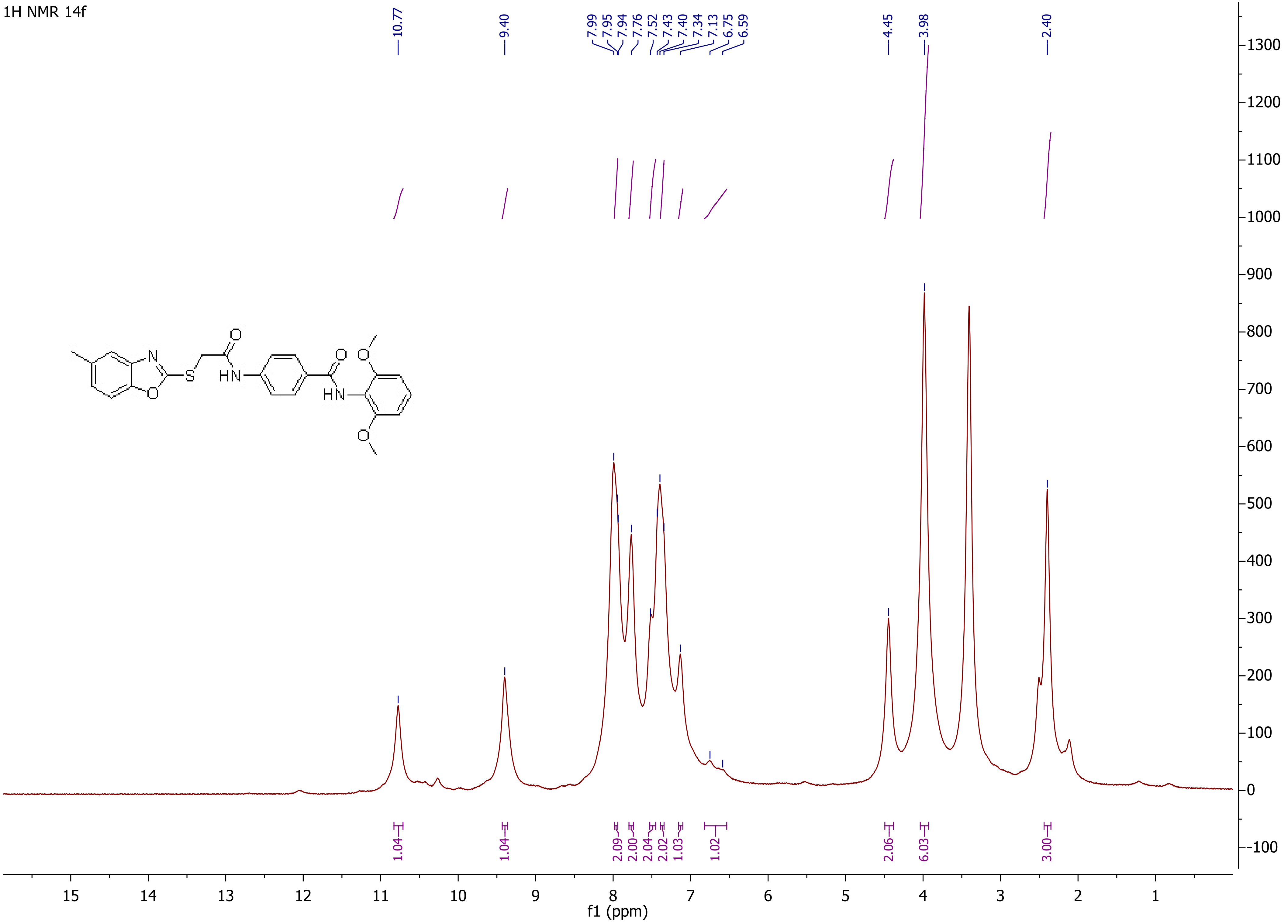

10.77
9.40
7.99
7.95
7.94
7.76
7.52
7.43
7.40
7.34
7.13
6.75
6.59
4.45
3.98
2.40
1.04
1.04
2.09
2.00
2.04
2.02
1.03
1.02
2.06
6.03
3.00
N
O
S
HN
O
HN
O
O
O
f1 (ppm)

1H NMR 14f

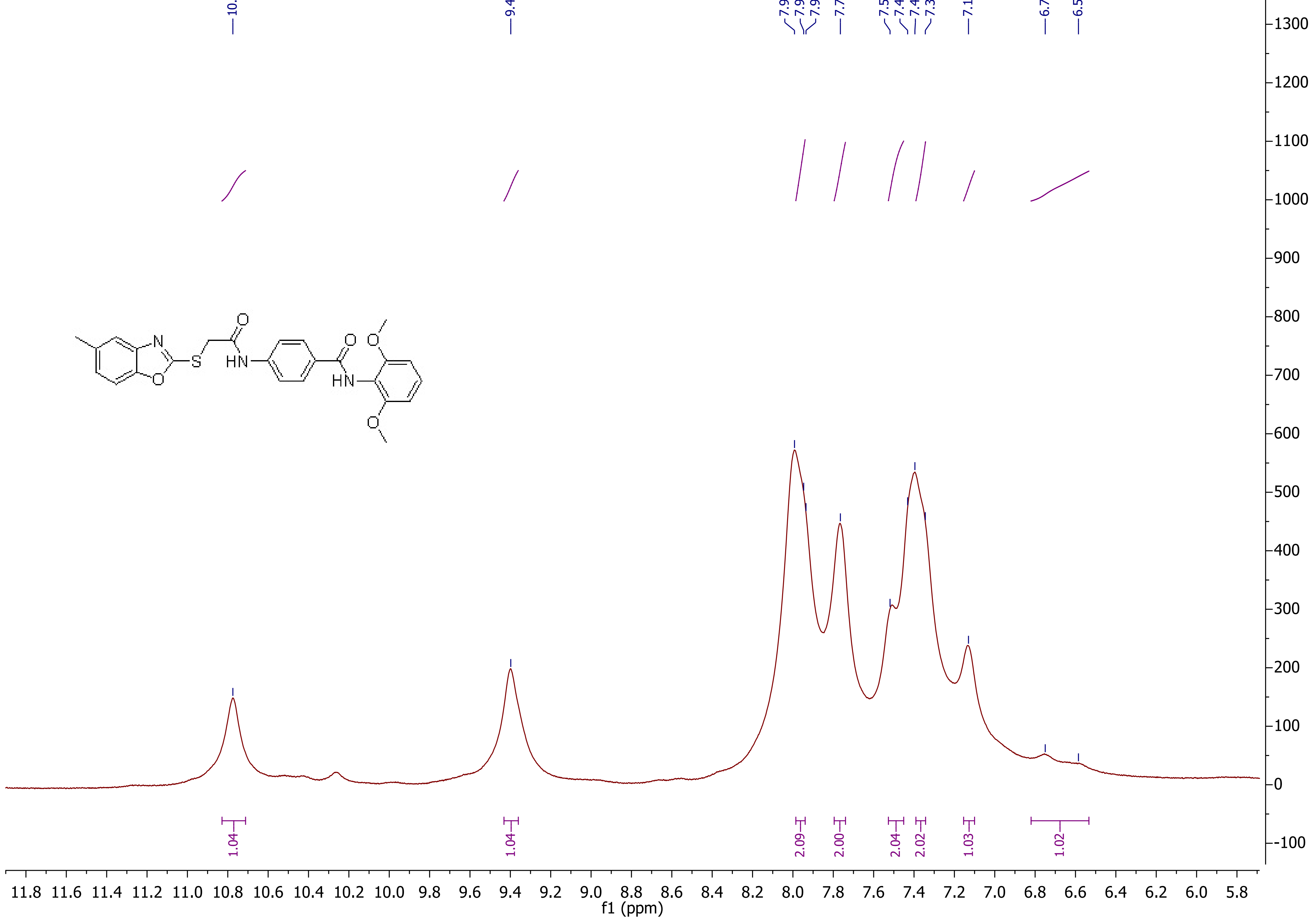

10.77
9.40
7.99
7.95
7.94
7.76
7.52
7.43
7.40
7.34
7.13
6.75
6.59
1.04
1.04
2.09
2.00
2.04
2.02
1.03
1.02
N
O
S
HN
O
O
HN
O
O
f1 (ppm)
11.8 11.6 11.4 11.2 11.0 10.8 10.6 10.4 10.2 10.0 9.8 9.6 9.4 9.2 9.0 8.8 8.6 8.4 8.2 8.0 7.8 7.6 7.4 7.2 7.0 6.8 6.6 6.4 6.2 6.0 5.8
1300 1200 1100 1000 900 800 700 600 500 400 300 200 100 0 -100

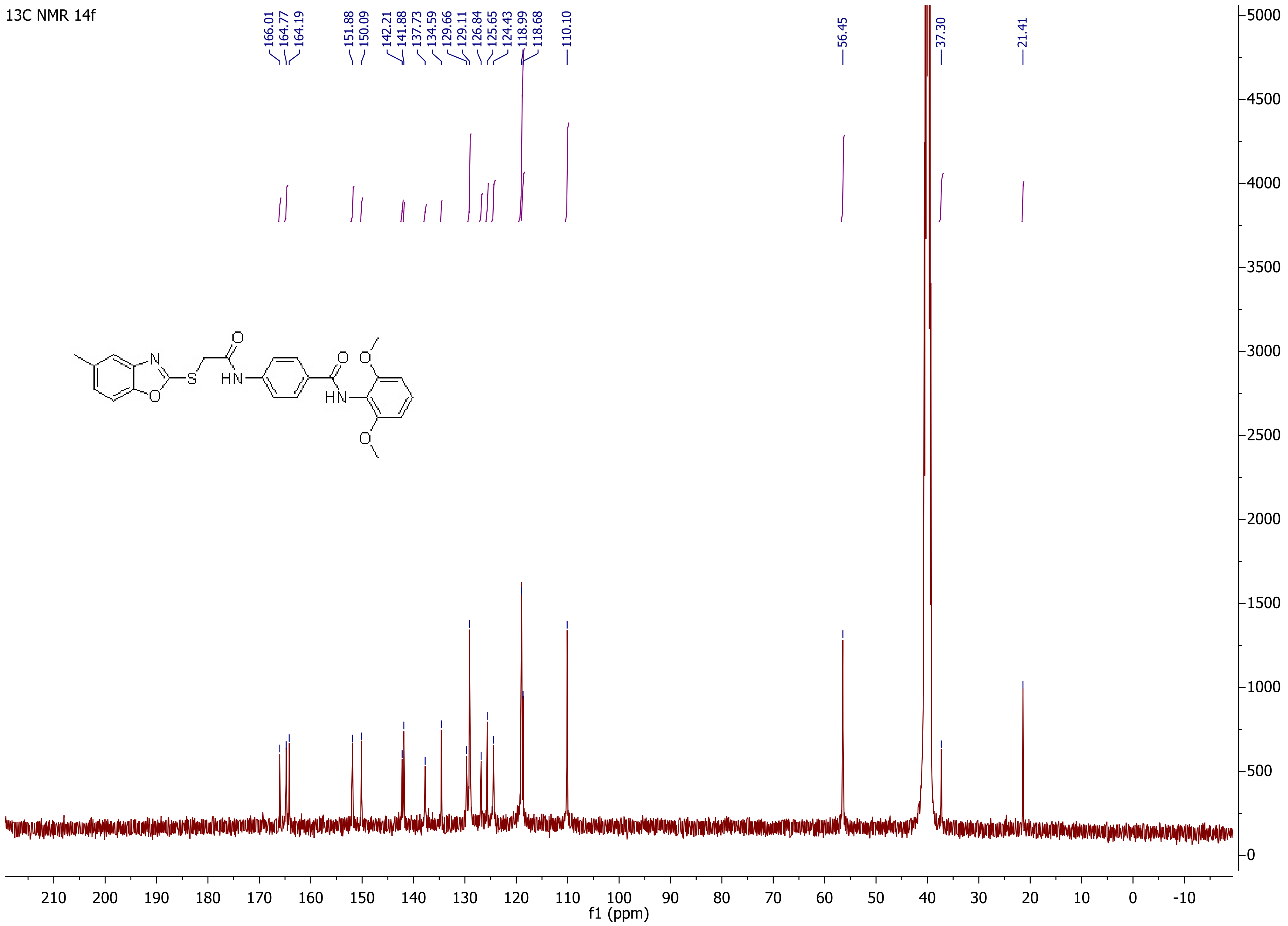
13C NMR 14f
166.01
164.77
164.19
151.88
150.09
142.21
141.88
137.73
134.59
129.66
129.11
126.84
125.65
124.43
118.99
118.68
110.10
56.45
37.30
21.41
f1 (ppm)

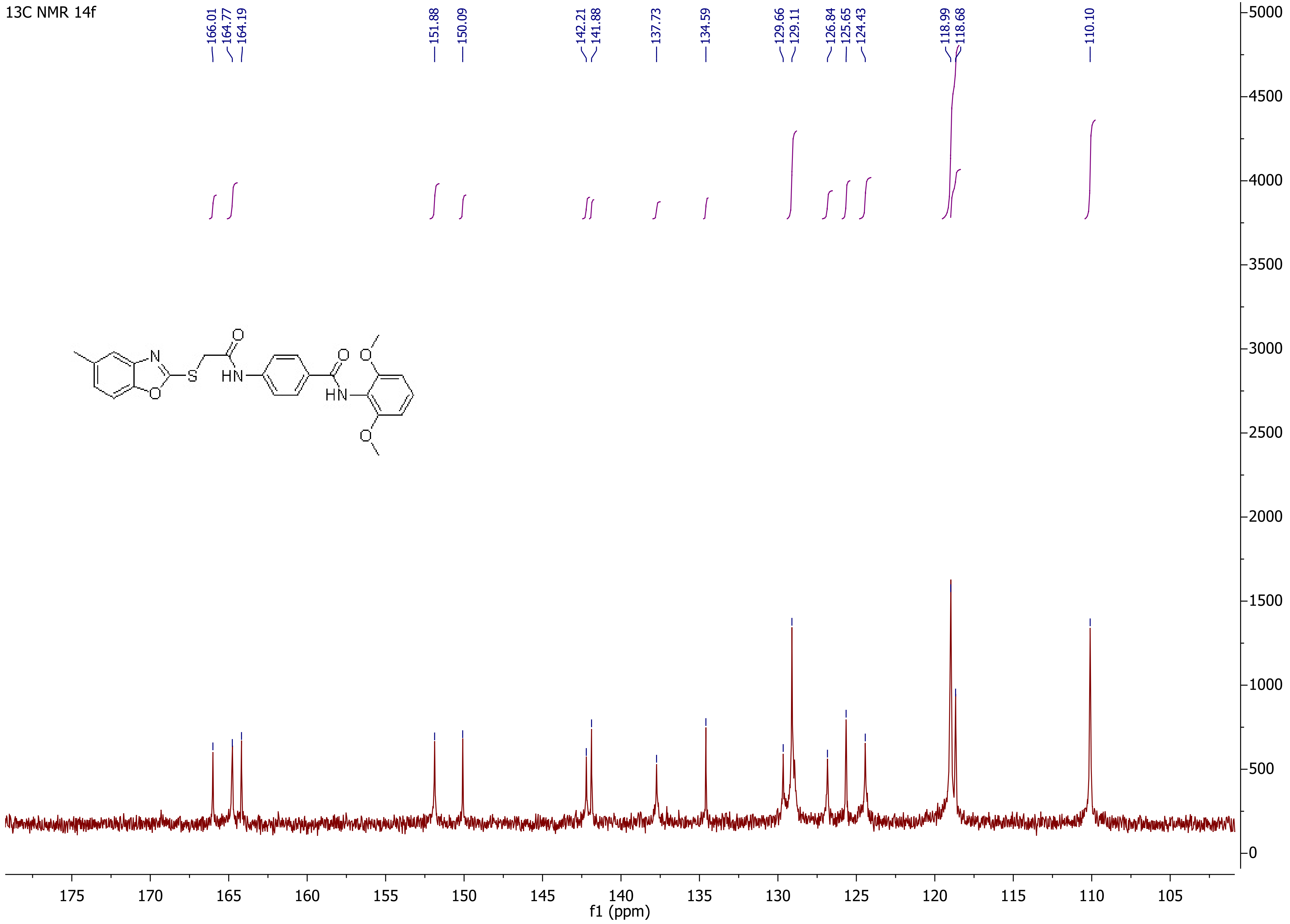
13C NMR 14f
166.01
164.77
164.19
151.88
150.09
142.21
141.88
137.73
134.59
129.66
129.11
126.84
125.65
124.43
118.99
118.68
110.10
f1 (ppm)

Peak Find - 5.jws

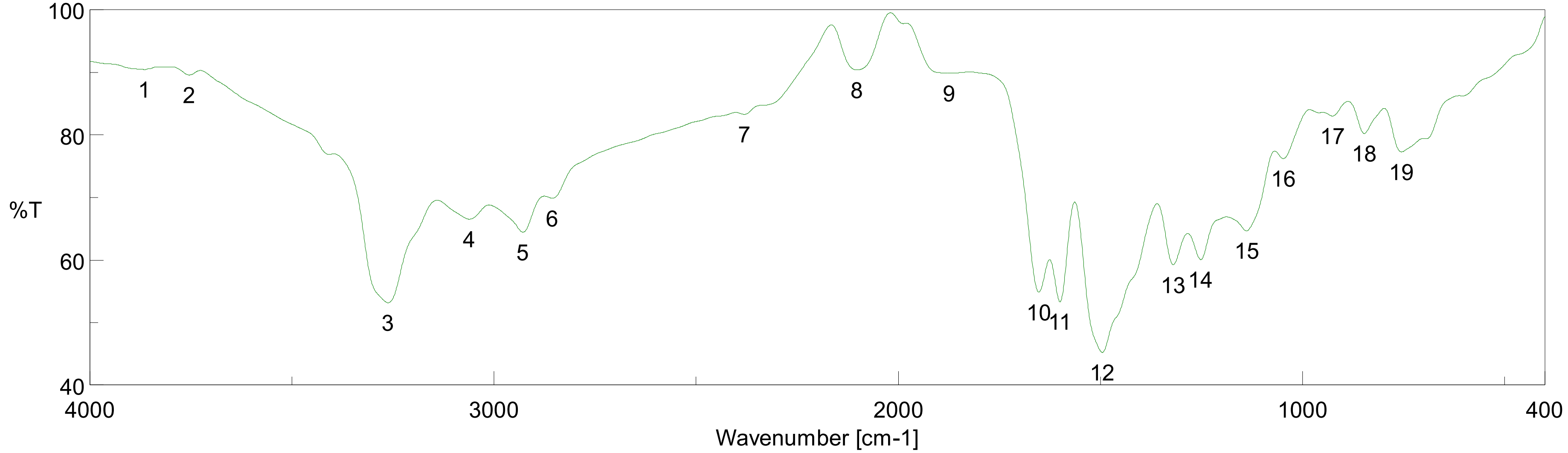

[Result of Peak Picking]

No.	Position	Intensity	No.	Position	Intensity	No.	Position	Intensity	No.	Position	Intensity
1	3865.61	90.4019	2	3754.73	89.5448	3	3262	53.1372	4	3061.44	66.4973
5	2928.38	64.4113	6	2856.06	69.8421	7	2380.69	83.2491	8	2102.03	90.3447
9	1873.51	89.8262	10	1651.73	54.8478	11	1599.66	53.2945	12	1495.53	45.2111
13	1320.04	59.2138	14	1251.58	60.0342	15	1137.8	64.6398	16	1047.16	76.1786
17	925.664	82.974	18	846.597	80.1986	19	754.995	77.2413			

Mass spec. of comp.14g

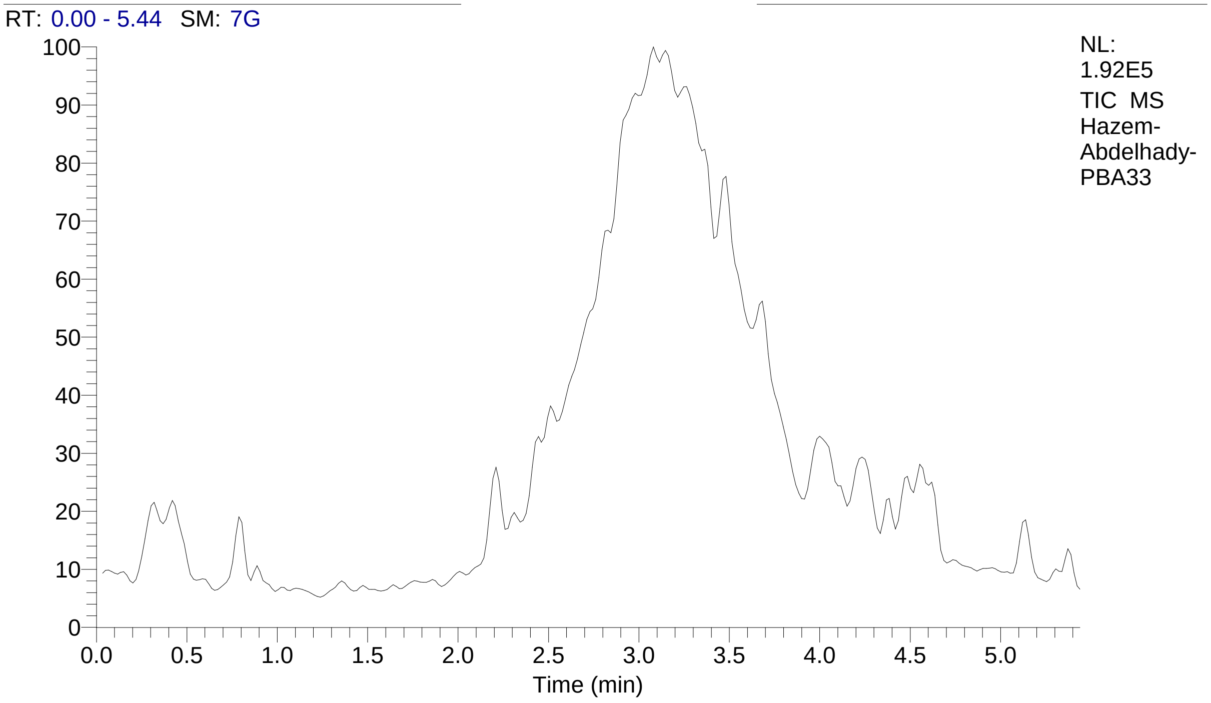

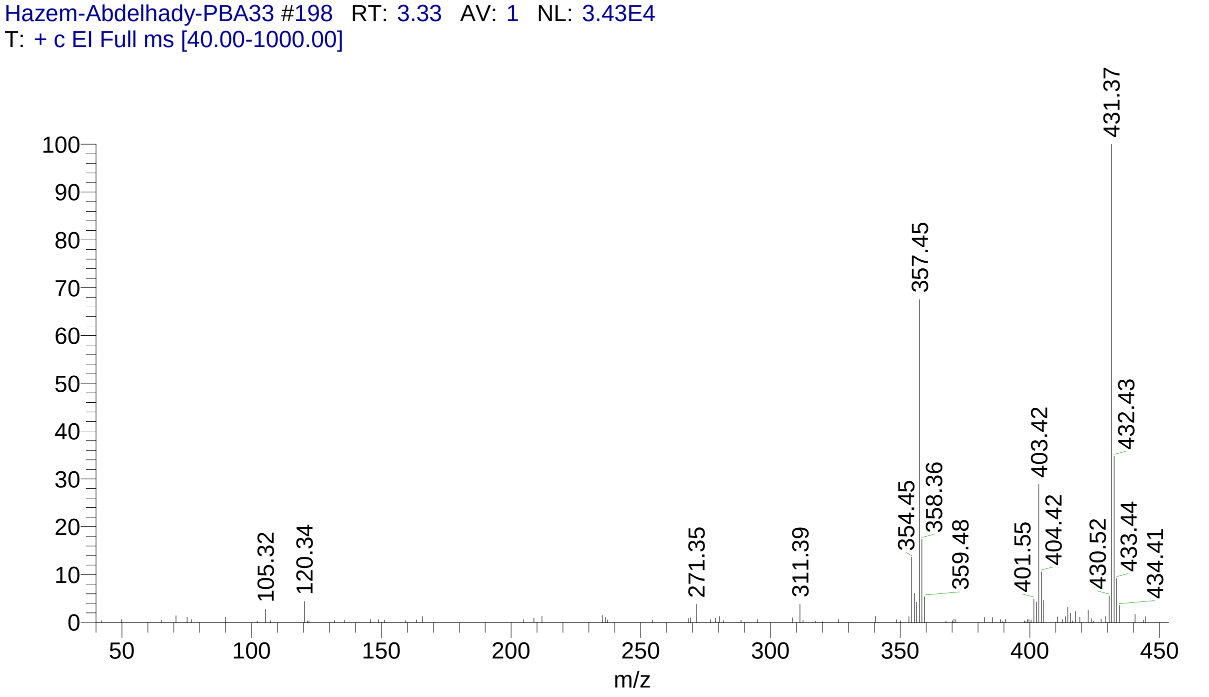

m/z	Intensity	Relative
42.03	134.8	0.39
49.82	210.8	0.62

65.22	136.3	0.40	
70.85	480.9	1.40	
75.16	375.4	1.10	
76.96	203.7	0.59	
89.83	361.3	1.05	
102.08	114.8	0.34	
105.32	934.1	2.73	
107.28	118.8	0.35	
120.34	1501.5		4.38
121.57	109.9	0.32	
122.08	109.9	0.32	
131.91	145.9	0.43	
135.97	165.1	0.48	
145.96	195.1	0.57	
149.07	201.6	0.59	
151.17	152.3	0.44	
159.27	132.4	0.39	
163.55	180.8	0.53	
165.96	407.8	1.19	
204.97	210.8	0.62	
208.82	315.3	0.92	
211.90	425.3	1.24	
235.34	491.4	1.43	
236.36	347.8	1.02	
237.25	182.3	0.53	
254.42	135.3	0.40	
268.28	270.2	0.79	
269.07	334.4	0.98	
271.35	1294.2		3.78
276.88	194.1	0.57	
278.84	319.8	0.93	
280.32	431.0	1.26	
281.88	128.7	0.38	
288.61	155.7	0.45	
295.11	200.3	0.58	
308.54	327.7	0.96	
311.39	1300.8		3.80
312.53	147.9	0.43	
317.47	94.9	0.28	
320.06	45.5	0.13	
326.23	202.9	0.59	
340.51	424.2	1.24	
348.66	199.7	0.58	
350.13	106.8	0.31	
353.35	406.2	1.19	
354.45	4649.4		13.57
355.44	2066.0		6.03
356.30	1437.2		4.20

357.45	23123.4		67.51
358.36	5948.4		17.37
359.48	1830.1		5.34
367.60	100.3	0.29	
370.13	128.9	0.38	
370.74	224.0	0.65	
371.40	197.7	0.58	
382.45	362.4	1.06	
385.60	347.7	1.02	
388.65	203.5	0.59	
389.52	80.8	0.24	
390.64	204.1	0.60	
397.85	115.2	0.34	
398.46	64.9	0.19	
399.06	191.9	0.56	
399.60	211.5	0.62	
400.46	175.5	0.51	
401.55	1680.0		4.90
402.56	1459.1		4.26
403.42	9890.7		28.88
404.42	3626.6		10.59
405.35	1575.1		4.60
410.74	393.3	1.15	
412.60	202.0	0.59	
413.63	414.8	1.21	
414.58	1095.5		3.20
415.58	665.2	1.94	
416.40	105.9	0.31	
417.57	782.6	2.28	
419.23	376.8	1.10	
422.47	875.4	2.56	
423.57	266.4	0.78	
424.58	84.4	0.25	
427.47	248.4	0.73	
429.26	427.2	1.25	
430.52	1900.3		5.55
431.37	34252.4		100.00
432.43	11898.3		34.74
433.44	3141.5		9.17
434.41	1198.9		3.50
440.48	579.7	1.69	
443.87	156.4	0.46	
444.42	413.0	1.21	

1H NMR 14g

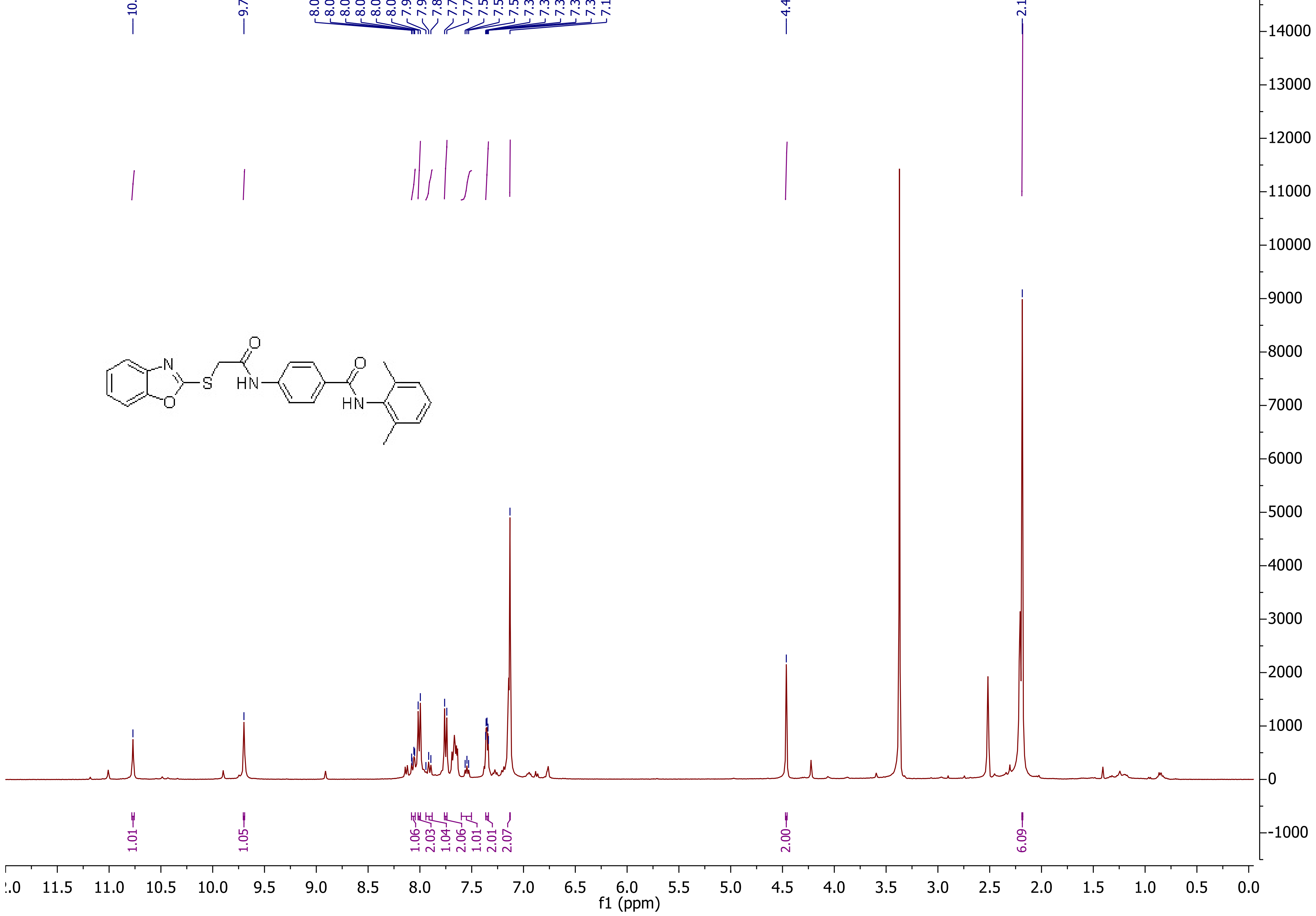
10.77
9.70
8.08 8.08 8.06 8.05 8.02 8.00 7.94 7.92 7.89 7.76 7.74 7.56 7.55 7.53 7.37 7.36 7.35 7.34 7.34 7.13
4.46
2.19
1.01
1.05
1.06 2.03 1.04 2.06 1.01 2.01 2.07
2.00
6.09
N
O
S
HN
O
HN
f1 (ppm)

1H NMR 14g

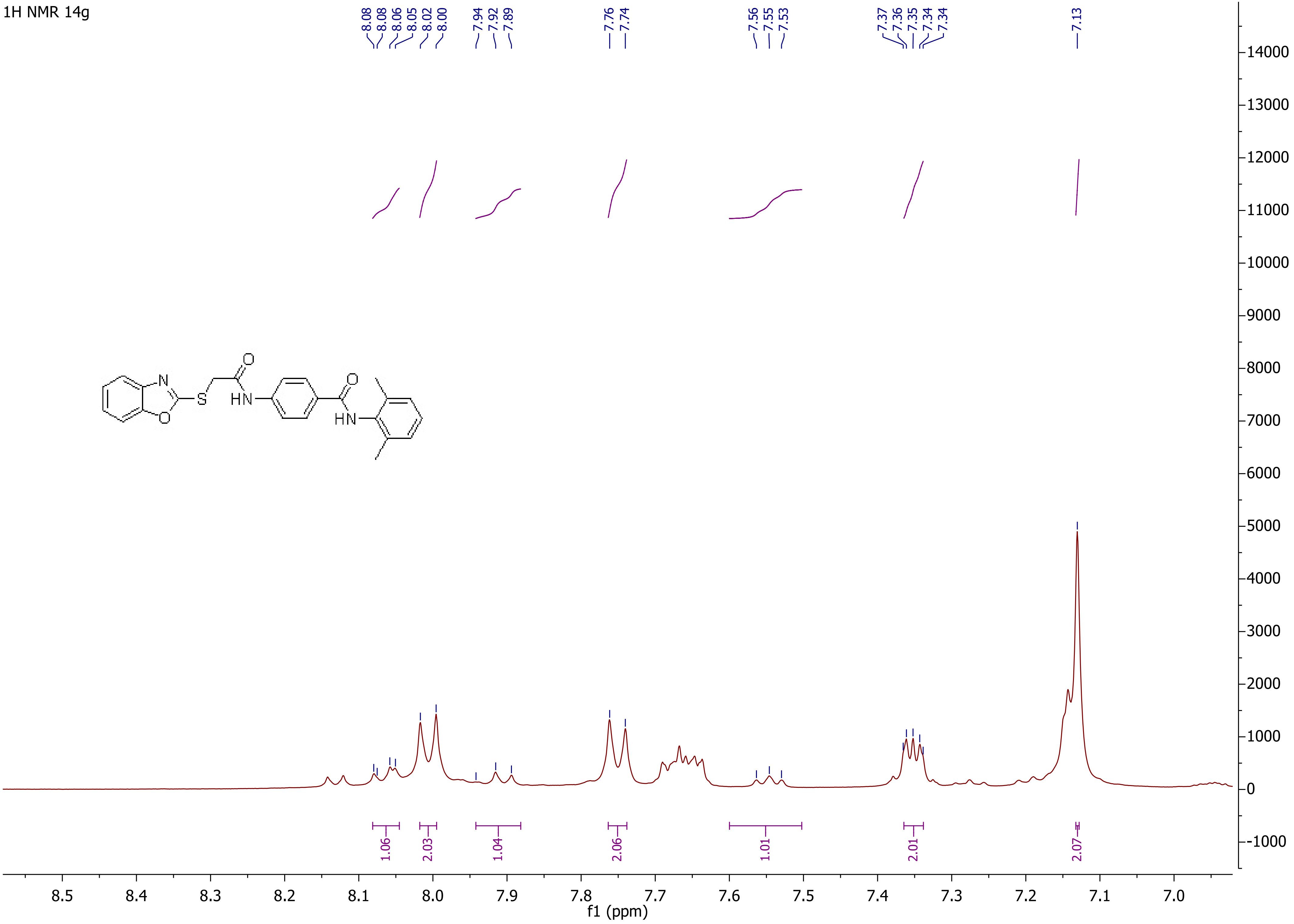

8.08
8.08
8.06
8.05
8.02
8.00
7.94
7.92
7.89
7.76
7.74
7.56
7.55
7.53
7.37
7.36
7.35
7.34
7.34
7.13
1.06
2.03
1.04
2.06
1.01
2.01
2.07
N
O
S
HN
O
HN
O
14000
13000
12000
11000
10000
9000
8000
7000
6000
5000
4000
3000
2000
1000
0
-1000
8.5
8.4
8.3
8.2
8.1
8.0
7.9
7.8
7.7
7.6
7.5
7.4
7.3
7.2
7.1
7.0
f1 (ppm)

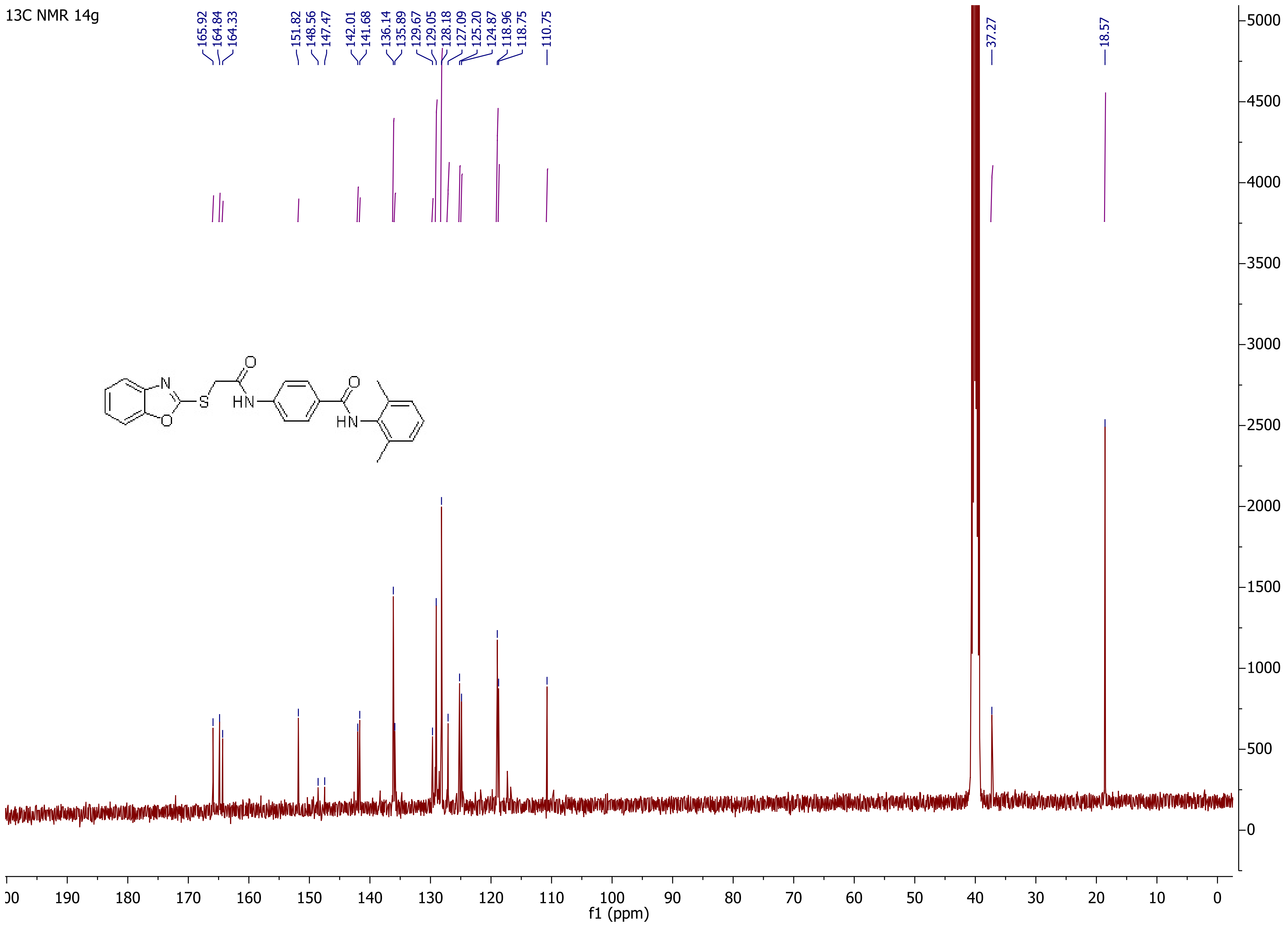
13C NMR 14g
165.92
164.84
164.33
151.82
148.56
147.47
142.01
141.68
136.14
135.89
129.67
129.05
128.18
127.09
125.20
124.87
118.96
118.75
110.75
37.27
18.57
N
O
S
HN
O
HN
O
f1 (ppm)

13C NMR 14g

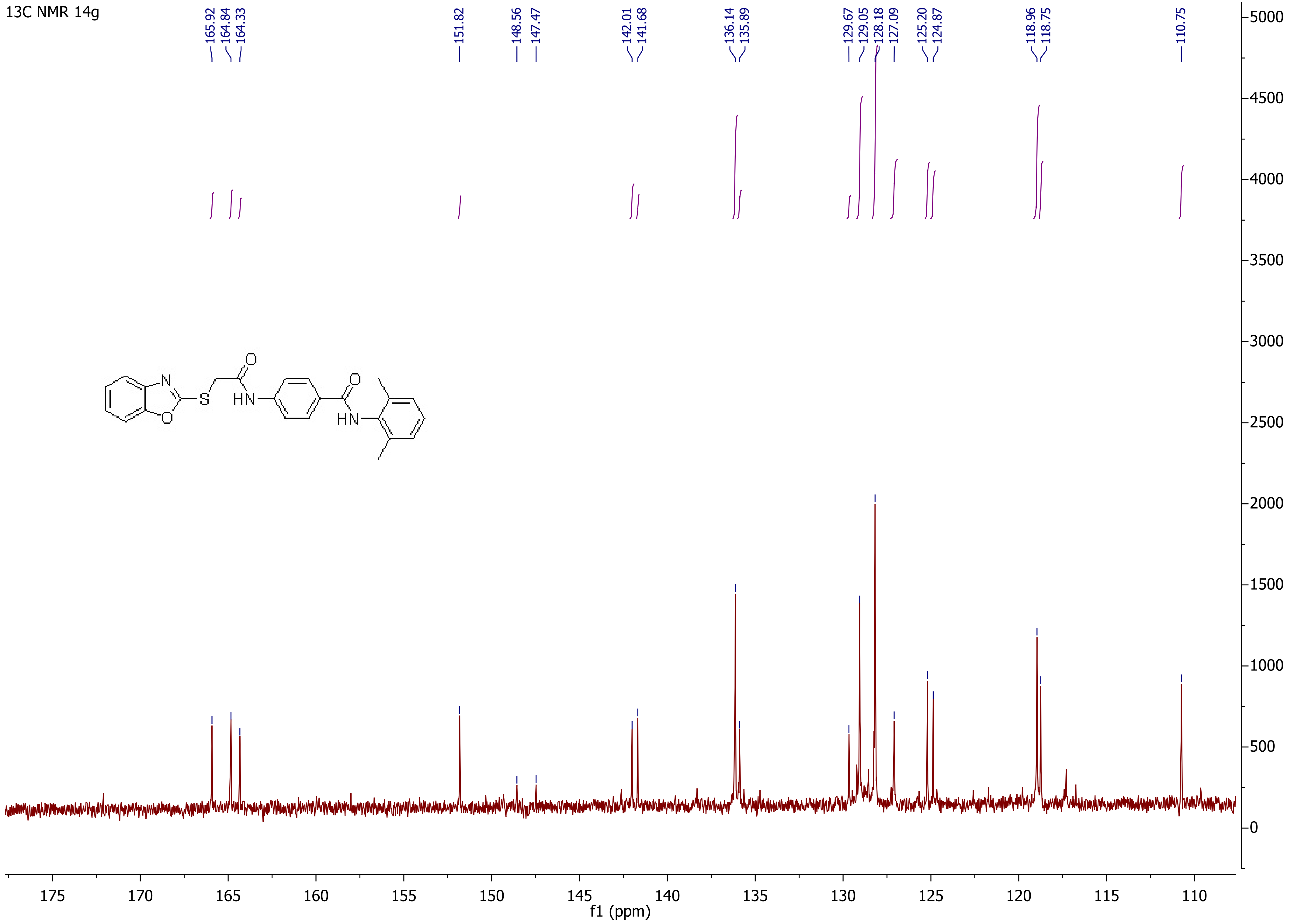

165.92
164.84
164.33
151.82
148.56
147.47
142.01
141.68
136.14
135.89
129.67
129.05
128.18
127.09
125.20
124.87
118.96
118.75
110.75
N
O
S
O
HN
O
HN
f1 (ppm)

IR of comp. 14h

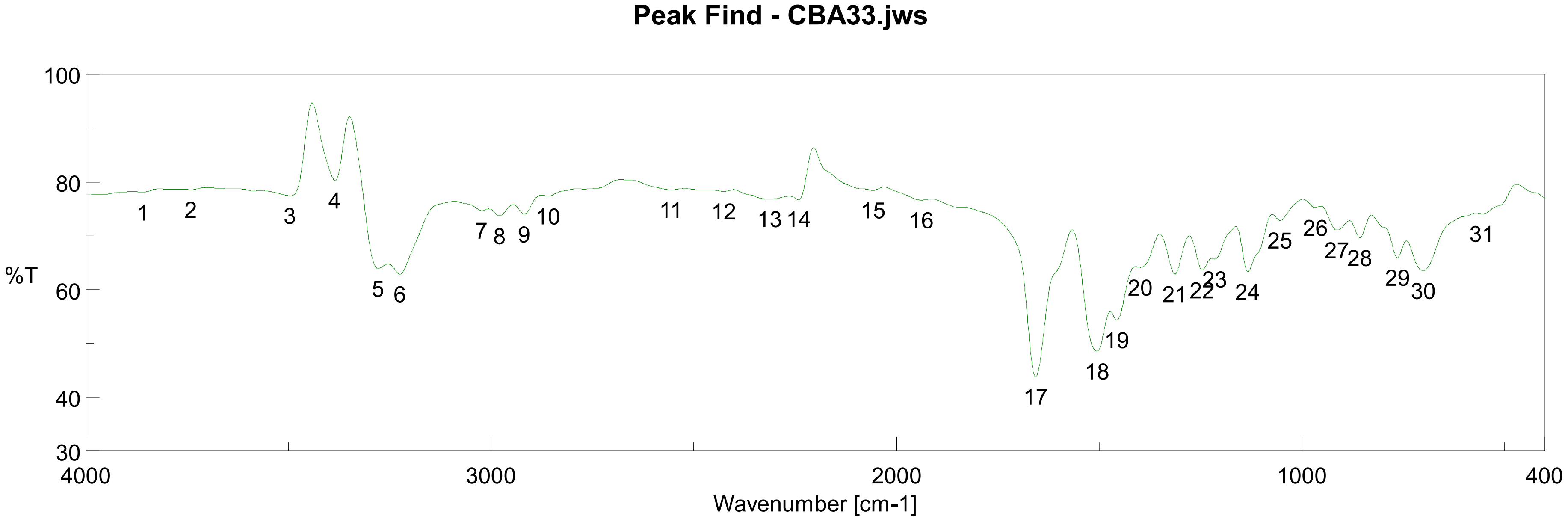

[Result of Peak Picking]

No.	Position	Intensity	No.	Position	Intensity	No.	Position	Intensity	No.	Position	Intensity
1	3858.86	78.068	2	3740.26	78.5135	3	3496.31	77.3856	4	3385.42	80.2257
5	3278.39	63.8685	6	3225.36	62.8238	7	3022.87	74.6589	8	2979.48	73.6601
9	2918.73	73.9839	10	2859.92	77.3582	11	2556.18	78.5082	12	2426.98	78.2011
13	2313.2	76.767	14	2241.84	76.6597	15	2057.67	78.4049	16	1939.07	76.5923
17	1656.55	43.7886	18	1505.17	48.5298	19	1455.99	54.3059	20	1398.14	64.0825
21	1313.29	62.856	22	1245.79	63.5925	23	1213.97	65.6104	24	1132.97	63.2724
25	1052.94	72.8054	26	966.162	75.1901	27	913.129	71.0139	28	856.239	69.5726
29	763.673	65.8767	30	700.034	63.501	31	554.434	74.0343			

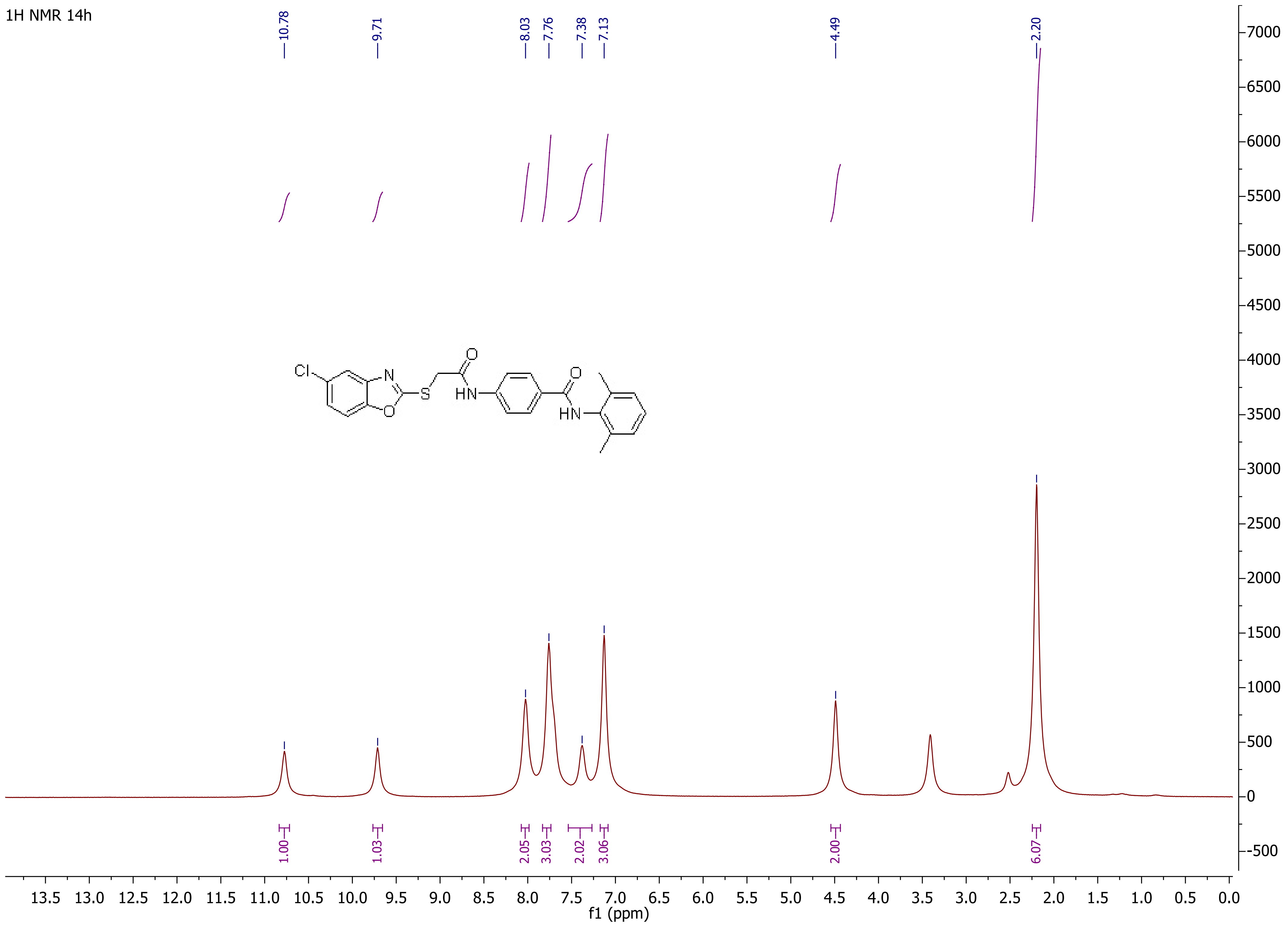

1H NMR 14h
10.78
9.71
8.03
7.76
7.38
7.13
4.49
2.20
1.00
1.03
2.05
3.03
2.02
3.06
2.00
6.07
f1 (ppm)

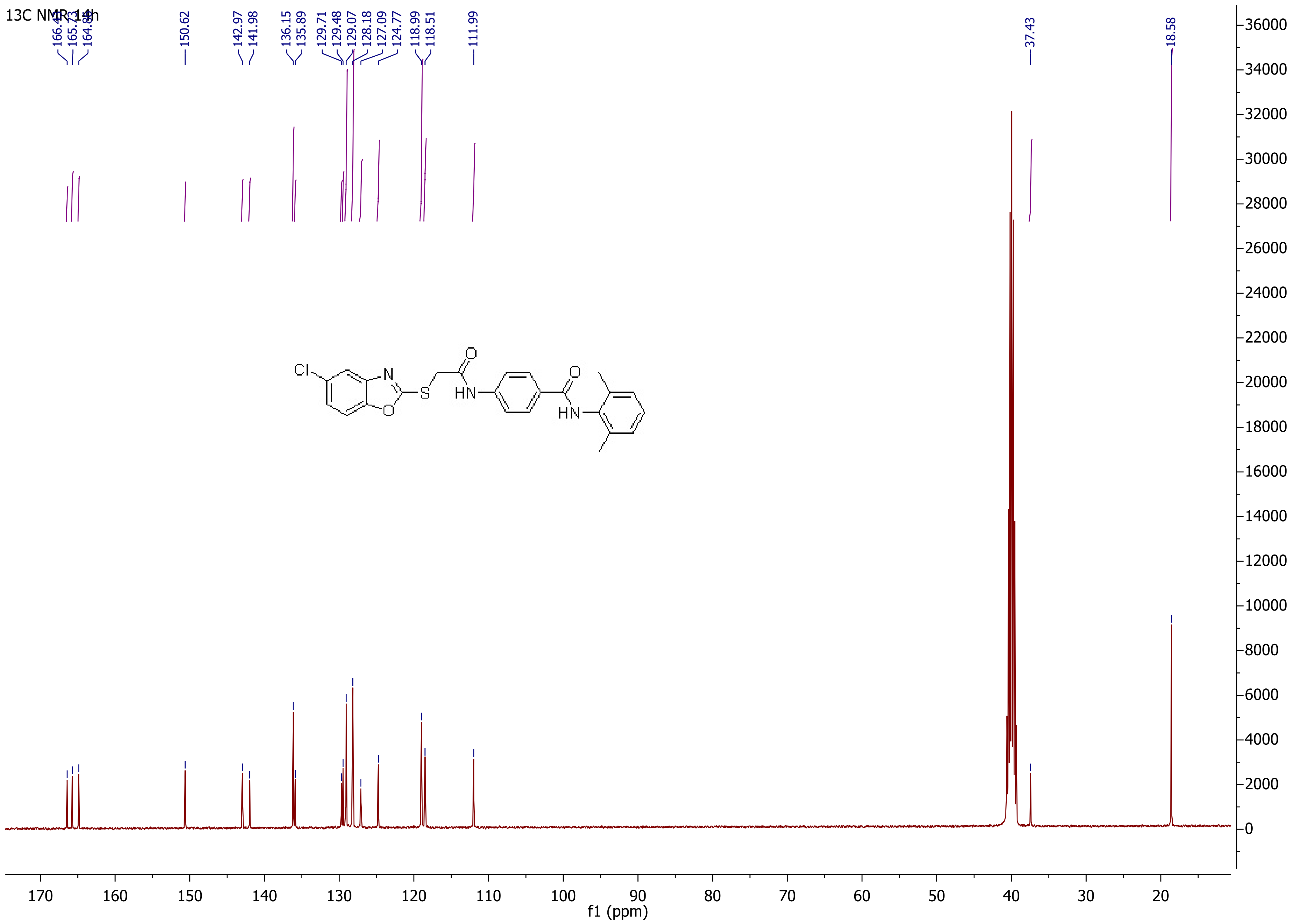

13C NMR 14h
166.41
165.73
164.89
150.62
142.97
141.98
136.15
135.89
129.71
129.48
129.07
128.18
127.09
124.77
118.99
118.51
111.99
37.43
18.58
Cl
N
O
S
O
HN
O
HN
f1 (ppm)

13C NMR 14b

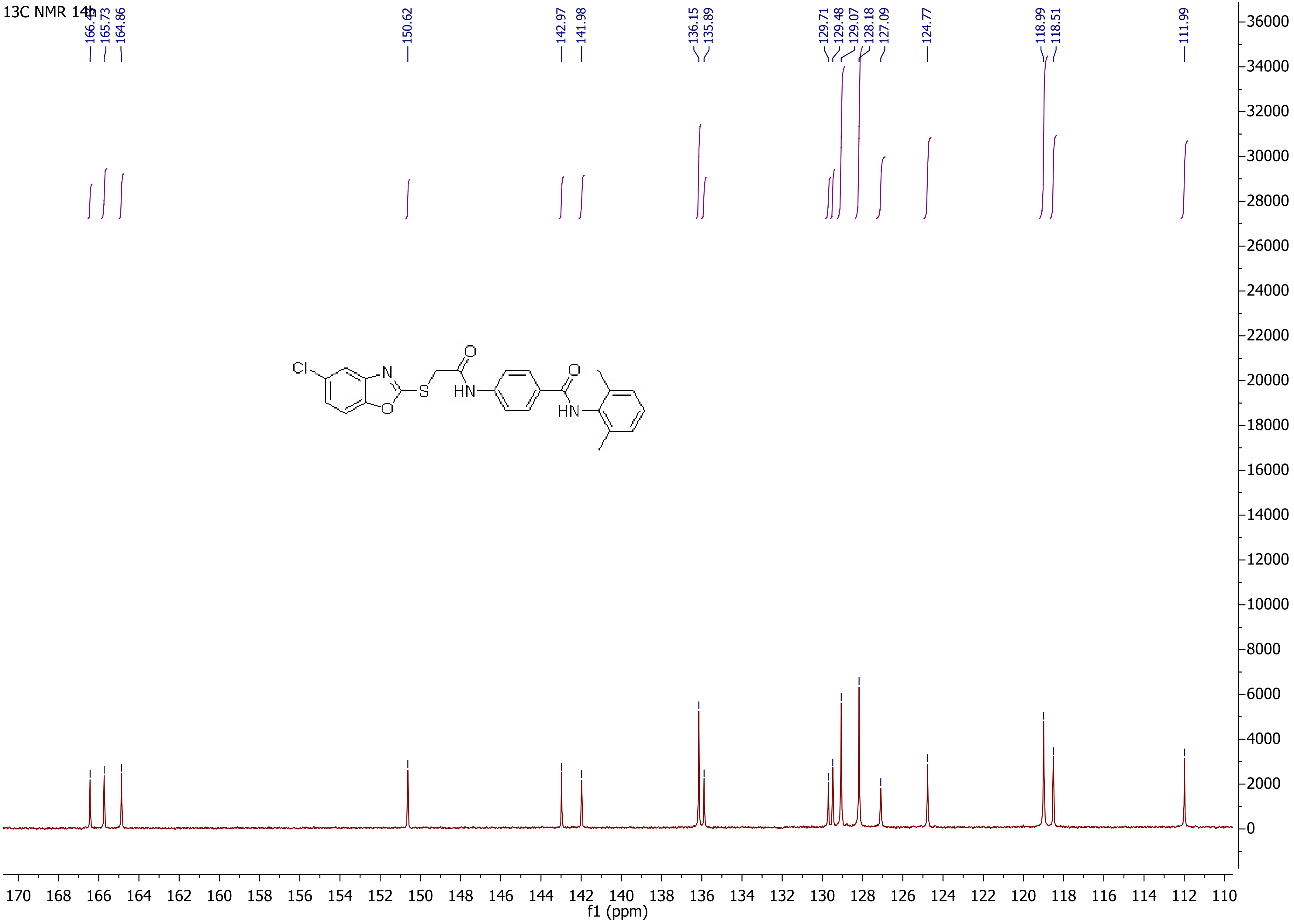

166.43
165.73
164.86
150.62
142.97
141.98
136.15
135.89
129.71
129.48
129.07
128.18
127.09
124.77
118.99
118.51
111.99
Cl
N
O
S
O
HN
O
HN
170 168 166 164 162 160 158 156 154 152 150 148 146 144 142 140 138 136 134 132 130 128 126 124 122 120 118 116 114 112 110
f1 (ppm)
36000 34000 32000 30000 28000 26000 24000 22000 20000 18000 16000 14000 12000 10000 8000 6000 4000 2000 0

IR of comp. 14i

Peak Find - 8.jws

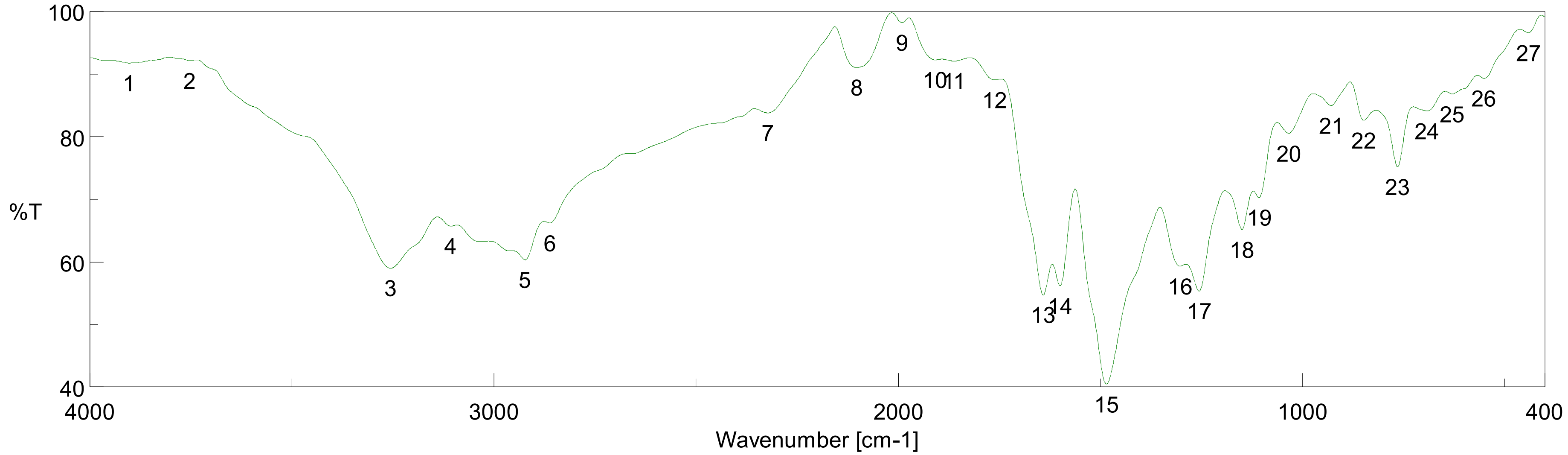

[Result of Peak Picking]

No.	Position	Intensity	No.	Position	Intensity	No.	Position	Intensity	No.	Position	Intensity
1	3903.22	91.7152	2	3752.8	92.0928	3	3256.22	58.9608	4	3106.76	65.6822
5	2923.56	60.3046	6	2861.84	66.2008	7	2322.84	83.7577	8	2102.03	90.9916
9	1990.18	98.2082	10	1909.18	92.2396	11	1861.93	92.0466	12	1761.65	89.0299
13	1641.13	54.6901	14	1599.66	56.1381	15	1484.92	40.4561	16	1302.68	59.2761
17	1255.43	55.3096	18	1149.37	65.1523	19	1106.94	70.2395	20	1033.66	80.4669
21	928.557	84.9174	22	847.561	82.5787	23	764.637	75.1607	24	691.355	84.0872
25	628.68	86.7879	26	550.577	89.2632	27	440.655	96.5689			

1H NMR 14i

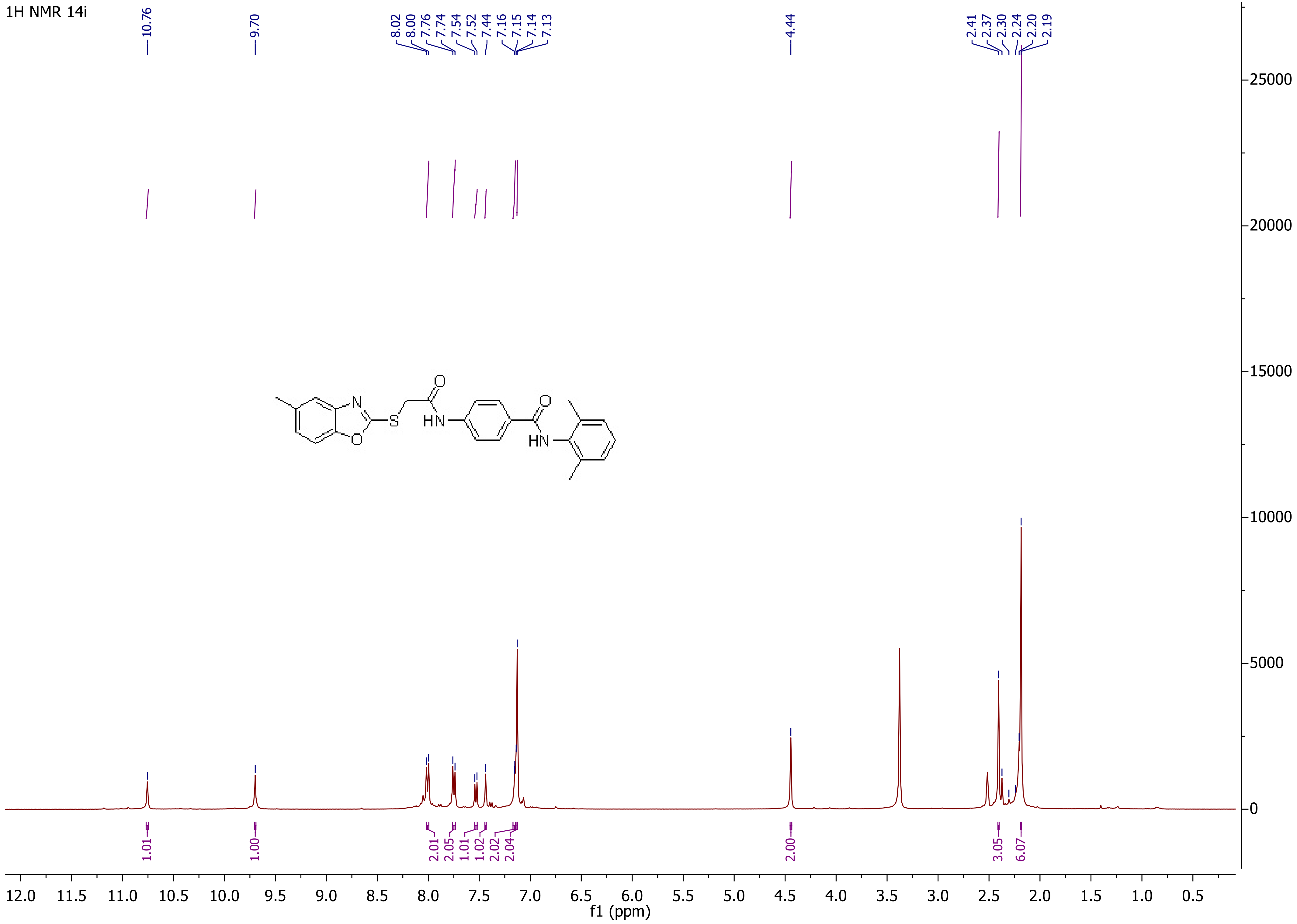

10.76
9.70
8.02
8.00
7.76
7.74
7.54
7.52
7.44
7.16
7.15
7.14
7.13
4.44
2.41
2.37
2.30
2.24
2.20
2.19
N
O
S
HN
O
O
HN
1.01
1.00
2.01
2.05
1.01
1.02
2.02
2.04
2.00
3.05
6.07
25000
20000
15000
10000
5000
0
12.0
11.5
11.0
10.5
10.0
9.5
9.0
8.5
8.0
7.5
7.0
6.5
6.0
5.5
5.0
4.5
4.0
3.5
3.0
2.5
2.0
1.5
1.0
0.5
f1 (ppm)

1H NMR 14i

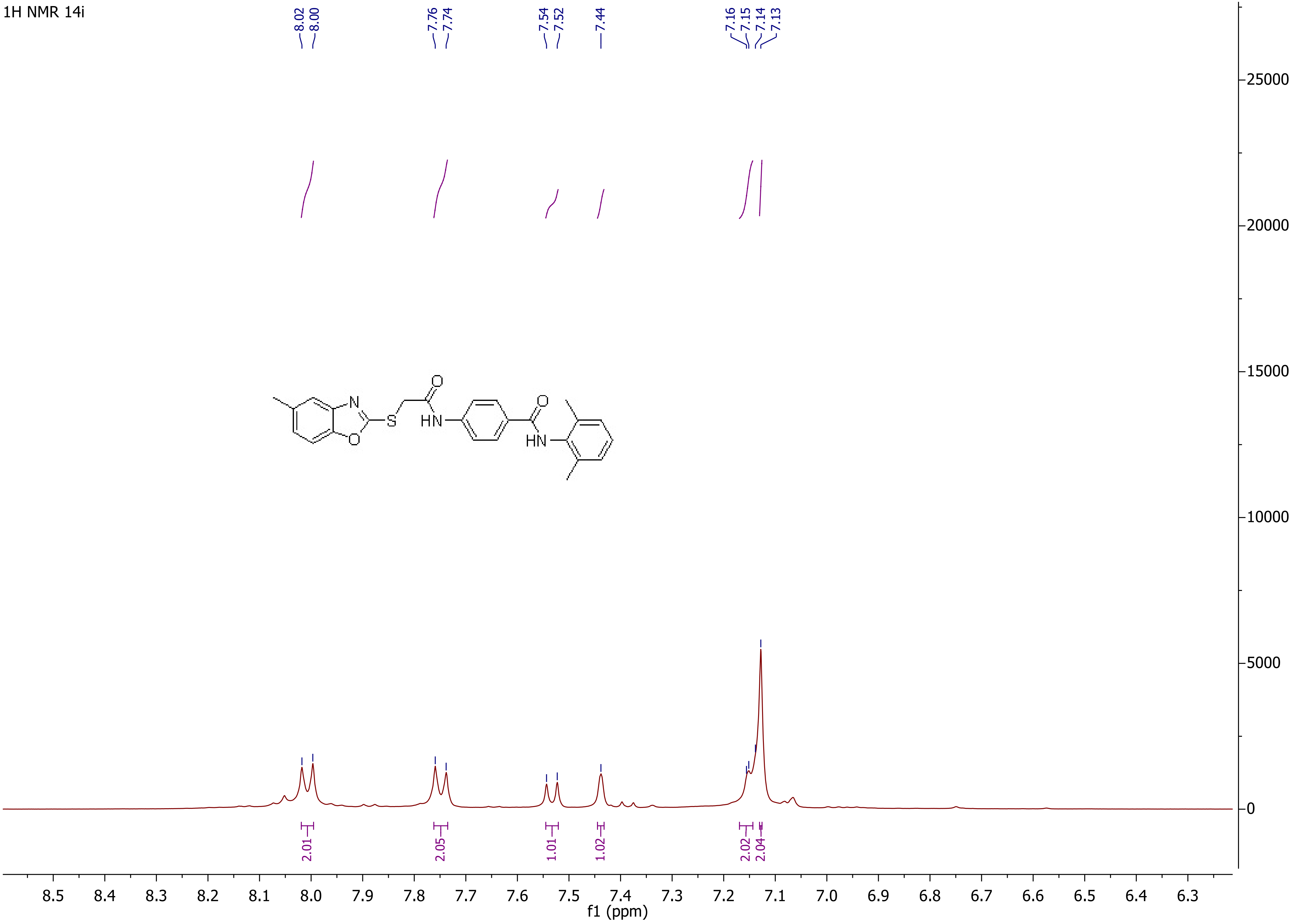

8.02
8.00
7.76
7.74
7.54
7.52
7.44
7.16
7.15
7.14
7.13
25000
20000
15000
10000
5000
0
HN
HN
N
O
S
O
O
2.01
2.05
1.01
1.02
2.02
2.04
8.5
8.4
8.3
8.2
8.1
8.0
7.9
7.8
7.7
7.6
7.5
7.4
7.3
7.2
7.1
7.0
6.9
6.8
6.7
6.6
6.5
6.4
6.3
f1 (ppm)

13C NMR 14i

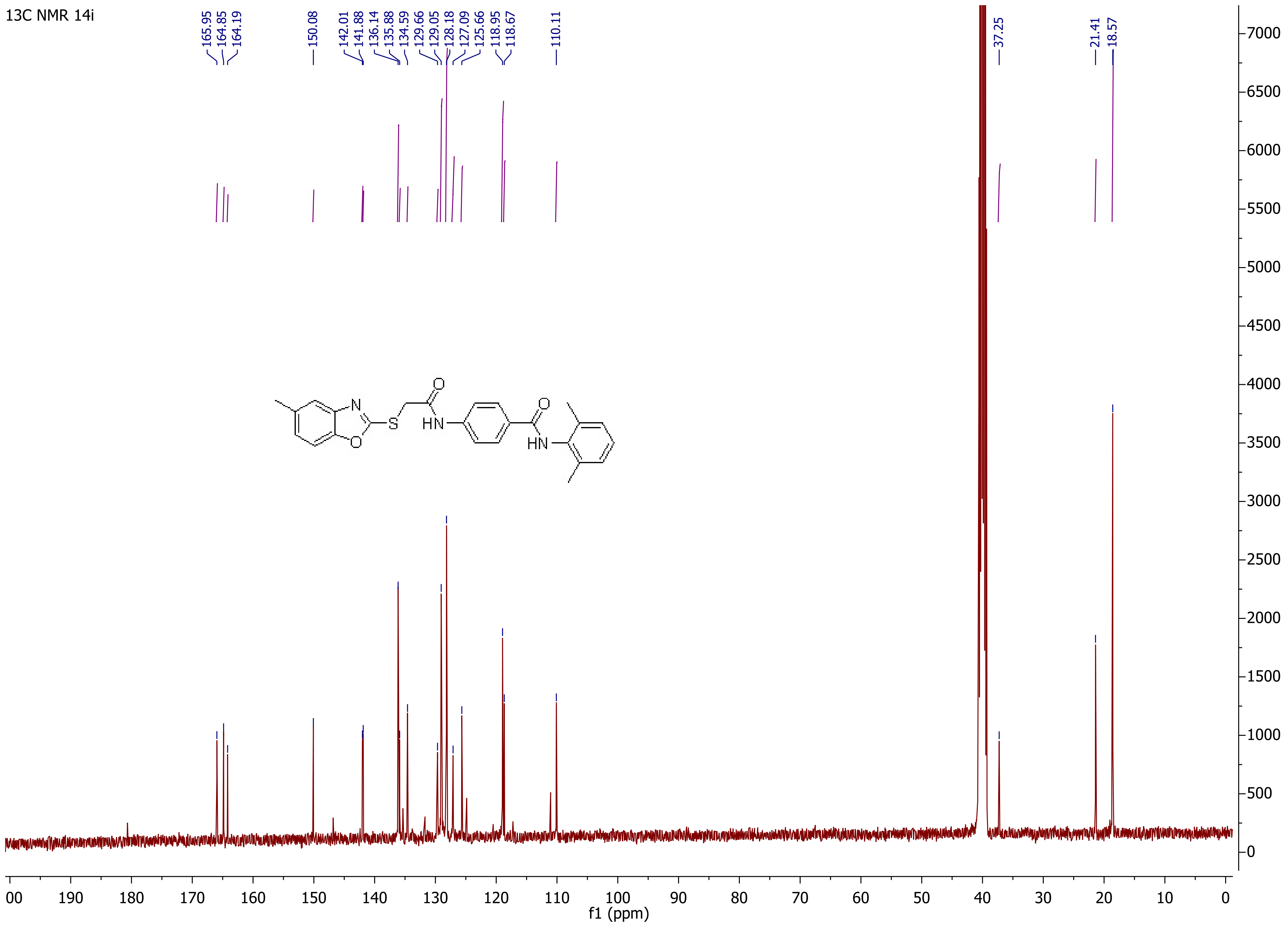

165.95
164.85
164.19
150.08
142.01
141.88
136.14
135.88
134.59
129.66
129.05
128.18
127.09
125.66
118.95
118.67
110.11
37.25
21.41
18.57
N
S
O
HN
O
O
HN
f1 (ppm)

13C NMR 14i

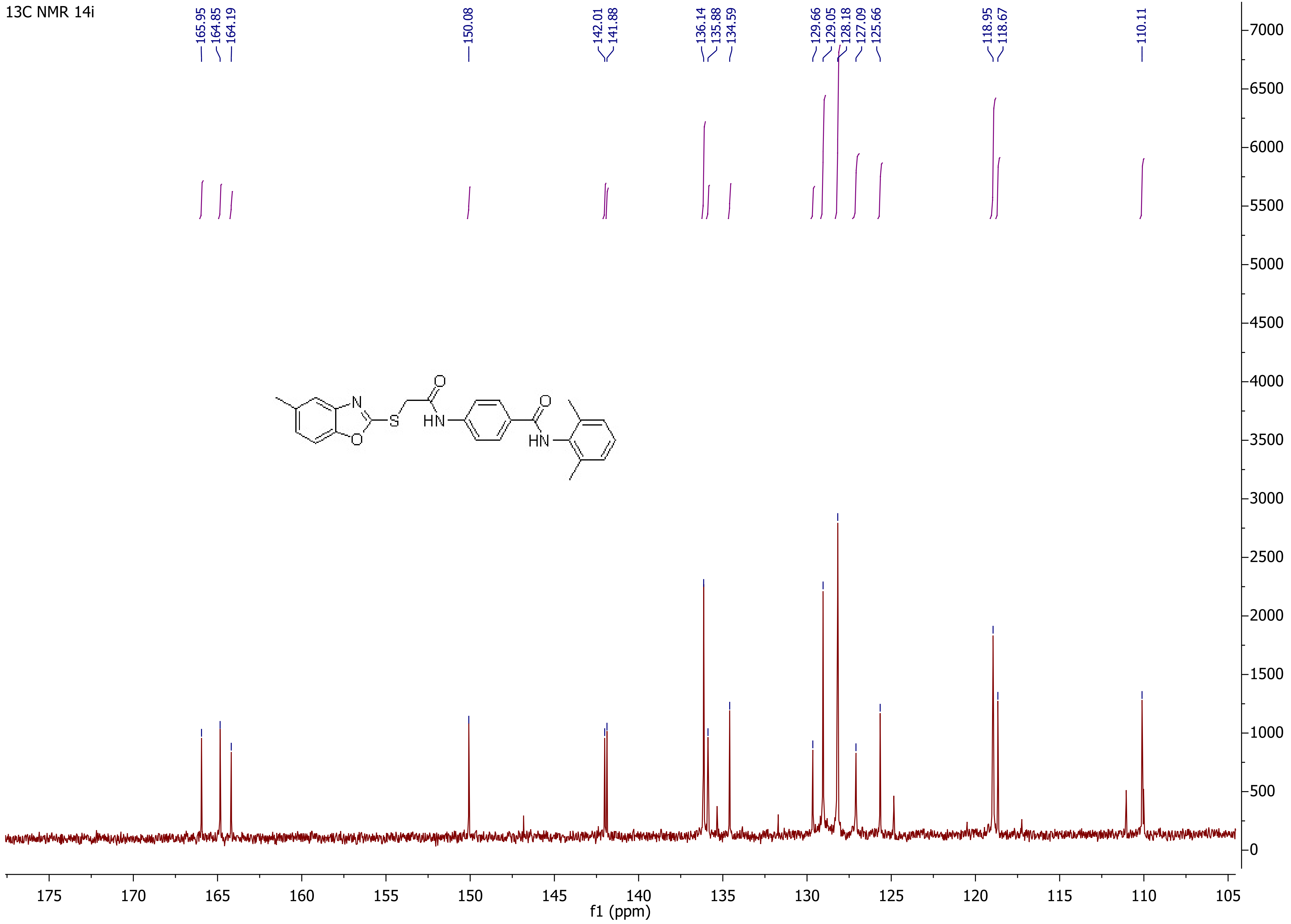
165.95
164.85
164.19
150.08
142.01
141.88
136.14
135.88
134.59
129.66
129.05
128.18
127.09
125.66
118.95
118.67
110.11
7000
6500
6000
5500
5000
4500
4000
3500
3000
2500
2000
1500
1000
500
0
175
170
165
160
155
150
145
140
135
130
125
120
115
110
105
f1 (ppm)
N
O
S
HN
O
O
HN

IR of comp. 14J

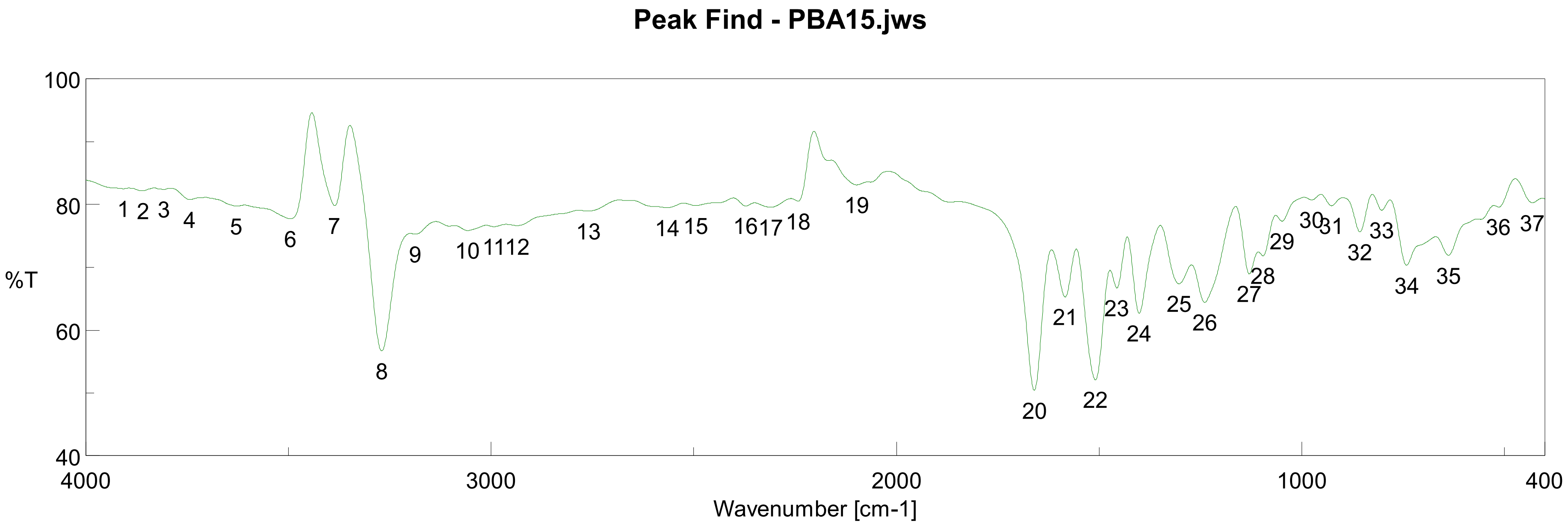

[Result of Peak Picking]

No.	Position	Intensity	No.	Position	Intensity	No.	Position	Intensity	No.	Position	Intensity
1	3907.07	82.4701	2	3858.86	82.1399	3	3807.76	82.3625	4	3743.15	80.774
5	3629.37	79.7227	6	3495.35	77.692	7	3386.39	79.7849	8	3269.72	56.6545
9	3186.79	75.2407	10	3058.55	75.828	11	2992.98	76.4294	12	2936.09	76.6114
13	2760.6	78.9112	14	2565.83	79.457	15	2495.44	79.7983	16	2372.01	79.7345
17	2310.3	79.5238	18	2242.81	80.485	19	2098.17	83.0865	20	1659.45	50.3954
21	1583.27	65.2484	22	1509.03	52.0723	23	1455.99	66.6729	24	1401.03	62.645
25	1302.68	67.3452	26	1239.04	64.3919	27	1129.12	68.9251	28	1095.37	71.8143
29	1048.12	77.316	30	974.84	80.705	31	926.628	79.7695	32	856.239	75.5981
33	802.242	79.0487	34	740.531	70.2878	35	637.358	71.8677	36	513.936	79.564
37	431.012	80.2208									

Mass spec. of comp.14j

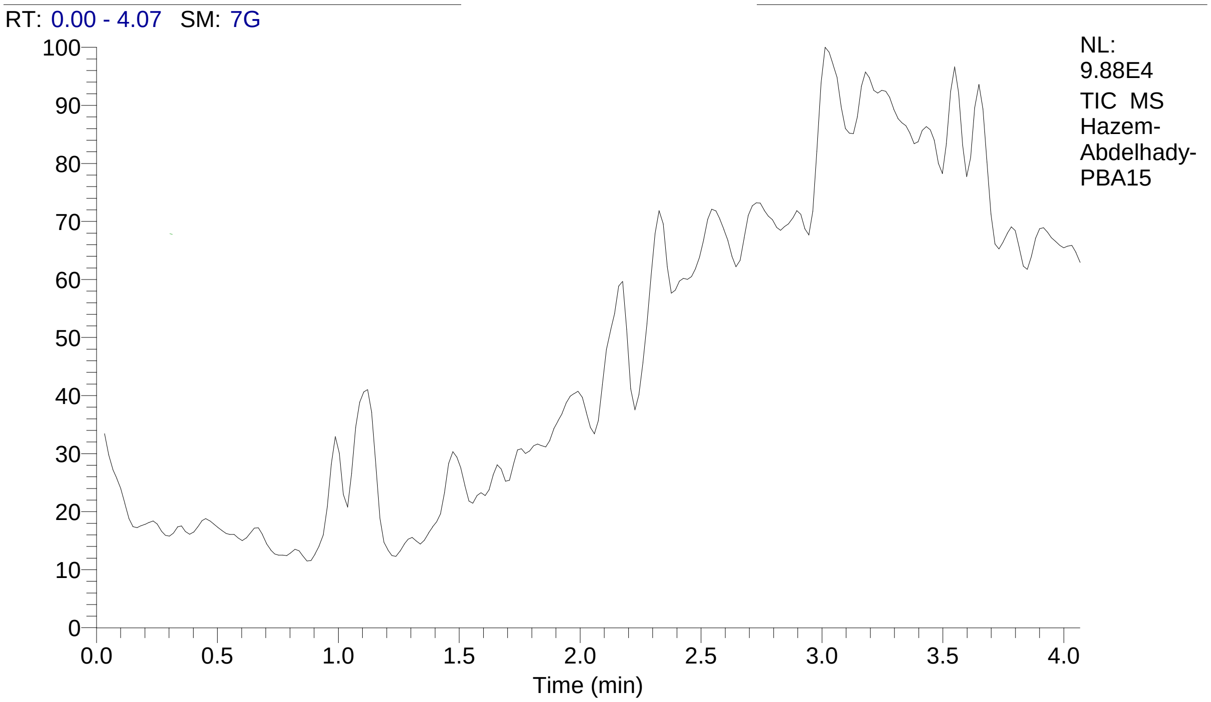

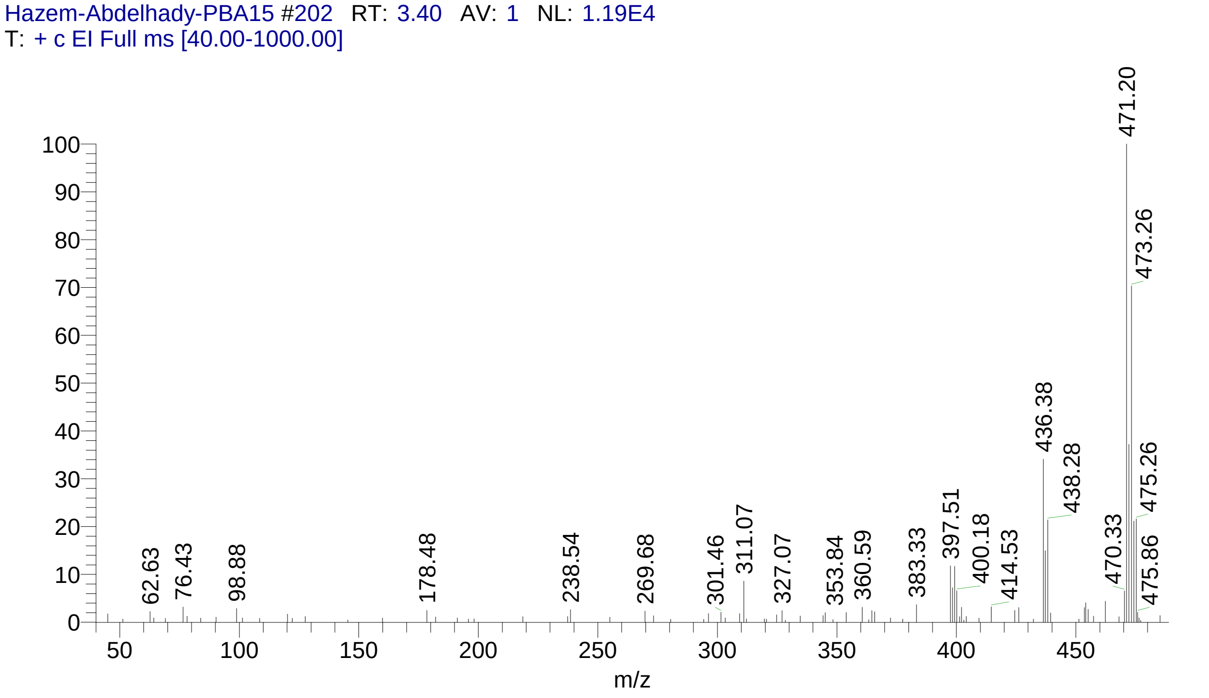

m/z	Intensity	Relative
44.95	211.5	1.77
51.29	77.2	0.65
62.63	269.3	2.26
64.22	108.9	0.91

69.04	98.7	0.83	
76.43	379.2	3.18	
78.16	148.3	1.24	
83.84	101.9	0.85	
90.24	125.7	1.05	
98.88	345.3	2.90	
101.32	110.7	0.93	
108.48	95.2	0.80	
120.11	199.9	1.68	
122.11	97.9	0.82	
127.52	141.2	1.18	
145.45	53.2	0.45	
159.88	103.2	0.87	
178.48	295.7	2.48	
182.09	129.9	1.09	
191.16	108.5	0.91	
195.81	81.5	0.68	
198.15	85.9	0.72	
218.61	136.1	1.14	
237.36	141.1	1.18	
238.54	311.7	2.61	
255.07	128.3	1.08	
269.68	276.6	2.32	
273.27	164.1	1.38	
280.50	75.3	0.63	
294.34	74.1	0.62	
296.27	212.4	1.78	
301.46	248.9	2.09	
303.29	109.1	0.91	
309.33	215.3	1.81	
311.07	1025.5		8.60
312.14	85.9	0.72	
319.65	87.7	0.74	
320.54	78.0	0.65	
324.74	187.9	1.58	
327.07	288.6	2.42	
328.39	48.4	0.41	
334.61	157.6	1.32	
344.23	165.2	1.39	
345.14	240.0	2.01	
348.33	66.9	0.56	
353.84	243.2	2.04	
360.59	374.8	3.14	
363.40	64.1	0.54	
364.68	293.4	2.46	
365.87	263.8	2.21	
372.41	110.3	0.92	
377.59	81.5	0.68	

383.33	435.2	3.65	
397.51	1408.4		11.81
398.40	860.5	7.22	
399.30	1395.2		11.70
400.18	785.5	6.59	
401.35	139.3	1.17	
402.18	373.2	3.13	
403.08	53.1	0.44	
404.13	143.1	1.20	
409.46	102.0	0.86	
414.53	386.9	3.24	
424.43	296.6	2.49	
426.13	368.4	3.09	
432.26	76.9	0.65	
436.38	4069.7		34.13
437.20	1782.8		14.95
438.28	2550.2		21.39
439.46	232.5	1.95	
451.26	81.7	0.69	
453.56	366.8	3.08	
454.13	486.8	4.08	
455.06	322.2	2.70	
457.39	148.0	1.24	
462.29	526.1	4.41	
467.99	136.3	1.14	
470.33	780.1	6.54	
471.20	11925.1		100.00
472.22	4436.0		37.20
473.26	8385.7		70.32
474.27	2522.9		21.16
475.26	2578.5		21.62
475.86	251.1	2.11	
476.46	107.5	0.90	
477.12	50.1	0.42	
485.22	166.9	1.40	

1H NMR 14j

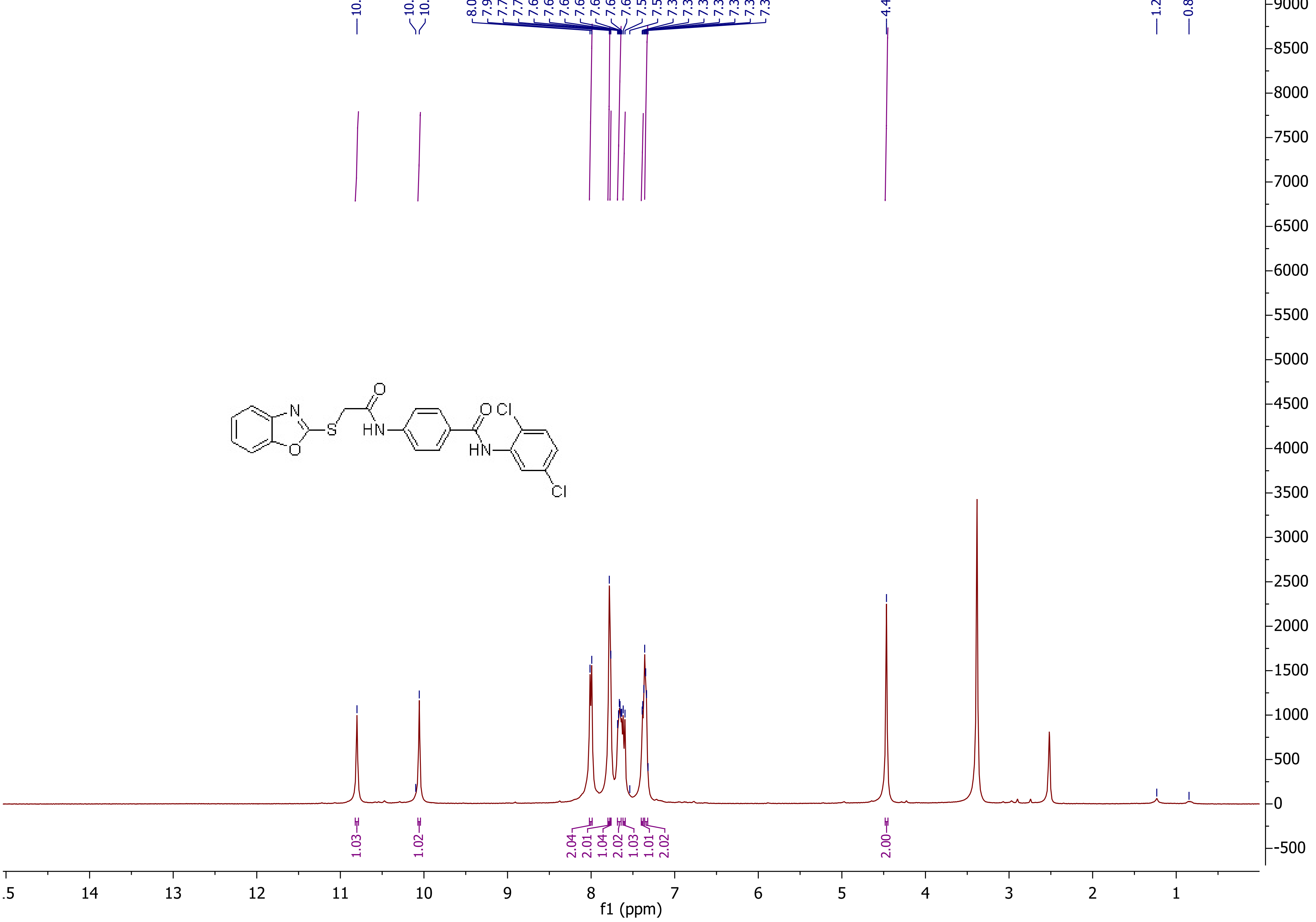

10.80
10.10
10.06
8.01
7.99
7.78
7.76
7.68
7.67
7.66
7.65
7.64
7.63
7.62
7.59
7.54
7.39
7.38
7.37
7.36
7.35
7.34
7.32
4.47
1.23
0.85
1.03
1.02
2.04
2.01
1.04
2.02
1.03
1.01
2.02
2.00
N
O
S
O
HN
O
Cl
HN
Cl
f1 (ppm)

1H NMR 14j

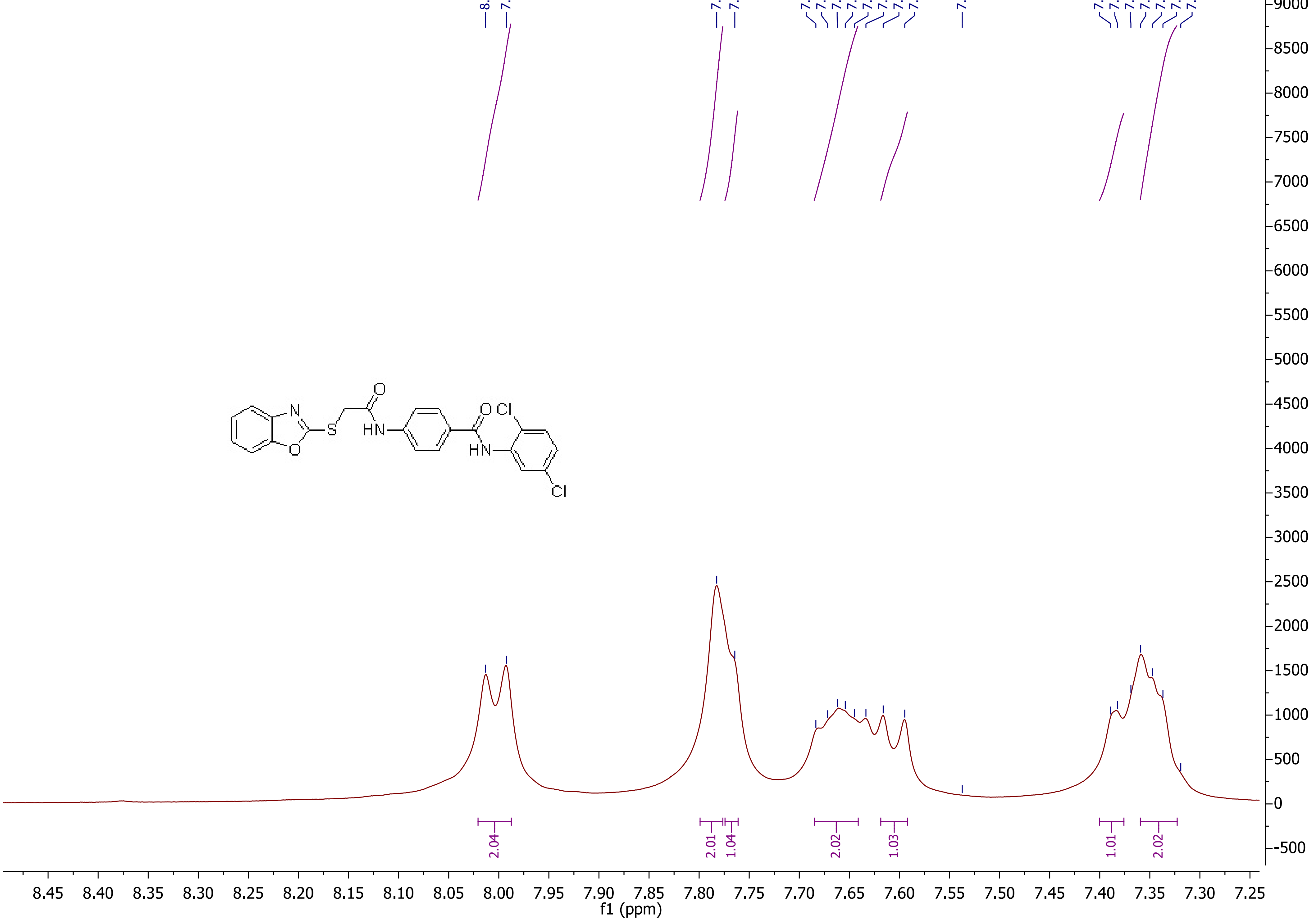
8.01
7.99
7.78
7.76
7.68
7.67
7.66
7.65
7.64
7.63
7.62
7.59
7.54
7.39
7.38
7.37
7.36
7.35
7.34
7.32
2.04
2.01
1.04
2.02
1.03
1.01
2.02
N
O
S
HN
O
O
Cl
HN
Cl
f1 (ppm)

13C NMR 14j

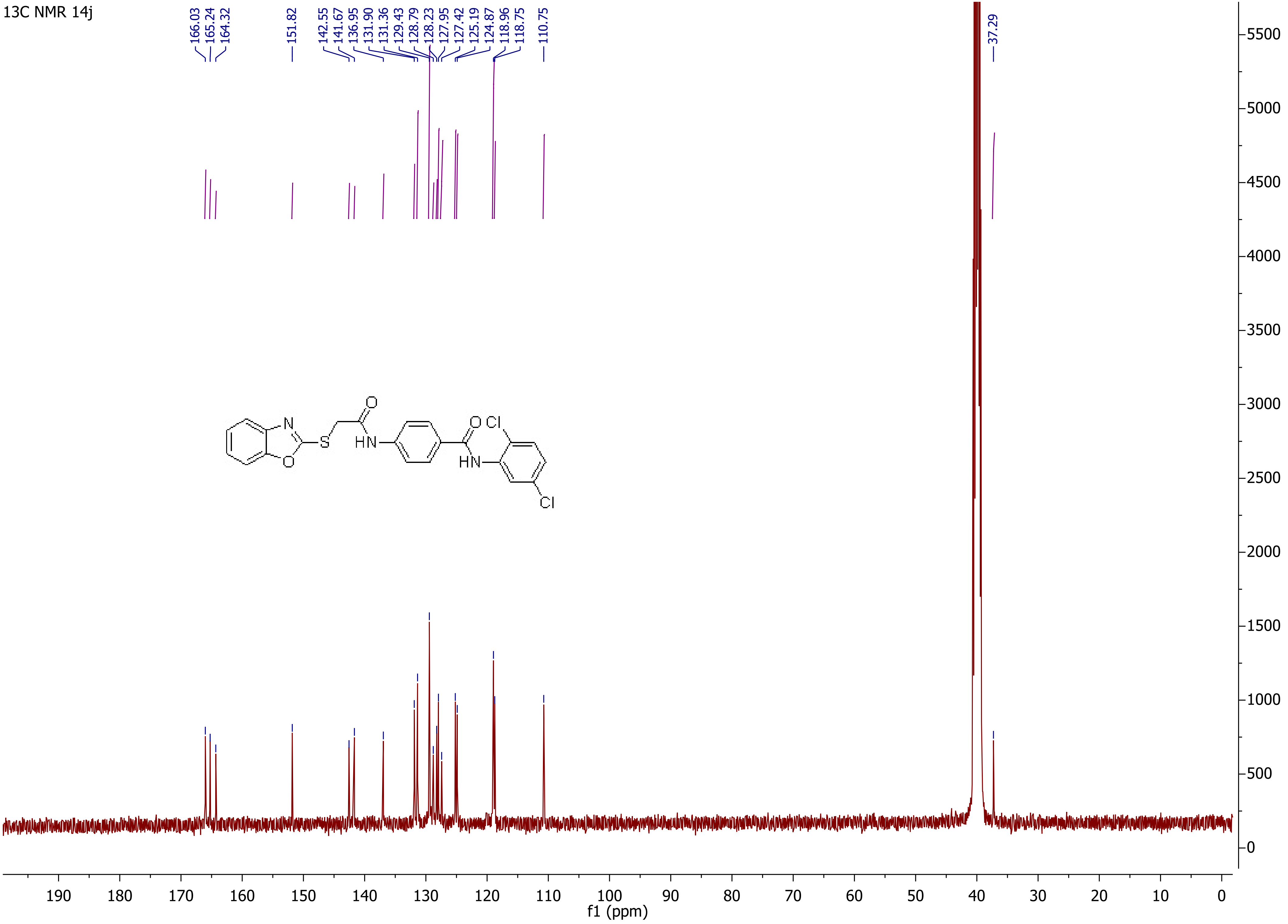

166.03
165.24
164.32
151.82
142.55
141.67
136.95
131.90
131.36
129.43
128.79
128.23
127.95
127.42
125.19
124.87
118.96
118.75
110.75
37.29
5500
5000
4500
4000
3500
3000
2500
2000
1500
1000
500
0
190
180
170
160
150
140
130
120
110
100
90
80
70
60
50
40
30
20
10
0
f1 (ppm)
N
O
S
O
HN
O
Cl
HN
Cl

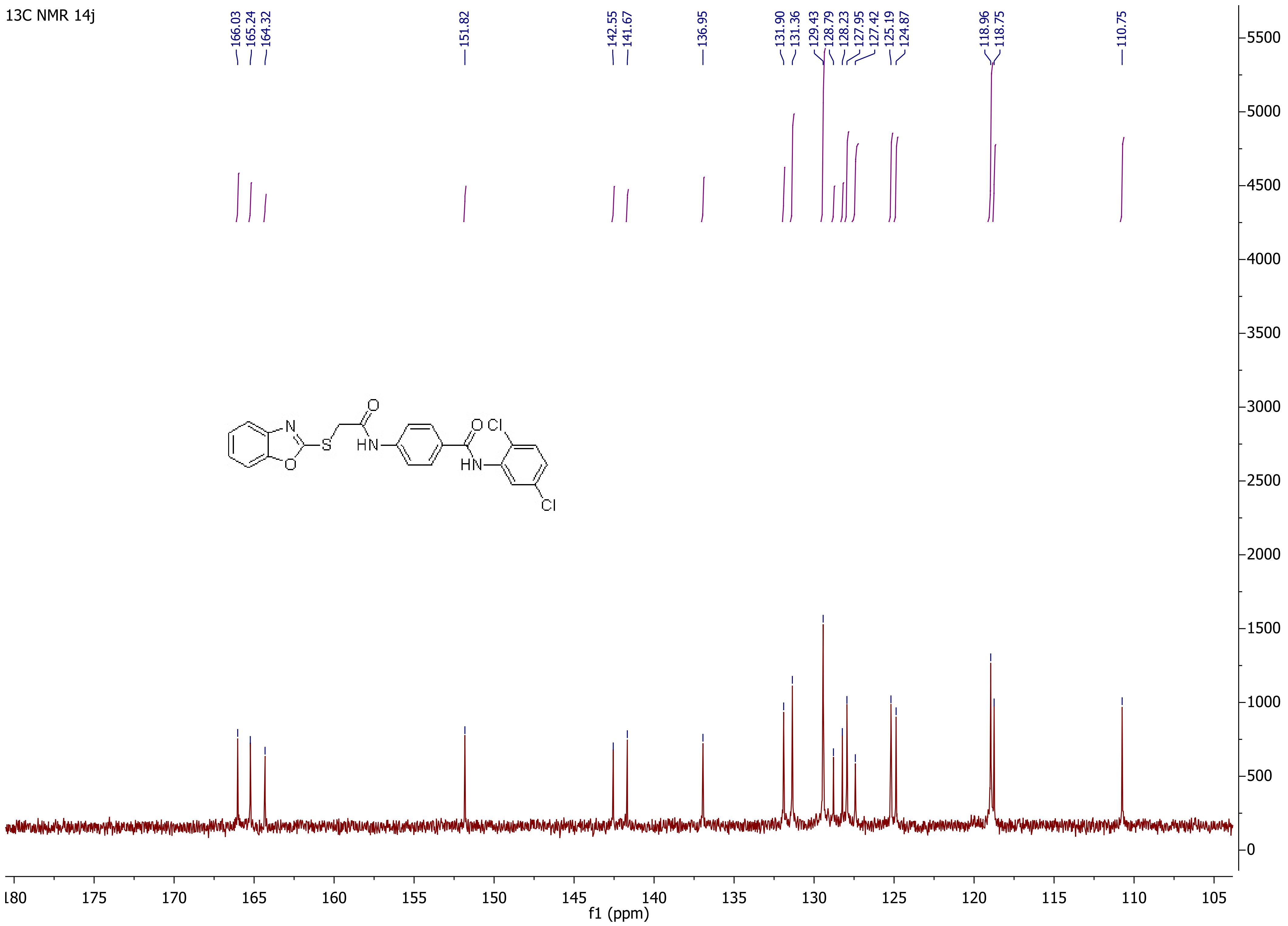
13C NMR 14j
166.03
165.24
164.32
151.82
142.55
141.67
136.95
131.90
131.36
129.43
128.79
128.23
127.95
127.42
125.19
124.87
118.96
118.75
110.75
f1 (ppm)

IR of comp. 14k

Peak Find - CBA15.jws

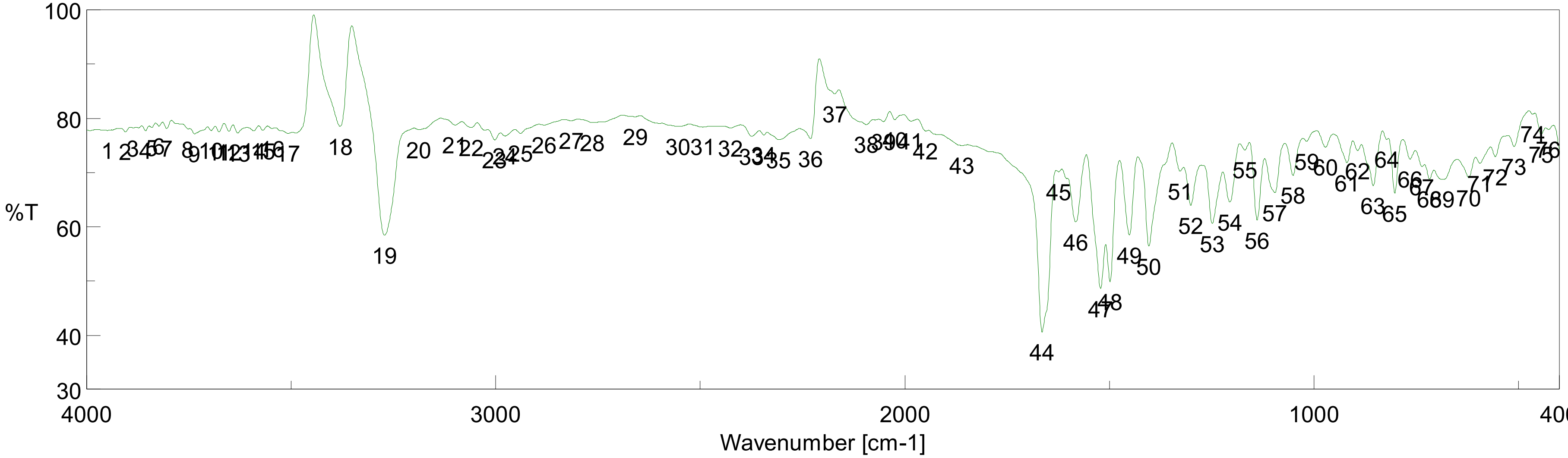

[Result of Peak Picking]

No.	Position	Intensity	No.	Position	Intensity	No.	Position	Intensity	No.	Position	Intensity
1	3949.5	77.7216	2	3905.15	77.4773	3	3886.83	78.165	4	3855.97	77.6827
5	3840.54	78.2688	6	3823.19	78.5352	7	3804.87	78.0876	8	3752.8	77.9817
9	3736.4	77.0834	10	3695.91	77.7181	11	3676.62	77.4715	12	3652.52	77.5617
13	3630.34	77.2422	14	3591.77	77.6548	15	3569.59	77.7305	16	3546.45	78.0706
17	3507.88	77.2003	18	3380.6	78.4567	19	3271.64	58.3975	20	3187.76	77.8377
21	3100.01	78.7278	22	3059.51	78.2716	23	3002.62	75.984	24	2977.55	76.7015
25	2939.95	77.196	26	2881.13	78.7209	27	2815.56	79.5094	28	2764.46	79.1923
29	2659.36	80.2445	30	2554.25	78.4521	31	2494.47	78.3366	32	2426.01	78.1591
33	2374.91	76.6301	34	2345.02	76.8962	35	2308.37	76.0087	36	2230.27	76.2174
37	2171.45	84.4767	38	2094.32	78.7991	39	2051.89	79.3469	40	2023.93	79.6922
41	1985.36	79.4221	42	1948.72	77.6185	43	1860.01	74.9525	44	1664.27	40.5193
45	1623.77	70.0815	46	1582.31	60.8265	47	1521.56	48.5421	48	1498.42	49.8024
49	1451.17	58.3951	50	1402.96	56.3467	51	1326.79	70.2036	52	1300.75	63.8801
53	1247.72	60.5518	54	1205.29	64.5257	55	1168.65	74.1331	56	1138.76	61.1721
57	1095.37	66.222	58	1051.01	69.4336	59	1017.27	75.7245	60	970.983	74.6394
61	918.914	71.7573	62	892.88	73.9823	63	854.311	67.5321	64	821.527	76.0529
65	802.242	66.1468	66	764.637	72.4304	67	735.71	71.0532	68	717.39	68.7723

[Result of Peak Picking]

No.	Position	Intensity	No.	Position	Intensity	No.	Position	Intensity	No.	Position	Intensity
69	685.57	68.6929	70	620.966	69.0008	71	593.968	71.6449	72	556.363	72.8842
73	510.08	74.7491	74	464.761	80.7362	75	444.512	76.9737	76	426.191	77.9106

Mass spec. of comp.14k

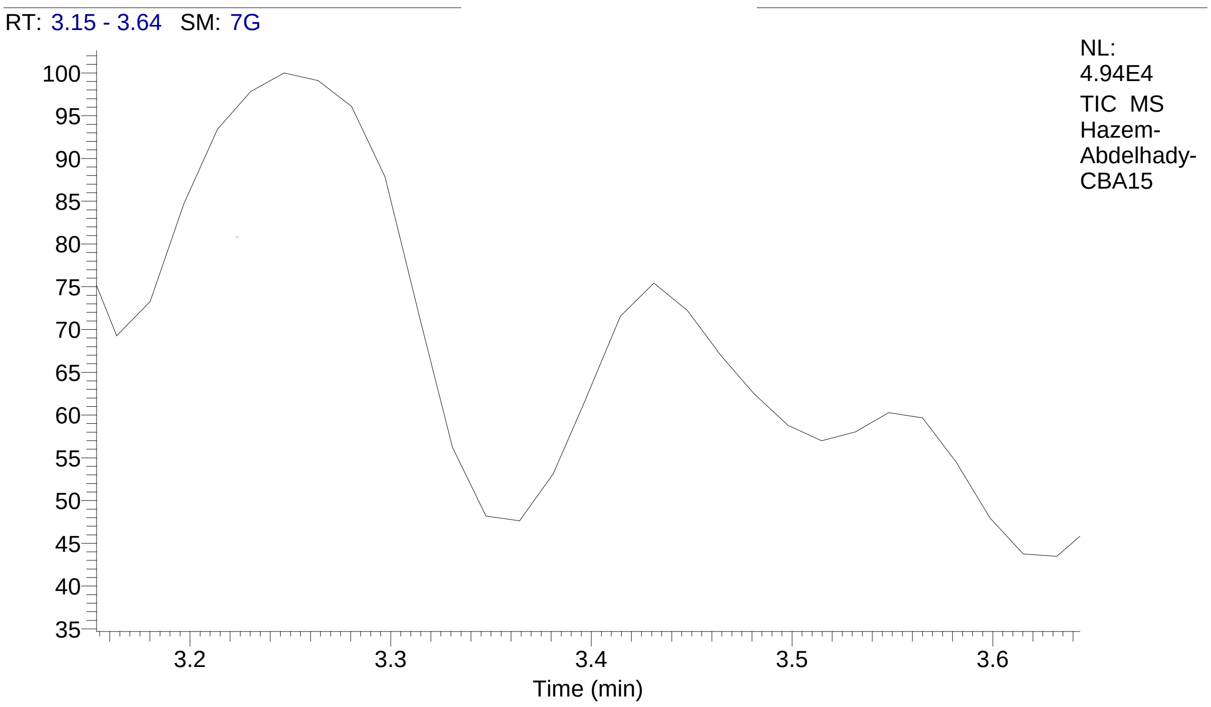

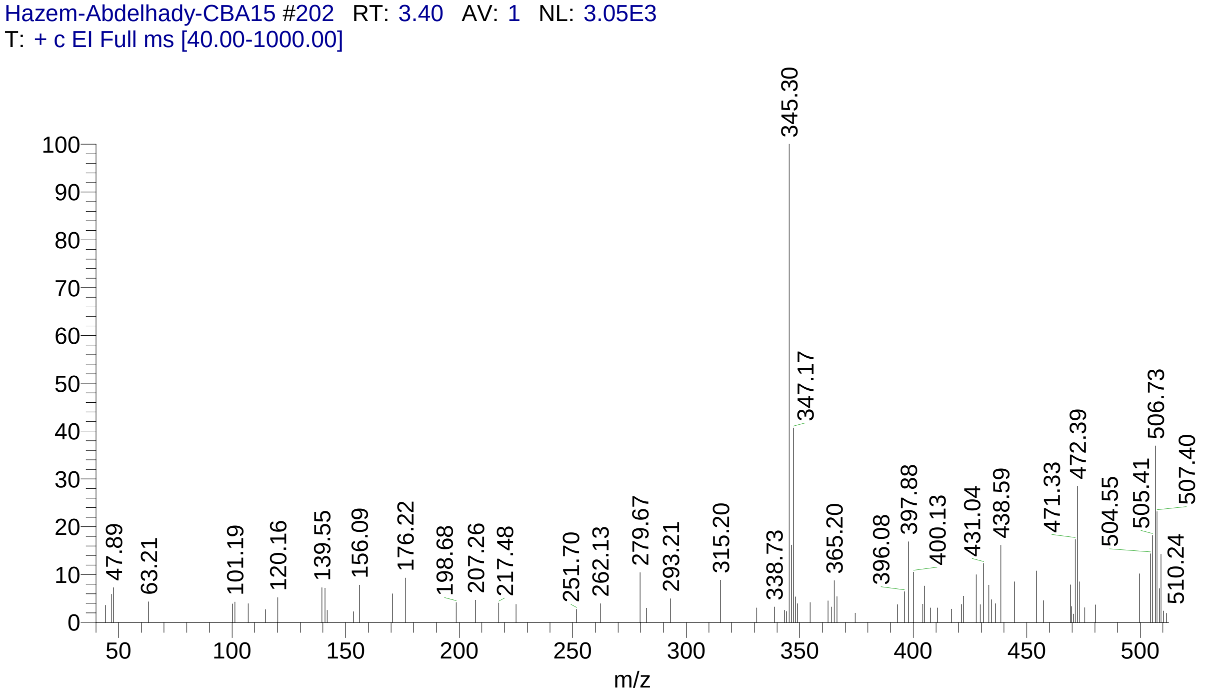

m/z	Intensity	Relative
44.29	108.4	3.55
47.02	179.2	5.87

47.89	222.7	7.29	
63.21	132.3	4.33	
100.08	117.5	3.85	
101.19	130.0	4.26	
107.05	119.3	3.91	
114.74	82.5	2.70	
120.16	158.1	5.18	
139.55	222.4	7.28	
140.89	219.3	7.18	
141.81	76.9	2.52	
153.34	67.9	2.22	
156.09	237.7	7.78	
170.52	182.9	5.99	
176.22	283.6	9.28	
198.68	127.1	4.16	
207.26	142.1	4.65	
217.48	123.6	4.05	
225.02	114.7	3.75	
251.70	84.1	2.75	
262.13	119.7	3.92	
279.67	318.4	10.42	
282.52	91.2	2.99	
293.21	151.5	4.96	
315.20	270.1	8.84	
331.07	92.8	3.04	
338.73	99.2	3.25	
343.27	78.1	2.56	
344.19	70.1	2.30	
345.30	3054.2		100.00
346.35	494.9	16.20	
347.17	1242.7		40.69
348.07	163.7	5.36	
349.08	119.7	3.92	
354.60	126.7	4.15	
362.47	137.5	4.50	
364.10	98.1	3.21	
365.20	266.5	8.73	
366.41	164.5	5.39	
374.40	59.6	1.95	
392.97	113.7	3.72	
396.08	196.9	6.45	
397.88	516.0	16.89	
400.13	320.6	10.50	
404.20	116.5	3.82	
405.06	232.8	7.62	
407.53	92.1	3.02	
410.64	92.3	3.02	
416.92	84.9	2.78	

421.15	114.8	3.76	
422.04	167.9	5.50	
427.65	305.0	9.99	
429.41	113.7	3.72	
431.04	376.4	12.32	
433.33	238.3	7.80	
434.33	145.6	4.77	
436.20	119.6	3.92	
438.59	493.0	16.14	
444.53	259.7	8.50	
454.21	328.1	10.74	
457.40	139.1	4.55	
469.27	240.4	7.87	
469.79	101.7	3.33	
470.44	54.0	1.77	
471.33	530.2	17.36	
472.39	870.2	28.49	
473.07	259.8	8.51	
475.61	93.7	3.07	
480.26	111.6	3.65	
499.67	310.0	10.15	
504.55	439.2	14.38	
505.41	556.2	18.21	
506.73	1127.8		36.93
507.40	707.6	23.17	
508.56	216.4	7.08	
509.17	436.1	14.28	
510.24	72.9	2.39	
511.57	58.4	1.91	

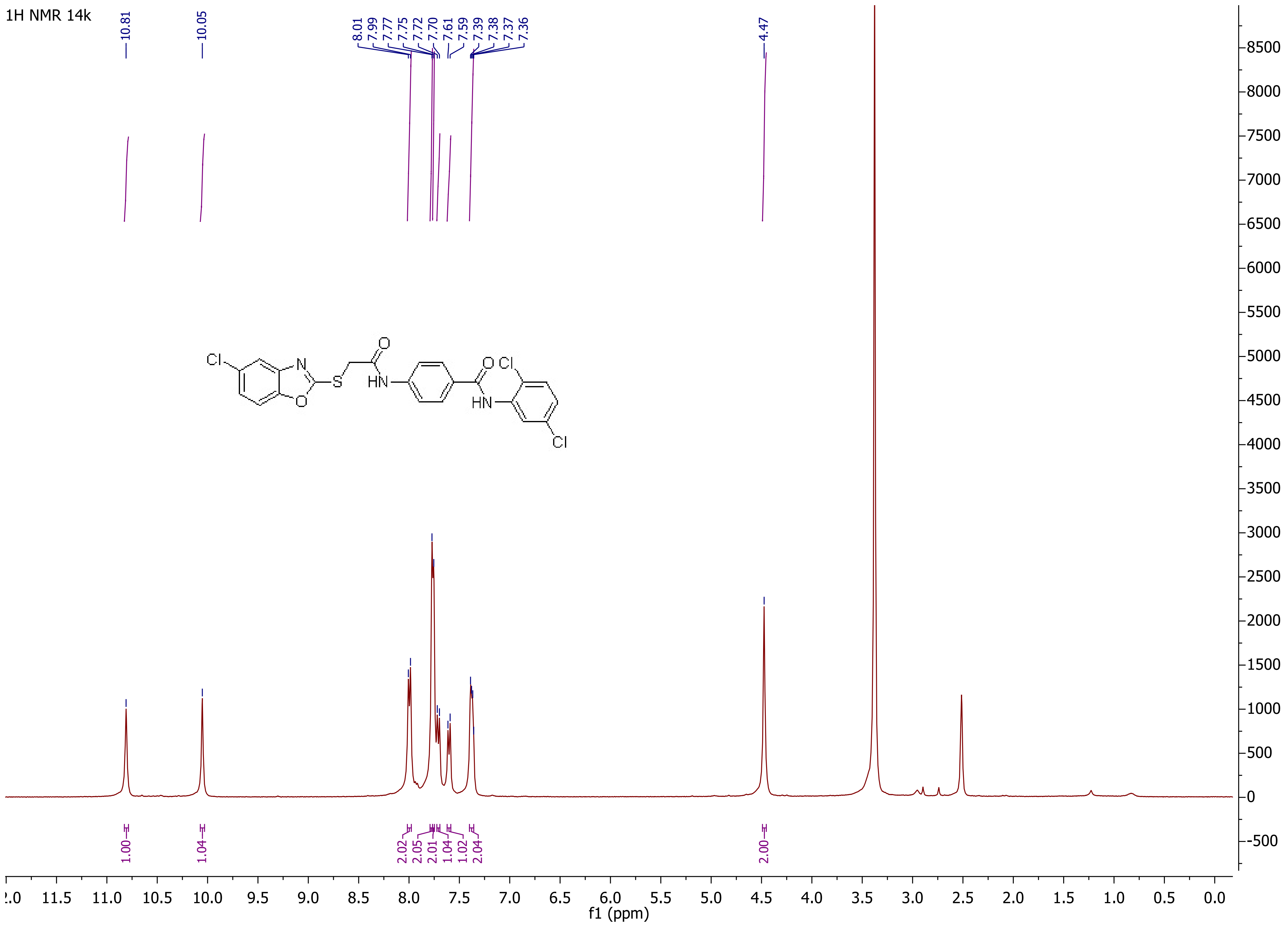

1H NMR 14k
10.81
10.05
8.01
7.99
7.77
7.75
7.72
7.70
7.61
7.59
7.39
7.38
7.37
7.36
4.47
1.00
1.04
2.02
2.05
2.01
1.04
1.02
2.04
2.00
f1 (ppm)

1H NMR 14k

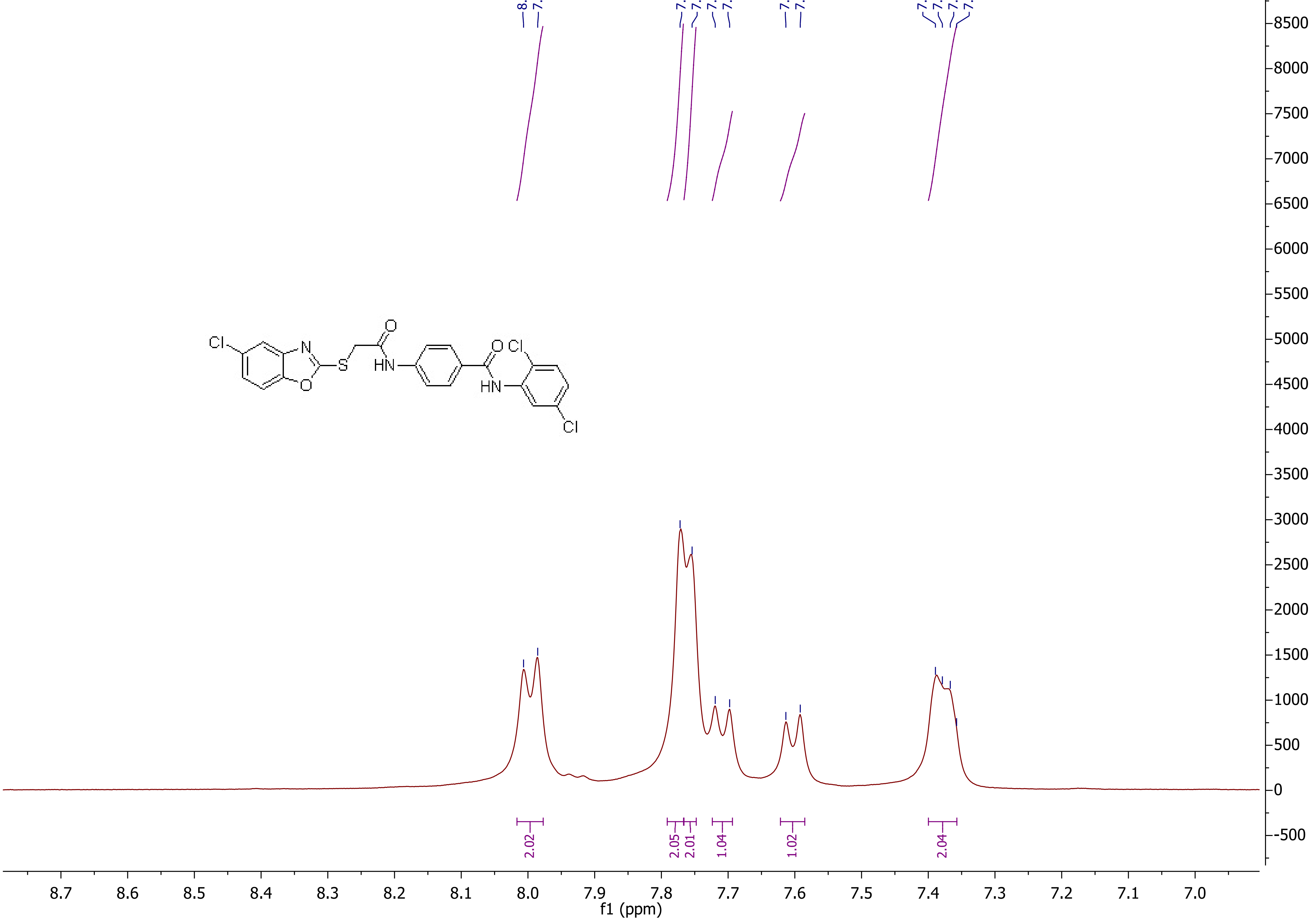

8.01
7.99
7.77
7.75
7.72
7.70
7.61
7.59
7.39
7.38
7.37
7.36
Cl
N
S
O
HN
O
O
Cl
HN
Cl
2.02
2.05
2.01
1.04
1.02
2.04
8.7
8.6
8.5
8.4
8.3
8.2
8.1
8.0
7.9
7.8
7.7
7.6
7.5
7.4
7.3
7.2
7.1
7.0
f1 (ppm)
8500
8000
7500
7000
6500
6000
5500
5000
4500
4000
3500
3000
2500
2000
1500
1000
500
0
-500

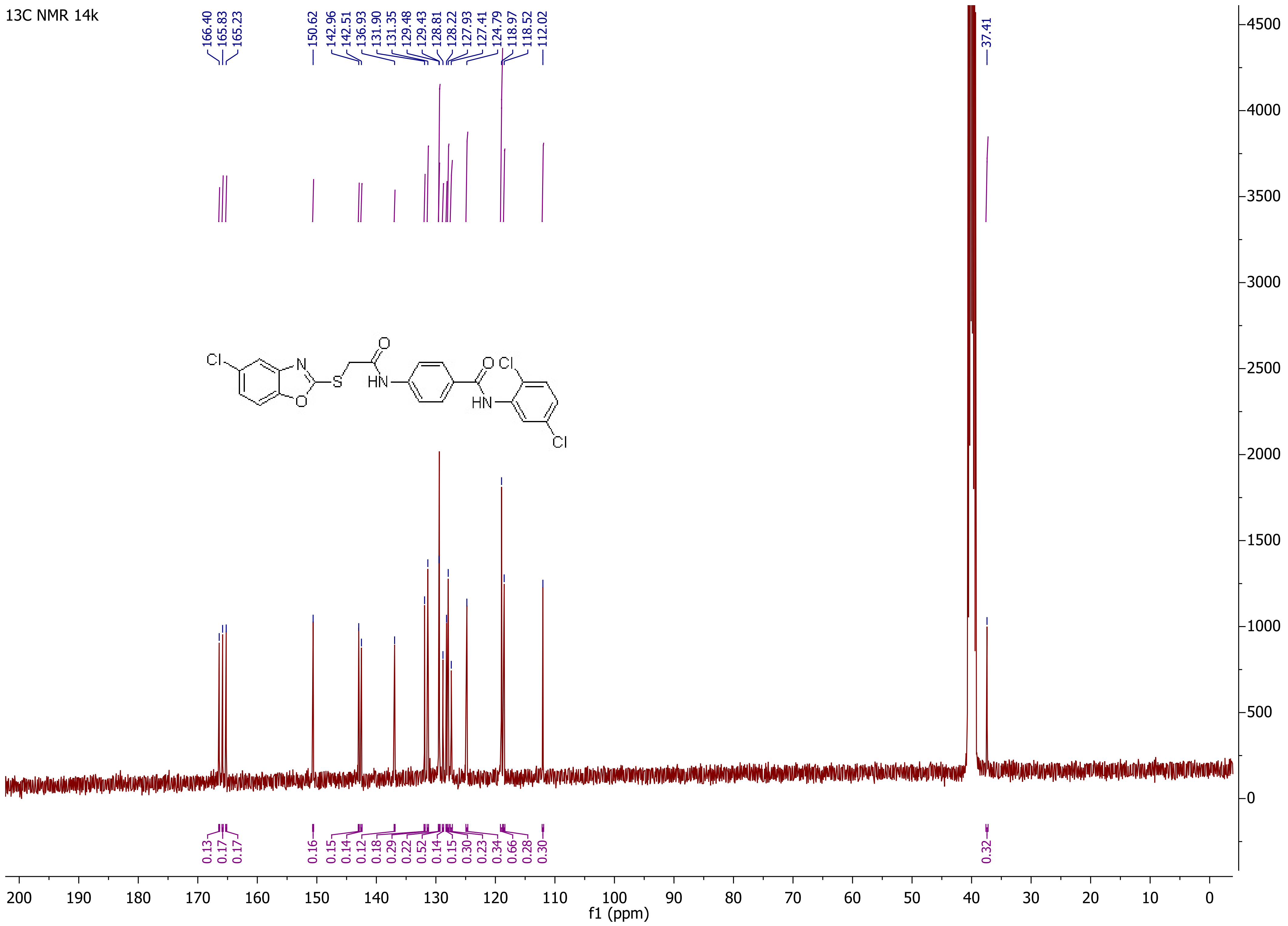

13C NMR 14k
166.40
165.83
165.23
150.62
142.96
142.51
136.93
131.90
131.35
129.48
129.43
128.81
128.22
127.93
127.41
124.79
118.97
118.52
112.02
37.41
0.13
0.17
0.17
0.16
0.15
0.14
0.12
0.18
0.29
0.22
0.52
0.14
0.15
0.30
0.23
0.34
0.66
0.28
0.30
0.32
f1 (ppm)

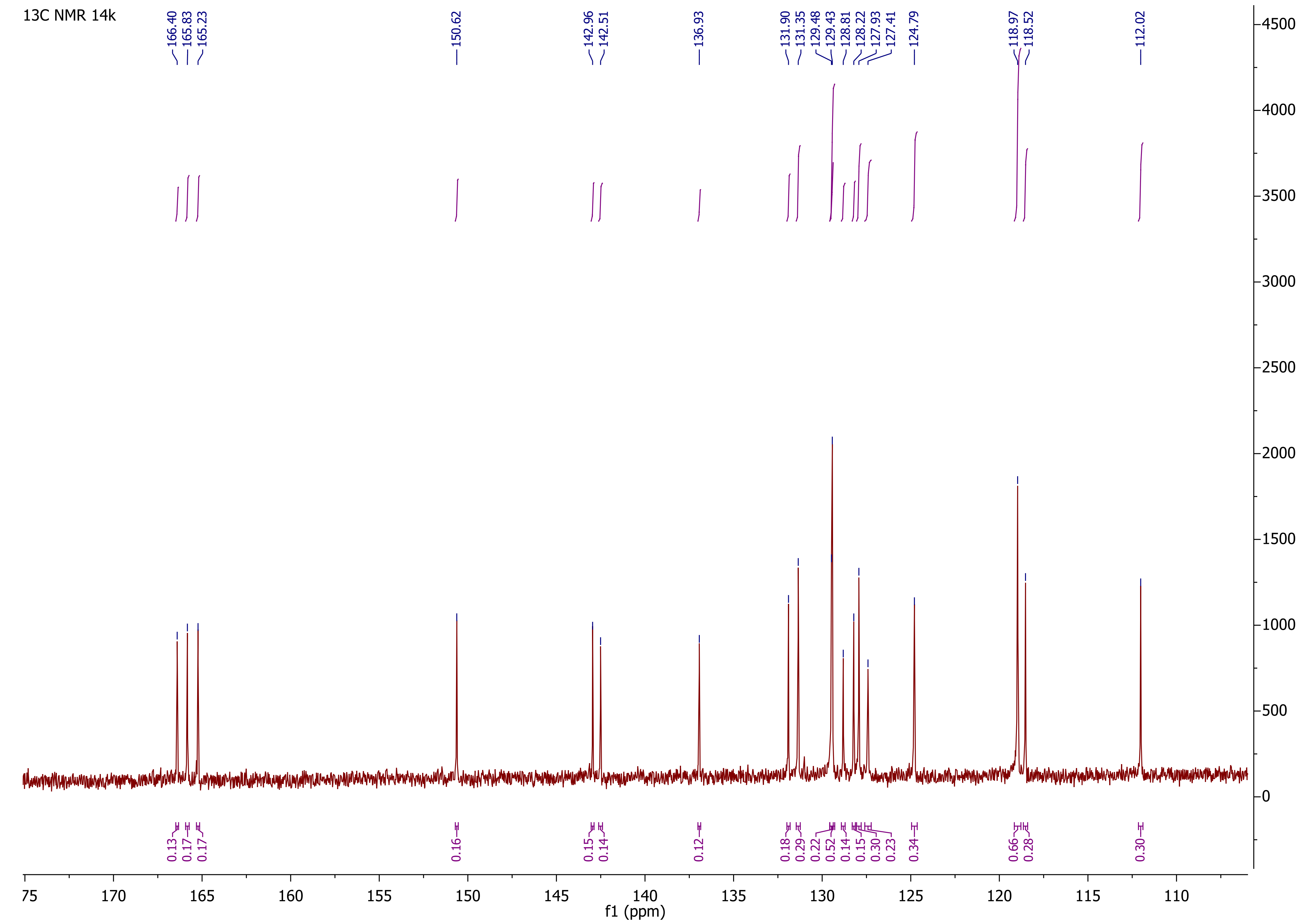

13C NMR 14k
166.40
165.83
165.23
150.62
142.96
142.51
136.93
131.90
131.35
129.48
129.43
128.81
128.22
127.93
127.41
124.79
118.97
118.52
112.02
0.13
0.17
0.17
0.16
0.15
0.14
0.12
0.18
0.29
0.22
0.52
0.14
0.15
0.30
0.23
0.34
0.66
0.28
0.30
f1 (ppm)

IR of comp. 14l

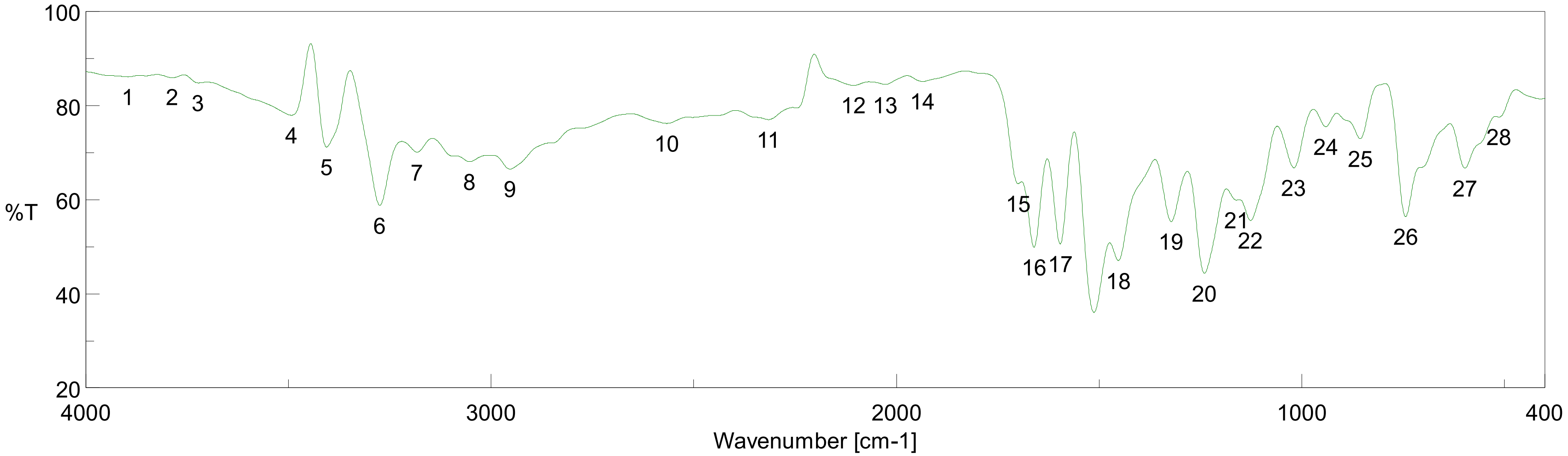

[Result of Peak Picking]

No.	Position	Intensity	No.	Position	Intensity	No.	Position	Intensity	No.	Position	Intensity
1	3896.47	86.0654	2	3787.51	85.8935	3	3722.91	84.7765	4	3492.45	77.9104
5	3405.67	71.147	6	3274.54	58.7631	7	3182.93	70.0625	8	3052.76	68.0576
9	2953.45	66.4723	10	2566.79	76.1726	11	2316.09	76.9798	12	2105.89	84.2624
13	2027.78	84.4463	14	1935.22	85.0842	15	1699.94	63.3668	16	1660.41	49.8673
17	1595.81	50.5504	18	1452.14	47.0185	19	1321.96	55.3153	20	1240	44.3345
21	1160.94	59.8701	22	1126.22	55.5455	23	1019.19	66.7325	24	940.128	75.4764
25	855.275	72.9285	26	743.424	56.363	27	596.861	66.6751	28	512.972	77.5242

1H NMR 14l

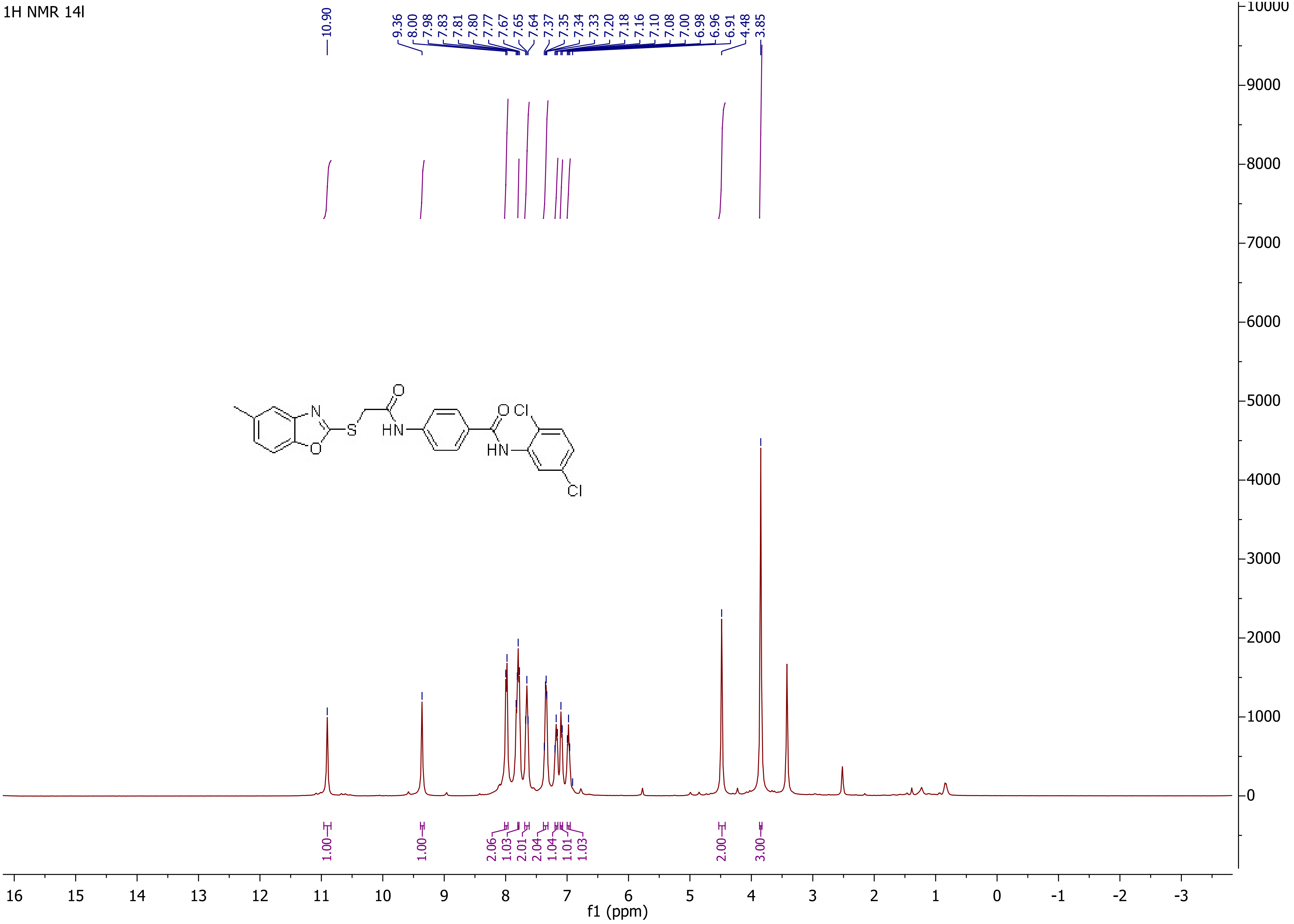
10.90
9.36
8.00
7.98
7.83
7.81
7.80
7.77
7.67
7.65
7.64
7.37
7.35
7.34
7.33
7.20
7.18
7.16
7.10
7.08
7.00
6.98
6.96
6.91
4.48
3.85
1.00
1.00
2.06
1.03
2.01
2.04
1.04
1.01
1.03
2.00
3.00
f1 (ppm)

1H NMR 14l

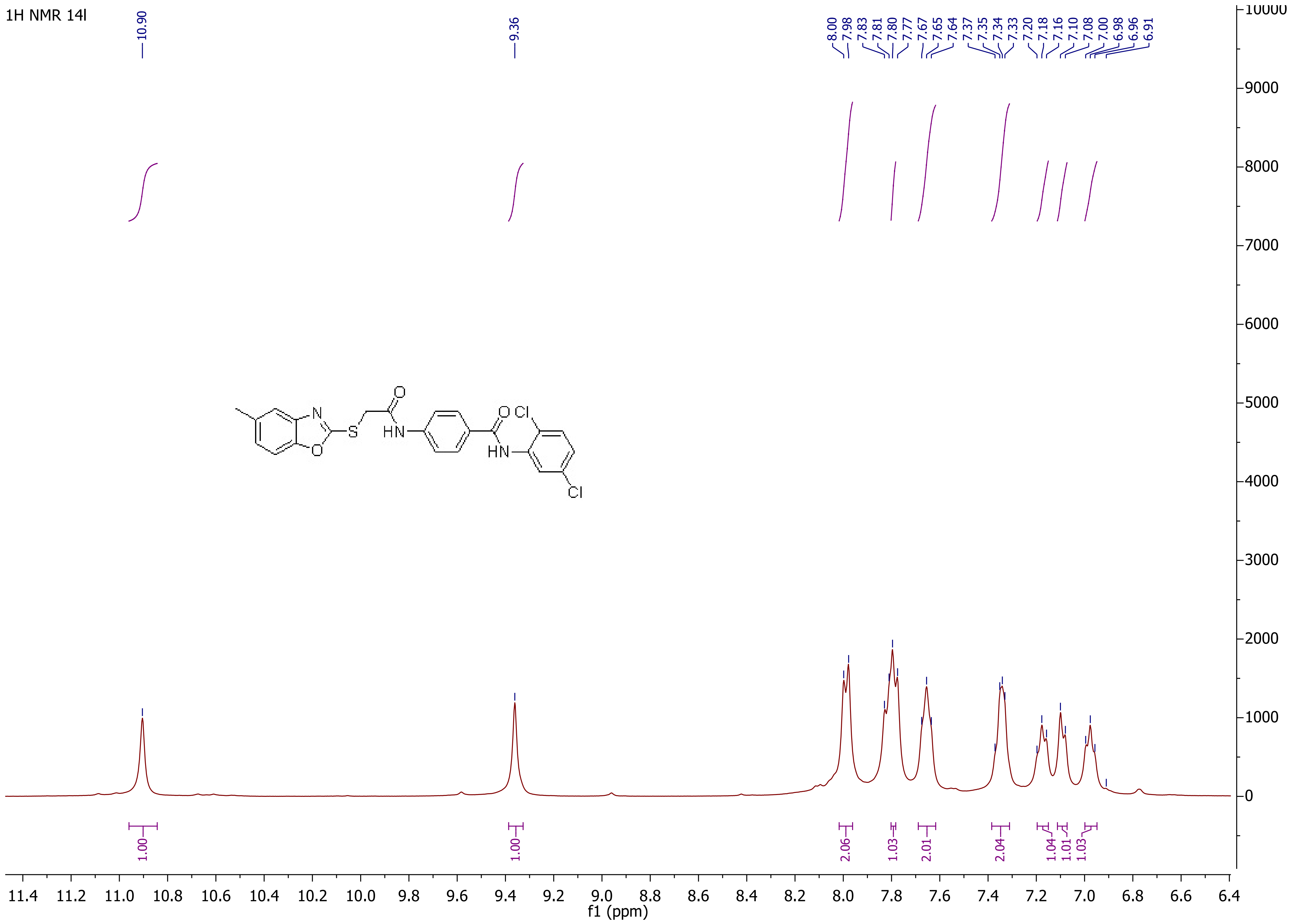

10.90
9.36
8.00
7.98
7.83
7.81
7.80
7.77
7.67
7.65
7.64
7.37
7.35
7.34
7.33
7.20
7.18
7.16
7.10
7.08
7.00
6.98
6.96
6.91
N
O
S
O
HN
O
Cl
HN
Cl
1.00
1.00
2.06
1.03
2.01
2.04
1.04
1.01
1.03
f1 (ppm)

13C NMR 14l

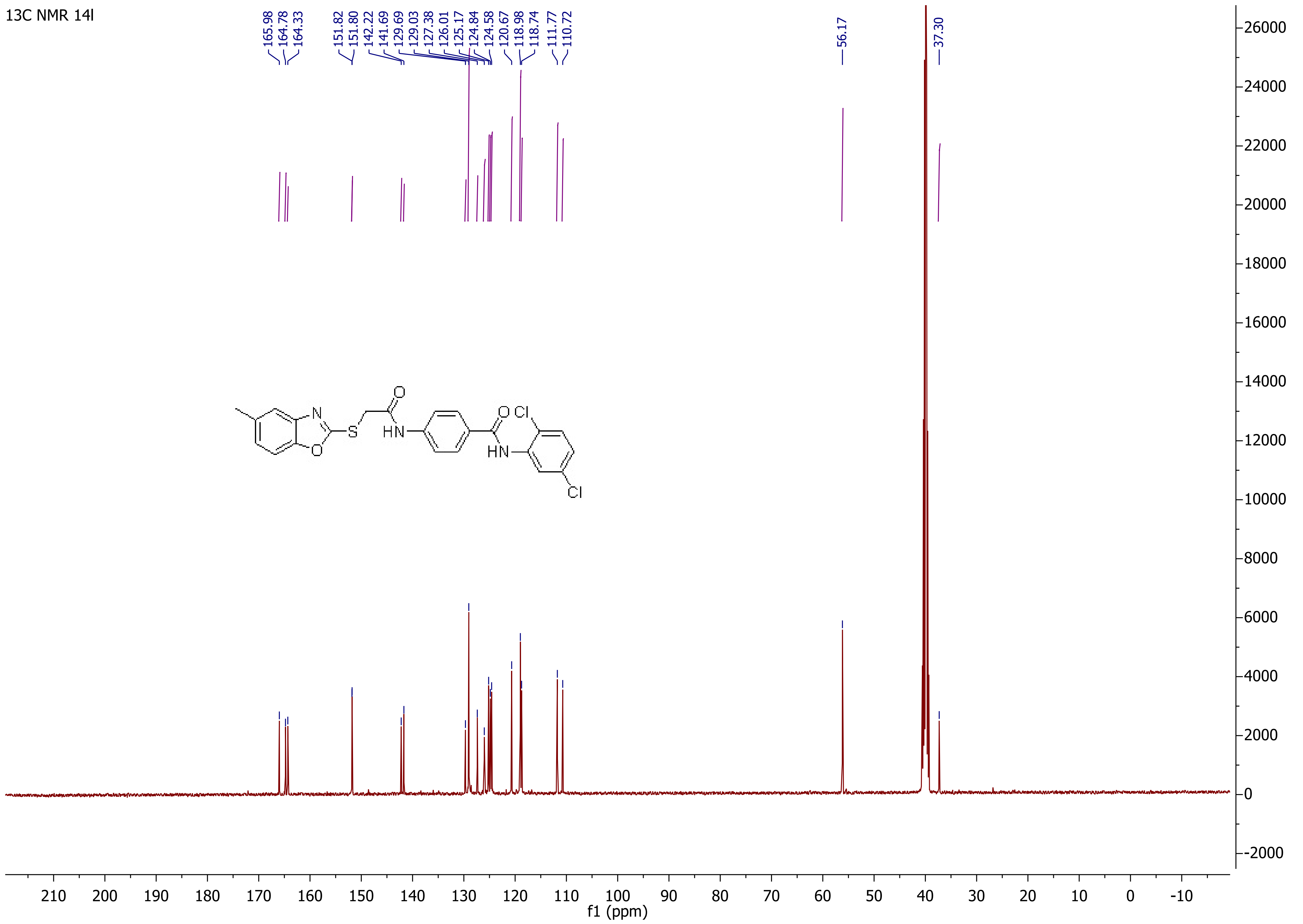

165.98
164.78
164.33
151.82
151.80
142.22
141.69
129.69
129.03
127.38
126.01
125.17
124.84
124.58
120.67
118.98
118.74
111.77
110.72
56.17
37.30
N
O
S
HN
O
O
Cl
HN
Cl
f1 (ppm)

13C NMR 14l

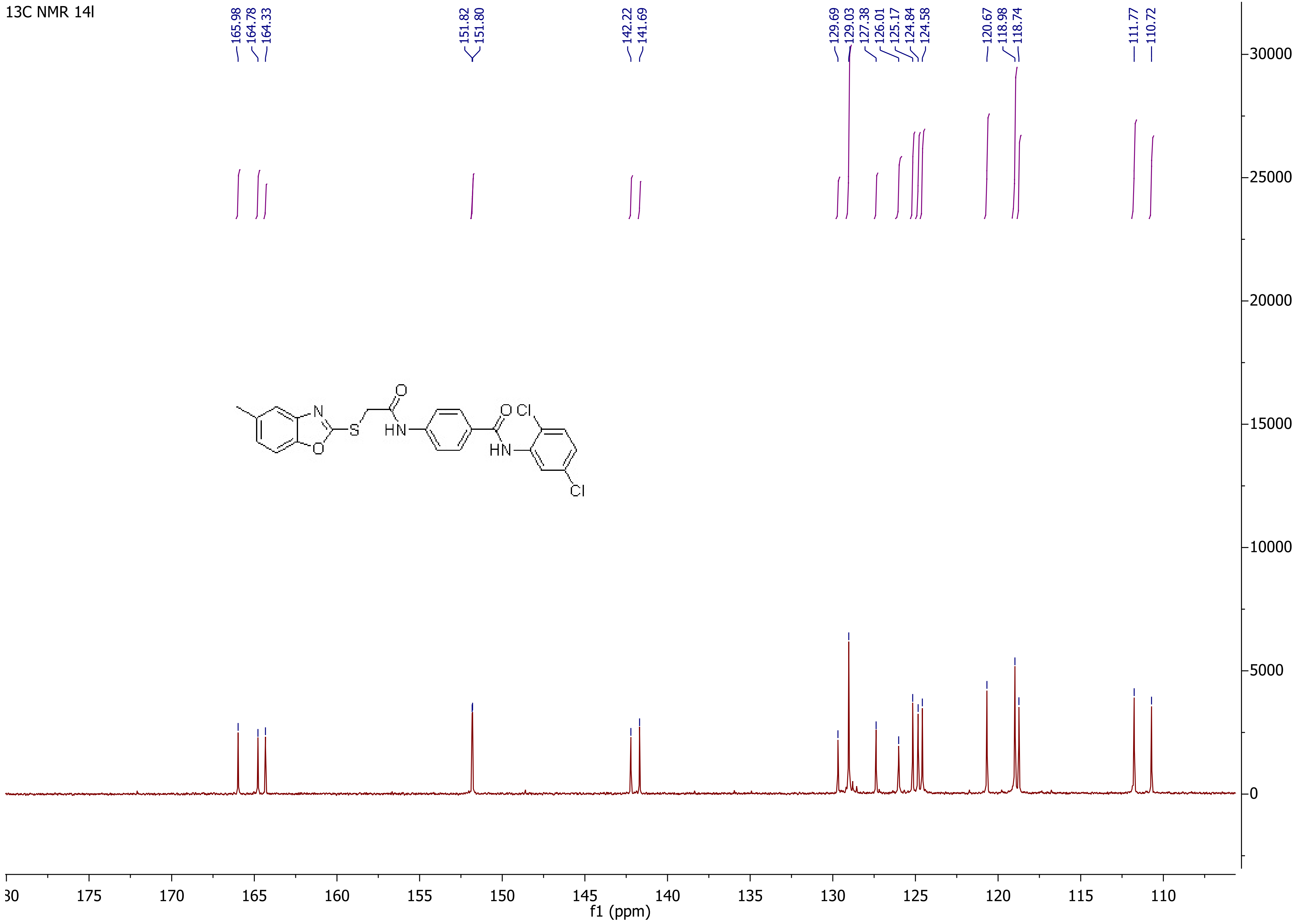
165.98
164.78
164.33
151.82
151.80
142.22
141.69
129.69
129.03
127.38
126.01
125.17
124.84
124.58
120.67
118.98
118.74
111.77
110.72
30000
25000
20000
15000
10000
5000
0
30
175
170
165
160
155
150
145
140
135
130
125
120
115
110
f1 (ppm)
N
O
S
O
HN
O
Cl
HN
Cl

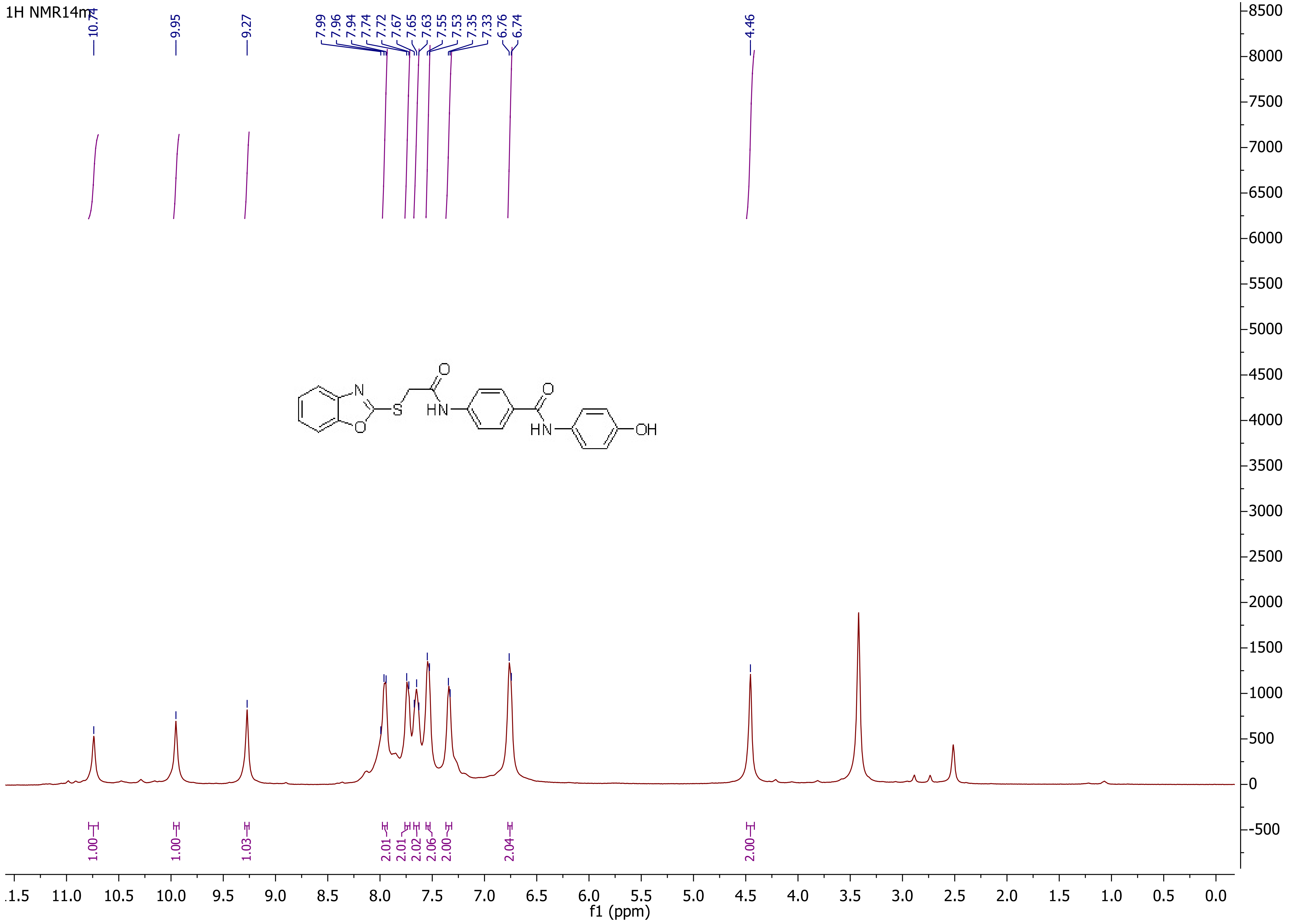
1H NMR14m
10.74
9.95
9.27
7.99
7.96
7.94
7.74
7.72
7.67
7.65
7.63
7.55
7.53
7.35
7.33
6.76
6.74
4.46
1.00
1.00
1.03
2.01
2.01
2.02
2.06
2.00
2.04
2.00
N
O
S
HN
O
HN
O
OH
f1 (ppm)

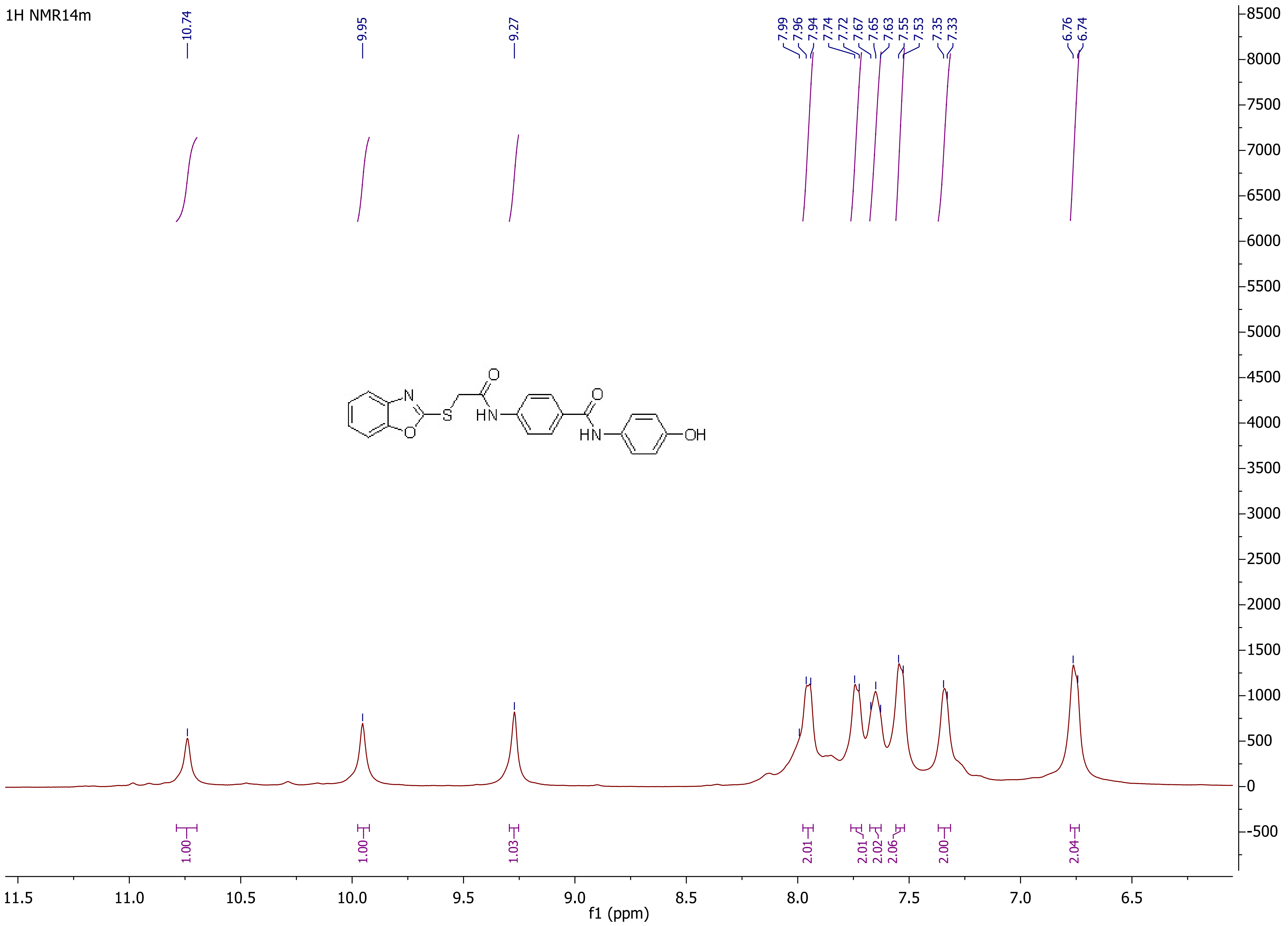
1H NMR14m
10.74
9.95
9.27
7.99
7.96
7.94
7.74
7.72
7.67
7.65
7.63
7.55
7.53
7.35
7.33
6.76
6.74
1.00
1.00
1.03
2.01
2.01
2.02
2.06
2.00
2.04
f1 (ppm)

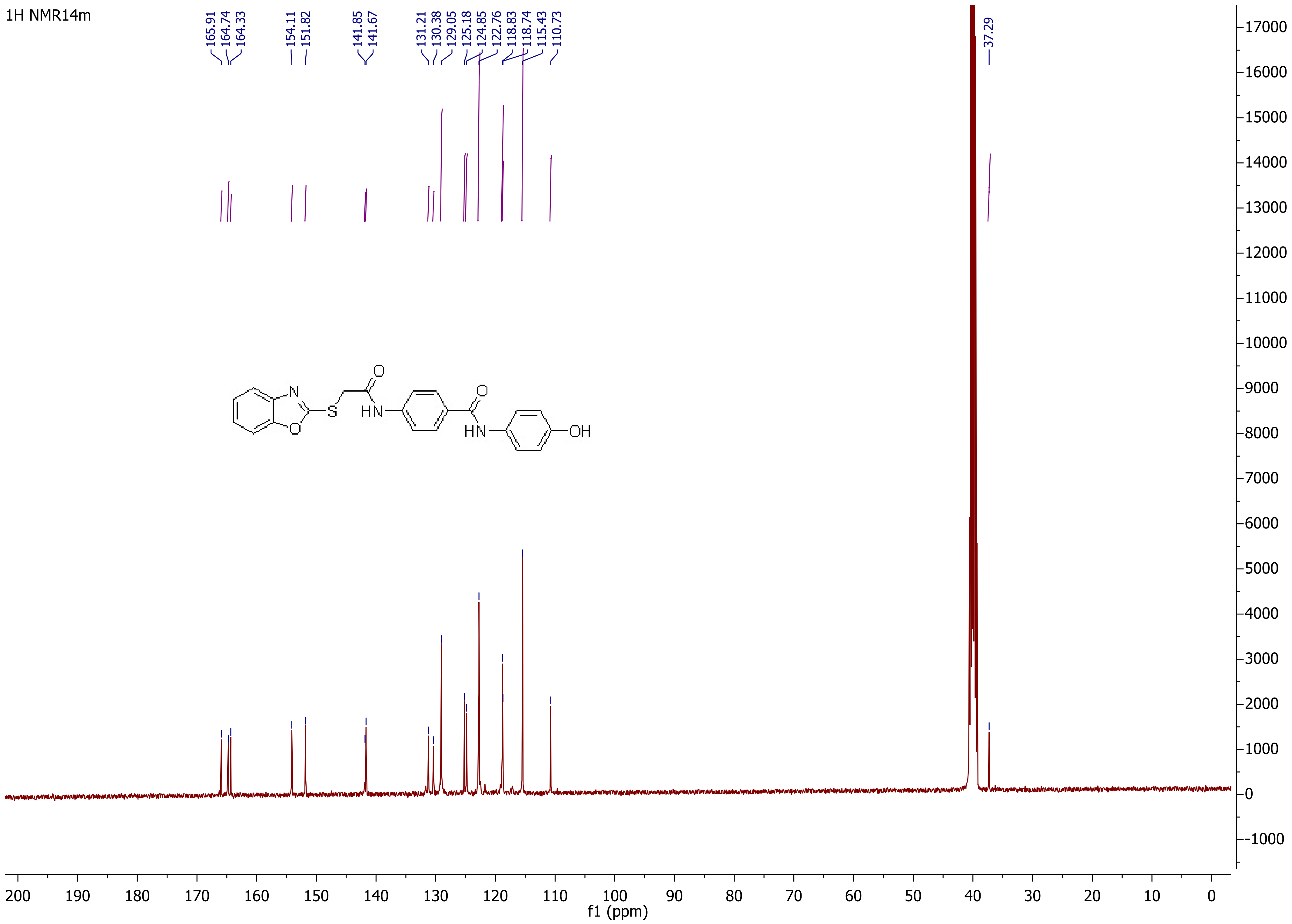
1H NMR14m
165.91
164.74
164.33
154.11
151.82
141.85
141.67
131.21
130.38
129.05
125.18
124.85
122.76
118.83
118.74
115.43
110.73
37.29
N
O
S
HN
O
O
HN
OH
f1 (ppm)
200 190 180 170 160 150 140 130 120 110 100 90 80 70 60 50 40 30 20 10 0
17000 16000 15000 14000 13000 12000 11000 10000 9000 8000 7000 6000 5000 4000 3000 2000 1000 0 -1000

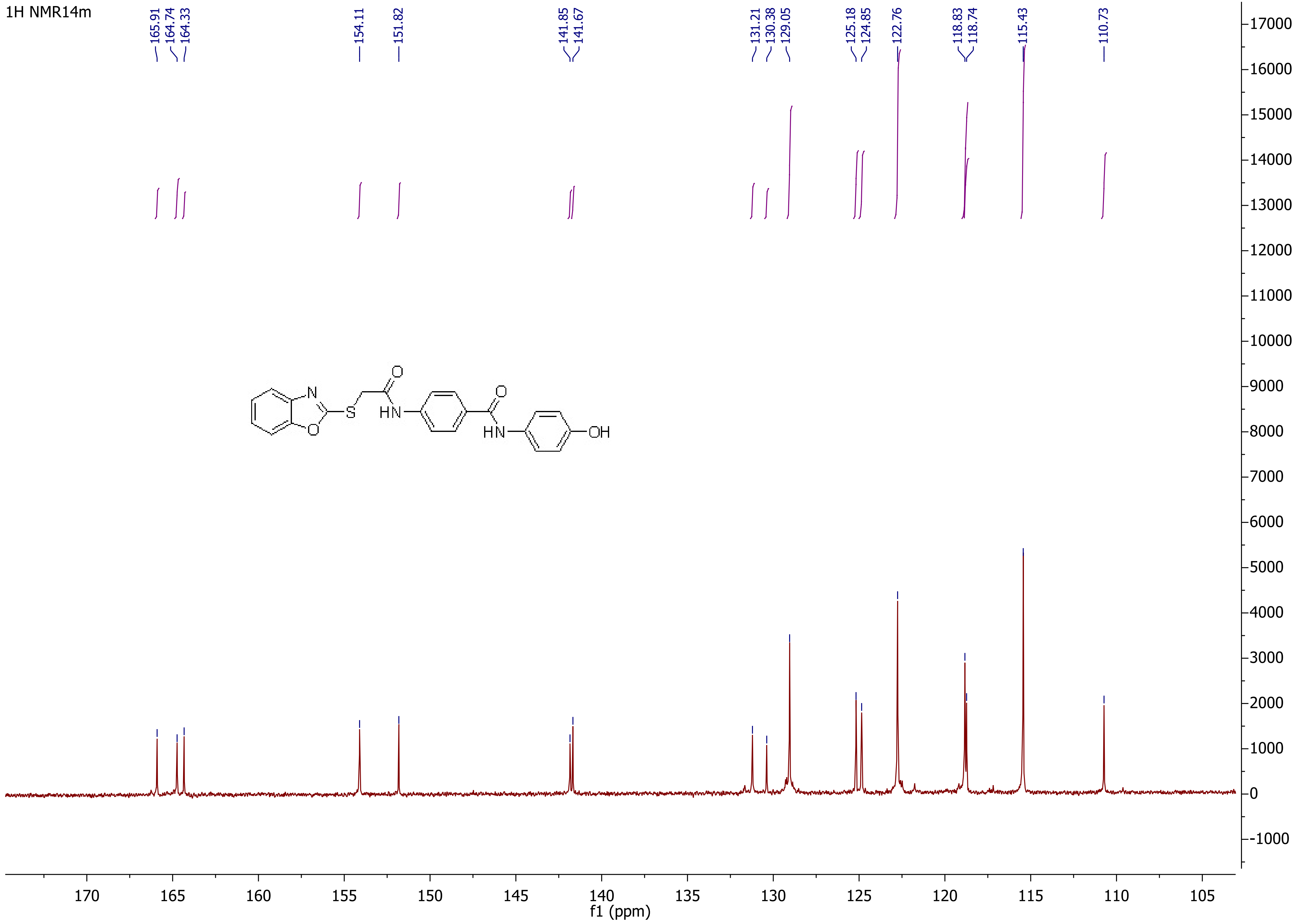
1H NMR14m
165.91
164.74
164.33
154.11
151.82
141.85
141.67
131.21
130.38
129.05
125.18
124.85
122.76
118.83
118.74
115.43
110.73
N
O
S
O
HN
O
HN
OH
f1 (ppm)

IR of comp. 14n

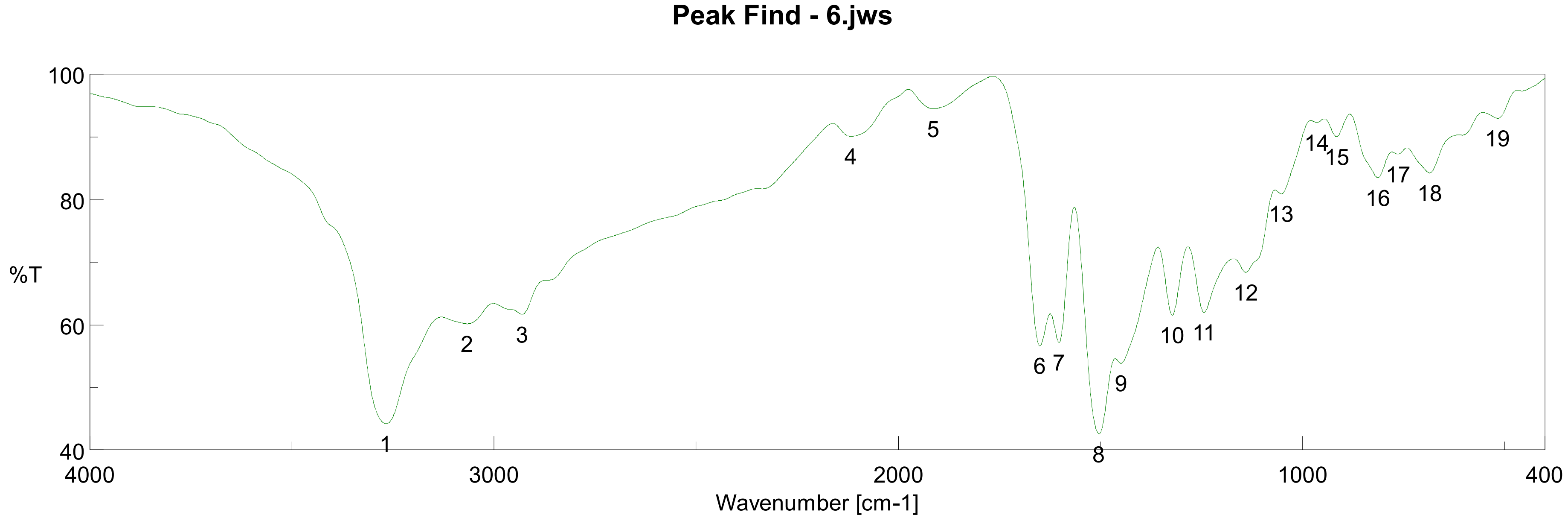

[Result of Peak Picking]

No.	Position	Intensity	No.	Position	Intensity	No.	Position	Intensity	No.	Position	Intensity
1	3267.79	44.1754	2	3067.23	60.1331	3	2930.31	61.6399	4	2117.46	90.0422
5	1913.04	94.4479	6	1649.8	56.6161	7	1602.56	57.1482	8	1503.24	42.5505
9	1448.28	53.8164	10	1321.96	61.5063	11	1242.9	61.9459	12	1139.72	68.3375
13	1051.98	80.8706	14	964.233	92.2747	15	915.058	90.0159	16	812.849	83.4462
17	763.673	87.2229	18	684.606	84.2249	19	516.829	92.9242			

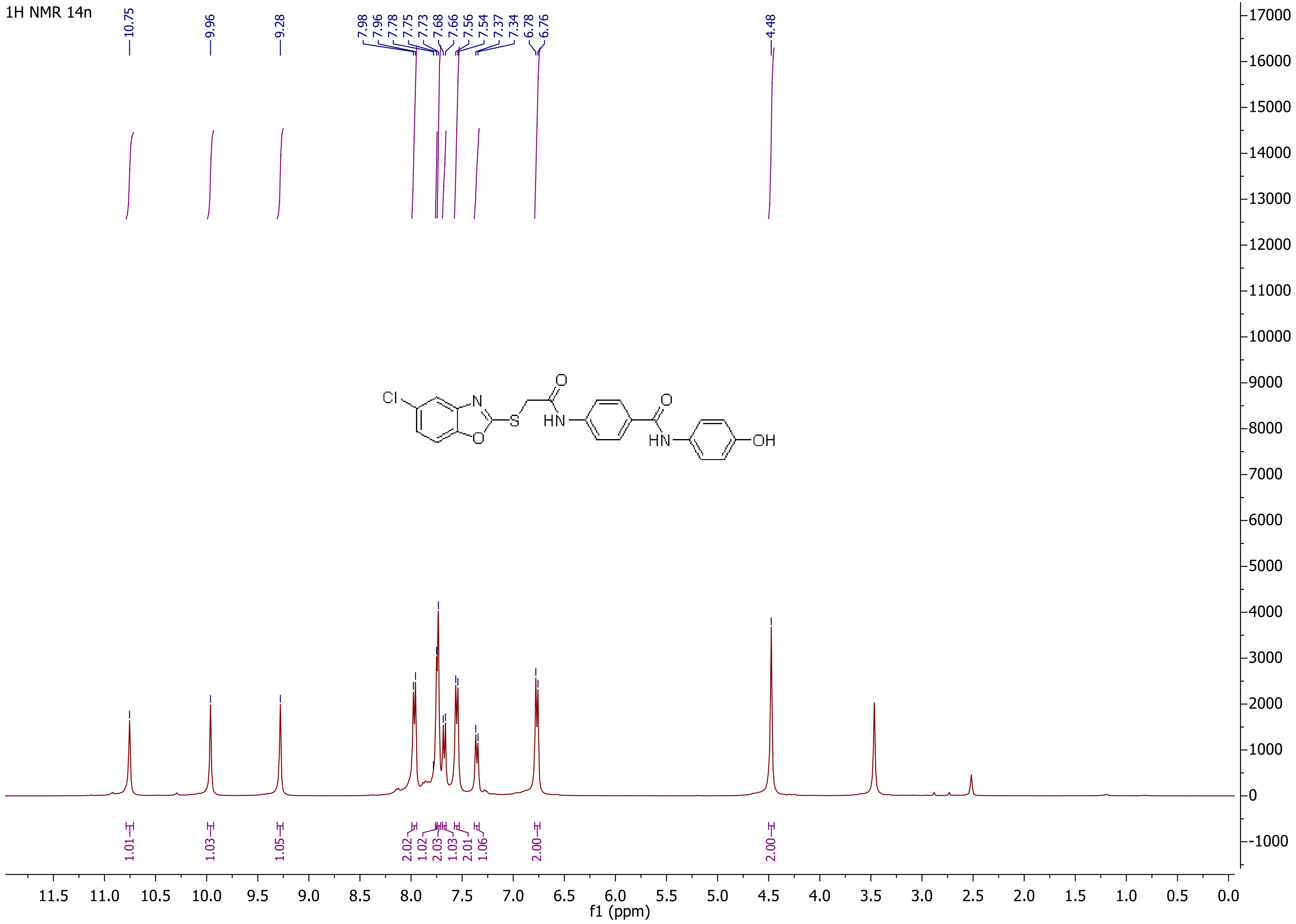
1H NMR 14n
10.75
9.96
9.28
7.98
7.96
7.78
7.75
7.73
7.68
7.66
7.56
7.54
7.37
7.34
6.78
6.76
4.48
Cl
N
O
S
O
HN
O
HN
OH
1.01
1.03
1.05
2.02
1.02
2.03
1.03
2.01
1.06
2.00
2.00
f1 (ppm)

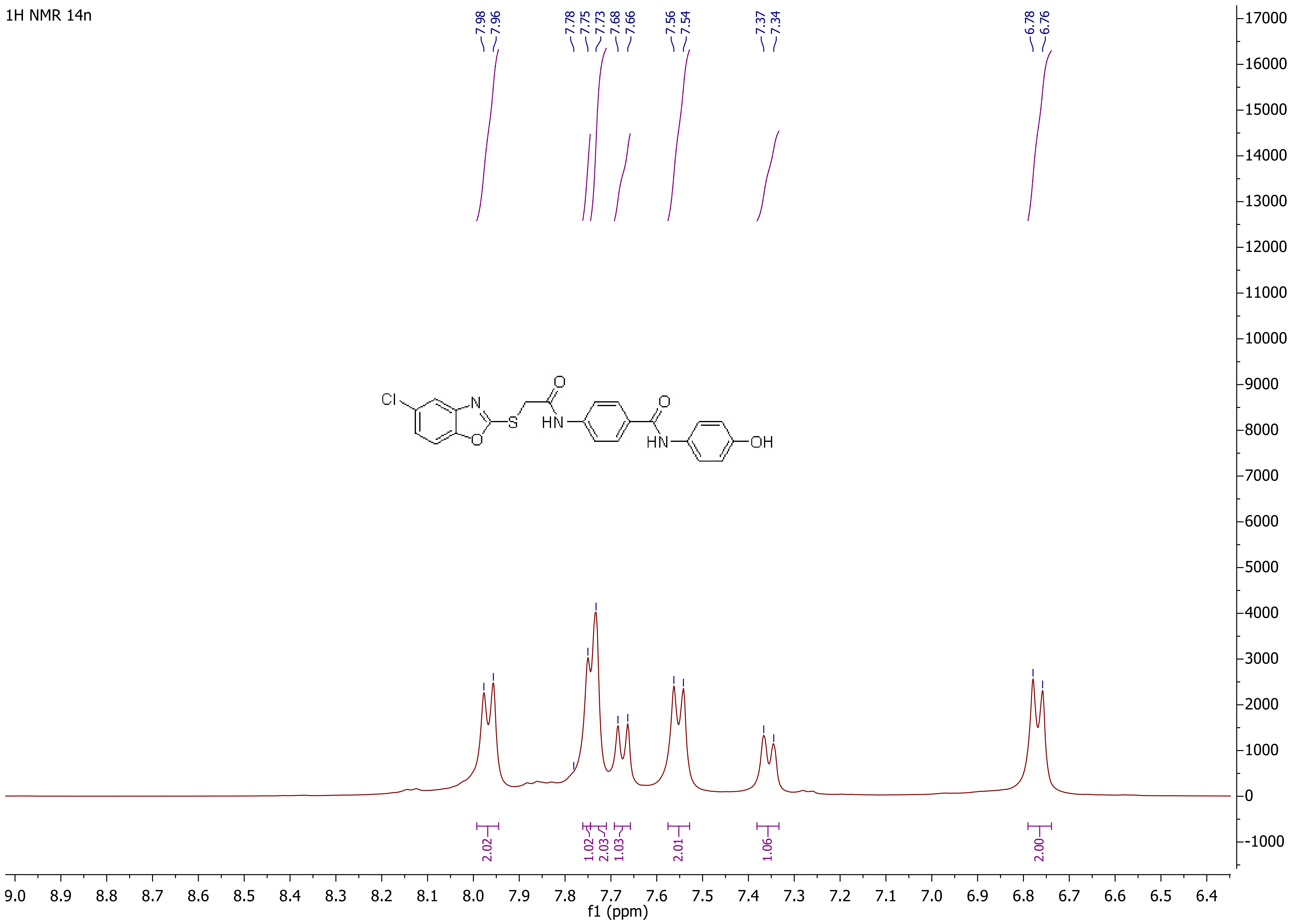
1H NMR 14n
7.98
7.96
7.78
7.75
7.73
7.68
7.66
7.56
7.54
7.37
7.34
6.78
6.76
Cl
N
O
S
HN
O
O
HN
OH
2.02
1.02
2.03
1.03
2.01
1.06
2.00
f1 (ppm)

13C NMR 14n

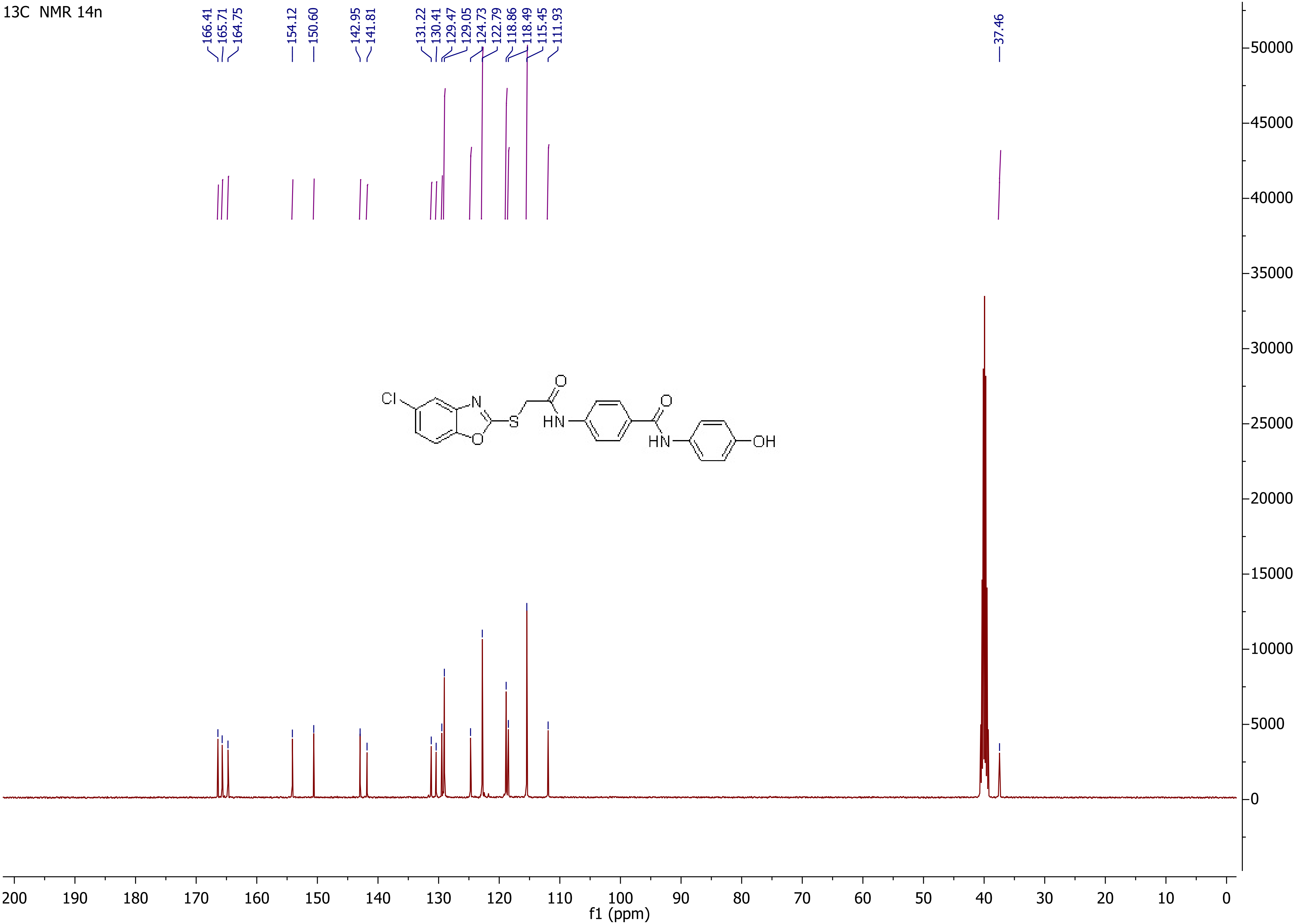

166.41
165.71
164.75
154.12
150.60
142.95
141.81
131.22
130.41
129.47
129.05
124.73
122.79
118.86
118.49
115.45
111.93
37.46
Cl
N
O
S
HN
O
HN
OH
f1 (ppm)

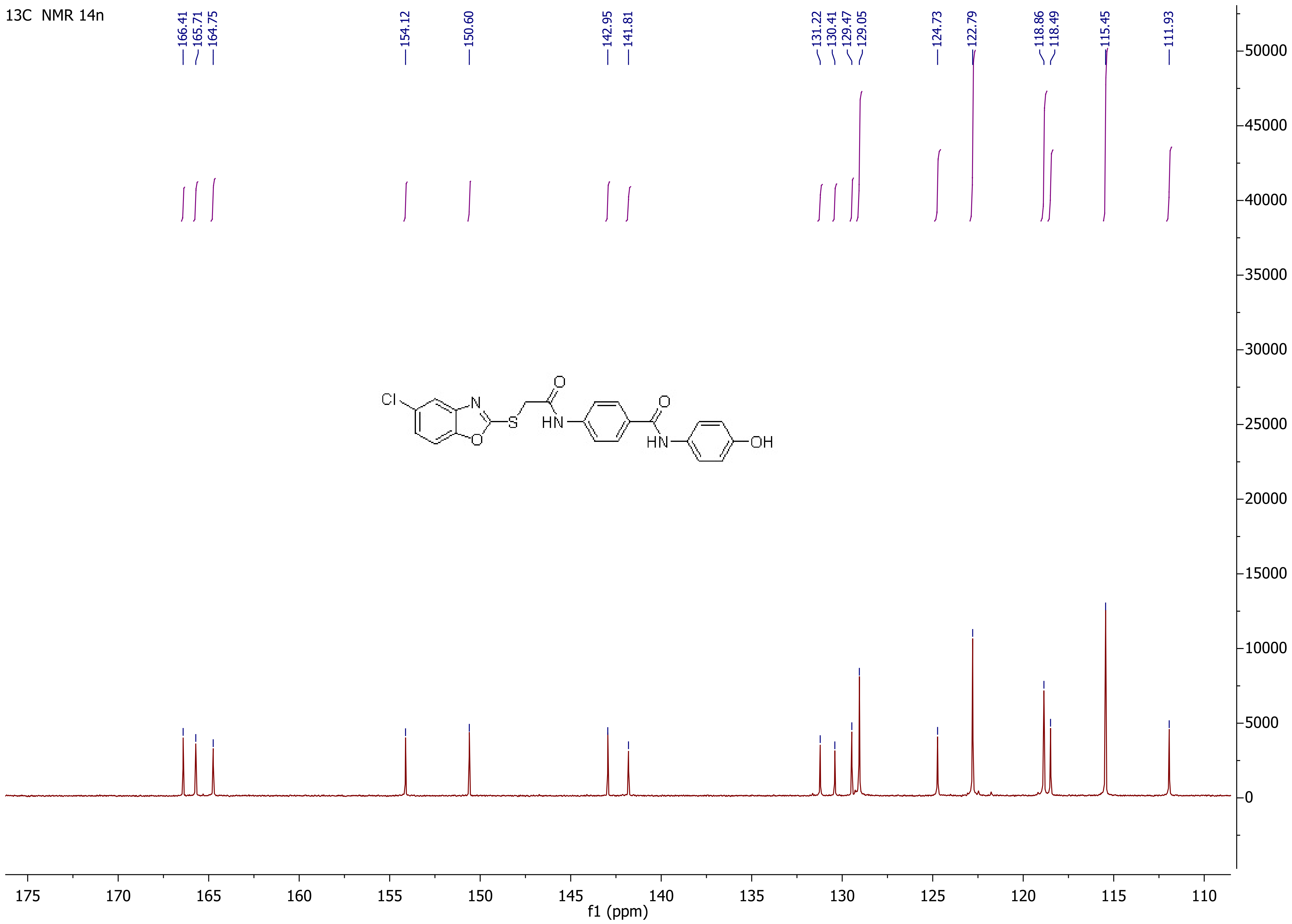

13C NMR 14n
166.41
165.71
164.75
154.12
150.60
142.95
141.81
131.22
130.41
129.47
129.05
124.73
122.79
118.86
118.49
115.45
111.93
Cl
N
O
S
HN
O
HN
OH
f1 (ppm)

IR of comp. 14o

Peak Find - 11.jws

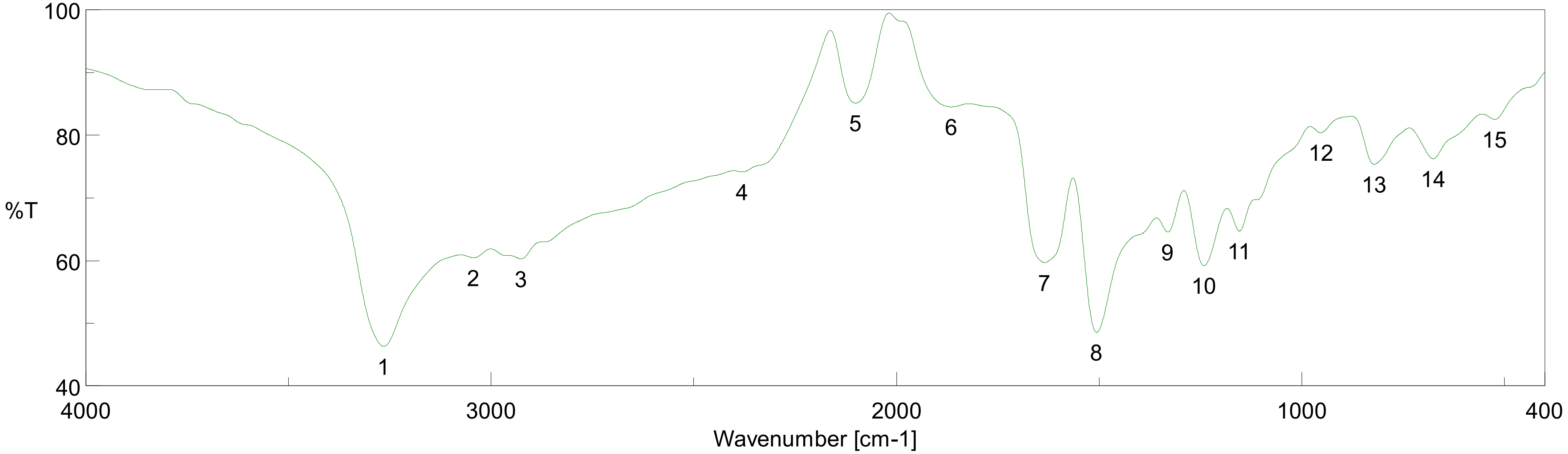

[Result of Peak Picking]

No.	Position	Intensity	No.	Position	Intensity	No.	Position	Intensity	No.	Position	Intensity
1	3264.89	46.2911	2	3043.12	60.4206	3	2926.45	60.2395	4	2379.73	74.1031
5	2101.06	85.0865	6	1863.86	84.4831	7	1634.38	59.6454	8	1506.13	48.5207
9	1330.64	64.5026	10	1240.97	59.1656	11	1153.22	64.6723	12	952.663	80.3541
13	820.563	75.3108	14	675.928	76.1907	15	523.579	82.4742			

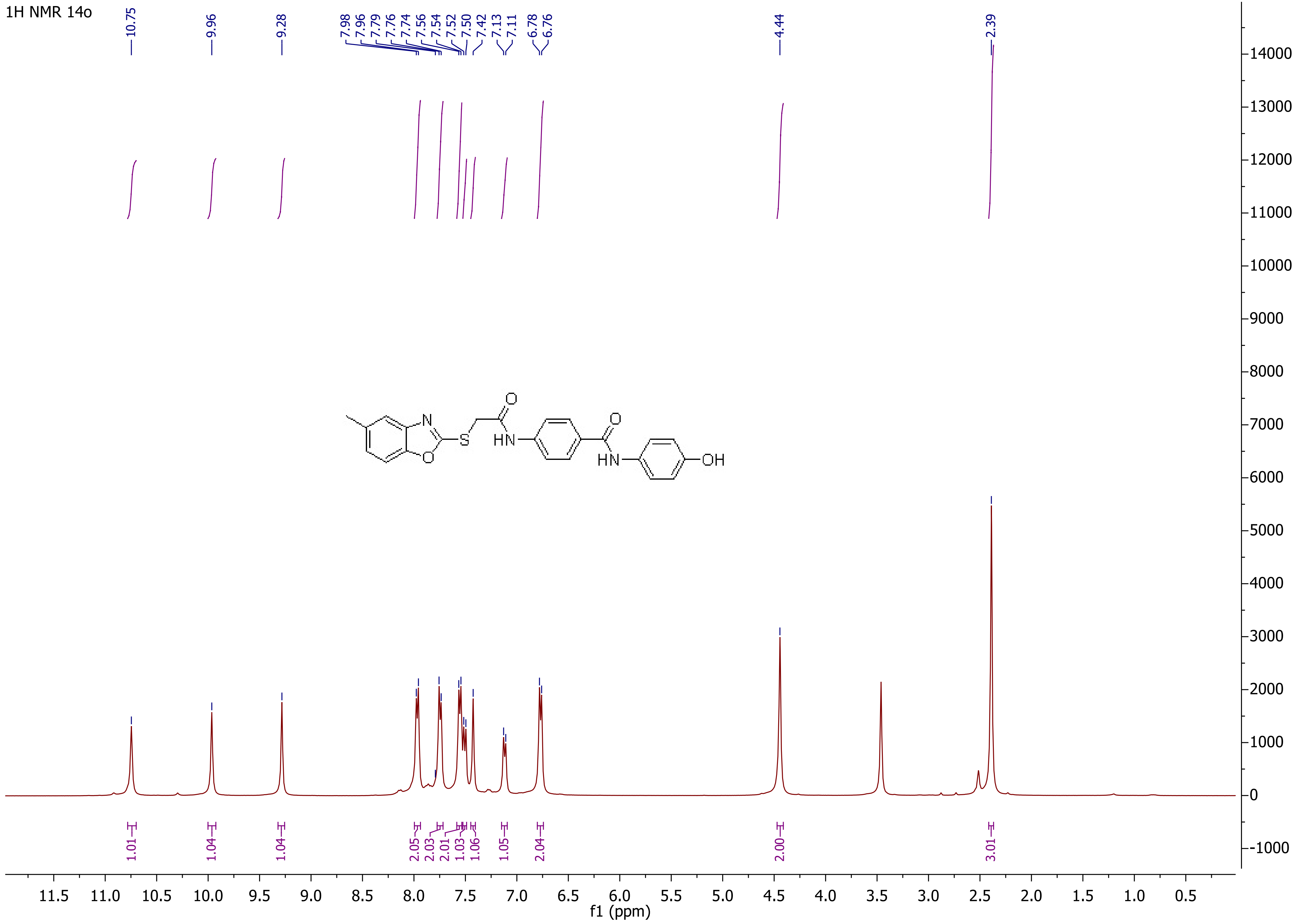

1H NMR 14o
10.75
9.96
9.28
7.98
7.96
7.79
7.76
7.74
7.56
7.54
7.52
7.50
7.42
7.13
7.11
6.78
6.76
4.44
2.39
N
S
O
HN
O
HN
O
OH
1.01
1.04
1.04
2.05
2.03
2.01
1.03
1.06
1.05
2.04
2.00
3.01
f1 (ppm)

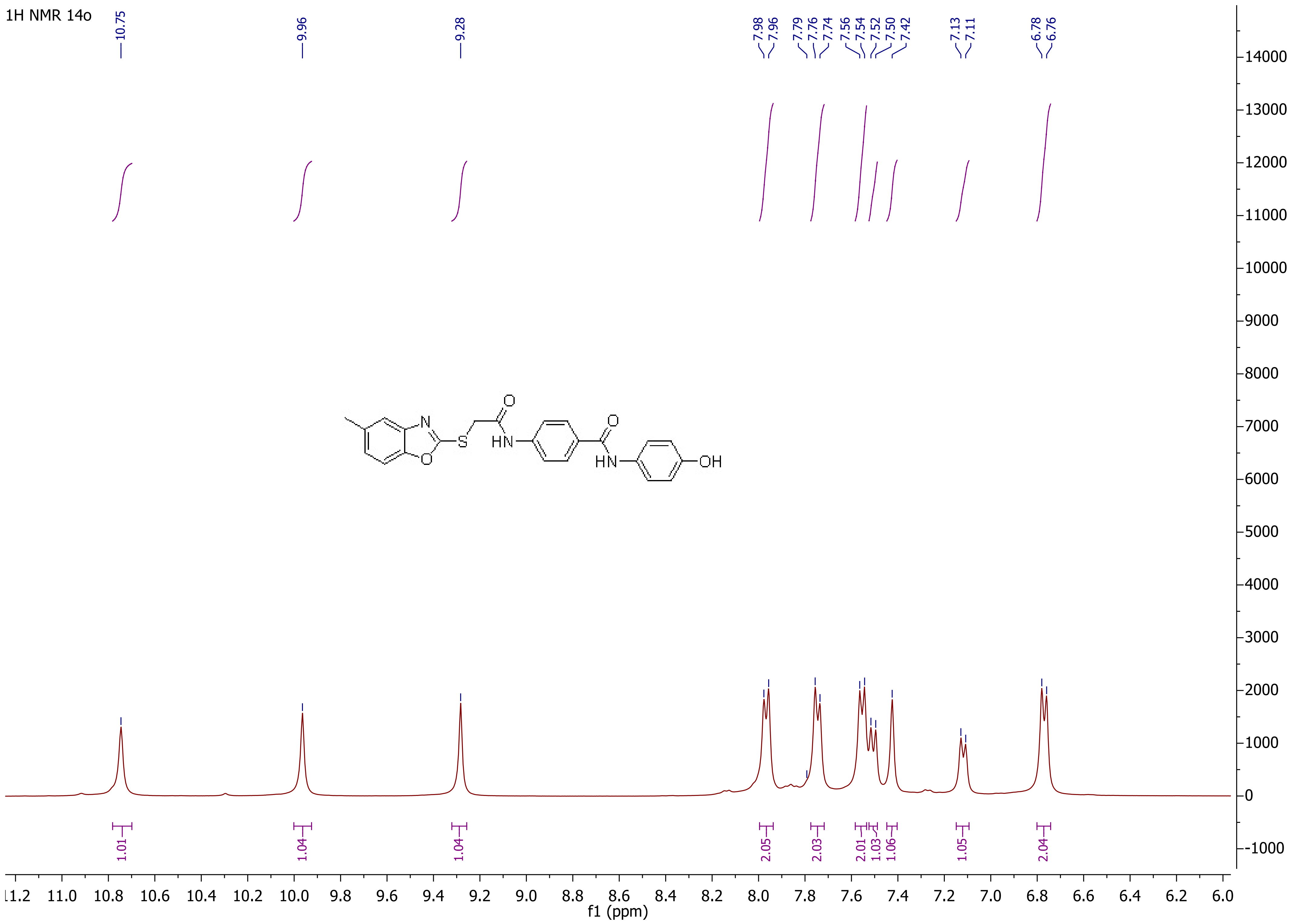
1H NMR 14o
10.75
9.96
9.28
7.98
7.96
7.79
7.76
7.74
7.56
7.54
7.52
7.50
7.42
7.13
7.11
6.78
6.76
N
S
O
HN
O
HN
O
OH
1.01
1.04
1.04
2.05
2.03
2.01
1.03
1.06
1.05
2.04
14000
13000
12000
11000
10000
9000
8000
7000
6000
5000
4000
3000
2000
1000
0
-1000
11.2
11.0
10.8
10.6
10.4
10.2
10.0
9.8
9.6
9.4
9.2
9.0
8.8
8.6
8.4
8.2
8.0
7.8
7.6
7.4
7.2
7.0
6.8
6.6
6.4
6.2
6.0
f1 (ppm)

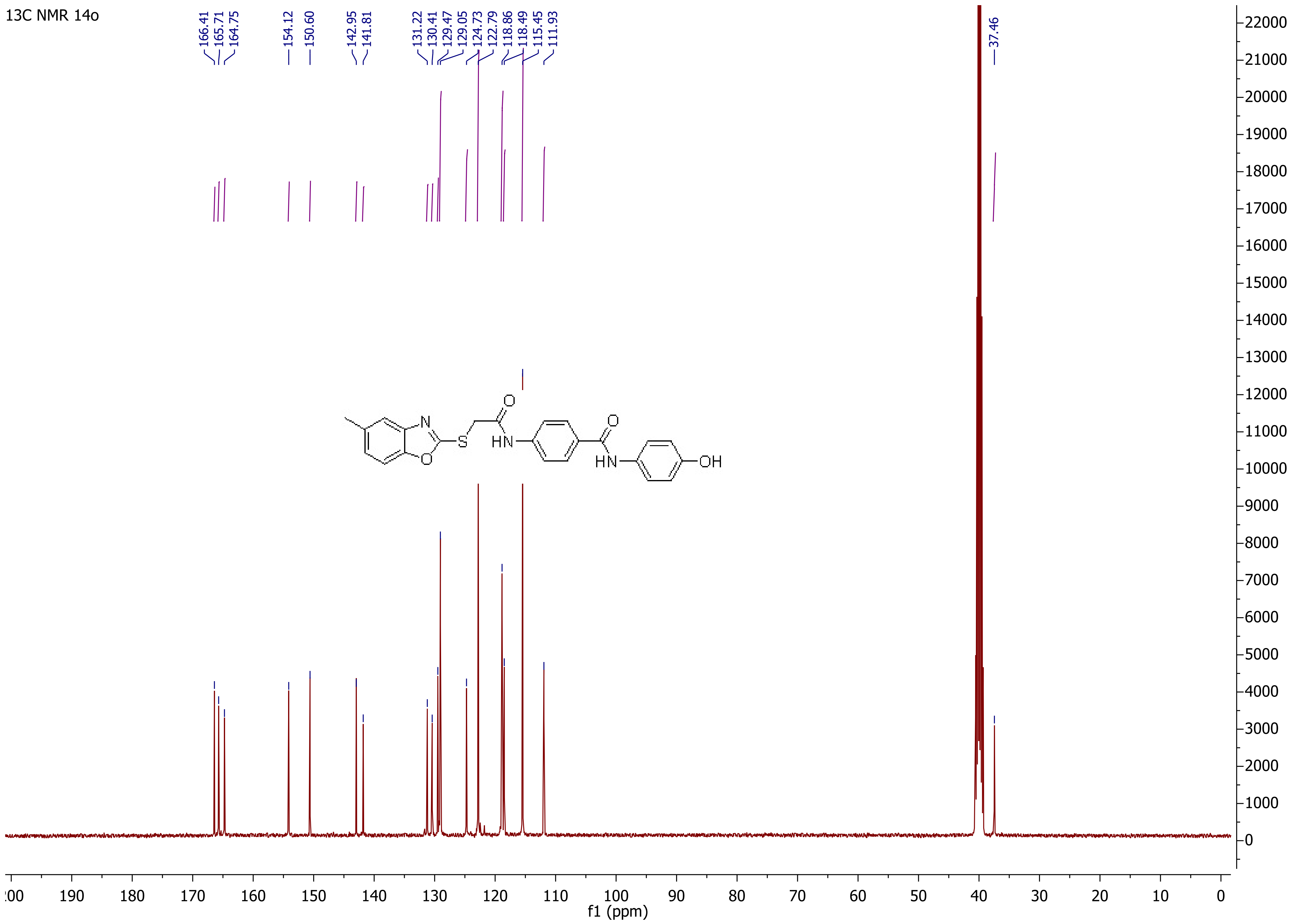
13C NMR 14o
166.41
165.71
164.75
154.12
150.60
142.95
141.81
131.22
130.41
129.47
129.05
124.73
122.79
118.86
118.49
115.45
111.93
37.46
N
O
S
O
HN
O
HN
OH
f1 (ppm)

13C NMR 14o

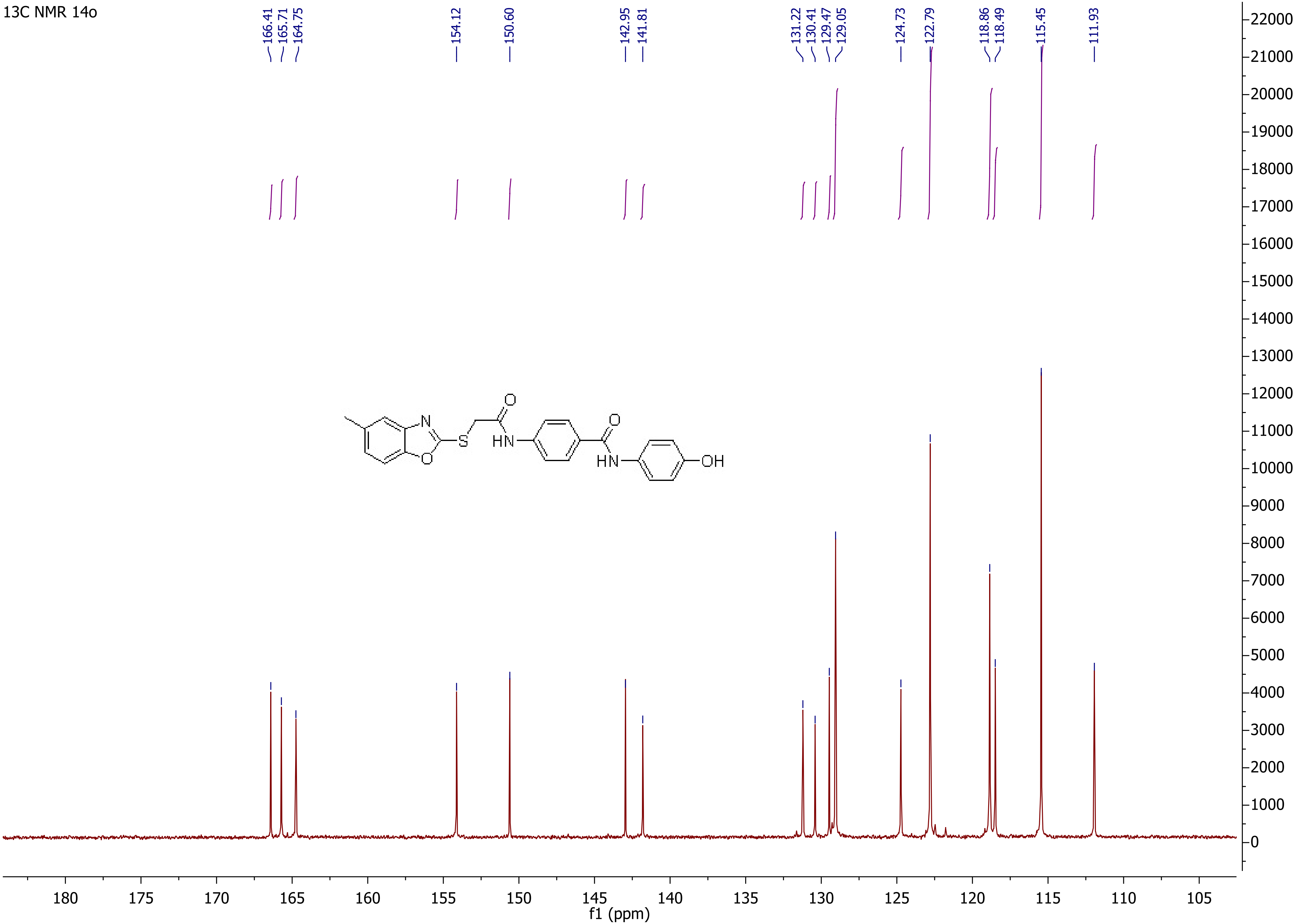
166.41
165.71
164.75
154.12
150.60
142.95
141.81
131.22
130.41
129.47
129.05
124.73
122.79
118.86
118.49
115.45
111.93
HN
HN
OH
S
N
O
f1 (ppm)

2.2. *In silico* ADME analysis

Pharmacokinetic properties (ADMET studies) of all compounds were also determined using Discovery Studio 4.0 software. The ADMET studies were investigated for sorafenib as well. In this respect, different descriptors were calculated including blood brain barrier (BBB) penetration, aqueous solubility, intestinal absorption, CYP2D6 binding, and plasma protein binding.

Results showed that all the synthesized compounds possess satisfactory ADMET profile and drug-likeness properties. With regards to compounds' BBB penetration levels, all compounds were expected to have no BBB permeability except for the **14a** analogue. Therefore, no possible side effects on the CNS were predicted. Additionally, the graphical illustration **(Fig. 14)** predicted that almost all compounds have high to moderate gastrointestinal absorption compared to the reference drug, sorafenib (**Table 4**).

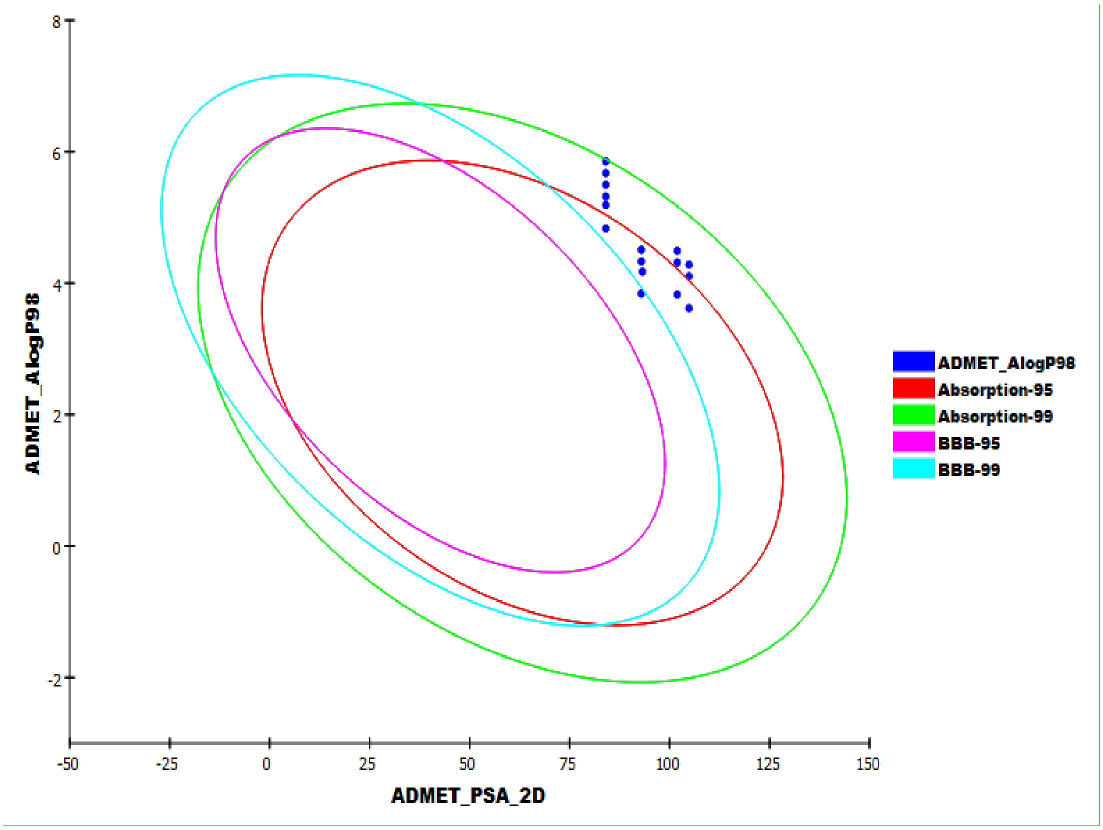

Fig. 14: The expected ADMET study.

Table 4. Predicted ADMET for the designed compounds and the reference drug

Comp.	BBB level[a]	Solubility level[b]	Absorption level[c]	CYP2D6 prediction[d]	PPB prediction[e]
14a	2	2	0	false	true
14b	4	2	0	false	true
14c	4	2	0	false	true
14d	4	2	0	false	false
14e	4	2	1	false	true
14f	4	2	1	false	true

14g	4	2	0	false	true
14h	4	1	1	false	true
14i	4	1	1	false	true
14j	4	1	1	false	true
14k	4	1	1	false	true
14l	4	1	1	false	true
14m	4	2	0	false	false
14n	4	2	1	false	false
14o	4	2	1	false	false
Sorafenib	4	1	0	false	true

[a] BBB level, blood brain barrier level, 0 = very high, 1 = high, 2 = medium, 3 = low, 4 = very low.
[b] Solubility level, 1 = very low, 2 = low, 3 = good, 4 = optimal.
[c] Absorption level, 0 = good, 1 = moderate, 2 = poor, 3 = very poor.
[d] CYP2D6, cytochrome P2D6, TRUE = inhibitor, FALSE = non inhibitor.
[e] PBB, plasma protein binding, FALSE means less than 90%, TRUE means more than 90%

2.3. Toxicity studies

The toxicity profiles of all the tested compounds were examined. This involves using of seven constructed toxicity models (illustrated in Table 5) utilizing Discovery studio 4.0 software.

The results revealed that all compounds were estimated to be non-carcinogen against FDA Rodent Carcinogenicity model except compound **14m-o.** Also**,** all the tested compounds exhibited TD_{50} values less than sorafenib, the values were ranging from 2.437 to 17.333 g/kg body. Furthermore, compounds **14g,h**, and **14j-o** displayed rat maximum tolerated dose values ranging from 0.089 to 0.460 g/kg body weight which were similar to or higher than that of sorafenib (0.089 g/kg body weight). Conversely, the rest of compounds showed maximum tolerated dose ranging from 0.050 to 0.085 g/kg body weight which were lower than that of sorafenib. Regarding rat oral LD_{50} model, all compounds except **14e** revealed rat oral LD_{50} values ranging from 1.073 to 4.988 g/kg body weight which were higher than that of sorafenib (0.823 g/kg body weight). Additionally**,** all compounds demonstrated LOAEL values ranging from 0.016 to 0.082 g/kg body weight which were extremely higher than that of sorafenib (0.005 g/kg body weight). Finally, all the tested members revealed mild irritancy and non-irritancy against ocular irritancy and skin irritancy models, respectively **(Table 5)**.

Table 5: Toxicity profile of the synthesized compounds

Comp.	FDA Rodent Carcinogenicity (Mouse- Female)	Carcinogenic Potency TD_{50} (Mouse)[a]	Rat Maximum Tolerated Dose (Feed)[b]	Rat Oral LD_{50}[b]	Rat Chronic LOAEL[b]	Ocular Irritancy	Skin Irritancy
**14a**	Non-Carcinogen	8.059	0.064	1.688	0.045	Mild	Non-Irritant
**14b**	Non-Carcinogen	9.300	0.078	1.073	0.031	Mild	Non-Irritant
**14c**	Non-Carcinogen	17.333	0.053	2.523	0.047	Mild	Non-Irritant
**14d**	Non-Carcinogen	8.291	0.062	1.258	0.044	Mild	Non-Irritant
**14e**	Non-Carcinogen	6.434	0.074	0.760	0.022	Mild	Non-Irritant
**14f**	Non-Carcinogen	12.024	0.050	1.747	0.030	Mild	Non-Irritant
**14g**	Non-Carcinogen	4.682	0.089	2.370	0.037	Mild	Non-Irritant
**14h**	Non-Carcinogen	3.651	0.109	1.437	0.019	Mild	Non-Irritant
**14i**	Non-Carcinogen	4.516	0.085	4.210	0.026	Mild	Non-Irritant
**14j**	Non-Carcinogen	4.830	0.112	2.207	0.034	Mild	Non-Irritant
**14k**	Non-Carcinogen	1.622	0.089	1.569	0.016	Mild	Non-Irritant
**14l**	Non-Carcinogen	2.437	0.092	3.168	0.017	Mild	Non-Irritant
**14m**	Single-Carcinogen	11.497	0.377	2.782	0.082	Mild	Non-Irritant
**14n**	Single-Carcinogen	9.594	0.310	4.988	0.045	Mild	Non-Irritant
**14o**	Single-Carcinogen	5.683	0.460	1.371	0.031	Mild	Non-Irritant
Sorafenib	Single-Carcinogen	19.236	0.089	0.823	0.005	Mild	Non-Irritant

[a] Unit: mg/kg body weight/day

[b] Unit: g/kg body weight

Injection Summary Report

SAMPLE INFORMATION

Sample Name:	CBA4 ammfo aceto 2575 f1 ph6	Acquired By:	System
Sample Type:	Unknown	Sample Set Name	
Vial:	80	Acq. Method Set:	sorafenib
Injection #:	3	Processing Metho	sorsfenib
Injection Volume:	2.00 ul	Channel Name:	262.9nm
Run Time:	30.0 Minutes	Proc. Chnl. Descr.:	W2996 PDA 262.9 nm (PDA
Date Acquired:	11/20/2021 12:01:29 PM EET		
Date Processed:	11/20/2021 3:08:11 PM EET		

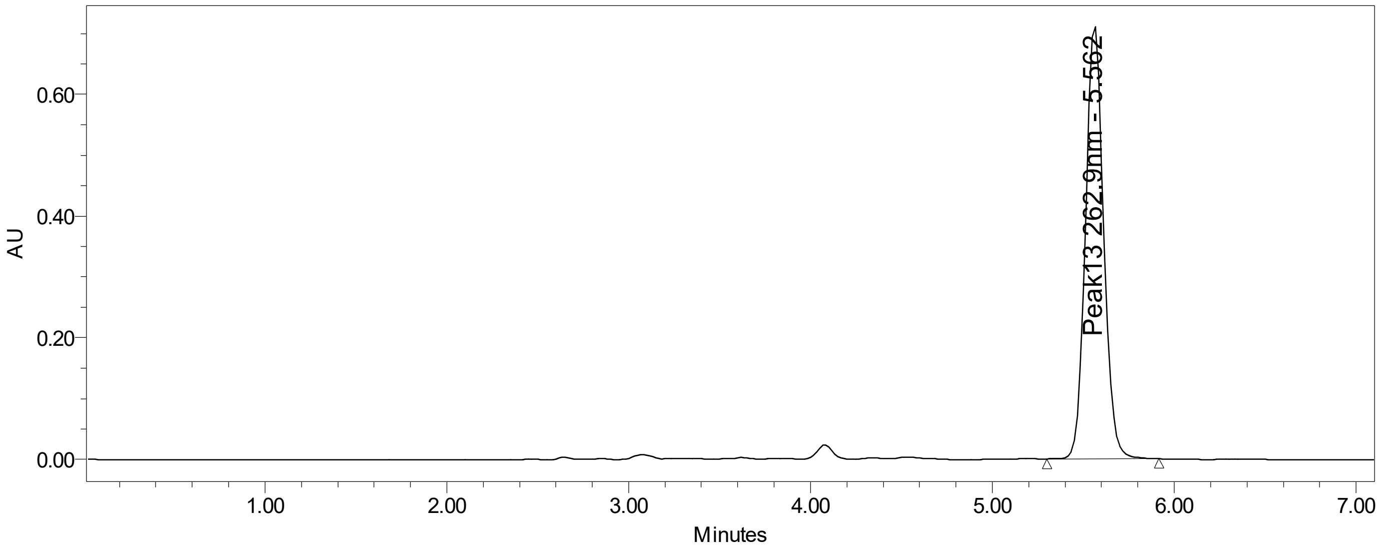

Channel: W2996 ; Processed Channel: W2996 PDA 262.9 nm (PDA 210.0 to 400.0 nm at 1.2 nm)
Result Id: 1127; Processing Method: sorsfenib

Peak Results

	Name	RT	Area	Height	Amount	Units
1	Peak13 262.9nm	5.562	4931742	710327		

PDA Result Table

	Name	RT	Purity1 Angle	Purity1 Threshold	Match1 Spect. Name	Match1 Angle	Match1 Threshold	Purity (Component)
1	Peak13 262.9nm	5.562	0.204	6.137				100.000

Purity Plot

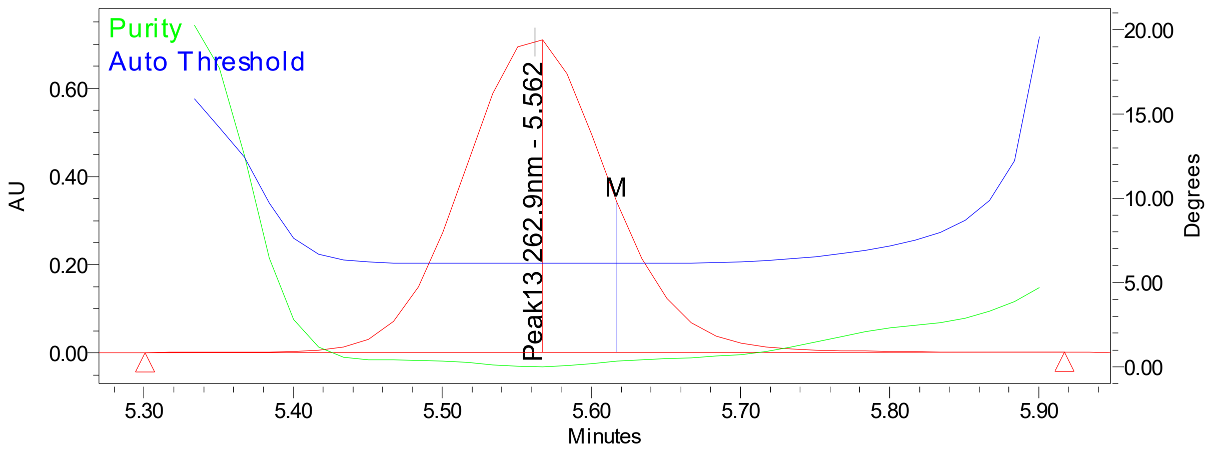

PA: 0.204 TH: 6.137

Peak: Peak13 262.9nm

Purity

Auto Threshold

Injection Summary Report

SAMPLE INFORMATION

Sample Name:	CBA15 ammf aceto 2575 f1 ph6	Acquired By:	System
Sample Type:	Unknown	Sample Set Name	
Vial:	82	Acq. Method Set:	sorafenib
Injection #:	1	Processing Metho	1
Injection Volume:	20.00 ul	Channel Name:	286.4nm
Run Time:	30.0 Minutes	Proc. Chnl. Descr.:	W2996 PDA 286.4 nm (PDA

Date Acquired: 11/21/2021 11:14:40 AM EET
Date Processed: 11/21/2021 1:31:30 PM EET

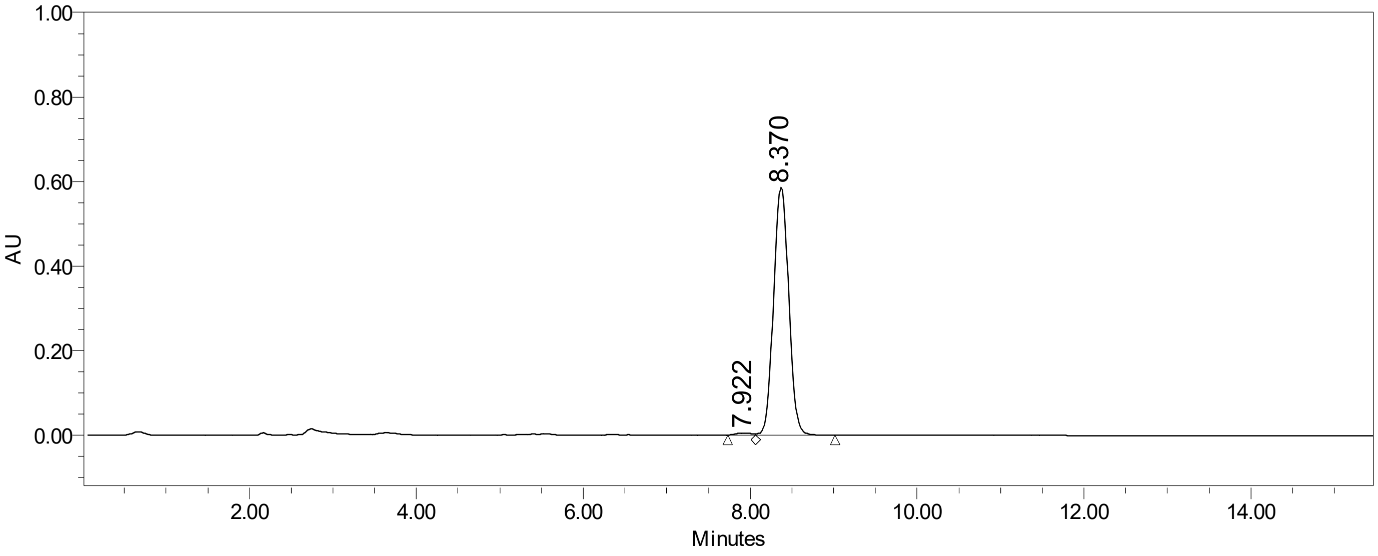

	Peak Name	RT	Area	% Area	Height	Purity (Component)
1	Peak1 286.4nm	7.922	65306	0.87	5095	100.000
2	Peak2 286.4nm	8.370	7411722	99.13	586369	100.000

3D Plot

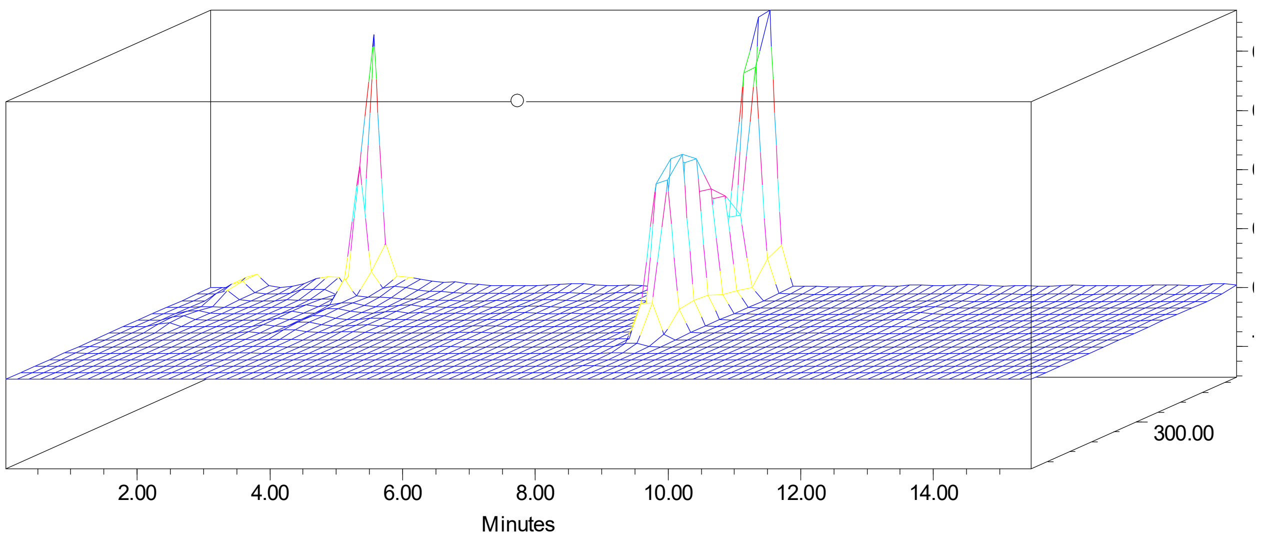

Multi Sample Summary

S A M P L E   I N F O R M A T I O N

Sample Name:	CBA23ammf aceto 2575 f1 ph6	Acquired By:	System
Sample Type:	Unknown	Sample Set Name	
Vial:	77	Acq. Method Set:	sorafenib
Injection #:	1	Processing Methoc	1
Injection Volume:	80.00 ul	Channel Name:	284.0nm
Run Time:	30.0 Minutes	Proc. Chnl. Descr.:	W2996 PDA 284.0 nm (PDA
Date Acquired:	11/21/2021 3:47:38 PM EET		
Date Processed:	11/24/2021 1:11:54 PM EET		

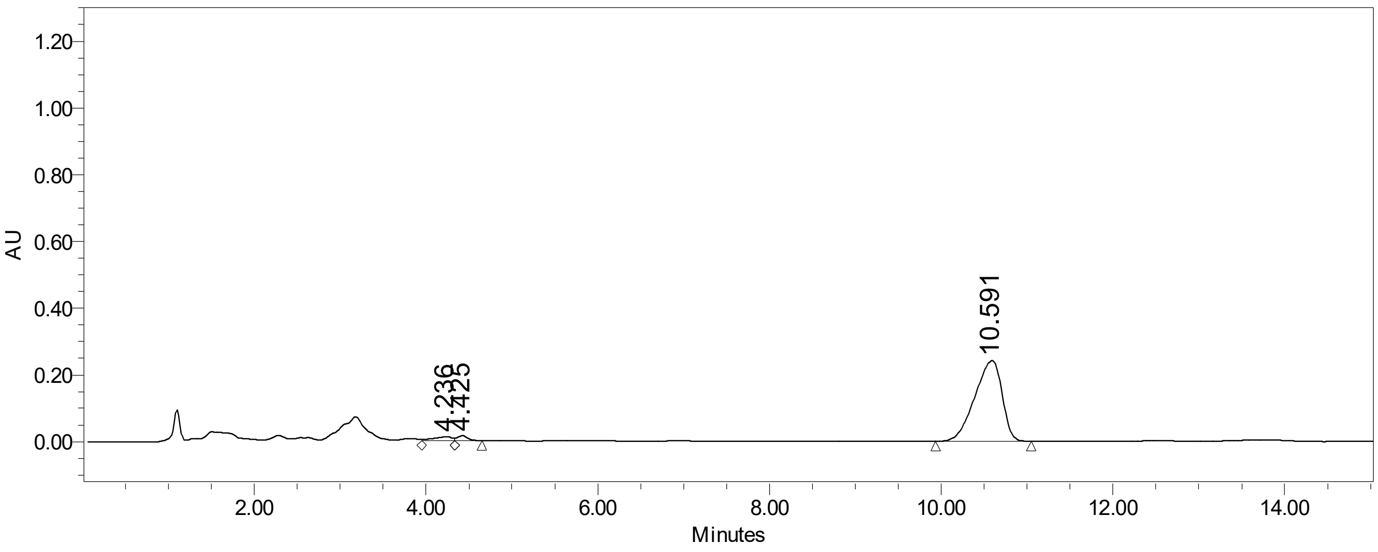

	RT	Area	% Area	Height
1	4.236	152234	2.70	10582
2	4.425	104530	1.86	13779
3	10.591	5373580	95.44	242278

3D Plot

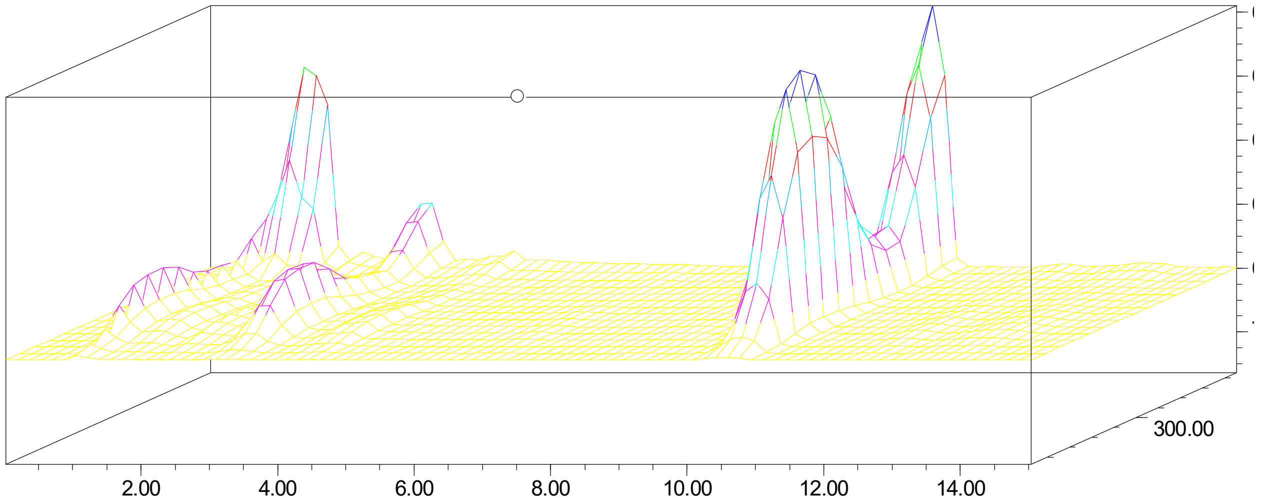

SAMPLE INFORMATION

Sample Name:	CBA33ammf aceto 2575 f1 ph6	Acquired By:	System
Sample Type:	Unknown	Sample Set Name	
Vial:	79	Acq. Method Set:	sorafenib
Injection #:	1	Processing Methoc	1
Injection Volume:	20.00 ul	Channel Name:	283.0nm
Run Time:	30.0 Minutes	Proc. Chnl. Descr.:	W2996 PDA 283.0 nm (PDA
Date Acquired:	11/21/2021 4:41:44 PM EET		
Date Processed:	11/24/2021 2:22:58 PM EET		

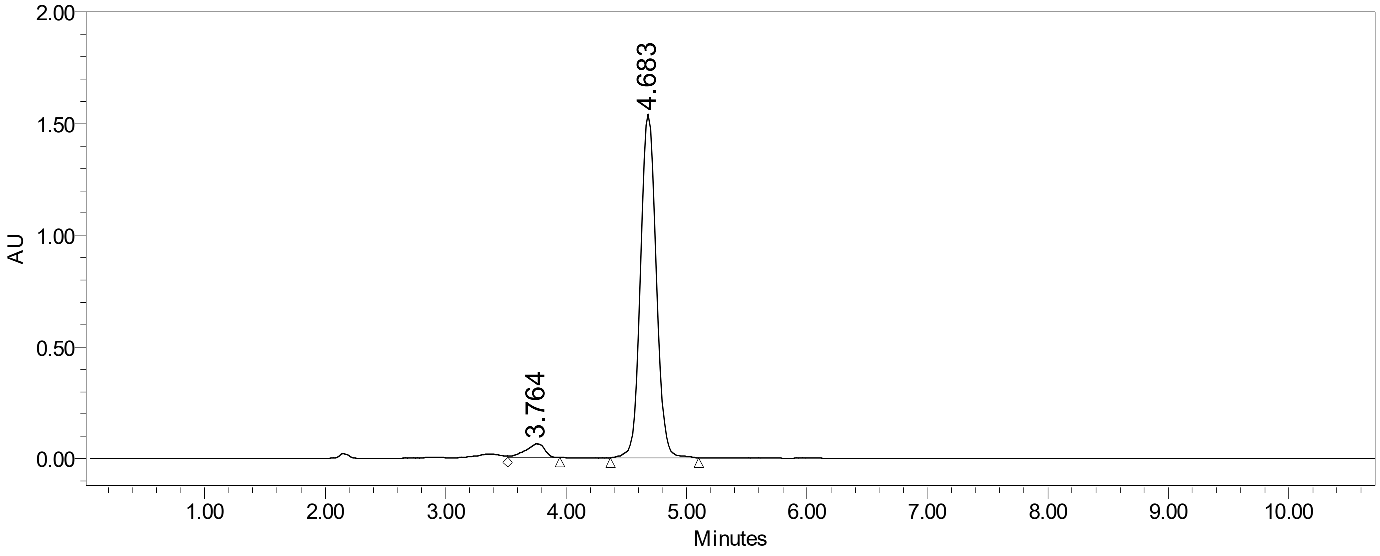

	RT	Area	% Area	Height
1	3.764	678079	4.63	61857
2	4.683	13974684	95.37	1535438

3D Plot

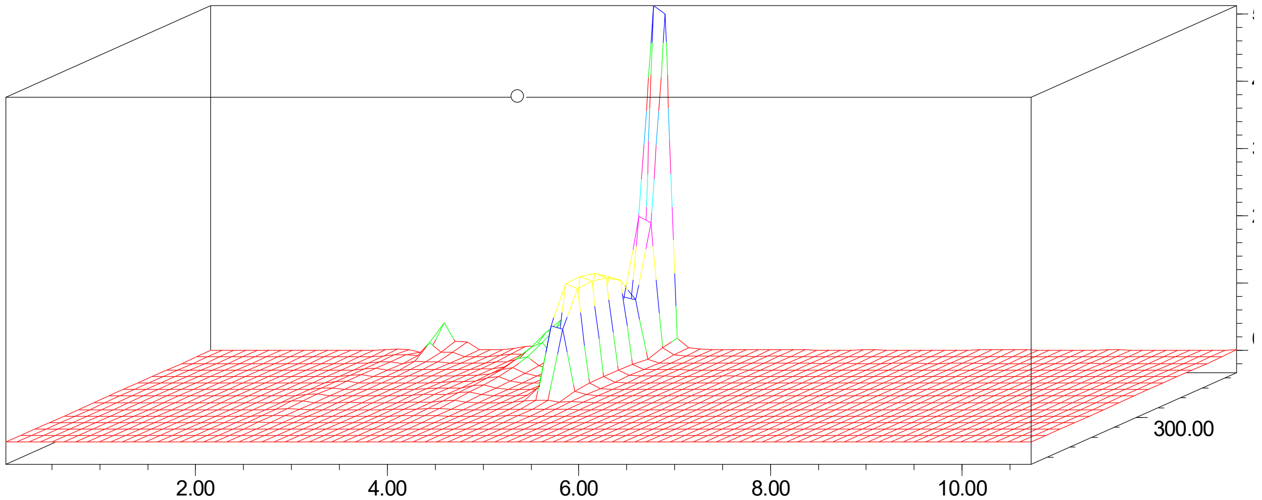

Injection Summary Report

SAMPLE INFORMATION

Sample Name:	CBA40 ammfacet 2575 1 ph6	Acquired By:	System
Sample Type:	Unknown	Sample Set Name	
Vial:	113	Acq. Method Set:	sorafenib
Injection #:	1	Processing Metho	1
Injection Volume:	3.00 ul	Channel Name:	283.0nm
Run Time:	30.0 Minutes	Proc. Chnl. Descr.:	W2996 PDA 283.0 nm (PDA
Date Acquired:	11/24/2021 2:04:45 PM EET		
Date Processed:	11/24/2021 2:21:10 PM EET		

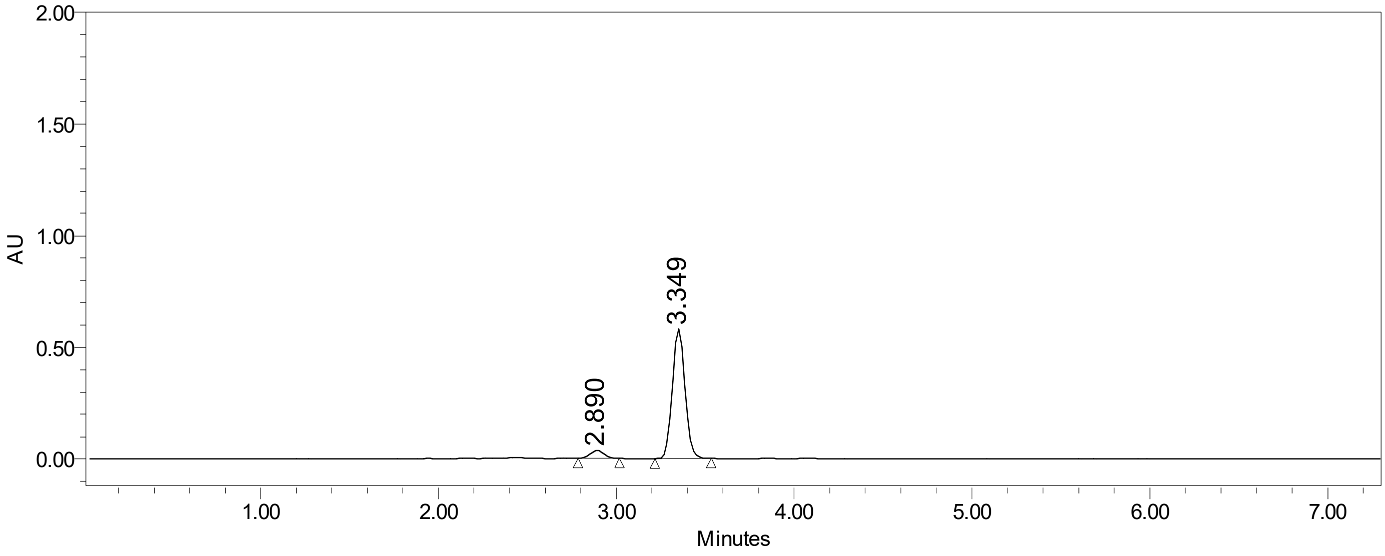

	RT	Area	% Area	Height
1	2.890	184133	6.00	35016
2	3.349	2884496	94.00	571693

3D Plot

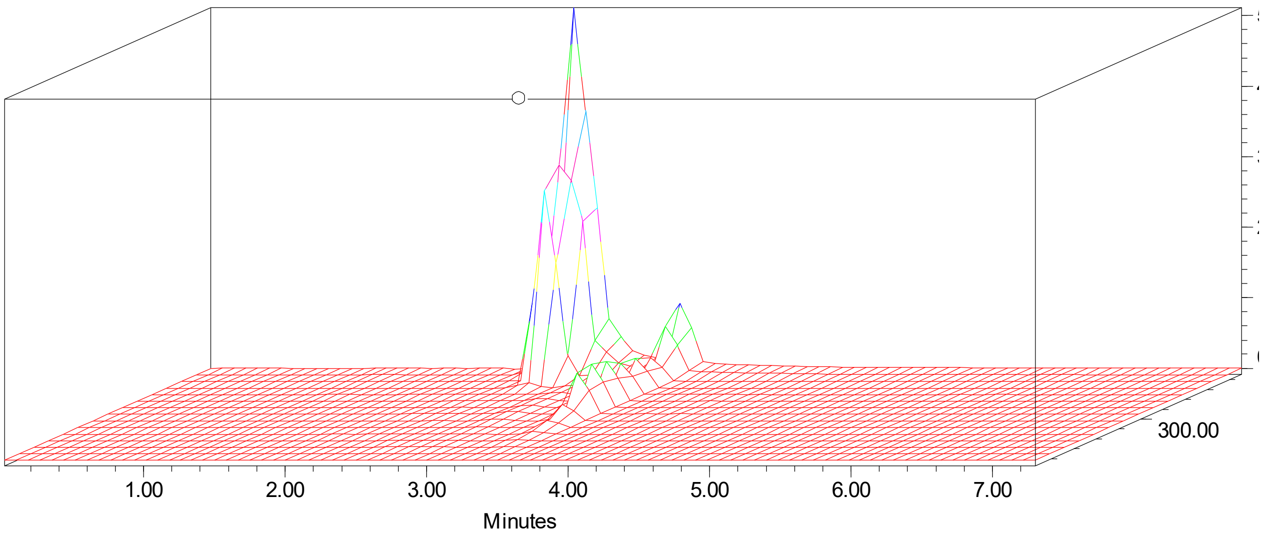

Multi Sample Summary

S A M P L E   I N F O R M A T I O N

Sample Name:	MBA4 ammf aceto 2575 f1 ph6	Acquired By:	System
Sample Type:	Unknown	Sample Set Name	
Vial:	83	Acq. Method Set:	sorafenib
Injection #:	1	Processing Metho	1
Injection Volume:	20.00 ul	Channel Name:	284.0nm
Run Time:	30.0 Minutes	Proc. Chnl. Descr.:	W2996 PDA 284.0 nm (PDA
Date Acquired:	11/21/2021 1:41:27 PM EET		
Date Processed:	11/21/2021 1:58:33 PM EET		

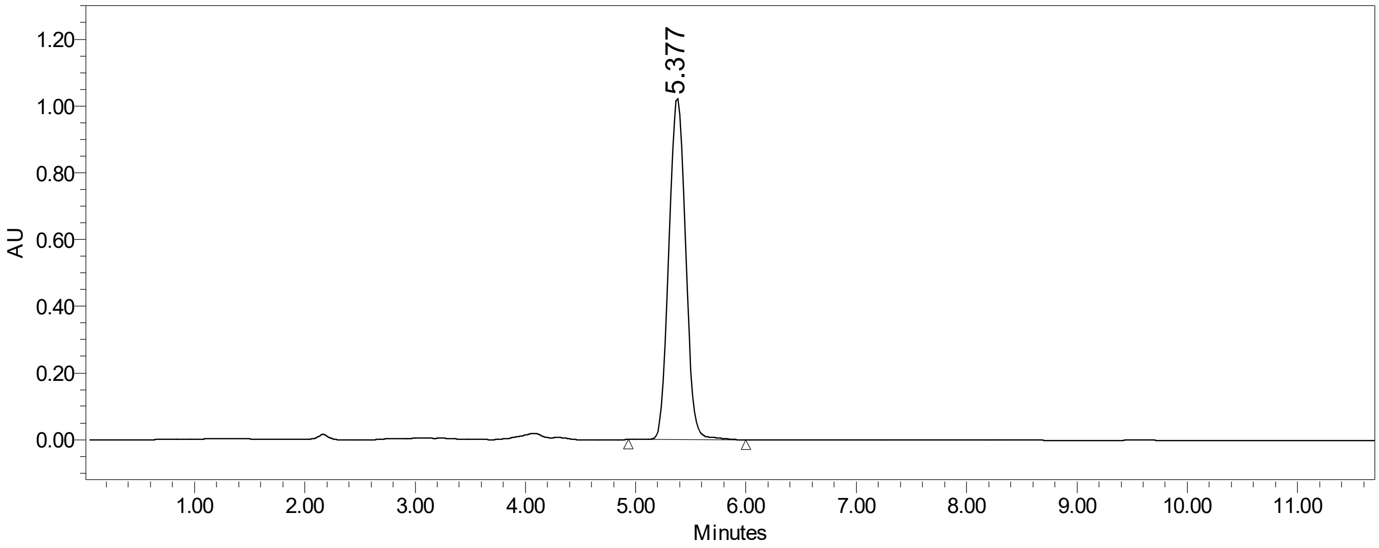

	Peak Name	RT	Area	% Area	Height	Purity (Component)
1	Peak3 284.0nm	5.377	10831487	100.00	1026326	100.000

3D Plot

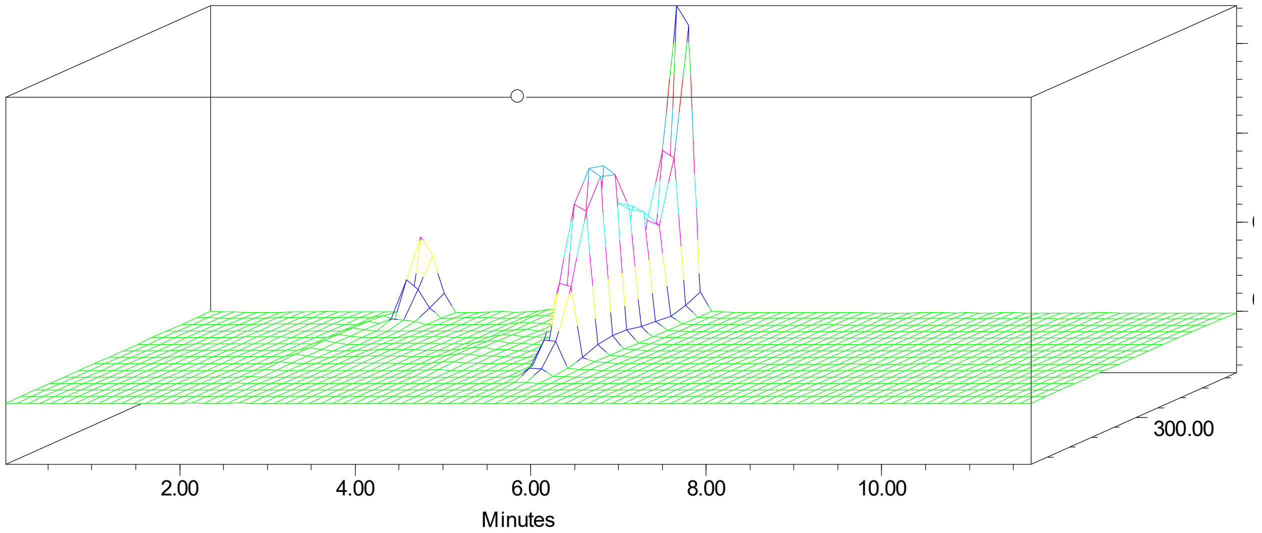

Multi Sample Summary

SAMPLE INFORMATION

Sample Name:	MBA15 ammf 2575 2 ph6 pda	Acquired By:	System
Sample Type:	Unknown	Sample Set Name	
Vial:	111	Acq. Method Set:	sorafenib
Injection #:	1	Processing Methoo	1
Injection Volume:	20.00 ul	Channel Name:	286.4nm
Run Time:	30.0 Minutes	Proc. Chnl. Descr.:	W2996 PDA 286.4 nm (PDA

Date Acquired: 11/24/2021 1:11:25 PM EET
Date Processed: 11/24/2021 1:21:34 PM EET

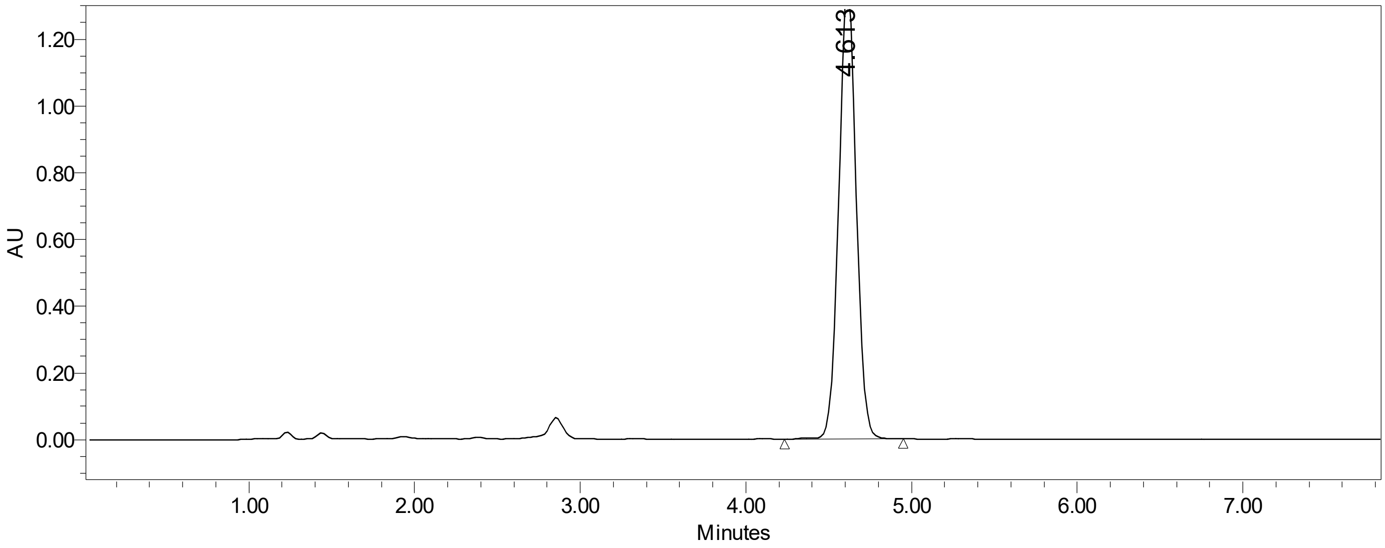

	RT	Area	% Area	Height
1	4.613	9928278	100.00	1361322

3D Plot

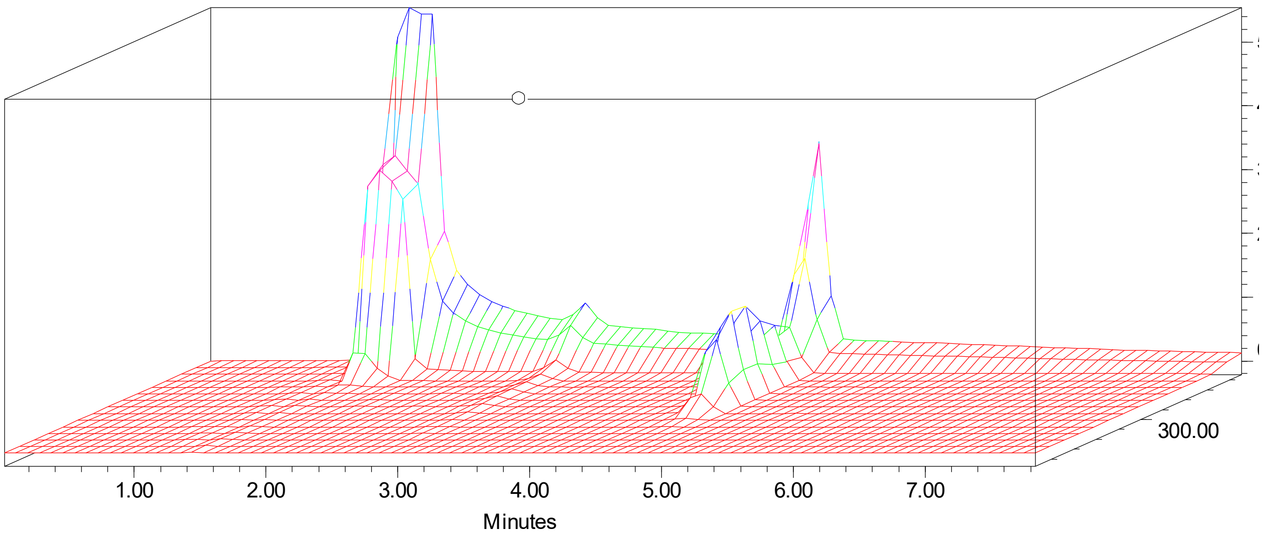

Injection Summary Report

SAMPLE INFORMATION

Sample Name:	MBA23ammf 2575 f1.2ph6 pda	Acquired By:	System
Sample Type:	Unknown	Sample Set Name	
Vial:	110	Acq. Method Set:	sorafenib
Injection #:	3	Processing Methoo	1
Injection Volume:	10.00 ul	Channel Name:	283.0nm
Run Time:	30.0 Minutes	Proc. Chnl. Descr.:	W2996 PDA 283.0 nm (PDA
Date Acquired:	11/24/2021 12:06:22 PM EET		
Date Processed:	11/24/2021 2:28:29 PM EET		

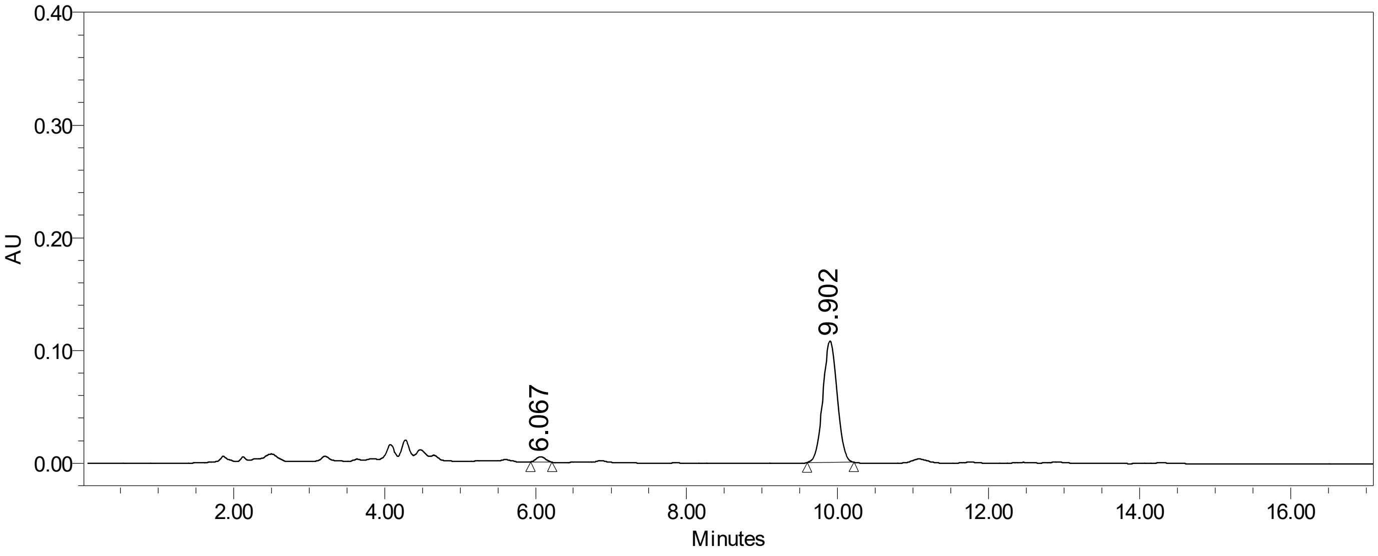

	RT	Area	% Area	Height
1	6.067	39142	2.67	4748
2	9.902	1425898	97.33	107918

3D Plot

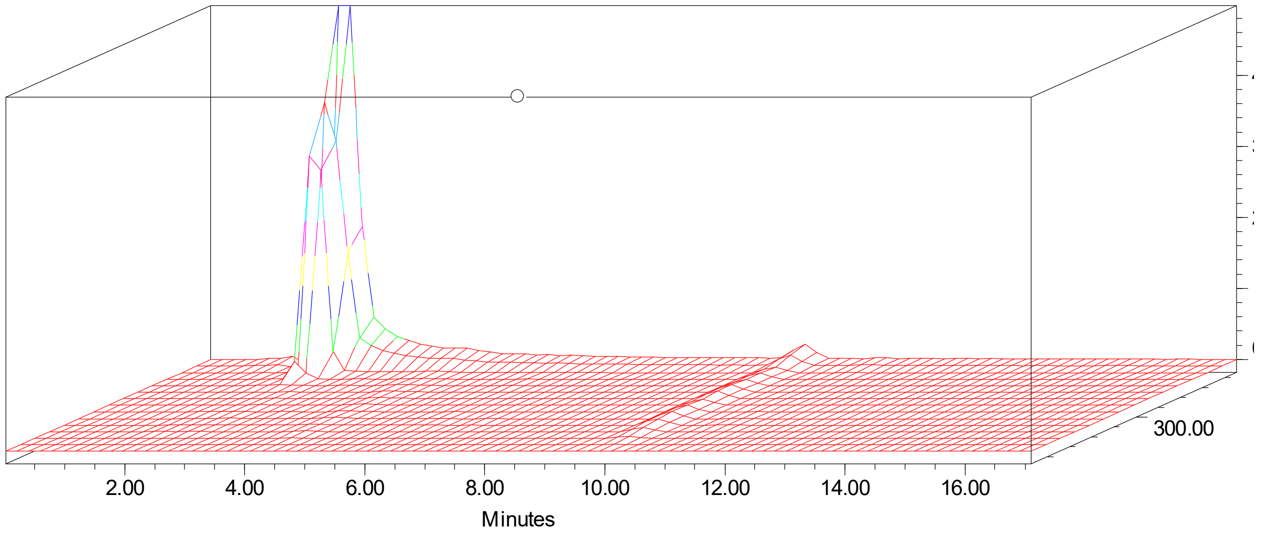

Multi Sample Summary

SAMPLE INFORMATION

Sample Name:	MBA23ammf aceto 2575 f1 ph6	Acquired By:	System
Sample Type:	Unknown	Sample Set Name	
Vial:	78	Acq. Method Set:	sorafenib
Injection #:	1	Processing Metho	1
Injection Volume:	20.00 ul	Channel Name:	286.4nm
Run Time:	30.0 Minutes	Proc. Chnl. Descr.:	W2996 PDA 286.4 nm (PDA
Date Acquired:	11/21/2021 4:09:59 PM EET		
Date Processed:	11/24/2021 1:07:15 PM EET		

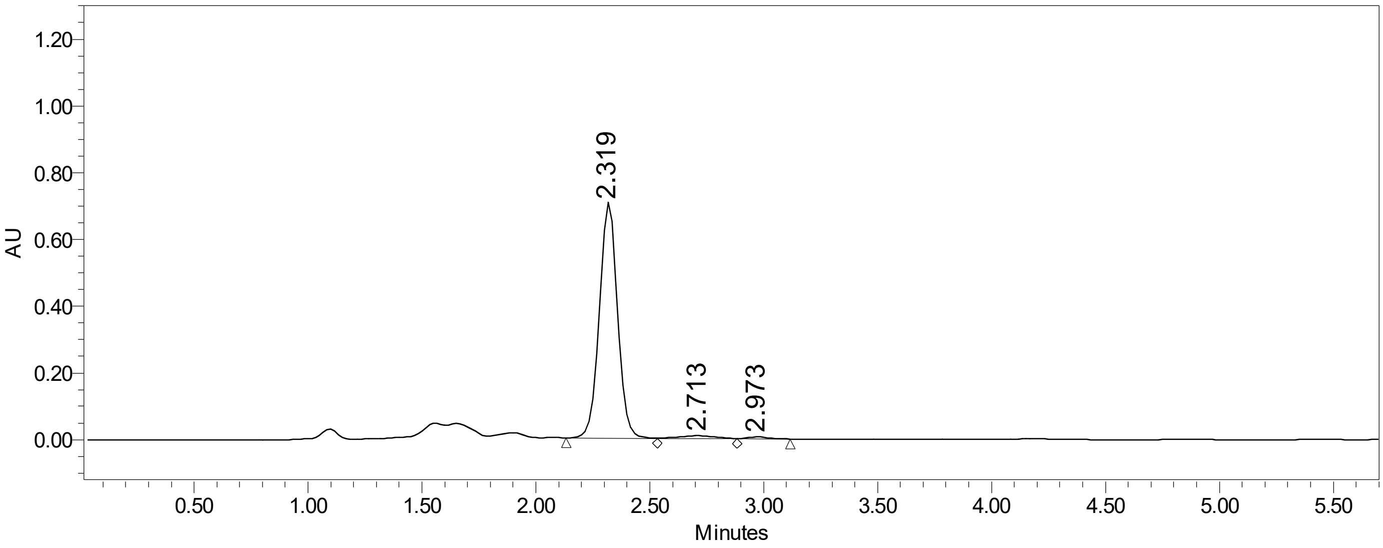

	RT	Area	% Area	Height
1	2.319	3955046	96.77	700659
2	2.713	95940	2.35	8579
3	2.973	35959	0.88	5653

3D Plot

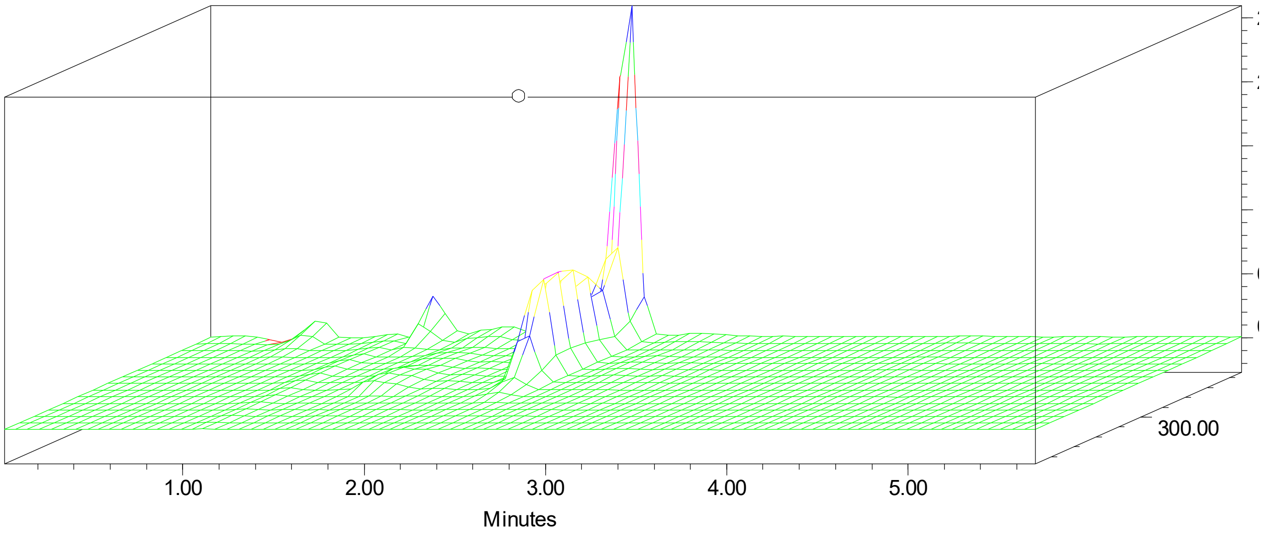

Injection Summary Report

SAMPLE INFORMATION

Sample Name:	MBA40ammf 2575 1 ph6 pda	Acquired By:	System
Sample Type:	Unknown	Sample Set Name	
Vial:	112	Acq. Method Set:	sorafenib
Injection #:	1	Processing Methoc	1
Injection Volume:	5.00 ul	Channel Name:	283.0nm
Run Time:	30.0 Minutes	Proc. Chnl. Descr.:	W2996 PDA 283.0 nm (PDA
Date Acquired:	11/24/2021 1:41:05 PM EET		
Date Processed:	11/24/2021 2:06:37 PM EET		

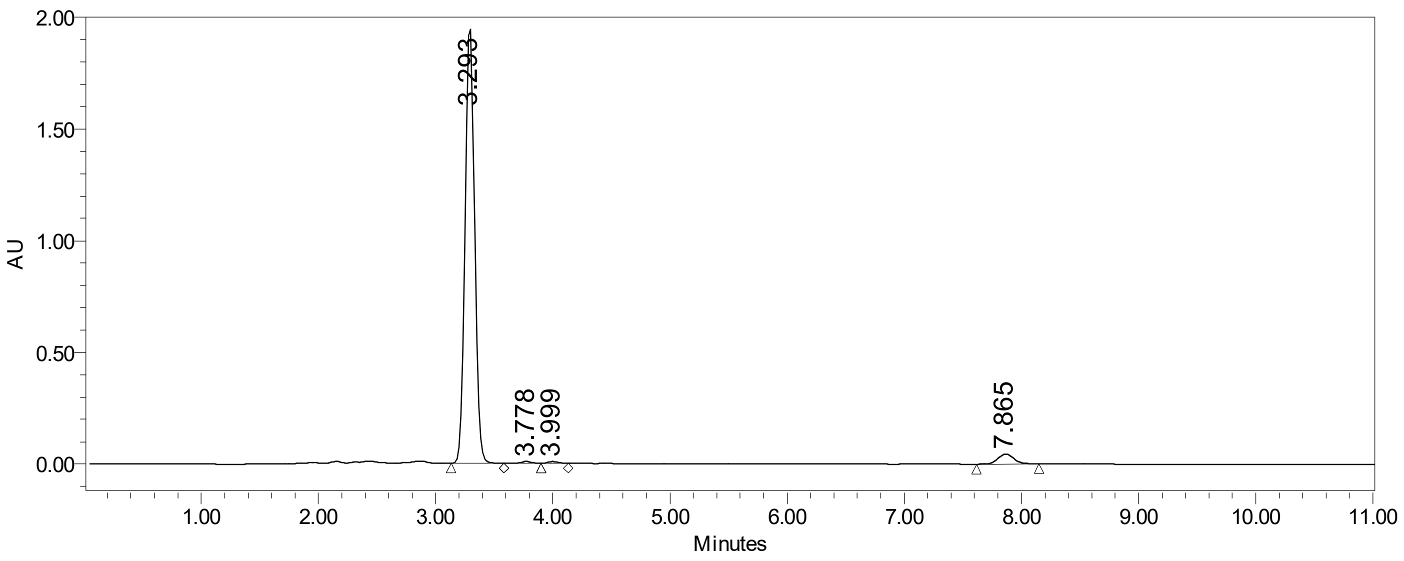

	RT	Area	% Area	Height
1	3.293	10768300	95.16	1948113
2	3.778	60084	0.53	9257
3	3.999	55593	0.49	8296
4	7.865	432265	3.82	44730

3D Plot

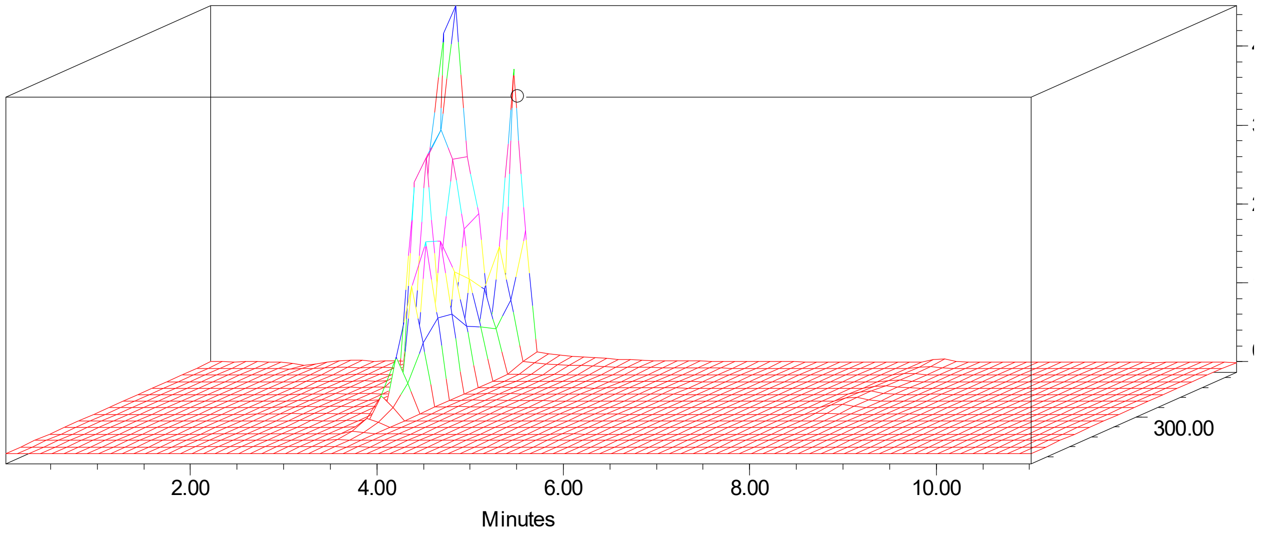

Empower™ 3 SOFTWARE

Multi Sample Summary

SAMPLE INFORMATION

Sample Name:	PBA4 ammf aceto 2575 f1 ph6	Acquired By:	System
Sample Type:	Unknown	Sample Set Name	
Vial:	75	Acq. Method Set:	sorafenib
Injection #:	1	Processing Methoc	1
Injection Volume:	10.00 ul	Channel Name:	286.4nm
Run Time:	30.0 Minutes	Proc. Chnl. Descr.:	W2996 PDA 286.4 nm (PDA
Date Acquired:	11/21/2021 2:03:07 PM EET		
Date Processed:	11/24/2021 12:55:51 PM EET		

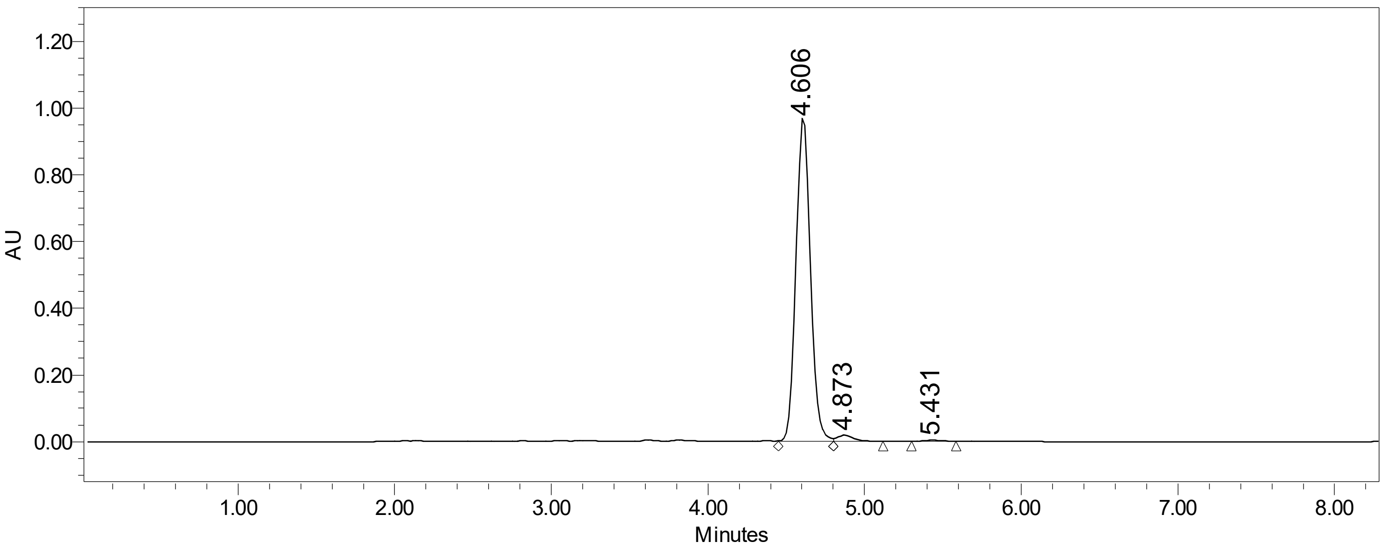

	RT	Area	% Area	Height
1	4.606	6185433	97.50	971333
2	4.873	134645	2.12	18715
3	5.431	23861	0.38	3429

3D Plot

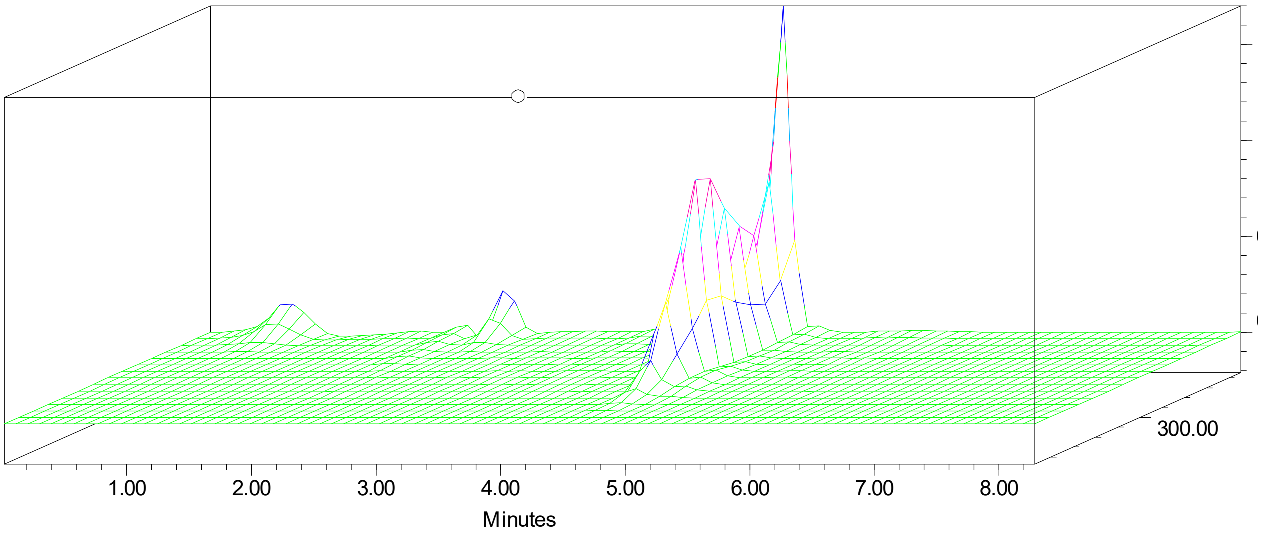

Multi Sample Summary

S A M P L E   I N F O R M A T I O N

Sample Name:	PBA15 ammf aceto 2575 f1 ph6	Acquired By:	System
Sample Type:	Unknown	Sample Set Name	
Vial:	76	Acq. Method Set:	sorafenib
Injection #:	1	Processing Metho	1
Injection Volume:	10.00 ul	Channel Name:	284.0nm
Run Time:	30.0 Minutes	Proc. Chnl. Descr.:	W2996 PDA 284.0 nm (PDA
Date Acquired:	11/21/2021 2:43:30 PM EET		
Date Processed:	11/24/2021 1:05:42 PM EET		

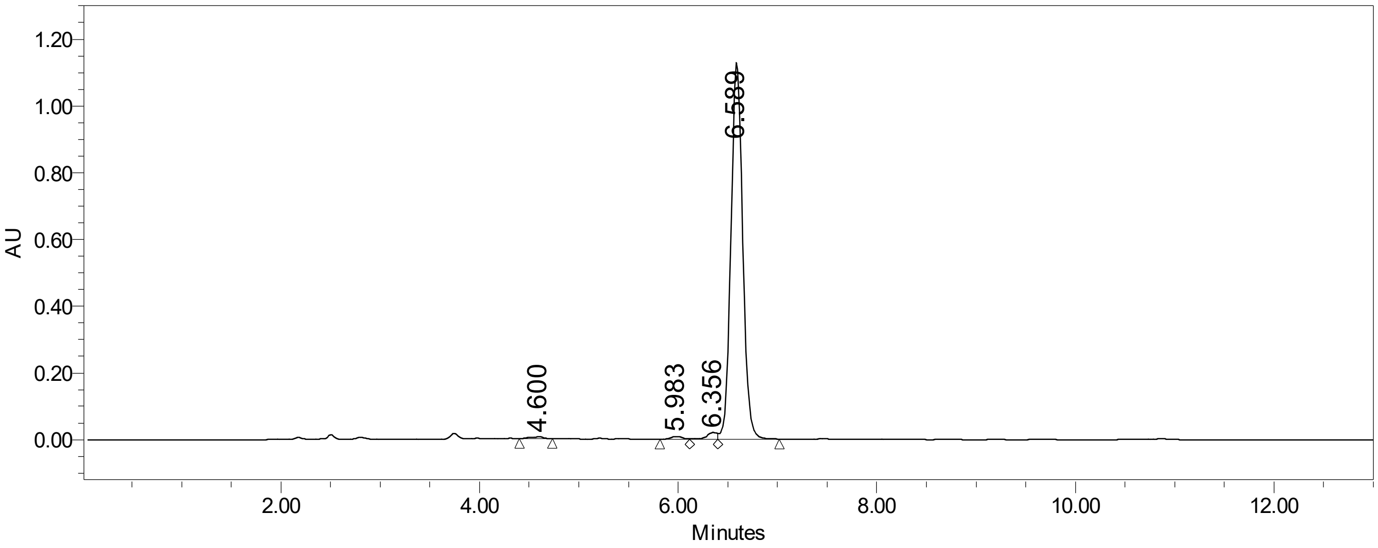

	RT	Area	% Area	Height
1	4.600	60732	0.64	5854
2	5.983	68471	0.72	8892
3	6.356	143043	1.50	20542
4	6.589	9234564	97.14	1130648

3D Plot

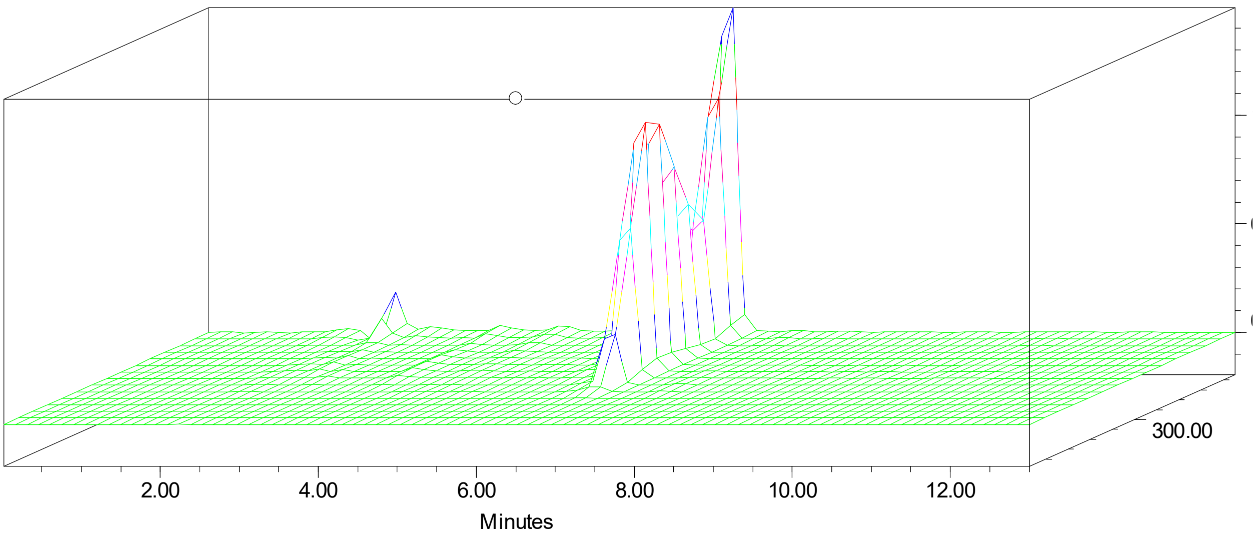

Injection Summary Report

S A M P L E   I N F O R M A T I O N

Sample Name:	PBA33old ammfacet 2575 1	Acquired By:	System
Sample Type:	Unknown	Sample Set Name	
Vial:	95	Acq. Method Set:	sorafenib
Injection #:	1	Processing Metho	1
Injection Volume:	3.00 ul	Channel Name:	283.0nm
Run Time:	30.0 Minutes	Proc. Chnl. Descr.:	W2996 PDA 283.0 nm (PDA
Date Acquired:	11/24/2021 1:54:36 PM EET		
Date Processed:	11/24/2021 2:07:07 PM EET		

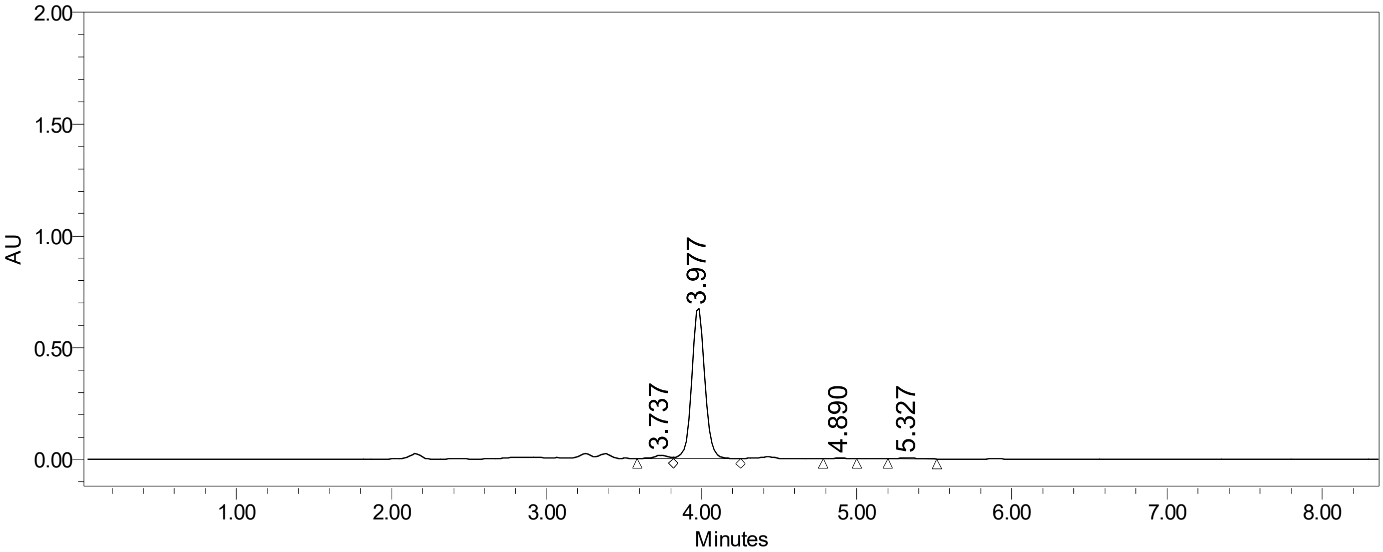

	RT	Area	% Area	Height
1	3.737	88260	2.13	14683
2	3.977	3991768	96.25	671786
3	4.890	24022	0.58	4361
4	5.327	43318	1.04	5423

3D Plot

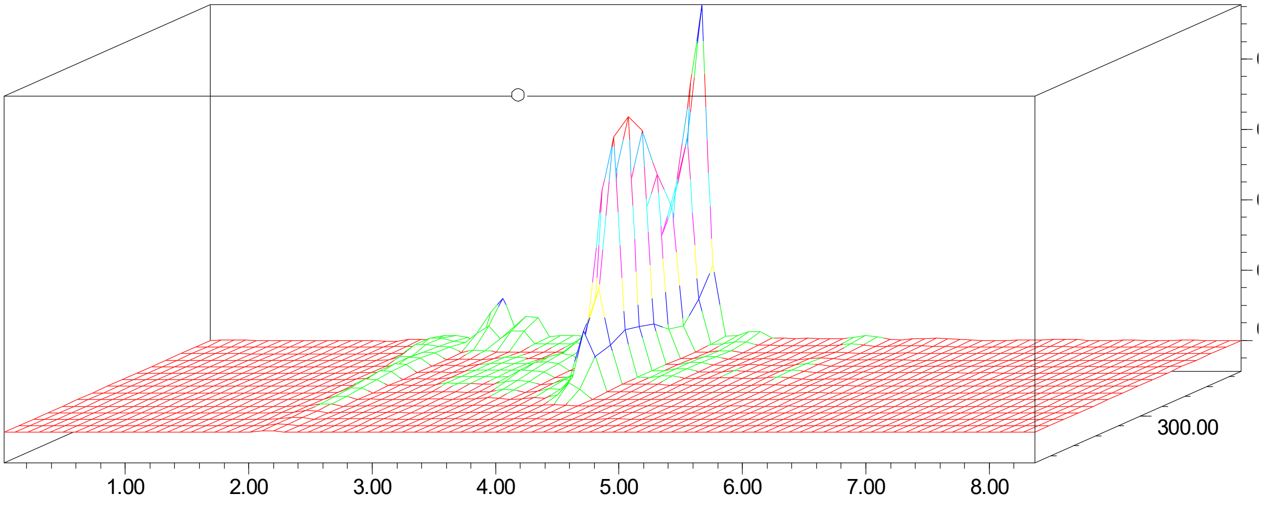

Supplement: Supplemental Material [file IENZ_A_2015343_SM4405.pdf]
